# The Association Between Serum *α*1‐Acid Glycoprotein and Obesity and Abdominal Obesity in Women: A Cross‐Sectional Study Based on NHANES Data From 2015 to 2018

**DOI:** 10.1155/ije/1513929

**Published:** 2026-01-30

**Authors:** Ling Sun, Lingyan He, Hao Zhang, Bruno Fink, Haihua Pan, Changlin Zhai

**Affiliations:** ^1^ Zhejiang Chinese Medical University, Hangzhou, Zhejiang, China, zcmu.edu.cn; ^2^ Department of Cardiology, The First Hospital of Jiaxing Affiliated Hospital of Jiaxing University, Jiaxing, Zhejiang, China; ^3^ Heart and Vascular Center Rhein-Nahe-Pfalz, Landkreis, Bad Kreuznach, Germany; ^4^ Noxygen Science Transfer & Diagnostics GmbH, Elzach, Germany

**Keywords:** abdominal obesity, inflammation, metabolic syndrome, NHANES, obesity, serum *α*1-acid glycoprotein

## Abstract

**Background:**

Obesity and abdominal obesity are major public health issues closely related to metabolic diseases. Serum *α*1‐acid glycoprotein (SSAGP), an acute‐phase reactant influenced by inflammation and metabolic status, has an unclear relationship with obesity and abdominal obesity. This study investigates this association in women.

**Methods:**

Using cross‐sectional data from NHANES (2015–2018), 2219 adult women were divided into three groups based on SSAGP levels (low, medium, and high). Multiple regression analyses assessed the relationship between SSAGP and BMI, waist circumference, obesity (BMI ≥ 30 kg/m^2^), and abdominal obesity (waist circumference ≥ 90 cm). Threshold and interaction analyses were also conducted.

**Results:**

As SSAGP levels increased, BMI, waist circumference, fasting blood glucose, insulin, and hs‐CRP levels rose significantly (*p* < 0.001), while HDL levels decreased (*p* < 0.001). SSAGP was positively correlated with BMI, waist circumference, obesity, and abdominal obesity (*p* < 0.0001). After adjusting for confounders, a one‐unit increase in SSAGP was associated with a 4.42 increase in BMI (95% CI: 3.08, 5.76), a 12.18 cm increase in waist circumference (95% CI: 9.22, 15.14), a 3.63‐fold increase in obesity risk (95% CI: 1.96, 6.72), and a 10.75‐fold increase in abdominal obesity risk (95% CI: 4.85, 23.85). Threshold effect analysis showed an inflection point (*K* = 1.2), with SSAGP having a stronger promoting effect below this point and an inhibitory effect above it (*p* < 0.001). Educational level significantly influenced the SSAGP‐obesity relationship (*p* = 0.0096).

**Conclusion:**

SSAGP levels are significantly associated with obesity and abdominal obesity in women, with educational level playing a modulatory role. SSAGP may serve as a potential biomarker for obesity risk. Future studies should explore the causal relationships and underlying mechanisms.

## 1. Introduction

Obesity and abdominal obesity are significant public health issues worldwide. According to the World Health Organization (WHO), obesity is a leading risk factor for cardiovascular diseases, type 2 diabetes, certain cancers, and chronic respiratory diseases [1]. As a specific type of obesity, abdominal obesity is closely linked to an increased risk of metabolic syndrome [2, 3], cardiovascular diseases [4], nonalcoholic fatty liver disease (NAFLD) [5], type 2 diabetes [6, 7], and certain cancers [8]. Considering the severity of health impacts and the increasing trend in the prevalence of obesity and abdominal obesity, deep research on its related factors and underlying mechanisms should be conducted.

Serum *α*1‐acid glycoprotein (SSAGP) is an acute‐phase reactant synthesized by the liver. It has a variety of biological functions, including drug transport, immune regulation, maintenance of capillary barrier function, and involvement in lipid metabolism [9, 10]. The levels of SSAGP are significantly increased in conditions such as inflammation, infection, autoimmune diseases (e.g., rheumatoid arthritis), and metabolic disorders (e.g., diabetes) [11, 12]. Moreover, SSAGP is abnormally expressed in the tumor microenvironment and is associated with tumor progression and metastasis [13–15]. Clinical application: Detection of SSAGP in clinical practice mainly serves the purpose of early diagnosis of inflammation and infection, and also for the estimation of disease activity, such as rheumatoid arthritis [16]. Further research is in progress to identify its value as a potential biomarker, mainly in metabolic diseases and oncology.

Recent studies have found that SSAGP is highly associated with metabolic disorders in obesity. For instance, a high level of SSAGP is associated with a higher percentage of body fat, which indicates that it may play an important role in the development and progression of obesity [17]. Besides, the levels of SSAGP are significantly higher in metabolic syndrome patients [18] and are closely related to relevant clinical risk factors [17], which indicates that SSAGP may be important in metabolic disorders. However, current research on the specific role of SSAGP in obesity, particularly abdominal obesity, remains relatively limited. Its function in the progression of obesity and the related underlying mechanisms still require further elucidation. This present study will investigate the association of SSAGP with obesity and abdominal obesity among female participants in order to supplement the literature on the subject. Thus, the work will be presenting hypothesized correlations of SSAGP levels with selected indicators of obesity, namely BMI and waist circumference, and explore modulating effects related to the participants’ socioeconomic characteristics, such as education level. It will not only provide a certain understanding of the possible mechanisms of SSAGP in the development of obesity but also offer a new biomarker for the early identification and intervention of obesity. Moreover, this study will lay a scientific foundation for optimizing strategies in the management of obesity in clinical practice, especially for the identification of high‐risk populations and the development of personalized measures for intervention.

## 2. Methods

### 2.1. Data Source and Study Sample

The data for this study were derived from the cross‐sectional surveys of NHANES conducted between 2015 and 2018. NHANES is a nationally representative study organized and carried out by NCHS that focuses on the assessment of health and nutritional status in adults and children of the United States. Data used for this research are from https://wwwn.cdc.gov/nchs/nhanes/default.aspx.

**FIGURE 1:**
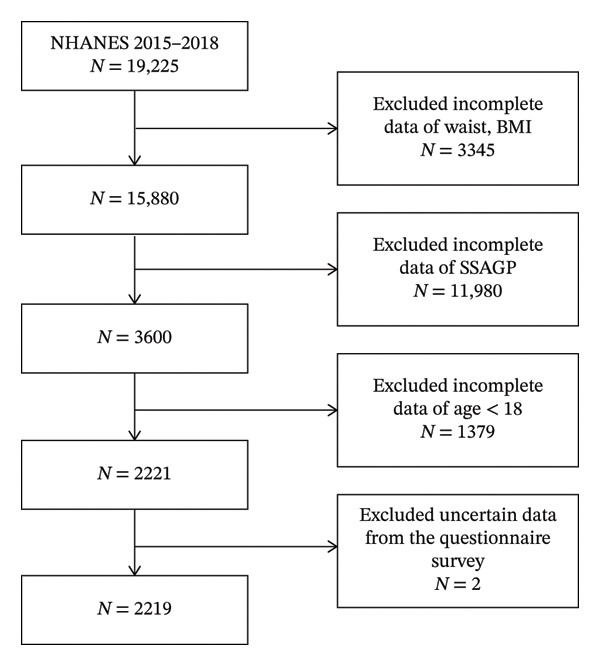
Study flowchart. NHANES, national health and nutrition examination surveys.

In this study, participants were selected as women aged ≥ 18 years from the database of NHANES between 2015 and 2018. Participants were excluded in the study based on the following conditions: (1) missing data regarding the level of SSAGP; (2) absent key indicators, including the following: BMI, waist circumference, blood lipids, and blood glucose; (3) participants declared as pregnant or lactating. A total of 2219 female participants were included in this final analysis (Figure 1).

### 2.2. Exposure and Outcome Variables

Exposure variable: SSAGP level was the primary exposure variable in this study. SSAGP levels were obtained through laboratory tests in NHANES, measured by immunoturbidimetry to determine the concentration of SSAGP in serum samples.

Outcome variable: Obesity is defined as BMI ≥ 30 kg/m^2^ [19]. Abdominal obesity in women is defined as waist circumference ≥ 80 cm [20].

BMI and waist circumference were analyzed as continuous variables to assess the linear relationship between SSAGP and obesity‐related indicators.

### 2.3. Covariate Selection

Based on literature review and clinical significance, the following covariates were selected: age, race/ethnicity (Mexican American, other Hispanic, non‐Hispanic White, non‐Hispanic Black, non‐Hispanic Asian, and other races), education level (< 9th grade, 9–11th grade, high school graduate/GED, some college/associate degree, college graduate, or above), alcohol use (yes/no), smoking status (every day, some days, not at all), hypertension (yes/no), diabetes (yes/no), moderate physical activity (yes/no), high‐sensitivity C‐reactive protein (hs‐CRP), fasting blood glucose (FBG), insulin levels, high‐density lipoprotein cholesterol (HDL‐C), triglycerides (TG), and glycated hemoglobin (HbA1c).

The selection of covariates was based on the following rationale: (1) Demographic and socioeconomic factors: Age and race/ethnicity represented fundamental demographic variables. Education level, as a socioeconomic indicator, was associated with health behaviors, healthcare access, and chronic disease risks, potentially influencing obesity‐related outcomes through multiple pathways [21]. (2) Behavioral and lifestyle factors: Alcohol use, smoking status, and physical activity were modifiable lifestyle factors. Physical activity directly affected energy expenditure and metabolic health, while smoking and alcohol consumption could influence obesity through pathways such as inflammation and endocrine regulation [22, 23]. (3) Clinical and metabolic factors: Hypertension and diabetes were included to control for comorbidity confounding [24, 25]. hs‐CRP served as a systemic inflammation marker linked to obesity and SSAGP. FBG, insulin, and HbA1c assessed glycemic status, while HDL‐C and TG reflected lipid metabolism profiles. These covariates were obtained through questionnaires or laboratory tests.

### 2.4. Statistical Analysis

Descriptive statistics: Continuous variables were described using mean ± standard deviation (SD) or median (interquartile range), while categorical variables were described using frequencies (percentages). Comparisons between different SSAGP level groups were performed using analysis of variance (ANOVA) or chi‐square tests. Multiple regression analysis: Linear regression was used to assess the association between SSAGP and BMI or waist circumference, while logistic regression was employed to evaluate the association between SSAGP and obesity or abdominal obesity. The analyses were conducted in three models: Model 1 was unadjusted; Model 2 was adjusted for age and race/ethnicity; Model 3 was further adjusted for insulin, HDL‐C, TG, HbA1c, and hs‐CRP. Threshold effect analysis: Piecewise linear regression models were used to evaluate the nonlinear relationship between SSAGP and BMI or waist circumference, identifying potential inflection points (*K* values). Interaction analysis: The interactions between SSAGP and factors such as education level, alcohol use, smoking status, hypertension, diabetes, and moderate physical activity on obesity and abdominal obesity were assessed. All statistical analyses were performed using Empower RCH 4.2, with a *p* value of < 0.05 considered statistically significant.

## 3. Results

### 3.1. Characteristics of Participants

A total of 2219 female participants were included in this study and were divided into three groups based on their SSAGP levels: low‐level group (0.54 ± 0.09), medium‐level group (0.76 ± 0.06), and high‐level group (1.06 ± 0.16). The results showed that SSAGP levels were significantly correlated with various physiological and metabolic indicators. As SSAGP levels increased, participants exhibited a decrease in the poverty‐income ratio (PIR) (*p* < 0.001) and HDL levels (*p* < 0.001), while FBG and insulin levels increased (*p* < 0.001). Additionally, low‐density lipoprotein (LDL) and TG levels increased (*p* < 0.001), total cholesterol (TC) levels slightly increased (*p* = 0.002), glycated HbA1c levels increased (*p* < 0.001), and hs‐CRP levels significantly increased (*p* < 0.001). Moreover, SSAGP levels were significantly correlated with age (*p* < 0.001), BMI (*p* < 0.001), waist circumference (*p* < 0.001), and education level (*p* < 0.001), but not significantly associated with alcohol use (*p* = 0.371) or hypertension (*p* = 0.353) (Table 1).

**TABLE 1:** Demographic characteristics of the study participants.

SSAGP tertile	Low (0.54 ± 0.09)	Middle (0.76 ± 0.06)	High (1.06 ± 0.16)	*p* value
*N*	740	739	740	
PIR	2.62 ± 1.61	2.35 ± 1.55	2.09 ± 1.47	< 0.001
HDL (mmol/L)	1.65 ± 0.39	1.45 ± 0.38	1.29 ± 0.37	< 0.001
FBG (mmol/L)	5.39 ± 0.77	5.69 ± 1.43	5.92 ± 1.52	< 0.001
INSULIN (pmol/L)	58.28 ± 42.54	78.99 ± 67.66	107.57 ± 69.26	< 0.001
LDL (mmol/L)	2.59 ± 0.70	2.79 ± 0.70	2.88 ± 0.71	< 0.001
TG (mmol/L)	0.85 ± 0.51	1.04 ± 0.59	1.23 ± 0.60	< 0.001
TC (mmol/L)	4.59 ± 0.95	4.70 ± 0.87	4.76 ± 0.98	0.002
HBA1C, %	5.28 ± 0.58	5.47 ± 0.90	5.64 ± 0.93	< 0.001
hs‐CRP (mg/L)	2.00 ± 3.55	3.01 ± 3.49	8.45 ± 10.61	< 0.001
AGE, years	31.61 ± 9.21	33.75 ± 9.14	34.46 ± 9.27	< 0.001
BMI (kg/m^2^)	25.15 ± 5.68	29.49 ± 6.99	34.39 ± 8.53	< 0.001
WAIST (cm)	85.49 ± 13.82	95.40 ± 15.83	107.48 ± 18.27	< 0.001
RACE				< 0.001
Mexican American	114 (15.41%)	160 (21.65%)	133 (17.97%)	
Other Hispanic	75 (10.14%)	111 (15.02%)	66 (8.92%)	
Non‐Hispanic White	229 (30.95%)	204 (27.60%)	284 (38.38%)	
Non‐Hispanic Black	122 (16.49%)	146 (19.76%)	173 (23.38%)	
Non‐Hispanic Asian	159 (21.49%)	85 (11.50%)	43 (5.81%)	
Other race including multiracial	41 (5.54%)	33 (4.47%)	41 (5.54%)	
EDUCATION				< 0.001
Less than 9th grade	53 (7.16%)	53 (7.17%)	40 (5.41%)	
9–11th grade	60 (8.11%)	74 (10.01%)	86 (11.62%)	
High school graduate/GED or equivalent	109 (14.73%)	152 (20.57%)	168 (22.70%)	
Some college or AA degree	246 (33.24%)	270 (36.54%)	294 (39.73%)	
College graduate or above	271 (36.62%)	190 (25.71%)	152 (20.54%)	
Less than 9th grade	1 (0.14%)	0 (0.00%)	0 (0.00%)	
ALCOHOL.USE				0.371
Yes	22 (2.97%)	29 (3.92%)	32 (4.32%)	
No	718 (97.03%)	710 (96.08%)	708 (95.68%)	
SMOKING				< 0.001
Everyday	225 (30.41%)	318 (43.03%)	375 (50.68%)	
Some days	64 (8.65%)	48 (6.50%)	44 (5.95%)	
Not at all	451 (60.95%)	373 (50.47%)	321 (43.38%)	
HYPERTENSION				0.353
Yes	647 (87.43%)	645 (87.28%)	662 (89.46%)	
No	93 (12.57%)	94 (12.72%)	78 (10.54%)	
DIABETES				< 0.001
Yes	22 (2.97%)	40 (5.41%)	56 (7.57%)	
No	715 (96.62%)	693 (93.78%)	673 (90.95%)	
Borderline	3 (0.41%)	6 (0.81%)	11 (1.49%)	
MEDIUM.JOB.ACTIVITY				0.016
Yes	239 (32.30%)	257 (34.78%)	291 (39.32%)	
No	501 (67.70%)	482 (65.22%)	449 (60.68%)	

### 3.2. Correlation Analysis of SSAGP With BMI, Waist Circumference, Obesity, and Abdominal Obesity

In the unadjusted model, SSAGP was significantly positively correlated with BMI, waist circumference, obesity, and abdominal obesity (*p* < 0.0001). After adjusting for age and race/ethnicity (Adjustment I), the associations between SSAGP and BMI, waist circumference, obesity, and abdominal obesity remained significant (*p* < 0.0001). Further adjustment for education level, alcohol use, smoking status, insulin levels, HDL, TG, HbA1c, and hs‐CRP (Adjustment II) did not attenuate these associations (*p* < 0.0001). Specifically, a one‐unit increase in SSAGP level was associated with an increase of 4.42 in BMI (95% CI: 3.08, 5.76), an increase of 12.18 cm in waist circumference (95% CI: 9.22, 15.14), a 3.63‐fold increase in the risk of obesity (95% CI: 1.96, 6.72), and a 10.75‐fold increase in the risk of abdominal obesity (95% CI: 4.85, 23.85) (Table 2).

**TABLE 2:** Multiple regression analyses of SSAGP with BMI, waist circumference, obesity, and abdominal obesity.

	Nonadjusted	*p* value	Adjust I	*p* value	Adjust II	*p* value
Y = BMI						
SSAGP	16.58 (15.37, 17.79)	< 0.0001	15.16 (13.95, 16.38)	< 0.0001	4.42 (3.08, 5.76)	< 0.0001
SSAGP tertile						
Low	0		0		0	
Middle	4.34 (3.61, 5.07)	< 0.0001	3.62 (2.90, 4.34)	< 0.0001	1.27 (0.62, 1.91)	0.0001
High	9.23 (8.50, 9.96)	< 0.0001	8.29 (7.57, 9.02)	< 0.0001	1.94 (1.18, 2.70)	< 0.0001
Y = WAIST						
SSAGP	39.51 (36.80, 42.23)	< 0.0001	35.54 (32.84, 38.23)	< 0.0001	12.18 (9.22, 15.14)	< 0.0001
SSAGP tertile						
Low	0		0		0	
Middle	9.91 (8.27, 11.54)	< 0.0001	8.14 (6.54, 9.74)	< 0.0001	3.04 (1.62, 4.46)	< 0.0001
High	21.98 (20.35, 23.62)	< 0.0001	19.45 (17.83, 21.07)	< 0.0001	5.65 (3.96, 7.33)	< 0.0001
Y = Obesity						
SSAGP	77.71 (48.86, 123.59)	< 0.0001	63.94 (39.59, 103.27)	< 0.0001	3.63 (1.96, 6.72)	< 0.0001
SSAGP tertile						
Low	1.0		1.0		1.0	
Middle	3.39 (2.66, 4.31)	< 0.0001	2.95 (2.30, 3.79)	< 0.0001	1.66 (1.23, 2.24)	0.0010
High	9.85 (7.70, 12.58)	< 0.0001	8.54 (6.63, 10.99)	< 0.0001	1.78 (1.27, 2.48)	0.0008
Y = Abdominal obesity						
SSAGP	143.38 (76.74, 267.88)	< 0.0001	93.83 (49.17, 179.06)	< 0.0001	10.75 (4.85, 23.85)	< 0.0001
SSAGP tertile						
Low	1.0		1.0		1.0	
Middle	3.48 (2.72, 4.44)	< 0.0001	3.01 (2.32, 3.90)	< 0.0001	1.73 (1.28, 2.32)	0.0003
High	11.49 (8.12, 16.27)	< 0.0001	9.52 (6.65, 13.63)	< 0.0001	2.73 (1.77, 4.23)	< 0.0001

*Note:* Adjust I adjust for AGE, RACE. Adjust II adjust for PIR; EDUCATION; HDL; FBG; INSULIN; LDL; TG; TC; HBA1C; CRP; ALCOHOL.USE; SMOKING; HYPERTENSION; DIABETES; MEDIUM.JOB.ACTIVITY; AGE; RACE.

### 3.3. Threshold Effect Analysis of SSAGP With BMI and Waist Circumference

**FIGURE 2:**
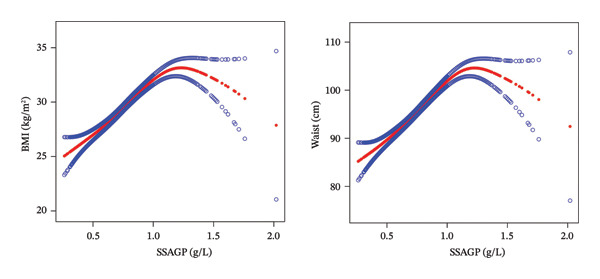
Fitted curves of SSAGP with BMI and waist circumference.

Threshold effect analysis revealed that the relationship between SSAGP and both BMI and waist circumference exhibited inflection points, with *K* values of 1.2 for both. Below the inflection point, a one‐unit increase in SSAGP level was associated with an increase of 9.94 (95% CI: 8.48, 11.41) in BMI and an increase of 23.98 cm (95% CI: 20.75, 27.20) in waist circumference. Above the inflection point, a one‐unit increase in SSAGP level corresponded to a reduction of 9.25 (95% CI: −15.78, −2.73) in BMI and a decrease of 21.57 cm (95% CI: −35.93, −7.21) in waist circumference. This indicates that SSAGP has a stronger promoting effect on BMI and waist circumference at lower levels, while an inhibitory effect is observed at higher levels. The results of the log‐likelihood ratio test were significant (*p* < 0.001) (Figure 2 and Table 3).

**TABLE 3:** Threshold effect analysis of SSAGP with BMI and waist circumference.

Outcome	BMI	*p* value	Waist	*p* value
Model I				
Linear effect	8.40 (7.04, 9.77)	< 0.0001	20.33 (17.33, 23.34)	< 0.0001
Model II				
Inflection point (K)	1.2		1.2	
Effect below K (Effect 1)	9.94 (8.48, 11.41)	< 0.0001	23.98 (20.75, 27.20)	< 0.0001
Effect above K (Effect 2)	−9.25 (−15.78, −2.73)	0.0055	−21.57 (−35.93, −7.21)	0.0033
Difference in effects (2‐1)	−19.19 (−26.13, −12.26)	< 0.0001	−45.55 (−60.82, −30.27)	< 0.0001
Predicted values at inflection points	37.36 (36.70, 38.03)		114.45 (112.96, 115.95)	
Log‐likelihood ratio test		< 0.001		< 0.001
Outcome	1.06, 1.23		1.06, 1.23	

*Note:* Adjusted variables: PIR; EDUCATION; FBG; HDL; INSULIN; LDL; TG; TC; HBA1C; CRP; ALCOHOL_USE; SMOKING; HYPERTENSION; DIABETES; MEDIUM_JOB_ACTIVITY; AGE; RACE.

### 3.4. Interaction Analysis

Interaction analysis revealed that education level significantly influenced the association between SSAGP and obesity (*p* = 0.0096), but not between SSAGP and abdominal obesity (*p* = 0.2644). In the subgroup with “college graduate or above” education, this association was strongest (OR = 319.06, 95%CI: 111.32, 914.51), substantially higher than that in lower education level groups such as “9x11th grade” (OR = 10.99, 95% CI: 3.22, 37.51) or “high school graduate/GED” (OR = 25.80, 95% CI: 9.77, 68.10). This indicated that education level positively moderated the effect of SSAGP on obesity risk, meaning that the positive association between elevated SSAGP levels and obesity risk was more pronounced among women with higher educational attainment. Additionally, age had a significant interaction with the association between SSAGP and abdominal obesity (*p* = 0.0095), but not with the association between SSAGP and obesity (*p* = 0.6313). Specifically, among women aged under 35 years, the association between SSAGP and abdominal obesity was stronger, suggesting that age may indirectly modulate the relationship between SSAGP and abdominal obesity through its impact on metabolic status and lifestyle. Other factors, including alcohol use, smoking, hypertension, diabetes, and moderate physical activity, did not show significant interactions with SSAGP (*p* > 0.05). The varying strengths of associations between SSAGP and obesity or abdominal obesity across different educational and age subgroups further indicate that education level and age may indirectly affect these relationships through their influence on lifestyle and metabolic status (Table 4).

**TABLE 4:** Interaction analysis of the correlation between SSAGP and obesity or abdominal obesity circumference due to related factors.

Subgroup	*N*	Obesity	*p*‐interaction	Abdominal obesity	*p*‐interaction
EDUCATION			0.0096		0.2644
Less than 9th grade	146	77.24 (11.05, 540.17)		88.58 (4.69, 1672.03)	
9–11th grade	220	10.99 (3.22, 37.51)		112.01 (15.08, 831.91)	
High school graduate/GED or equivalent	429	25.80 (9.77, 68.10)		73.42 (17.69, 304.70)	
Some college or AA degree	810	86.63 (40.09, 187.18)		352.25 (114.61, 1082.62)	
College graduate or above	613	319.06 (111.32, 914.51)		83.95 (28.21, 249.88)	
Less than 9th grade	1				
ALCOHOL.USE			0.9981		0.1893
Yes	83	84.22 (7.80, 909.52)		55.22 (3.86, 790.30)	
No	2136	77.35 (48.17, 124.19)		159.77 (83.67, 305.07)	
SMOKING			0.1047		0.2001
Everyday	918	37.05 (18.74, 73.23)		128.80 (46.58, 356.20)	
Some days	156	67.80 (12.23, 375.79)		1132.73 (81.82, 15,681.89)	
Not at all	1145	140.81 (70.02, 283.13)		111.99 (47.79, 262.45)	
HYPERTENSION			0.6296		0.3866
Yes	1954	90.52 (54.90, 149.24)		168.23 (85.73, 330.13)	
No	265	26.32 (7.59, 91.28)		48.02 (8.95, 257.80)	
DIABETES			0.7160		0.9995
Yes	118	81.34 (7.14, 926.57)		62.71 (0.06, 71,271.41)	
No	2081	76.00 (47.02, 122.86)		132.87 (70.64, 249.93)	
Borderline	20	0.96 (0.01, 96.35)		3356.39 (0.02, inf.)	
MEDIUM.JOB.ACTIVITY			0.2158		0.8925
Yes	787	95.40 (43.45, 209.48)		126.35 (45.80, 348.53)	
No	1432	67.81 (38.18, 120.42)		154.88 (70.02, 342.63)	
Age, years					
≤ 35	1267	82.16 (44.37, 152.12)	0.6313	171.95 (79.14, 373.64)	0.0095
> 35, ≤ 60	952	65.48 (32.14, 133.40)		68.86 (23.19, 204.53)	

## 4. Discussion

This study, based on data from NHANES 2015–2018, aimed to explore the relationship between SSAGP levels and obesity and abdominal obesity in women. Results showed that SSAGP levels were significantly positively correlated with BMI, waist circumference, obesity, and abdominal obesity. Specifically, for every one‐unit increase in SSAGP, there was a corresponding rise of 4.42 in BMI (95% CI: 3.08, 5.76), a corresponding rise of 12.18 cm in waist circumference (95% CI: 9.22, 15.14), a 3.63‐fold rise in the odds of obesity (95% CI: 1.96, 6.72), and a 10.75‐fold rise in the odds of abdominal obesity (95% CI: 4.85, 23.85). These associations were still significant in the model with multiple potential confounding factors adjusted.

Besides, threshold effect analysis showed a nonlinear relationship between SSAGP and both BMI and waist circumference with an inflection point of 1.2. When the SSAGP level was below the inflection point, SSAGP promoted BMI and waist circumference more effectively. While exceeding that point, its promoting effect weakened, and even transformed into an inhibitory effect. It implies that different levels of SSAGP may exert different impacts on obesity. These findings indicate that SSAGP is not only a potential biomarker for obesity but may also play an important role in the development and progression of obesity.

In recent years, the relevance of SSAGP to obesity has been of concern in an increasing number of studies. Most of these surmise AGP as an important biomarker for obesity and its metabolic diseases. Wu et al. [26] using data from the 2015–2018 National Health and Nutrition Examination Survey (NHANES) in the United States found that various fat‐related indicators were significantly positively correlated with AGP levels in a study of 2295 adult women. The indicators include the following: BMI (*β* = 23.65, 95% CI: 20.90–26.40) and TPF (*β* = 25.91, 95% CI: 23.02–28.80). Furthermore, the android/gynoid fat ratio and visceral/subcutaneous fat ratio also varied positively with AGP levels, demonstrating the intimate relationship of fat distribution with the levels of AGP. In studies conducted during adolescence, Ferrari et al. [27] measured 876 European adolescents and reported that overweight/obese adolescents had higher values of AGP when compared with lean/normal‐weight adolescents. The BMI z‐scores and fat mass were positively correlated with AGP, indicating that the association between obesity and AGP levels is also significant in adolescents. Paes‐Silva et al. [28] demonstrated that AGP levels are elevated in adolescents with abdominal obesity and that there are gender differences in the association between fat‐soluble vitamin levels and obesity.

Compared with these studies, our study also utilized data from NHANES 2015–2018 but focused on 2219 adult women to investigate the relationship between SSAGP and obesity and abdominal obesity. Our study further confirmed the association between AGP and obesity and quantified the strength of this relationship. Additionally, we found that education level significantly influenced the association between SSAGP and obesity, a finding not covered in some previous studies. This provides new insights into the role of socioeconomic factors in the relationship between obesity and AGP. However, our results differ from some studies. For example, certain studies have found that the association between SSAGP and obesity is more pronounced in men [17], while our study was limited to women. This may be due to differences in metabolic and inflammatory responses among genders.

In our study, the levels of SSAGP were significantly associated with obesity and abdominal obesity, and these might have a close link with its biological roles. As one acute‐phase reactant, SSAGP not only participates in several inflammatory processes but also is involved in regulating metabolism [29–31]. Inflammation represents one of the central pathophysiological mechanisms that drive the development of obesity and metabolic syndrome [32]. Most often, obesity is associated with a state of chronic low‐grade inflammation, and increased levels of SSAGP represent a response to such an inflammatory condition. Several studies have demonstrated that inflammatory cytokines, including tumor necrosis factor *α* (TNF‐*α*), IL‐1, IL‐8, IL‐11, and IL‐6, as well as other acute‐phase proteins (APPs) such as C‐reactive protein (CRP), haptoglobin (Hp), serum amyloid A (SAA), and hemopexin, can regulate AGP expression levels [33–35]. The study by Su et al. [33] further supports the direct involvement of SSAGP in inflammatory pathways, demonstrating that SSAGP can induce tissue factor (TF) expression in monocytes and promote the secretion of TNF‐*α*, thereby initiating inflammatory and coagulation responses. This may represent a key mechanism through which it participates in obesity‐related inflammation.

From a cellular and molecular perspective, Khanna et al. [36] reported that in the adipose tissue of obese individuals, macrophages undergo a phenotypic shift from M2 macrophages (anti‐inflammatory) to M1 macrophages (proinflammatory). Furthermore, adipose tissue secretes various adipokines and hormones—such as leptin and visfatin—which play critical roles in immune homeostasis and glucose metabolism. In addition, SSAGP may bind a wide range of drugs and lipids [37–39], which are involved in their metabolic pathways. In the obese state, SSAGP could further potentiate the metabolic disturbances of obesity through altered lipid metabolism and/or pharmacokinetics.

Multiple studies have indicated that obesity‐related inflammation involves multiorgan crosstalk. As the primary site of SSAGP synthesis, the liver is central to the pathophysiology of obesity and NAFLD. The spleen is directly connected to visceral adipose tissue and the liver through the portal circulation. They form a “spleen–liver axis” that critically regulates immune cell reprogramming and systemic inflammation [40]. Brummer et al. [41] demonstrated that obesity‐associated inflammation remodels the immune landscapes of the spleen, liver, and systemic circulation. The spleen–liver axis regulates obesity‐mediated systemic and hepatic immune dysregulation through the accumulation of myeloid‐derived suppressor cells (MDSCs) and natural killer T (NKT) cells in the spleen, underscoring the cell‐specific role of this axis in obesity‐induced immune dysregulation. By influencing monocyte and macrophage function, SSAGP may reshape the immune microenvironment of the spleen–liver axis and exacerbate obesity‐related metabolic dysfunction [36]. Therefore, SSAGP may serve not only as an inflammatory marker but also as a serological indicator of spleen–liver axis activation, providing a new perspective for evaluating metabolic disturbances associated with obesity. From the metabolic point of view, it is also interesting to note the relationship between SSAGP and insulin resistance. Insulin resistance is a common pathological feature in both obesity and type 2 diabetes [42, 43], and insulin resistance is associated with SSAGP levels [44]. This suggests that SSAGP may contribute to the development of obesity‐related metabolic disorders through its influence on the insulin signaling pathway. Moreover, the role of SSAGP in adipocytes should not be underestimated. It was reported that AGP inhibits the expression of lipogenesis genes, which in turn reduces glucose oxidation and its conversion to fatty acids [45]; this could be one of the direct mechanisms through which it affects obesity.

Furthermore, interaction analysis revealed that education level and age significantly moderated the relationship between SSAGP and obesity. Specifically, education level positively moderated the relationship between SSAGP and obesity. This may be attributed to the fact that individuals with higher education levels typically benefit from more systematic health monitoring and healthier lifestyle practices. Their SSAGP levels are less likely to be influenced by incidental inflammation and may better reflect chronic low‐grade inflammation associated with obesity. In contrast, individuals who have low educational attainments are more likely to lead highly unhealthy lifestyles characterized by high‐calorie intake, absence of physical activity, and smoking habits [46, 47]. Notably, education level significantly moderated the association between SSAGP and overall obesity but did not significantly affect the relationship with abdominal obesity. This suggests that socioeconomic and behavioral factors may primarily influence overall weight regulation through systemic energy balance, whereas fat distribution may be more strongly governed by intrinsic biological factors such as genetics and hormones.

On the other hand, age also moderated the relationship between SSAGP and abdominal obesity, with a particularly pronounced association observed in women under 35 years of age. This may reflect biological variations in fat distribution across different life stages: abdominal fat in younger women might be more sensitive to inflammatory signals or influenced by endocrine changes during reproductive periods. With advancing age, alterations in fat distribution and the increased prevalence of comorbidities may complicate the relationship between SSAGP and abdominal obesity [48]. In summary, the association between SSAGP and obesity is shaped by a combination of inflammatory, metabolic, lifestyle, and socioeconomic factors. The moderating role of sociodemographic factors should be emphasized in both obesity research and clinical practice.

Based on the NHANES database, our study has a large sample size and is nationally representative, allowing for a robust reflection of the relationship between SSAGP and obesity among adult women in the United States. Additionally, our study employed multiple statistical methods, including multiple regression analyses and threshold effect analysis, to comprehensively evaluate the association between SSAGP and obesity. We also considered the modulatory effects of socioeconomic factors such as education level, providing new insights into the complex etiology of obesity. However, our study’s cross‐sectional design precludes the determination of causality. Future research should involve prospective cohort studies or intervention trials to further validate the causal relationship between SSAGP and obesity. Moreover, due to the limited availability of male SSAGP samples in the NHANES database (restricted to males under 5 years old), our study was confined to females, precluding inference about the situation in males. Future studies should validate these findings in a broader population. Moreover, biomarkers that may be associated with affecting the level of SSAGP—for instance, levels of inflammation markers and hormone—were not measured in our study. It therefore may lead to incomplete interpretation for understanding the comprehensive association between SSAGP and obesity. Lastly, not considering all the possible confounders could imply unmeasured confounding, affecting the result.

Conclusively, a significant relationship of SSAGP was noted in females with obesity and abdominal obesity, reflecting the potential significance of the socioeconomic element. The results here are new ways being proposed for the early identification and intervention for obesity, whereas the relationship, causes, and mechanisms remain to be tested further in any other study.

## 5. Conclusion

This study, based on the NHANES data from 2015 to 2018, indicated that the level of SSAGP was significantly associated with obesity and abdominal obesity among women. SSAGP showed positive correlations with BMI, waist circumference, obesity, and abdominal obesity; these associations remained robust after adjustment for multiple confounding factors. Moreover, the threshold effect of SSAGP‐OB indicated that SSAGP had different effects on obesity at different levels. The association of SSAGP and obesity was moderated by education level; age interacted with the association between SSAGP and abdominal obesity, which implied that socioeconomic status and age may indirectly affect the risk of obesity through lifestyle and metabolic status.

While providing a new angle in the study of the function of SSAGP in the processes of obesity, the cross‐sectional nature of this investigation precludes it from attempting any assessment regarding its causal factor. Future analyses focusing on an examination of how it influences obesity as a disease mechanistically must validate the utility of SSAGP as an appropriate biomarker in humans.

## Author Contributions

Data curation: Ling Sun and Lingyan He. Funding acquisition: Changlin Zhai and Haihua Pan. Project administration: Changlin Zhai. Software: Ling Sun. Validation: Haihua Pan and Lingyan He. Visualization: Ling Sun. Writing–original draft: Ling Sun and Haihua Pan. Writing–review and editing: Changlin Zhai, Hao Zhang, and Bruno Fink.

All authors contributed to editorial changes in the manuscript. All authors have participated sufficiently in the work and agreed to be accountable for all aspects of the work.

## Funding

This research was funded by the Jiaxing Health Science and Technology Program (JWKD‐25002), the Zhejiang Province Traditional Chinese Medicine Scientific Research Fund (2023ZL700), and the Clinical Key Specialty Construction Project of Zhejiang Province‐‐Cardiovascular Medicine (2024‐ZJZK‐001).

## Disclosure

All authors read and approved the final manuscript.

## Ethics Statement

The studies involving human participants were reviewed and approved by the National Center for Health Statistics. The patients/participants provided written informed consent to participate in this study.

## Conflicts of Interest

The authors declare no conflicts of interest.

## Supporting Information

In addition to the main manuscript, we have included supporting informations to enhance the transparency and comprehensiveness of our study. Supporting raw data contain the complete set of raw data used in our analysis. This dataset includes all the original measurements and observations, providing essential support for the statistical analyses and conclusions presented in the main text.

## Supporting information


**Supporting Information** Additional supporting information can be found online in the Supporting Information section.

## Data Availability

The original contributions presented in the study are included in the article/supporting information, and further inquiries can be directed to the corresponding author.
