# Supplementary material for: The Association Between Serum α1‐Acid Glycoprotein and Obesity and Abdominal Obesity in Women: A Cross‐Sectional Study Based on NHANES Data From 2015 to 2018
Source: Int J Endocrinol. 2026 Jan 30;2026:1513929. doi: 10.1155/ije/1513929 (PMC12857777; doi:10.1155/ije/1513929)
Supplement: Supplementary file 1 — Supporting Information Additional supporting information can be found online in the Supporting Information section. [file IJE-2026-1513929-s001.pdf]

SEQN	PIR	EDUCATIO	HDL	FBG	INSULIN	LDL	TG	TC	HBA1C	CRP	ALCOHOL.	SMOKING	HYPERTEN	BPQ090D	DIABETES	DEPRESSI	MEDIUM.J	GENDER	AGE	RACE	HEIGHT	WEIGHT	BMI	WAIST	SSAGP
86384	0.5	1	1.06	12.6	153.18	3.698	2.506	5.9	6.8	2.9	2	1	1	1	1	0	1	2	37	3	160.2	88	34.28922	116.1	0.74
84285	0.54	4	1.06	5.65	45.06	4.215	1.05	5.77	6	9.4	2	1	1	1	1	0	1	2	44	7	162.6	86.2	32.60365	102.1	1.06
86087	2.36	4	2.43	7.7892	398.5376	2.82194	1.45692	5.09	7.1	12.4	2	3	1	1	1	0	1	2	31	1	154.1	100.6	42.36358	114.4	0.849
84904	2.55	5	1.27	8.3585	196.994	3.98926	1.618166	6.62	7.6	24.7	2	3	1	1	1	0	1	2	48	6	163.3	88.4	33.14972	97.4	1.07
86262	5	5	1.27	6.16	105.54	2.224	0.813	3.85	5	23	1	1	1	1	1	0	1	2	31	4	163.6	105.8	39.5293	128.3	0.692
91899	5	5	2.2	4.77	15.54	2.198	0.406	4.58	5.6	0.08	2	3	1	1	1	0	1	2	48	6	160.2	53.8	20.96318	82	0.373
85341	0.94	5	0.75	8.053600	180.9732	1.95868	1.6059	2.79	6	0.7	2	3	1	2	1	0	1	2	37	4	145.9	75.8	35.60891	105.4	0.631
84579	5	5	0.91	6.811733	261.3724	2.9397	2.33805	5.09	5.3	14	2	3	1	2	1	0	1	2	27	6	167.1	84.6	30.29824	96.6	0.969
91993	4.14	2	1.14	11.18109	205.9392	2.8824	1.66681	4.63	8.6	31.5	2	3	1	2	1	0	1	2	39	1	169.9	132.8	46.00567	147.2	0.65
86882	2.74	1	1.06	12.3008	234.3816	2.952545	1.60024	4.34	11.7	5.7	2	3	1	2	1	0	1	2	41	1	162.2	97.7	37.13581	112.2	0.769
88811	3.2586	4	1.53	12.3032	247.5609	2.922926	1.53696	4.5	10.2	6.1	2	3	1	1	1	0	1	2	19	6	156.9	45.1	18.32022	71.1	0.745
92277	0.21	3	2.33	5.61	11.94	2.146	1.095	4.97	5.2	0.3	1	1	1	1	2	0	1	2	45	3	169.3	71.1	24.80594	97	0.681
84927	2.47	5	1.03	5.83	122.88	2.198	1.14	3.75	5.7	2.3	2	3	1	1	2	0	1	2	43	3	174.5	130.9	42.98815	135.6	0.636
91215	1.13	2	1.32	6.27	158.52	3.284	1.31	5.2	6	6	2	1	1	1	2	0	1	2	43	1	159.3	94.2	37.12097	103.6	0.875
88211	0.56	3	1.01	6.8398	164.4656	2.75762	1.68576	4.47	6.6	52.3	2	1	2	1	2	0	1	2	44	4	168.1	137.1	48.5179	140.9	1.37
90260	5	5	1.53	5.9256	83.4528	3.69076	1.229733	5.64	5.4	2.6	2	3	2	1	2	0	1	2	45	4	173.7	97.3	32.24877	97.4	0.777
92198	2.4874	4	1.99	5.38	166.92	3.595	0.937	6.03	5.5	7.4	1	3	1	1	2	0	1	2	47	4	165.5	95.3	34.7934	106	0.835
91536	2.73	4	1.29	5.8732	83.0812	3.512186	1.31576	5.53	5.5	0.6	2	1	1	1	2	0	1	2	37	7	162	78.8	30.02591	94.8	0.621
91984	0.84	4	1.27	5.265911	65.9376	3.369514	0.89316	5.22	5.3	2.2	1	1	1	2	2	0	1	2	49	7	166.2	61.9	22.40932	84.5	0.827
86968	2.72	3	1.58	5.212433	54.3084	2.24323	0.71014	4.09	5.4	5.4	2	1	1	2	2	0	1	2	25	2	164.4	122.2	45.21344	95	1.23
87480	3.79	5	1.32	5.66	80.16	1.81	1.434	3.78	5.5	7.9	2	1	1	2	2	0	1	2	31	3	159.1	78.2	30.89345	98.9	0.951
90020	0.45	4	1.32	5.6866	104.3312	3.05928	1.17017	5.02	5.7	3.9	2	1	1	2	2	0	1	2	33	2	173.3	129.1	42.98621	133.7	1.25
91421	0.82	3	1.09	5.4798	106.7938	3.57386	1.61062	5.72	5.5	4.6	2	1	1	2	2	0	1	2	41	4	165.4	135.3	49.45689	153	0.999
90463	1.1	4	1.58	5.7512	85.6317	3.80038	1.18292	6.05	5.5	4.6	2	1	1	2	2	0	1	2	44	3	165.1	51.5	18.89353	73.6	0.922
84429	1.1896	2	1.24	5.3978	59.347	2.260178	0.87651	3.9	4.7	2.3	2	1	1	2	2	0	1	2	45	4	163.9	121.7	45.3036	140	0.866
92225	2.31	5	0.93	5.5621	136.8216	2.73278	1.31664	4.42	5.3	2.7	2	3	1	2	2	0	1	2	42	1	147.6	76.9	35.29829	108.2	0.912
92761	5	4	1.14	5.853965	126.8004	3.23984	1.65738	5.2	5.6	13.3	2	3	1	2	2	0	1	2	26	3	170.3	112.2	38.68687	116.2	1.12
87544	3.77	4	1.91	5.55	66.72	2.069	0.373	4.16	5.4	9.9	2	3	1	2	2	0	1	2	28	6	152.1	89.5	38.68696	113	0.688
84021	1.47	4	1.55	4.94	136.8	2.556756	0.78199	4.4	5	1.7	2	3	1	2	2	0	1	2	29	7	162.5	81.5	30.86391	105.6	0.748
88335	0.93	3	1.89	5.483777	82.32	3.738693	1.15147	6.05	5.7	2.4	2	1	1	2	2	0	1	2	33	6	159.5	58.5	22.99506	79	0.55
86297	1.7	4	1.14	5.3996	84.329632	2.714596	0.988925	4.5	5	0.9	2	1	1	2	2	0	1	2	35	3	158	81.4	32.60695	107.1	0.521
91922	1.16	4	1.11	5.482266	100.992	3.97828	1.71806	6.1	5.5	9	2	1	1	2	2	0	1	2	39	4	170.1	128.3	44.34228	135.4	0.848
89927	0.82	4	1.11	5.5	33.3	3.414	1.31	5.12	5.4	1.1	2	1	1	2	2	0	1	2	41	3	160.5	81.7	31.71553	120.6	0.735
93483	4.3	4	1.06	5.609466	67.6236	1.94674	0.87848	3.47	5.5	2.3	2	3	1	2	2	0	1	2	42	3	169	91.2	31.93166	110.8	0.995
91082	1.76	2	2.48	5.88	30.36	2.741	0.553	5.48	5.6	1	2	3	1	2	2	0	1	2	45	1	163.2	74.3	27.89642	89.3	0.651
87721	1.323027	4	0.93	5.66	140.7	3.284	0.926	4.63	5.8	10.5	2	1	1	2	2	0	1	2	45	4	158.7	95.7	37.99777	122.9	0.915
90295	3.52	5	1.06	5.7016	87.6192	2.306970	1.073255	4.01	5.8	0.3	2	3	1	2	2	0	1	2	48	4	167.2	81.9	29.2962	97.8	0.522
84948	4.83	5	1.63	5.2	74.52	2.146	0.982	4.22	5.7	17.2	2	3	1	2	2	0	1	2	41	4	162.5	103	39.00592	117	1.15
91111	2.769	1	2.3	9.27	1243.92	2.479436	1.16332	4.29	6	6.4	2	3	1	2	2	0	1	2	46	1	144.4	74.1	35.53725	99.2	0.793
90501	1.461770	4	1.24	5.2118	94.2372	2.38044	1.019753	4.06	4.9	3.3	2	1	2	2	2	0	1	2	21	3	154.6	58.4	24.43396	84.3	0.572
89651	1.32	4	1.47	5.064	66.396	2.710603	0.67387	4.58	4.958	1.1	2	3	2	2	2	0	1	2	26	2	145.4	45.8	21.6639	71.4	0.653
83857	5	5	1.58	5.8074	70.4682	3.83968	1.033276	6.47	4.8	0.9	2	3	2	2	2	0	1	2	31	3	159	70.4	27.847	85	0.623
84859	1.94	5	1.16	5.38	122.16	2.146	0.361	3.47	4.8	1	2	3	2	2	2	0	1	2	42	4	163.7	99.6	37.16738	124.7	0.795
85566	1.15	4	1.68	5.38	38.28	4.319	1.208	6.54	5.4	8.7	1	1	1	2	2	0	1	2	42	3	161.5	107.2	41.10075	116.1	1.14
86838	5	4	0.8	5.27	151.44	3.982	1.524	5.48	5.7	3.3	1	1	1	2	2	0	1	2	44	3	163.8	92.4	34.4385	107.9	1.12
83914	2.32	4	2.07	5.919248	101.286	4.3001	1.44514	7.5	5.6	6.7	1	3	1	2	2	0	1	2	23	2	171.5	99.3	33.76144	115.4	0.609
88790	3.72	5	1.97	5.5	20.04	2.198	0.339	4.32	5.6	0.2	1	3	1	2	2	0	1	2	31	6	156.9	48.6	19.74197	74	0.368
88674	0.08	2	1.45	5.66	144.78	3.64474	1.50894	5.53	5.8	3.9	1	1	1	2	2	0	1	2	27	4	162.8	117.5	44.3332	130.3	1.19
90516	3.7	3	2.74	5.256	53.1816	3.494961	1.16529	5.74	4.8	2.4	1	1	1	2	2	0	1	2	28	2	148	63.7	29.08145	89.7	0.386
83993	2.1	3	1.34	6.043348	96.7572	4.2623	1.46404	6.59	6.3	3.1	1	3	1	2	2	0	1	2	41	4	156.4	121.9	49.83451	141	0.966
92475	5	4	2.77	5.2539	42.4472	3.027586	0.70832	5.3	4.6	0.9	1	3	1	2	2	0	1	2	49	3	170.9	55	18.83122	73.4	0.933
84023	1.920433	3	1.81	5.064142	36.957	1.690716	0.450119	3.67	5.2	0.08	2	1	1	2	2	0	1	2	19	3	156.2	49.4	20.2472	71.7	0.611
90917	5	5	1.19	5.2914	80.17944	2.317133	1.08422	3.96	4.9	6	2	1	2	2	2	0	1	2	19	3	164	86.1	32.0122	92.9	0.686
86249	0.31	1	1.06	5.82355	80.4039	1.86666	0.771300	2.82	5.2	3.5	2	1	1	2	2	0	1	2	24	7	170.6	92.7	31.8509	122.5	0.855
84130	4.49	3	2.51	5	49.08	2.845	1.152	5.87	5.3	4.2	2	1	1	2	2	0	1	2	26	3	174.5	62.5	20.52528	78.3	0.507
89805	2.89	5	2.48	5.200542	40.4184	1.82942	0.50624	3.96	5	0.08	2	1	1	2	2	0	1	2	26	3	161	57.5	22.18279	78.7	0.703
86805	0.92	3	1.03	5.611066	88.490932	2.149258	1.1829	3.75	5.6	2.4	2	1	1	2	2	0	1	2	31	2	156.9	80.8	32.82203	98.1	1.01
90670	0.42	4	1.37	5.55	34.38	1.836	1.231	3.78	5.3	0.5	2	1	1	2	2	0	1	2	34	3	155.5	61.1	25.26856	78.8	0.689
91916	5	4	1.68	5.26348	50.4752	2.388335	0.56709	4.34	5.5	1.1	2	1	1	2	2	0	1	2	35	3	157.1	50.8	20.58313	78.1	0.874

84879	0.82	2	1.34	5.685452(	108.1005	3.096895	1.19536	5.07	5.9	2.9	2	1	1	2	2	0	1	2	35	1	164.1	107.9	40.06861	119.5	0.906
84956	5	4	2.72	5.3717	44.6268	2.981353	0.9832	5.46	4.8	0.08	2	1	1	2	2	0	1	2	43	3	163	58.3	21.94287	87.5	1.07
91358	1.54	4	0.91	5.7822	167.1156	2.01016	1.12442	2.61	5.1	12.1	2	1	1	2	2	0	1	2	43	7	169.3	60.5	21.10773	79.8	1.1
87007	3.09	4	0.85	11.5	112.32	4.81	2.789	6.93	7.7	4.8	2	1	1	2	2	0	1	2	45	3	164.4	106.5	39.40451	122.2	1.09
86447	0.75	3	1.47	5.11	40.98	1.94	0.756	3.75	4.7	0.4	2	2	2	2	2	0	1	2	22	3	157.4	64.1	25.87311	82.5	0.629
92675	2.75	4	1.89	5.16	32.76	2.25	0.418	4.32	5.3	1.6	2	2	1	2	2	0	1	2	30	1	156.6	56.1	22.87596	83	0.561
86720	5	5	1.99	6.33	21.54	3.1202	0.653207	5.43	5.5	0.3	2	2	1	2	2	0	1	2	34	1	156.2	50.3	20.61607	76.2	0.437
92642	1.09	4	1.37	5.255	62.112	2.851475	0.82012	4.68	5.3	2.1	2	2	1	2	2	0	1	2	34	3	163.9	86.9	32.34908	104.4	0.817
88116	5	5	1.66	5.22	21.18	3.31	0.553	5.22	4.9	0.4	2	2	1	2	2	0	1	2	41	3	165.9	67.2	24.41611	89.3	0.545
88786	1.19	3	0.8	11.5	318.78	2.556814	2.47445	3.59	8.4	16	2	2	1	2	2	0	1	2	43	4	175.2	131.1	42.71048	139.5	0.812
89434	2.02	5	2.4	4.94	20.28	2.96703	0.515575	5.33	5.2	0.08	2	2	1	2	2	0	1	2	46	6	159.2	60.2	23.75256	77.2	0.39
88694	1.64	3	1.32	5.27	65.94	1.914	1.389	3.88	5.2	1.8	2	3	1	2	2	0	1	2	19	7	148.1	66.2	30.18199	90.1	0.48
87821	0.82	4	1.34	4.77	74.64	2.172	0.723	3.85	5.2	1.8	2	3	1	2	2	0	1	2	20	3	165.8	91.2	33.17613	108	0.87
92358	4.21	4	0.98	4.44	19.38	2.948	1.163	4.47	5.1	2.6	2	3	1	2	2	0	1	2	21	3	164	58.7	21.82481	81.4	0.408
90614	1.74	2	1.86	5.406	115.8624	3.359833	1.23482	5.38	4.6	29.2	2	3	1	2	2	0	1	2	23	3	151.8	69.3	30.0739	97.7	0.651
85131	4.21	4	2.22	5.2558	61.8864	2.645733	0.734046	4.99	4.9	2.5	2	3	1	2	2	0	1	2	24	3	157.9	54.5	21.8591	68.4	0.464
86218	1.74	3	1.5	5.193333	63.4236	1.895	0.58319	3.52	5	2.1	2	3	2	2	2	0	1	2	25	3	161	58.4	22.52999	89.8	0.521
86905	4.39	4	1.09	4.94	31.92	2.198	0.745	3.62	5.1	10.9	2	3	1	2	2	0	1	2	26	3	173.3	108.2	36.02717	110.2	1.01
90502	1.910117	4	2.12	5.0436	44.6464	2.410551	0.62816	4.76	4.9	1	2	3	1	2	2	0	1	2	27	3	166.7	78.7	28.32067	91.1	0.566
85408	2.5	4	1.53	5.1574	66.438	1.924423	0.567055	3.34	4.8	1.2	2	3	2	2	2	0	1	2	30	7	165.5	90.2	32.93143	105.8	0.365
89304	4.63	5	1.97	4.61	24.66	2.508	0.711	4.81	5.2	0.6	2	3	1	2	2	0	1	2	30	3	176.9	82.4	26.33125	94.4	0.668
83752	5	4	1.73	5.0604	58.6398	2.654669	0.93569	4.76	4.8	0.9	2	3	1	2	2	0	1	2	30	2	163.6	71.2	26.60195	90.7	0.767
87005	1.35	4	0.83	6.137111	179.0478	2.862386	1.3398	4.47	5.8	4.9	2	3	1	2	2	0	1	2	31	1	158.3	75.3	30.04922	92.4	0.968
84305	0.36	3	1.45	5.196	54.627	3.19674	1.30862	5.48	5	1.7	2	3	1	2	2	0	1	2	36	1	153.2	75.5	32.1684	110.7	0.986
91515	2.24	4	1.89	5.012564	39.27	2.0145	0.47994	3.96	4.3	0.5	2	3	1	2	2	0	1	2	38	3	172.8	66.4	22.23723	81.6	0.516
91983	3.52	4	0.91	5.7286	141.7548	2.73136	1.45128	4.47	5.3	8.1	2	3	1	2	2	0	1	2	38	3	163.6	89.1	33.28979	101	1.22
91334	5	5	1.58	5.16	40.38	3.051	2.111	5.59	5.3	0.7	2	3	1	2	2	0	1	2	39	6	163.4	52.6	19.7007	77	0.565
84033	3.11	4	1.16	5.22	49.2	2.146	0.914	3.72	5.5	4	2	3	1	2	2	0	1	2	41	7	163.4	89.7	33.59606	96	1.02
90610	3.72	5	1.53	5.367813	50.4384	2.315133	0.60692	4.01	5.5	1.4	2	3	1	2	2	0	1	2	43	4	161.8	72.5	27.6937	90.9	0.558
85354	3.71	3	1.68	5.66	93.9	2.974	1.863	5.51	6	0.2	2	3	1	2	2	0	1	2	45	4	166.4	111.2	40.16041	127.8	0.566
85302	2.89	3	1.37	5.11	29.64	3.129	0.621	4.78	5.5	0.4	2	3	1	2	2	0	1	2	46	3	157.8	72.1	28.95485	96	0.694
88160	0.17	3	0.88	5.5126	89.134133	1.93538	1.29376	3.13	5.1	2	2	1	2	2	2	0	1	2	19	2	156.1	59.6	24.4591	84.8	0.784
91791	5	4	1.86	5.37	46.32	2.67101	0.7076	4.84	5	0.7	2	3	1	2	2	0	1	2	20	7	154.5	45.2	18.9357	87.3	0.372
92478	3.42	4	0.88	6.33	245.58	3.439	2.506	5.46	5.8	15.8	2	1	1	2	2	0	1	2	20	2	165.5	132.4	48.33837	128.8	0.915
88607	1.65	4	1.99	5.681048	95.3208	4.20442	1.472985	6.9	4.9	10	2	3	1	2	2	0	1	2	21	3	148.5	74.3	33.69271	102.6	0.483
85515	2.1	4	1.45	4.61	72.36	3.62	0.508	5.3	5.4	2.5	2	3	1	2	2	0	1	2	21	4	167.9	122.7	43.52544	133.2	0.928
83969	2.01	4	1.22	5.36028	93.2868	2.355786	1.179763	4.19	5	8.5	2	1	2	2	2	0	1	2	21	3	164.9	95.8	35.23094	107.2	1.08
91305	0.66	4	1.86	5.05	31.74	1.991	0.756	4.19	4.9	0.3	2	3	1	2	2	0	1	2	22	4	150.7	46	20.25496	70.4	0.373
90361	0.75	4	2.3	5.136942	40.914	2.065776	0.557266	4.27	5	0.08	2	3	1	2	2	0	1	2	22	3	157.9	62.2	24.94745	85.4	0.543
86321	1.07	4	1.66	5.08356	39.8568	2.07645	0.67414	4.09	5.1	0.7	2	3	1	2	2	0	1	2	25	6	150.2	49.3	21.8528	77.5	0.49
86961	3.65	5	1.47	4.94	57.6	2.767	1.558	4.97	4.8	2.8	2	3	1	2	2	0	1	2	25	3	154.4	56.9	23.86809	77	0.746
92551	5	5	1.34	5.4552	83.98584	2.069878	0.816951	3.47	5.4	4.5	2	3	2	2	2	0	1	2	25	4	167.5	85.3	30.40321	95.8	0.961
92129	3.4	4	1.89	5.371533	106.3692	2.89528	1.136073	5.17	5.4	13.6	2	3	1	2	2	0	1	2	26	3	161.8	82.9	31.66631	102.8	0.622
88065	3.72	5	1.68	5.77	29.34	2.172	0.587	4.11	5.1	0.6	2	3	1	2	2	0	1	2	26	2	154.6	48.4	20.25006	70.4	0.73
93392	0.38	4	2.09	5.7175	111.9252	2.969405	1.23332	5.25	5.6	5.8	2	1	1	2	2	0	1	2	26	3	171.2	126.8	43.26251	139.5	0.977
93524	2.99	4	1.34	5.8828	98.65679	2.341893	0.94112	3.93	5.4	37.7	2	3	1	2	2	0	1	2	26	1	161.7	115.8	44.28825	128.1	1.2
92190	0.92	4	1.5	5.10808	46.7388	3.386423	1.12404	5.92	5	0.08	2	3	1	2	2	0	1	2	27	7	150.2	56.7	25.13293	83.4	0.615
85878	5	5	1.78	5.28535	68.97	2.21462	0.69534	3.98	4.9	2.4	2	3	2	2	2	0	1	2	27	3	161.2	83.4	32.0949	102.2	0.734
83781	3.357325	5	1.4	5.38	83.82	2.069	0.666	3.78	5.6	7.1	2	3	1	2	2	0	1	2	27	4	160.7	87.8	33.99874	103.6	0.751
90804	1.77	4	1.45	5.5369	75.2196	3.66284	1.09995	5.74	5.6	2.8	2	3	1	2	2	0	1	2	27	4	167.9	120.1	42.60314	131.8	0.804
88835	5	5	1.99	5.145060	20.85836	2.434165	0.486053	4.4	5.2	0.3	2	2	1	2	2	0	1	2	28	2	160.3	60.3	23.46661	87.2	0.551
93610	2.333939	4	1.76	5.221013	45.31652	2.1625	0.6268	4.01	5.5	3.3	2	3	1	2	2	0	1	2	28	4	170.6	116.5	40.02837	118.6	0.562
91366	2.23	4	1.22	5.16	63.12	2.896	0.565	4.37	5.2	1.2	2	1	1	2	2	0	1	2	28	1	155.3	75.3	31.22138	98.9	0.624
88666	2.88	4	1.71	5.33	66.06	1.965	0.61	3.96	4.8	0.4	2	3	2	2	2	0	1	2	28	3	166.8	78.9	28.35861	92.3	0.66
84924	3.73	5	1.81	5.06855	46.9428	2.626333	1.1373	4.89	4.4	4.6	2	3	1	2	2	0	1	2	28	3	163.5	77.9	29.14083	110.2	0.675
88720	1.49	3	1.45	5.05	64.8	1.112	0.361	2.72	5.6	0.2	2	3	1	2	2	0	1	2	28	4	165.3	61.6	22.54421	80.5	0.72
91249	5	5	2.22	5.5	43.62	2.394382	0.675386	4.58	4.3	0.08	2	3	1	2	2	0	1	2	29	3	165.4	60.4	22.07832	79.5	0.606
87482	1.84	4	1.16	5.4546	103.56	2.209	1.066033	3.85	5.4	11.3	2	3	1	2	2	0	1	2	29	1	153.9	79.9	33.73413	104	1.06
87634	5	5	1.5	5.167657	70.1602	2.861369	1.03176	4.84	4.8	0.8	2	3	2	2	2	0	1	2	30	3	162.7	70.1	26.48152	82.9	0.723
93120	2.97	5	0.85	6.106948	170.7702	4.39252	2.465186	8.66	5.8	13.3	2	2	1	2	2	0	1	2	30	3	175.6	109.8	35.60847	114.6	0.904

84171	5	4	0.96	5.509027	78.06773	3.041653	1.56554	4.99	5.3	1.5	2	1	1	2	2	0	1	2	30	3	156.5	81	33.07169	105	1
85132	5	5	1.84	5.202592	27.54488	1.842347	0.489044	3.65	5.1	0.3	2	3	1	2	2	0	1	2	31	2	169.2	72.6	25.35922	83.2	0.592
85407	3.24	5	2.12	5.16	19.86	2.302	0.395	4.6	5.4	4	2	2	1	2	2	0	1	2	31	6	161.6	50.7	19.41446	76.3	0.693
87942	0.92	3	1.47	5.5	83.28	1.526	0.305	3.13	5.9	9.5	2	3	2	2	2	0	1	2	32	4	157	131.4	53.30845	136.4	0.863
91999	5	4	2.4	5.11	24.84	4.344	0.531	6.98	5.2	0.5	2	2	1	2	2	0	1	2	33	3	160.8	66.3	25.64138	90.9	0.673
92510	1.23	4	0.91	5.519466	120.4605	3.94878	1.91068	6	5.5	8.2	2	1	1	2	2	0	1	2	33	3	164.2	107.4	39.83437	127.7	1.07
89750	0.9	3	1.03	5.568666	82.64213	2.294838	0.87364	3.98	5.6	1.8	2	1	1	2	2	0	1	2	33	1	160.2	72.8	28.36654	90.1	1.31
92506	0.98	2	1.11	6	46.32	2.896	0.914	4.42	5	1.4	2	1	1	2	2	0	1	2	34	1	155.8	68.3	28.13752	97.4	0.775
93267	4.22	5	2.2	5.394581	43.5735	2.398002	0.58416	4.65	5.3	0.3	2	3	1	2	2	0	1	2	35	3	174.2	64.6	21.28807	83	0.45
87808	3.82	5	1.68	5.2836	43.90043	3.255842	1.00114	5.53	4.7	1	2	3	1	2	2	0	1	2	35	3	171	71.9	24.58876	85.4	0.636
86795	0.05	4	1.09	5.27	126.54	4.06	1.106	5.66	5.6	7.8	2	1	1	2	2	0	1	2	35	7	151.5	82.6	35.98776	106.4	0.764
87162	1.94	4	1.24	5.61	152.7	3.077	1.761	5.12	5.4	4.4	2	3	1	2	2	0	1	2	35	1	155.3	88.5	36.69445	113.2	0.873
84419	2.06	5	1.32	5.6304	119.5476	2.63516	1.04934	4.34	5.5	23.6	2	3	1	2	2	0	1	2	35	1	157.8	114	45.78159	133.7	1.32
92051	2.88	3	1.42	5.2858	78.1344	2.226521	0.933911	4.09	5	5.7	2	1	2	2	2	0	1	2	36	1	153.9	68.9	29.08988	91.1	0.327
90287	0.77	4	1.6	5	43.5	2.224	0.756	4.16	5.5	3.2	2	3	1	2	2	0	1	2	36	4	170	71.3	24.67128	94.2	0.634
86510	1.1	4	1.73	5.37	177.18	2.586	1.118	4.84	5.6	4.5	2	3	1	2	2	0	1	2	36	2	172.8	126.9	42.49855	132.6	1.06
87994	4.58	5	1.6	5.397066	49.6996	2.66178	0.6345	4.5	5.6	0.9	2	3	1	2	2	0	1	2	37	4	166.7	77.6	27.92483	96.5	0.769
86528	1.76	4	1.01	5	18.66	1.94	0.655	3.26	5.6	1.4	2	3	1	2	2	0	1	2	37	4	163.1	63.5	23.87074	82.1	0.806
88605	2.55	3	1.42	5.5374	77.1976	3.93542	1.70705	6.31	5.4	5.7	2	3	2	2	2	0	1	2	37	3	161.2	62.8	24.16738	84.8	1.09
86177	2.56	5	1.76	5.109728	46.8624	2.35775	0.60914	4.45	5.1	0.6	2	3	1	2	2	0	1	2	38	3	169	65.7	23.0034	82.8	0.453
87969	5	5	1.4	5.7288	113.1564	4.23756	1.533726	6.47	5.3	11.2	2	3	1	2	2	0	1	2	38	1	152.5	81.4	35.00134	106.5	1.02
91570	4.22	5	1.14	5.4364	60.5148	2.1201	0.96072	3.8	5.2	0.5	2	3	1	2	2	0	1	2	39	6	157.6	56.6	22.78788	82.2	0.476
87520	3.7	5	1.4	5.33	30.48	1.862	0.723	3.59	5.4	0.5	2	3	1	2	2	0	1	2	39	2	172.2	63.1	21.27958	84.2	0.704
92998	5	4	1.4	4.74	101.64	2.405	0.813	4.16	5	5.5	2	3	1	2	2	0	1	2	39	2	162.8	89.9	33.91961	100	0.914
92412	5	5	1.4	5.162657	45.952	3.161513	1.19219	5.22	5	1.7	2	3	1	2	2	0	1	2	40	3	164.3	72.2	26.7462	101.6	0.782
84308	5	5	2.15	5.2334	62.17113	2.187316	0.53406	4.19	5.7	0.9	2	3	1	2	2	0	1	2	41	3	177.4	64.9	20.62232	80.3	0.516
93030	5	5	2.38	5.399	87.33218	2.93905	0.974393	5.28	5.7	0.5	2	3	1	2	2	0	1	2	42	1	166.7	68.2	24.54218	88.3	0.393
88632	1.49	5	1.58	5.544133	94.5192	2.871535	1.09382	4.94	5.5	9.9	2	3	1	2	2	0	1	2	42	4	165.4	71.6	26.17231	92.9	0.766
85319	1.49	3	1.09	5.592466	105.2556	2.58042	1.169576	4.45	5.8	0.6	2	1	1	2	2	0	1	2	42	2	150.9	75	32.9369	94	0.797
86590	4.08	5	0.96	5.44	212.04	2.638	3.771	5.33	5.9	10.3	2	2	1	2	2	0	1	2	42	7	172	109.3	36.94565	115.2	0.9
86681	1.47	5	2.15	5.3262	41.949	2.680122	0.69292	4.84	5.6	0.08	2	3	1	2	2	0	1	2	44	2	156.4	48.6	19.86839	73.1	0.693
90932	5	4	2.72	5.33	42.84	2.689	0.44	5.61	5.2	1.7	2	3	1	2	2	0	1	2	45	3	156.9	64.6	26.24138	88.2	0.452
86941	3.81986	4	2.56	5.2231	37.36114	2.94873	0.62544	5.3	5	0.3	2	3	1	2	2	0	1	2	45	2	159.5	61	23.97775	81	0.522
92419	1.65	4	1.16	6.22	144.54	2.431	1.005	4.06	5.6	8.9	2	3	1	2	2	0	1	2	45	3	167	116.1	41.62932	117.9	1.27
85942	1.76	5	2.15	5.215477	45.5124	2.688685	0.67594	4.99	5.3	0.9	2	3	1	2	2	0	1	2	46	3	160.3	65.3	25.41243	92.9	0.627
93279	1.49	4	1.22	5.44	79.38	1.836	0.587	3.31	5.6	1.4	2	1	1	2	2	0	1	2	46	4	170.9	87.1	29.82181	107.1	0.775
85200	4.44	4	1.55	5.36488	61.39628	2.34762	0.64316	4.14	5.6	6.7	2	3	1	2	2	0	1	2	46	1	159.2	130.6	51.52963	142.9	0.989
93116	2.38	4	1.3676	5.828145	104.7919	2.712065	1.21184	4.628236	5.6	10.57873	2	3	1	2	2	0	1	2	47	4	164.3	53.9	19.96704	75.2	0.422
93488	5	4	1.45	5.82	30.6	1.345	0.621	3.08	5.8	4	2	3	1	2	2	0	1	2	47	4	158	57.9	23.1934	79.4	0.701
89457	5	5	1.6	5.227821	42.94931	2.903360	0.731996	4.91	5.1	0.4	2	3	1	2	2	0	1	2	48	3	173	67.1	22.41973	83.2	0.645
90293	2.47	3	1.42	6.418711	161.179	3.29146	1.501465	5.4	7	4.6	2	3	1	2	2	0	1	2	48	4	166.2	82.8	29.97563	91.7	0.74
90808	2.08	4	1.91	4.77	13.98	3.31	0.565	5.48	5.3	0.6	2	2	1	2	2	0	1	2	48	3	155	54.3	22.60146	78	0.748
85580	0.95	4	1.6	4.94	29.76	3.388	0.734	5.33	5.6	61.3	2	3	1	2	2	0	1	2	48	4	170.5	68.9	23.70121	86.9	1.2
90168	5	5	1.63	4.61	18	2.793	0.677	4.73	5	2.5	2	3	1	2	2	0	1	2	49	3	175.3	68.8	22.38848	82.3	0.535
91750	4.93	5	2.12	5.77	38.04	2.793	0.79	5.28	5.4	0.08	2	3	1	2	2	0	1	2	49	3	166.2	60.6	21.93868	79.5	0.569
90593	1.94	4	1.66	5.94	122.5414	3.27648	1.12016	5.38	6.1	18.5	2	1	1	2	2	0	1	2	49	4	162.3	114.1	43.31603	121.2	0.875
88530	3.89	3	1.42	5.16	41.16	3.465	1.231	5.46	5.4	6.3	2	3	1	2	2	0	1	2	49	3	171.6	81.1	27.54142	97.1	0.97
90609	5	4	1.16	5.99492	151.5864	2.86278	1.47824	4.81	5.5	22.3	2	3	1	2	2	0	1	2	49	3	169.9	82.6	28.61497	104	1.21
89702	0.6	4	2.35	4.68	43.44	2.379	0.384	4.91	4.6	0.08	2	3	1	2	2	0	1	2	18	4	167.4	64	22.83858	85.2	0.525
89654	1.77	4	2.02	4.77	43.92	2.198	0.553	4.47	5.7	0.3	2	3	1	2	2	0	1	2	18	4	157.6	56.8	22.86841	72	0.69
83809	3.3	3	2.04	5.22	43.14	1.577	0.169	3.7	5.7	0.08	2	1	1	2	2	0	1	2	20	4	166.8	72.9	26.20206	92.6	0.414
93178	3.09	4	1.11	8.38	774.42	2.28296	1.30848	3.75	5.5	4.6	2	3	1	2	2	0	1	2	20	7	170.4	112.3	38.67591	115.8	1.13
88970	5	5	1.19	5.4318	92.4484	2.759326	1.094052	4.53	5.1	2	2	1	1	2	2	0	1	2	23	6	148.7	48.1	21.7532	71	0.388
90837	0.01	2	0.88	5.5994	108.0876	2.187191	1.4423	3.75	5.4	0.9	2	1	1	2	2	0	1	2	23	4	166.4	77.9	28.13396	88.8	0.866
86670	0.94	5	1.91	5.194866	43.2732	2.301032	0.786	4.53	5.2	6.4	2	3	1	2	2	0	1	2	25	1	155.2	61.1	25.36634	85.5	0.702
84192	1.58	4	1.42	5.2876	72.103	2.58157	0.8192	4.42	5.1	3.4	2	1	1	2	2	0	1	2	27	2	149.4	85.1	38.12663	112	0.758
90926	5	5	1.45	5.9884	160.134	3.10138	1.4462	5.09	5.9	15.3	2	3	1	2	2	0	1	2	28	6	150.9	82.7	36.31843	109.9	0.622
91887	0.67	3	1.16	5.299714	78.3648	2.2186	0.924753	4.03	5.2	2.8	2	1	1	2	2	0	1	2	28	1	167.1	135.8	48.63477	137.9	0.674
88833	2.89	5	1.86	5.3756	103.6758	2.54822	0.967573	4.4	5.6	20.6	2	3	1	2	2	0	1	2	28	3	167.9	114.8	40.72307	117.9	0.894
88072	3.14	4	1.99	5.171470	47.1864	2.260866	0.456582	4.45	5.3	0.7	2	3	1	2	2	0	1	2	31	3	156.1	53.3	21.87366	78.2	0.702

89230	2.22	4	1.24	4.94	98.88	2.017	2.811	4.55	4.8	5.7	2	3	1	2	2	0	1	2	31	3	163.4	94.9	35.54366	119.4	0.717
87046	0.74	5	1.03	5.5572	83.1472	2.1506	0.924800	3.7	5.5	0.9	2	1	1	2	2	0	1	2	32	3	165.4	66.7	24.38119	81	0.722
85396	3.547017	5	1.5	5.33	54.78	2.638	0.768	4.5	5.4	1.2	2	3	1	2	2	0	1	2	33	6	160.6	71	27.52753	93.7	0.747
87080	0.94	1	1.19	5.72	117.66	3	1.298	4.78	5.8	1.5	2	1	1	2	2	0	1	2	33	2	145.8	72.2	33.96426	102.2	0.856
92428	5	5	1.09	5.5852	110.6112	3.63356	1.8004	5.82	5.2	4.4	2	3	1	2	2	0	1	2	33	6	153.9	63.2	26.68332	87.5	0.932
88412	2.19	4	1.63	4.72	46.5	2.664	0.463	4.5	5	1.6	2	3	1	2	2	0	1	2	34	4	154.9	52	21.67208	75.4	0.72
92802	5	4	1.5	5.747411	87.3096	3.15336	1.19512	5.2	5.6	5.6	2	3	1	2	2	0	1	2	38	3	169.7	131.3	45.5933	128	1.18
86322	1.37	4	1.16	5.7904	122.2128	4.03048	1.44958	6.13	6.1	8.2	2	1	1	2	2	0	1	2	39	2	161.4	92.1	35.35514	103.1	0.842
88293	2.69	5	1.6	5.11	37.02	2.302	0.937	4.34	5.3	2	2	3	1	2	2	0	1	2	41	4	165.1	69.3	25.42372	91	0.803
87636	1.23	4	1.58	5.996	109.3146	2.574445	0.9926	4.24	6	21.3	2	1	1	2	2	0	1	2	42	4	168.3	123.8	43.70714	110.1	1.14
84837	1.65	2	1.37	5.716052	120.8292	3.175055	1.16707	5.09	5.9	4.7	2	1	1	2	2	0	1	2	45	1	155.7	72.3	29.82367	92.4	0.603
90186	2.53	3	1.66	5.61	72.48	1.81	1.456	4.14	5.1	3.3	2	3	1	2	2	0	1	2	48	4	165.9	89	32.33681	99.9	0.582
91078	1.41	2	1.78	6.11	31.68	2.224	1.66	4.76	4.7	0.5	1	1	1	2	2	0	1	2	36	3	163.6	47.4	17.70972	75.1	1.05
85779	1.993	3	1.11	6.22	115.02	1.991	1.513	3.8	5.9	6.3	2	3	1	2	2	0	1	2	35	1	168.4	100.5	35.43903	115.4	0.952
90189	1.59	3	1.37	5.11	22.56	2.431	0.44	4.01	4.6	0.4	2	2	1	2	2	0	1	2	23	1	159.9	53.9	21.08103	78	0.604
91893	1.17	4	1.06	5.55	57	3.879	0.723	5.28	5.2	3.2	2	1	1	2	2	0	1	2	33	7	155.7	82.9	34.19616	100.4	0.842
84825	1.41	4	1.16	5.824333	133.6788	2.417225	1.17452	4.09	5.7	10.9	2	3	1	2	2	0	1	2	41	4	159.8	144.1	56.43005	141	1.2
86050	1.07	5	0.93	5.381866	122.6472	3.65847	1.791	5.69	5.5	6.8	2	1	1	2	2	0	1	2	39	4	151.7	80.4	34.93694	110.2	0.731
91821	2.06	3	1.97	5.192313	41.23967	2.103186	0.54658	4.19	5.5	1.2	2	3	1	2	2	0	1	2	47	6	152.1	52	22.47734	78	0.607
91323	2	4	1.19	4.77	104.58	1.991	0.723	3.52	4.8	1.2	1	1	2	2	2	0	1	2	26	4	172	93.3	31.53732	106.9	0.781
91794	1.07	3	1.91	4.88	36	1.681	0.327	3.75	5.3	0.5	1	1	1	2	2	0	1	2	25	1	165.9	59.5	21.61843	78.3	0.63
89160	1.36	3	1.29	6.1	35.58	4.629	1.174	6.47	5.2	1.5	1	1	1	2	2	0	1	2	35	3	172.8	78.3	26.22251	100.2	0.803
85399	0.92	4	1.29	4.11	66.66	3.362	0.779	5.02	5.5	3.9	1	1	1	2	2	0	1	2	35	3	165.3	104.4	38.20804	128.2	1.05
85787	4.46	4	1.16	5.2608	80.2896	1.931853	0.98192	3.13	4.9	0.08	1	3	2	2	2	0	1	2	26	3	178.1	62.3	19.64085	81.7	0.623
91714	0.97	4	2.22	4.27	36.12	4.474	2.032	7.63	5.5	4.4	1	3	1	2	2	0	1	2	35	7	162.8	72.1	27.20361	108.6	0.587
89825	0.06	3	1.68	5.423	48.8952	1.954853	0.55052	3.7	5.8	0.08	1	1	1	2	2	0	1	2	23	4	161.6	52.9	20.2569	68.5	0.481
84440	1.7	2	2.15	5.5044	48.8226	2.824375	0.66362	4.99	4.9	0.4	1	1	1	2	2	0	1	2	30	7	168.8	66.8	23.44399	82.5	0.784
84248	0.35	4	1.81	5.169546	35.904	1.854742	0.5882	3.9	5.5	0.5	2	1	1	2	2	0	1	2	29	4	163.5	71.4	26.70931	97.5	1.18
89839	2.46	4	1.24	5.4038	80.42864	3.03616	1.202806	4.99	5.2	0.8	2	1	1	2	2	0	1	2	36	3	168.5	85.5	30.11385	96	0.604
93174	2.48	4	1.73	5.22508	31.4208	2.25714	0.53194	4.03	5	0.8	2	2	1	2	2	0	1	2	23	1	167	68.1	24.41823	88	0.537
93274	0.08	4	1.22	5.38	196.92	3.465	1.434	5.33	5.4	41	2	2	1	2	2	0	1	2	23	3	161	107.7	41.54932	123	0.992
84490	4.57	4	1.14	5.6224	102.2024	2.894846	1.62192	4.84	5.1	3.8	2	2	1	2	2	0	1	2	28	1	151.8	75.7	32.85128	113.2	0.792
84797	5	4	1.97	5.2027	38.36017	2.045726	0.522365	4.01	5.4	0.6	2	3	1	2	2	0	1	2	25	3	161.5	57.4	22.0073	76.4	0.454
91163	1.69	4	1.66	5.054564	40.8656	2.0962	0.65074	4.06	4.9	1.1	2	3	1	2	2	0	1	2	28	1	158.6	63.7	25.32404	82.4	0.428
84816	3.5	4	1.45	5.16	57.9	2.172	0.835	4.01	5	6.6	2	3	2	2	2	0	1	2	28	3	157.8	45.9	18.43311	71	0.867
88818	3.07	3	1.24	5.5432	95.1684	3.53836	1.48307	5.72	5	5.2	2	3	1	2	2	0	1	2	28	1	151.7	89.8	39.02161	122.4	1.01
83933	1.32	2	1.16	5.603652	105.4054	3.308865	1.66254	5.38	5.2	3.3	2	3	1	2	2	0	1	2	31	3	164.6	90.8	33.51399	102.2	0.982
93213	1.24	1	1.58	5.26916	45.4104	2.46602	0.661233	4.27	5.2	0.6	2	3	1	2	2	0	1	2	32	1	159.3	71.9	28.33331	94.1	0.502
89980	3.3	4	1.09	5.657466	77.2184	2.62169	0.97423	4.42	5.4	0.8	2	3	1	2	2	0	1	2	33	2	168.1	88.3	31.24822	103.1	0.765
88932	1.18	4	1.81	5.221228	51.5746	3.355619	0.833818	5.72	5.1	2.7	2	3	1	2	2	0	1	2	38	2	156.5	72.5	29.6012	103.7	0.724
84073	1.42	3	1.03	5.71	135.54	2.017	1.66	3.8	5.8	12.1	2	3	1	2	2	0	1	2	39	2	158.5	80.2	31.92389	103.1	0.822
88082	3.73	4	1.09	6.09532	135.2092	3.124	1.76844	5.17	5.8	8.6	2	3	1	2	2	0	1	2	44	3	163.9	101.2	37.67234	112.1	0.971
93189	1.64	2	1.97	5.77	52.08	2.922	0.655	5.2	5.2	1.6	2	3	1	2	2	0	1	2	47	1	155.2	78.3	32.50711	106.2	0.668
84025	1.23	4	1.32	5.209168	75.5864	3.369627	1.14844	5.33	5.4	2.2	2	1	1	2	2	0	1	2	18	3	168.2	96.2	34.00346	92.7	1.05
89215	1.38	3	1.37	5.125742	51.7764	1.75698	0.534823	3.36	5	0.08	2	1	2	2	2	0	1	2	19	2	162.5	47.9	18.13964	69.1	0.351
87441	5	4	1.34	6	85.02	1.629	0.395	3.15	5.1	0.6	2	3	1	2	2	0	1	2	19	3	175.9	67.2	21.71889	77.3	0.618
92043	1.09	4	1.47	4.9702	39.37034	2.21828	0.63712	4.16	4.9	0.4	2	3	1	2	2	0	1	2	19	3	166.5	64.2	23.15829	83.1	0.669
84457	2.63	4	1.03	4.27	179.28	1.112	1.073	2.64	4.9	0.4	2	1	2	2	2	0	1	2	19	3	159	71.4	28.24255	99.2	0.774
85284	5	4	2.28	4.66	36.06	1.94	1.31	4.81	5.3	8.4	2	3	1	2	2	0	1	2	20	3	165.6	68.7	25.05163	86.3	0.619
84964	1.99	3	1.58	5.83	112.62	1.733	0.824	3.7	5	0.5	2	3	2	2	2	0	1	2	22	1	161.5	59.2	22.69743	80.3	0.405
84658	1.65	4	1.99	5.15676	42.0672	2.050326	0.5486	4.16	5.2	1.2	2	3	1	2	2	0	1	2	22	4	158.4	41.9	16.69951	58.7	0.668
92815	1.49	3	1.32	5.3408	84.7104	2.03616	0.710411	3.78	5.4	3	2	3	1	2	2	0	1	2	22	2	169.1	101.8	35.60087	117.5	0.675
84494	1.24	3	1.09	5.2704	80.5416	2.48919	0.99215	4.24	4.4	1.5	2	1	1	2	2	0	1	2	22	2	165.6	59.8	21.80623	78.7	0.834
85869	1.6	4	1.5	5.055164	45.8112	2.39566	0.61388	4.34	4.7	0.08	2	3	1	2	2	0	1	2	23	2	160.5	50.5	19.60385	74.5	0.416
85309	2.516035	4	1.37	5.1076	50.16464	3.201629	1.0398	5.51	4.8	0.3	2	3	1	2	2	0	1	2	23	1	156.3	63.9	26.1567	86.8	0.602
88184	5	3	1.47	5.11	61.26	2.586	0.745	4.4	5.7	9.2	2	3	1	2	2	0	1	2	23	3	180.3	92.2	28.36217	94.7	0.635
86226	1.7	4	1.81	5.019849	45.73765	2.35288	0.58884	4.42	5.3	0.4	2	3	1	2	2	0	1	2	23	3	166.1	55.9	20.26154	74	0.708
90883	0.51	4	1.09	5.22	119.82	2.664	1.264	4.32	5.7	12.2	2	1	1	2	2	0	1	2	23	1	163.2	103.2	38.74712	117.2	1.01
86863	5	5	0.96	5.2	42.36	2.87	1.219	4.4	5.2	4.5	2	1	1	2	2	0	1	2	24	1	151.8	66.2	28.7286	86.5	0.763
84539	2.383918	3	1.55	5.05	27.12	1.267	0.632	3.1	4.8	2.1	2	3	1	2	2	0	1	2	25	3	169.5	88.4	30.76896	96.4	0.674

93305	1.5	4	1.11	5.82	342.9	2.780586	1.498586	4.47	5.4	2.5	2	3	1	2	2	0	1	2	25	1	151.2	74.8	32.71885	102.2	0.78
84395	1.24	5	1.68	5.77	74.76	1.888	0.485	3.8	5.2	5.4	2	3	1	2	2	0	1	2	25	3	166.3	106.9	38.65389	112.8	1.08
89832	1.7	5	1.86	5.034773	44.115	1.97596	0.49995	3.83	5.5	1	2	3	1	2	2	0	1	2	26	1	167.3	63	22.50861	82.8	0.48
87088	0.97	4	1.71	5.08056	40.9266	2.11478	0.64778	4.22	5.2	0.9	2	3	1	2	2	0	1	2	27	4	166.4	64.8	23.40283	81.1	0.671
87066	1.73	4	1.76	5.626911	105.0864	2.567145	1.082093	4.68	5.9	6.4	2	3	1	2	2	0	1	2	28	6	154.9	55.3	23.04742	83.4	0.479
84029	2.211723	3	1.78	5.16	43.2	2.689	1.626	5.22	5.3	3.9	2	3	1	2	2	0	1	2	28	1	158.5	51.1	20.34053	77.4	0.518
93606	1.65	4	0.98	5.8164	141.3864	2.30204	1.03418	3.62	5.1	47.2	2	1	1	2	2	0	1	2	28	3	153.6	50.4	21.3623	75.1	0.639
86189	0.99	3	1.71	5.14725	39.1095	2.14956	0.69956	4.14	5	4.5	2	3	1	2	2	0	1	2	28	3	163.2	68.1	25.56859	90.2	0.769
84852	1.57	4	1.11	5.51218	94.818	2.17874	1.143613	3.85	5.3	10.4	2	1	1	2	2	0	1	2	28	3	169.2	74.7	26.09275	96	1.07
88684	2.24	3	1.47	5.3134	79.2106	2.673513	1.045035	4.6	5.3	10.6	2	1	1	2	2	0	1	2	28	1	158	97.7	39.13636	118.1	1.14
89474	4.71	5	1.63	5.4938	121.9674	3.72032	1.076753	5.77	5.5	14.3	2	3	1	2	2	0	1	2	29	3	170.4	119.3	41.0867	127.5	0.637
92938	2.13	5	1.47	5.38	55.5	2.25	0.565	3.98	5.4	0.9	2	3	1	2	2	0	1	2	30	6	155.5	59.4	24.5655	86.7	0.718
86436	1.69	5	1.22	5.33	72.6	2.845	2.19	5.07	5	0.6	2	3	1	2	2	0	1	2	30	3	170.5	87	29.9275	102.6	0.835
89601	1.49	4	1.34	5.33	74.04	2.224	0.542	3.83	5.1	3	2	3	2	2	2	0	1	2	30	3	160.6	89.6	34.73897	103	1.02
86461	1.89	4	1.03	5.4908	116.7875	3.512995	1.343013	5.3	5.8	2.4	2	1	1	2	2	0	1	2	31	2	161.7	97.8	37.40407	125	1.01
89774	2.85	5	1.01	6.33	130.68	3.103	1.355	4.73	4.5	10	2	3	1	2	2	0	1	2	32	3	168	133.3	47.22931	130.4	1.11
87243	0.56	1	1.4	6.1	91.86	2.715	0.756	4.45	5.4	1	2	1	1	2	2	0	1	2	33	2	148	75.5	34.46859	95	0.734
92633	5	5	2.02	5.1636	34.7258	2.09879	0.557719	4.03	4.7	1.4	2	3	1	2	2	0	1	2	34	3	182.3	94	28.28489	108.2	0.805
86520	5	5	1.09	5.05	90.12	1.81	0.677	3.21	5.1	10.6	2	3	2	2	2	0	1	2	39	3	168.7	104	36.54291	113.2	0.989
84766	5	4	0.75	5.4846	106.308	2.09024	1.3954	3.34	5.1	4.8	2	3	2	2	2	0	1	2	40	4	165.7	100.8	36.71263	122	0.654
92767	0.8	1	1.37	5.252946	61.158	3.484237	1.55512	6.05	4.9	0.6	2	3	1	2	2	0	1	2	41	1	153	68.7	29.34769	93.5	0.616
89737	1.8053	1	1.53	5.3456	41.62214	2.2473	0.57256	4.03	4.8	0.3	2	3	1	2	2	0	1	2	46	1	159.4	63	24.79499	82.7	0.408
86476	1.213097	4	1.19	5.239133	71.1366	1.958551	0.83484	3.57	5.4	0.08	2	1	1	2	2	0	1	2	18	4	169.7	62.6	21.73755	70.3	0.471
88234	1.37	5	2.17	5.21065	40.548	2.64057	0.69246	4.94	5.4	0.08	2	3	1	2	2	0	1	2	18	2	154.7	46.1	19.26284	71.4	0.475
90664	1.76	4	1.29	5.144636	46.71768	1.89853	0.654908	3.57	5.1	0.08	2	1	1	2	2	0	1	2	18	3	167.7	65.9	23.43254	81.1	0.542
91862	2.687866	4	2.09	4.88	62.4	2.379	0.203	4.58	5	1	2	3	1	2	2	0	1	2	18	4	159.9	78.8	30.81976	86.1	0.595
85565	1.47	4	1.06	4.63	48.54	1.862	0.858	3.31	4.9	0.8	2	1	1	2	2	0	1	2	18	1	143	40.4	19.75647	63	0.597
84450	0.99	4	1.27	5.126556	57.72	2.263671	0.66276	3.98	5.4	0.08	2	1	1	2	2	0	1	2	18	1	159.7	50.7	19.87916	73.5	0.662
86666	2.19	4	1.86	5	50.46	1.758	0.248	3.72	5.5	0.3	2	1	1	2	2	0	1	2	18	4	158	53.5	21.43086	70	0.698
89613	2.5	4	0.98	5.48388	110.1516	2.08739	1.20304	3.34	5	5.6	2	3	2	2	2	0	1	2	18	3	167.6	114.7	40.83339	125.8	1.06
84973	1.15	4	1.03	5.712666	151.3932	2.316885	1.35372	3.96	5.8	6.3	2	3	1	2	2	0	1	2	18	3	165.3	119.1	43.58791	119	1.53
89934	1.38	4	1.73	5.090467	34.27608	1.88766	0.57012	3.96	5.4	0.08	2	3	1	2	2	0	1	2	19	4	148.3	41.3	18.7788	72.5	0.403
90327	2.48	4	1.22	6	112.74	3.414	1.524	5.33	5.1	2.8	2	3	1	2	2	0	1	2	19	6	153.9	69.8	29.46987	99.3	0.811
90911	0.49	4	1.63	5.273633	38.4924	1.804449	0.568148	3.7	5.6	0.6	2	1	1	2	2	0	1	2	19	1	162.4	62.9	23.84946	82.5	0.944
88360	2.97	3	1.34	5.317742	62.5848	2.104353	0.91125	3.96	5.3	1.8	2	3	1	2	2	0	1	2	20	1	151.9	66.3	28.73413	88.5	0.929
88049	0.85	4	1.22	5.44	107.46	2.56	1.727	4.58	5.2	16.2	2	1	1	2	2	0	1	2	20	3	161.8	100	38.19821	111.8	1.27
92657	1.24	1	1.32	5.145122	49.746	2.64696	0.69817	4.37	5.1	0.08	2	1	1	2	2	0	1	2	24	1	151.7	61	26.50688	83.4	0.475
91927	3.77	5	1.32	5.61	35.22	2.767	0.44	4.29	4.8	0.5	2	3	1	2	2	0	1	2	24	3	162	67.8	25.83448	86.5	0.862
85040	1.65	1	1.58	5.27	18.9	1.681	0.429	3.47	4.9	0.3	2	1	1	2	2	0	1	2	25	1	150.6	46.7	20.5905	77.6	0.324
84187	3.33	3	1.19	5.544	83.0516	2.016915	0.93864	3.8	4.9	0.7	2	3	2	2	2	0	1	2	25	3	160.2	55.4	21.58662	82.4	0.64
84300	4.4916	5	1.29	5.537277	93.2772	3.62834	1.36387	5.72	5.1	2.8	2	3	1	2	2	0	1	2	25	1	154.7	78.2	32.67579	92.7	0.858
89111	2.97	5	1.4	5.4282	72.38364	2.132978	0.784553	3.7	5	4.1	2	3	2	2	2	0	1	2	27	3	155.6	59.7	24.65785	80	0.502
87707	1.24	5	1.42	4.66	25.8	3.233	0.497	4.89	5	0.8	2	2	1	2	2	0	1	2	27	3	169.9	63.5	21.99819	89.4	0.826
92497	2.5904	1	1.19	5.94	91.14	3	1.66	4.94	5.9	11.7	2	3	1	2	2	0	1	2	29	1	140.3	76.1	38.66066	106.8	0.924
88507	0.28	1	1.16	5.6364	122.988	2.402952	1.18914	4.01	5.7	8.5	2	1	1	2	2	0	1	2	32	2	154.9	62.2	25.92314	87.2	0.946
84759	0.89	4	0.88	6	83.58	1.888	0.711	3.1	5.7	3.6	2	3	2	2	2	0	1	2	34	1	155.6	69.1	28.54032	97	0.728
89854	0.02	5	1.22	5.4224	110.374	2.604846	1.21236	4.29	5.4	8.5	2	1	1	2	2	0	1	2	37	1	158.2	82	32.7643	99.2	1.09
89994	1.6	2	1.09	5.5229	89.22908	2.09676	1.01772	3.41	5.6	2.8	2	1	2	2	2	0	1	2	45	2	158.7	85.8	34.06696	100.5	0.988
84065	2.12	4	1.19	5.3312	79.9956	2.80217	1.04192	4.6	5.2	0.2	2	1	1	2	2	0	1	2	48	3	161.4	67.4	25.87336	89.6	0.693
84659	0.52	5	1.4	5.61	69.66	3.414	1.535	5.51	6.2	4.5	2	1	1	1	3	0	1	2	47	4	168.1	136.2	48.19941	151.2	0.777
91400	1.12	5	1.81	6.72	158.7	1.862	1.806	4.5	6.3	116.2	2	3	2	1	3	0	1	2	46	4	173.4	120.7	40.14293	128.7	1.62
88440	2.82	3	1.01	6.5166	171.5202	2.425443	1.58574	4.32	5.9	1.5	2	3	1	1	3	0	1	2	39	3	166.8	88.1	31.66532	103.2	0.79
87905	0.45	2	0.8	6.27	94.336	2.069	1.05	3.36	6	2.3	2	1	2	2	3	0	1	2	42	1	149.3	72.1	32.34563	102.4	0.923
91291	1.03	3	1.5	8.813890	172.0356	2.61084	1.709	4.71	5.6	1.2	2	2	1	1	1	1	1	2	32	7	163.8	103.1	38.4265	127.9	0.697
89813	2.26	4	0.93	6.27	77.28	4.396	2.123	6.31	6.2	37.9	2	3	1	1	1	1	1	2	40	3	152.6	75.2	32.29303	109.2	1.43
87487	1.74	4	1.42	7.9788	115.0135	2.3846	1.47036	4.01	7.3	2.3	2	3	1	1	1	1	1	2	43	1	170.3	89.3	30.79088	106.3	0.579
89639	2.44	5	1.03	10.7	292.56	2.664	1.253	4.27	9.5	5.6	2	3	1	1	1	1	1	2	29	3	178	111.8	35.28595	117.7	0.895
92674	0.74	3	1.4	9.06	97.08	3.724	2.168	6.1	7.8	8.5	2	1	1	1	1	1	1	2	45	1	145.5	57.3	27.06628	86.4	0.899
84006	0.87	2	0.98	8.274690	144.6924	4.40022	2.31678	7.16	6.7	2.8	2	1	1	2	1	1	1	2	45	4	159.8	85	33.28629	104	1.26
85948	0.16	4	1.66	4.83	60.12	3.103	0.881	5.17	5.5	11.7	2	3	1	2	1	1	1	2	38	4	166.4	103.7	37.45175	120.6	0.755

91211	2.68	4	1.01	10.6738	194.864	3.178314	2.02055	5.33	8.6	13.5	2	3	1	2	1	1	1	2	40	1	168.4	149.8	52.82356	149.2	1.18
90745	3.99	4	1.22	8.49	57.72	2.327	2.021	4.47	6.9	26.1	2	3	1	2	1	1	1	2	32	3	168.8	131.6	46.18607	147.8	0.895
83959	0.8	4	1.06	7.395566	226.8276	2.978065	2.1284	5.12	5.1	5.8	2	1	1	2	1	1	1	2	22	3	162.9	126.3	47.59498	129.7	1
90146	0.04	3	1.34	9.226690	103.1472	2.96298	1.64156	5.02	7.2	3.9	2	1	1	1	1	1	1	2	34	4	162.5	72.8	27.56923	94.2	0.964
86323	1.41	2	1.37	5.83	60.78	2.483	1.298	4.45	7.8	4.9	2	1	1	2	1	1	1	2	24	1	157.5	68.7	27.69463	97.2	0.474
89243	3.72	5	1.53	6.1884	107.7768	4.49296	1.91422	9.34	5.7	9.4597	2	3	1	1	2	1	1	2	49	4	168.4	96.7	34.09905	107.6	0.879
88439	5	5	1.27	5.33	84.06	4.267	1.739	6.34	5.5	1.6	1	3	1	1	2	1	1	2	47	3	167.7	113.3	40.28689	109.8	0.78
85173	0.34	1	1.22	5.42	48.78	4.655	1.005	6.34	5.6	4.5	2	1	1	1	2	1	1	2	45	2	153.4	69.1	29.36482	87.3	0.836
87892	1.87	4	1.55	5.83	132.66	2.043	0.937	4.03	5.4	0.7	1	2	1	2	2	1	1	2	29	4	162.9	85.2	32.10683	108.5	0.494
93266	1.19	1	1.01	5.773577	92.1144	4.256391	2.60942	7.14	5.4	1.4	2	2	1	2	2	1	1	2	46	6	148.3	54.1	24.59886	89.6	0.374
88892	2.23	3	0.98	5.831511	177.8079	2.2188	1.17862	3.67	5.5	5.7	2	3	1	2	2	1	1	2	27	4	166.1	82.3	29.8305	93.6	0.879
86453	5	5	1.01	5.4246	99.8832	2.986602	1.67156	5.07	5	0.9	2	1	1	2	2	1	1	2	36	7	157.2	82.7	33.46574	101.5	0.545
85384	3.5208	5	3.31	5.4264	58.23	3.181085	1.09579	6.36	4.7	0.5	2	3	2	2	2	1	1	2	32	7	169.9	75.7	26.22462	99.1	0.322
91091	0.84	4	1.63	5.2333	67.3908	2.139148	0.59576	3.93	5.4	5.7	2	3	2	2	2	1	1	2	22	4	154.6	105.1	43.97276	119.7	0.815
91722	1.65	3	1.58	5.07888	57.3816	2.624063	0.9618	4.76	4.9	2.6	1	1	2	2	2	1	1	2	25	3	170.8	87.7	30.06241	96.8	1.06
91228	0.54	3	1.22	5.05	19.98	3.129	0.531	4.58	5.6	0.7	1	1	1	2	2	1	1	2	31	4	152.5	47	20.20962	70	0.74
90447	0.3	1	1.45	5.4738	61.4736	2.30104	0.74906	3.9	5.6	4.5	1	1	1	2	2	1	1	2	43	3	159.3	58.2	22.93461	78.6	1.24
92423	0.42	4	0.85	6.18385	154.242	2.411571	1.66907	4.03	5.6	9.6	1	2	1	2	2	1	1	2	37	1	158.2	127.5	50.94449	141.6	1.06
89647	1.36	4	1.63	6.94	66.66	3.31	1.084	5.43	5.7	18.5	1	2	1	2	2	1	1	2	47	3	159.6	91.4	35.88231	114.3	1.02
91324	2.09	4	1.68	5.1093	65.5536	2.524958	0.72457	4.53	5	3	1	3	2	2	2	1	1	2	43	3	164	74.1	27.55057	95.6	0.923
85114	2.24	4	1.34	5.26055	69.3252	1.85058	0.6422	3.15	5	2.7	2	1	2	2	2	1	1	2	22	2	171.6	70.2	23.8398	85.4	0.945
89012	0.93	4	1.53	4.94	25.08	1.784	0.35	3.47	5.3	1.1	2	1	1	2	2	1	1	2	24	3	165.7	75.2	27.38879	87	0.61
84989	3.74	3	1.32	6.22	66.42	3.258	0.835	4.97	5.6	2	2	1	1	2	2	1	1	2	37	7	172.1	100.9	34.06665	115.2	0.75
92555	1.15	2	1.27	5.4154	92.92003	2.25816	0.93418	3.65	5.3	12.4	2	1	1	2	2	1	1	2	37	3	162.6	93.5	35.36474	114	0.889
86260	2.99	3	0.83	6.363198	126.373	4.33148	2.62462	7.47	4.9	4.8	2	2	1	2	2	1	1	2	26	3	161.2	97.9	37.67494	111.6	1.05
92206	1.85	4	1.76	5.22805	51.564	2.97151	0.91341	5.22	5.2	0.7	2	3	1	2	2	1	1	2	20	1	160.7	46.5	18.00616	69.4	0.928
83897	1.68	5	1.6	5.11	80.76	2.741	0.452	4.55	5	1.4	2	3	2	2	2	1	1	2	29	3	160.6	79.1	30.668	100.4	0.967
92858	0.19	4	1.78	5.3078	62.8182	3.484065	0.947633	5.66	5.4	1.9	2	3	1	2	2	1	1	2	31	7	161.4	75.1	28.82922	91.2	0.794
85055	5	4	1.27	5.5	63.54	1.862	0.406	3.31	4.8	0.5	2	3	2	2	2	1	1	2	39	3	171.4	59.9	20.38943	78.7	0.849
90384	4.48	4	0.98	5.94	29.34	4.241	2.224	6.23	5.6	5.8	2	3	1	2	2	1	1	2	47	3	167.2	78.6	28.11577	98.1	1.05
91275	2.89	4	1.11	5.943066	122.4348	2.24774	0.91935	3.59	5.7	3.7	2	3	1	2	2	1	1	2	19	3	164.6	55.2	20.37415	74.3	0.957
89823	2.76	4	1.81	5.1318	76.9362	2.562957	0.91462	4.73	4.9	11.7	2	3	1	2	2	1	1	2	21	3	147	55.4	25.63747	76.9	0.633
92239	3.33	5	1.34	5.3542	80.10024	2.140924	0.790601	3.83	5.1	6.8	2	3	2	2	2	1	1	2	22	3	153.9	88.6	37.40731	119.1	0.646
84587	0.77	4	1.11	5.11	103.8	1.758	0.418	3.05	5.6	9	2	3	1	2	2	1	1	2	22	1	158.4	99.7	39.73606	117.8	1.09
91957	0.94	5	1.37	4.55	48.96	2.017	0.406	3.57	5.5	0.08	2	3	1	2	2	1	1	2	23	4	165.2	54.8	20.07985	72.7	0.415
86600	1.26	3	1.58	5.1362	56.8704	2.590596	0.62328	4.45	5.4	1.7	2	1	1	2	2	1	1	2	25	3	160.5	60.7	23.56344	89	0.893
93365	3.2	4	2.28	5.2687	40.002	2.240969	0.553449	4.53	4.9	0.08	2	3	1	2	2	1	1	2	26	3	165.1	60.1	22.04856	83.2	0.466
86029	4.54	5	0.83	6.0978	150.1598	2.79388	1.4969	4.58	5.8	8.3	2	3	1	2	2	1	1	2	26	3	164.9	107.9	39.68077	125.3	1.2
93661	0.74	4	1.09	6.1592	158.0856	2.21292	1.10424	3.57	6.1	24.7	2	1	1	2	2	1	1	2	27	2	157.6	101.8	40.98598	127	0.974
87200	5	5	1.55	5.285324	39.66757	2.270523	0.507586	4.06	5.3	0.08	2	3	1	2	2	1	1	2	28	3	161.7	53	20.2701	73.8	0.461
87510	3.2	5	1.78	5.38	15.96	3.801	0.689	5.9	5	3	2	3	1	2	2	1	1	2	29	3	162.4	59.5	22.5603	81	1.09
92545	5	5	1.53	5.389342	37.72142	1.921117	0.516766	3.36	5.1	0.3	2	3	1	2	2	1	1	2	31	3	167	71.5	25.63735	91	0.578
84628	1.74	4	1.19	4.4	27.48	4.319	1.389	6.15	4.7	3.6	2	3	1	2	2	1	1	2	32	1	145	60.9	28.96552	88.2	0.687
83947	2.67	4	1.81	4.61	34.08	2.689	1.423	5.15	4.8	1.4	2	3	1	2	2	1	1	2	33	3	168.4	73.5	25.9181	96.6	0.309
91997	1.72	4	1.24	5.465766	87.7196	3.480372	1.23966	5.48	5.2	4.1	2	1	1	2	2	1	1	2	35	6	163.7	77.7	28.99504	104.5	1.05
91734	2.06	4	1.45	5.125576	39.8596	1.756916	0.4771	3.57	5.4	0.08	2	1	1	2	2	1	1	2	36	4	174.3	92.9	30.57884	102.6	0.66
88891	4.25	4	1.22	6.245711	128.2338	2.10728	1.17941	3.65	6	6.8	2	3	1	2	2	1	1	2	36	7	166.4	168.6	60.89069	151.2	1.15
92804	2.47	4	1.45	5.3097	60.97404	3.439189	1.18749	5.87	5.1	0.5	2	3	1	2	2	1	1	2	37	4	169.2	111.6	38.98194	116.5	0.627
88182	1.56	4	1.09	6.106592	95.3832	4.28546	1.9785	7.09	5	2.1	2	2	1	2	2	1	1	2	38	7	165.7	119	43.3413	138.2	1.02
90767	0.98	3	1.09	5.5302	92.79	3.064975	1.44354	5.07	5.4	3	2	1	1	2	2	1	1	2	40	1	154	70.1	29.5581	101	0.93
83736	1.23	4	1.37	4.66	32.52	3.672	0.531	5.28	5.6	0.5	2	2	1	2	2	1	1	2	42	4	164.9	55.2	20.30008	80.4	0.721
89659	5	4	2.82	4.33	24.9	1.914	0.395	4.91	5.3	0.08	2	3	1	2	2	1	1	2	43	3	175.1	69.4	22.63535	91	0.661
87852	5	5	1.4	5.6652	87.2624	3.223389	1.293	5.38	5.7	0.2	2	3	1	2	2	1	1	2	48	3	169.5	70.6	24.5734	87.3	0.494
91383	4.94	5	2.59	5.33	34.8	3.698	0.779	6.65	5.4	0.4	2	3	1	2	2	1	1	2	48	3	169.4	72.3	25.19483	90.4	0.644
90398	2.51	4	1.97	5	17.58	1.526	0.406	3.67	5.1	0.3	2	3	1	2	2	1	1	2	23	3	168.1	62.3	22.04716	71.5	0.506
90742	5	4	1.76	4.66	16.38	3.181	0.474	5.15	5.2	0.3	2	2	1	2	2	1	1	2	27	6	152.8	41.9	17.94599	67.2	0.262
85958	0.88	4	0.96	5.27	58.14	3.595	2.777	5.82	4.8	1.2	2	1	1	2	2	1	1	2	32	3	174.5	81	26.60077	108.4	0.987
84547	1.09	4	1.99	5.5224	109.662	2.650425	1.07656	4.84	5.6	7.7	2	2	1	2	2	1	1	2	37	2	153.4	108.4	46.06579	105.4	0.791
87427	0.74	4	1.14	5.5	102.96	1.526	0.768	3.03	5.5	1.5	2	1	1	2	2	1	1	2	22	2	166.1	92.6	33.56385	103.9	0.651
93600	3.01	3	2.25	5.11	66.72	1.94	0.474	4.4	5.1	0.08	1	1	1	2	2	1	1	2	22	3	159.2	48	18.93892	71.2	0.583

86981	1.24	3	1.55	4.61	24.84	1.371	1.005	3.39	4.8	1	1	1	1	2	2	1	1	2	27	3	171.4	61.3	20.86598	76	0.793
89233	2.06	3	0.67	6.1072	156.8064	2.25658	1.41354	3.47	5.4	21.1	1	1	1	2	2	1	1	2	31	3	158.2	86.5	34.56234	118.5	1.01
85343	0.83	3	1.34	5.44	87.84	1.991	1.039	3.8	5.7	0.9	1	1	1	2	2	1	1	2	21	1	164.4	85.9	31.78261	99.2	0.857
88123	2	5	1.71	5.256628	38.1756	1.863485	0.57246	3.39	5.1	0.9	1	1	1	2	2	1	1	2	26	3	171.1	64.6	22.06645	83.5	0.71
85160	0.34	4	1.45	5.33	47.4	1.629	0.576	3.34	4.2	0.7	2	1	2	2	2	1	1	2	26	3	161.2	59	22.70502	79.4	0.656
87030	0.13	4	2.12	5.5546	89.2788	3.51442	1.35592	5.95	5.5	3.6	2	2	1	2	2	1	1	2	36	3	161.5	75.9	29.10025	93.5	1.07
85361	1.38	1	1.84	5.263256	31.9992	2.33674	0.5523	4.34	5.5	0.4	2	2	1	2	2	1	1	2	43	1	154.5	58.5	24.50749	90.5	0.588
85390	2.88	3	1.97	5.1524	42.708	2.249516	0.54054	4.37	5.2	0.5	2	3	1	2	2	1	1	2	23	1	162.3	78.3	29.7252	97.2	0.767
86159	3.145357	4	2.17	5.2855	38.7704	2.064878	0.501131	4.24	5	0.6	2	3	1	2	2	1	1	2	20	2	165.1	69.8	25.60715	82.3	0.708
85599	0.85	4	1.99	6.11	61.56	2.896	0.982	5.33	5.8	0.4	2	2	1	2	2	1	1	2	21	7	167.3	61.4	21.93696	81.4	0.563
86022	0.55	4	1.47	5.05	19.2	1.681	0.282	3.28	4.8	1	2	1	1	2	2	1	1	2	23	3	161.1	80.8	31.13295	94	0.453
88601	0.6	3	0.98	5.5267	123.9384	2.19736	1.32812	3.8	5.3	11.2	2	1	1	2	2	1	1	2	24	1	159.6	67.1	26.34249	91.8	1.08
91721	1.252533	4	1.06	5.4612	94.0728	2.1456	1.3126	3.34	4.4	13.2	2	1	2	2	2	1	1	2	26	3	158.4	72.1	28.73591	90	1.32
85138	0.78	4	1.11	6	131.28	2.901685	1.48778	4.81	5.2	6.2	2	1	1	2	2	1	1	2	31	3	160.9	85.9	33.18036	103.1	0.987
89637	0.11	1	1.11	5.5	48	3.284	0.937	4.84	5.2	1.9	2	1	1	2	2	1	1	2	40	1	151.9	63.4	27.47728	96.2	0.802
87238	2.67	4	1.73	5.189666	54.04656	2.983202	0.7633	4.97	5.5	1.2	2	3	1	2	2	1	1	2	19	2	166.1	56.3	20.40653	74	0.321
87423	0.52	3	1.71	5.07403	25.50890	1.644770	0.56026	3.23	4.9	0.8	2	1	1	2	2	1	1	2	31	3	162.8	65.7	24.78886	85.5	0.575
88261	0.37	3	1.47	5.94	79.56	2.638	1.14	4.63	6.2	8.6	2	1	1	2	2	1	1	2	31	2	162.2	78.7	29.9139	109	0.898
90266	0.4	2	1.4	5.11	69.36	3.195605	1.00448	5.17	5.4	2	2	1	1	2	2	1	1	2	33	1	151.6	54.1	23.53959	85.4	0.808
88451	0.68	1	1.06	5.54	50.82	2.793	0.711	4.19	5.4	3.7	2	1	1	2	2	1	1	2	34	1	154.7	78.6	32.84293	99.5	0.742
85222	1.9	4	1.06	5.310942	82.7724	2.113793	0.9861	4.03	5.1	0.3	2	1	1	2	2	1	1	2	35	3	164.8	67.1	24.70632	95	1.04
89036	2.39	5	1.06	5.6508	125.982	3.64531	1.98914	5.87	5.4	4.1	2	3	1	2	2	1	1	2	46	3	160.9	76.4	29.51082	104.6	0.875
92455	0.3	5	1.01	5.573	115.794	3.69122	1.65142	5.61	5.8	7.1	2	1	1	2	2	1	1	2	49	1	155	71.5	29.76067	105	1
85453	1.05	2	1.09	4.27	19.86	2.922	1.547	4.71	5.2	4.1	1	1	1	2	3	1	1	2	42	2	162	64.5	24.57705	94	1.42
86011	5	3	1.6	6.05	52.8	2.612	0.847	4.6	5.6	2	2	3	1	2	3	1	1	2	45	6	144.2	48.9	23.51681	79.9	0.797
89712	2.82	4	1.01	5.83	108.42	4.163	0.813	5.53	7.7	2.6	2	3	1	1	1	2	1	2	48	4	162.7	91.1	34.41464	122.6	0.544
87281	1.62	1	0.93	5.88	22.68	2.017	1.445	3.62	5.9	3.8	1	1	1	1	2	2	1	2	48	3	154.7	53.5	22.35492	88.5	1.01
91475	1.96	3	0.85	5.581	108.9072	2.308516	1.46676	3.75	5	18.3	2	1	2	2	2	2	1	2	24	2	152.9	129.7	55.47854	150.6	1.1
90994	1.68	3	1.42	5.38	30	1.94	0.361	3.52	5.2	0.7	2	1	1	2	2	2	1	2	21	3	167	61.6	22.08756	73.5	0.693
88540	0.53	2	1.27	5.3324	69.144	2.05144	0.8282	3.44	4.8	1	2	1	2	2	2	2	1	2	22	3	170.3	76.7	26.44637	101.6	0.706
84716	1.07	2	1.01	5.38	106.14	3.646	1.761	5.46	5.5	13.7	2	3	1	2	2	2	1	2	44	3	161.8	110.1	42.05622	120.6	1.12
87799	4.72	5	1.53	5.5955	114.2268	3.85634	1.1345	6.1	5.6	13.6	2	3	1	2	2	2	1	2	48	2	162.7	101.9	38.49454	116.2	1.67
85591	1	4	1.4	5.2	78.72	2.922	1.614	5.07	5.6	2	2	1	1	2	2	2	1	2	18	6	154.7	75.4	31.50582	97.5	0.555
90842	1.61	4	1.22	5	113.7	2.043	0.813	3.62	5	0.2	2	1	2	2	2	2	1	2	25	3	158.8	84.5	33.50856	104.3	0.67
85936	2.52	4	1.78	5.2	26.1	2.379	1.005	4.63	5	4.9	2	3	1	2	2	2	1	2	27	2	162.3	58.3	22.13255	79.8	0.683
85438	5	4	1.24	5.4522	68.71064	2.650366	0.91345	4.65	5.1	0.8	2	3	1	2	2	2	1	2	45	3	173.6	86.2	28.60275	104.9	0.516
87501	2.61	2	1.68	5.472366	57.88056	3.05094	0.92869	5.09	5.3	0.7	2	1	1	2	2	2	1	2	36	4	163.2	51.5	19.33601	71	0.684
88254	0.34	4	1.68	4.97	22.68	2.405	0.655	4.4	5.3	1.9	2	2	1	2	2	2	1	2	19	2	158.4	50.3	20.04738	72.3	0.271
93222	2.68	3	2.43	5.277348	60.2004	4.225675	1.427286	6.96	5.2	4.3	2	1	1	2	2	2	1	2	22	3	164	67	24.91077	89	0.721
85611	2.06	3	1.76	5.18478	39.3372	2.717236	0.69546	4.91	5	0.08	2	3	1	2	2	2	1	2	20	6	154.2	39	16.40196	65.6	0.577
87895	0.69	1	1.58	5.27	8.94	2.146	0.553	3.98	5.5	0.9	2	1	1	1	2	3	1	2	37	3	163.5	72.2	27.00858	95.5	0.812
86490	2.1386	5	1.14	5.390766	116.9796	3.64886	1.5912	5.9	5.4	14.7	2	1	1	2	2	3	1	2	31	3	167.9	98.5	34.94096	115.4	1.36
85676	2.38	4	1.16	5.66	101.82	2.431	0.542	3.85	5.6	4.8	2	3	1	2	2	3	1	2	32	4	165.2	111.4	40.81926	112.3	1.04
88504	0.66	2	0.96	4.94	87.9	3.569	0.756	4.86	5.3	2.7	2	1	1	2	2	3	1	2	22	3	156.6	104.5	42.61208	122	0.787
87697	5	4	1.81	5.05	41.4	2.606183	0.69721	4.91	4.7	0.2	2	3	1	2	2	3	1	2	37	3	182.6	70.8	21.234	84	0.677
93050	1.19	4	1.22	5.11	140.46	3.801	0.531	5.25	5.1	4	2	1	1	2	2	3	1	2	20	4	170	80.1	27.71626	94	0.988
89362	5	5	1.53	5.33	82.98	2.793	0.824	4.71	5.8	18.8	2	3	1	2	2	3	1	2	41	3	156.3	114.4	46.82827	109.2	0.93
84128	0.59	4	1.58	5.14	71.76	1.914	0.576	3.75	5.9	5.4	2	3	1	2	2	3	1	2	46	1	163.7	78.7	29.3682	95.5	0.902
89620	0.68	3	0.98	5.5	87.42	2.043	2.529	4.19	5.1	3.4	1	3	1	2	2	3	1	2	27	7	166.9	99.2	35.61222	108	0.974
88205	0.17	3	1.47	5.1282	77.55	2.20476	0.68864	3.78	5.1	12.8	2	1	2	2	2	3	1	2	23	1	176.6	133.8	42.90172	138.3	1.11
85631	3.7	3	1.5	5.269276	39.963	2.070052	0.502815	3.59	5.1	0.08	2	1	1	2	2	3	1	2	20	2	170.3	54.2	18.68831	75.2	0.389
88707	1.1	2	1.6	5.55	61.68	3.129	0.779	5.09	5.6	0.5	2	1	1	2	2	3	1	2	23	4	151.4	61.5	26.83017	94.5	0.645
92124	1.58	4	1.29	5.38	167.4	2.819	1.434	4.76	5.8	17.6	2	1	1	2	3	3	1	2	42	4	158.6	109.2	43.41265	121.2	0.884
84298	5	5	1.71	4.66	34.08	2.379	1.016	4.55	5.1	1.3	2	3	1	2	1	0	1	2	36	6	158.1	59.9	23.96421	91	0.674
91826	0.95	3	1.11	5.493	87.0924	2.12192	0.9188	3.44	5.2	18.9	2	1	1	2	2	1	1	2	42	4	151.3	62	27.08406	92.2	0.986
87473	0.74	3	2.2	6.38	82.62	2.741	0.858	5.33	6.6	1	2	2	1	2	2	1	1	2	44	2	166.4	89.1	32.17889	113.5	0.644
84841	0.2	3	2.09	5.046148	60.1386	4.27666	1.7613	8.82	3.8	1.3	2	3	1	2	2	0	1	2	27	4	155.3	55.9	23.17762	85.1	1.6
90422	3.2132	4	1.63	5.94	46.92	3	0.914	5.04	5.5	3.9	2	3	1	2	2	0	1	2	42	4	165.5	73.9	26.9804	88.4	0.709
86697	0.21	5	1.06	5.16	124.8	3.75527	1.80138	5.97	5.8	5.6	2	1	1	2	2	0	1	2	42	4	161.4	99.4	38.15745	108.8	0.95
85477	2.09	4	1.14	5.884377	93.9516	3.71562	1.99718	6	6	1.6	2	3	1	2	2	1	1	2	44	1	156.6	75.6	30.8275	101.1	0.847

92065	2.48	5	1.32	7.66	283.08	2.06962	1.10958	3.28	5.6	3.9	2	3	2	2	2	0	1	2	47	2	157.4	60.4	24.37965	76.7	1.03
84937	0.42	2	1.32	5.6198	57.40662	2.905093	0.750256	4.55	5.4	0.6	2	1	1	2	2	0	1	2	26	4	173.1	73.3	24.46301	80.8	0.807
89080	1.086680	4	0.93	5.566316	103.8533	2.80634	1.2204	4.29	5.8	1.7	2	1	1	2	2	0	1	2	34	3	166.2	69.3	25.0883	87.4	0.949
90726	2.16	5	2.12	5.224727	42.3744	2.77965	0.66124	5.02	5.3	0.08	2	3	1	2	2	0	1	2	32	6	159.5	53.8	21.14759	76.4	0.493
92784	2.46	4	1.91	5.1299	46.29034	3.111983	0.75422	5.61	4.9	0.2	2	3	1	2	2	0	1	2	23	3	166.9	74.6	26.78096	93	0.633
84487	2.2	4	2.66	5.61	33.9	2.405	0.361	5.22	4.9	0.5	2	2	1	2	2	0	1	2	24	3	171	56	19.15119	70.8	0.699
86632	0.33	2	1.37	5.511666	100.5432	2.39366	0.90032	4.19	5.4	11.1	2	1	1	2	2	0	1	2	24	4	153.1	78.8	33.61831	94.6	0.994
91101	0.9096	3	1.55	5.20195	35.44610	1.496756	0.55598	2.97	5	0.5	2	1	2	2	2	1	1	2	25	4	161.8	61.4	23.4537	83.1	0.8
88580	0.85	4	1.11	5.5	67.08	1.577	0.835	3.08	5.8	3.6	2	1	1	2	2	1	1	2	27	2	159.8	91.1	35.67507	108.2	0.917
87802	2.02	5	0.91	6.1176	180.1248	2.24002	1.16764	3.41	6.1	28.8	2	1	1	2	2	0	1	2	27	4	175.6	162.1	52.56952	143.3	0.924
88147	3.3	4	1.14	5.631733	96.7482	3.592835	1.56192	5.66	5.1	3.5	2	3	1	2	2	0	1	2	27	3	161.7	101.4	38.7809	121	1.1
91531	0.74	1	0.98	5.8306	124.8984	3.335705	2.20747	5.38	5.4	5.1	2	1	1	2	2	1	1	2	28	1	153.6	71.2	30.17849	103.2	0.797
83802	2.4012	3	1.84	5.203	55.5606	2.86002	0.78236	5.15	5.2	1.8	2	3	1	2	2	0	1	2	29	1	169.8	68.4	23.72361	93	0.674
88583	1.67	4	1.11	5.340566	80.46	3.77042	1.80068	6.03	4.8	4.4	2	1	1	2	2	1	1	2	31	4	160.2	102.3	39.86122	114.6	1.06
91139	1.2	4	1.45	5.9248	110.3445	4.06728	1.72942	6.44	5.7	8.330066	2	1	1	2	2	0	1	2	32	3	167.9	87.2	30.93251	100.5	0.707
93665	4.12	5	2.12	5.208533	29.9802	2.030386	0.463432	4.06	4.7	0.4	2	3	1	2	2	0	1	2	34	3	169.6	69	23.98819	91.5	0.649
85627	2.15	3	1.4	5.2566	115.1496	2.852741	1.05736	4.84	5.2	18.9	2	1	1	2	2	0	1	2	35	3	165.8	65.6	23.86354	95.3	0.91
86876	4.94	5	1.84	5.27	32.28	3.051	0.677	5.2	5.1	0.2	2	3	1	2	2	0	1	2	38	6	155.8	61.4	25.29493	83.9	0.605
86613	3.52	1	1.66	5	18.06	2.302	0.452	4.16	4.8	0.5	2	3	1	2	2	0	1	2	38	3	162.9	58.1	21.89445	78.1	0.637
87171	1.41	4	1.63	5.1887	43.5852	2.399722	0.7229	4.32	5.1	4.5	2	3	1	2	2	0	1	2	40	1	156.5	66.1	26.98813	85.4	0.836
85759	4.99	4	1.16	5.573866	84.7212	4.01716	1.80168	6.08	5.3	5.7	2	3	1	2	2	0	1	2	43	2	155.7	78.3	32.29866	90.3	0.903
93242	0.49	3	2.07	5.345257	74.0262	2.37148	0.79352	4.27	5.6	7.5	2	1	1	2	2	1	1	2	46	4	178.7	67.7	21.20018	82.2	0.909
86482	0.98	1	0.75	5.61	107.94	4.577	3.218	6.8	5.4	18.9	2	2	1	2	2	0	1	2	46	3	166.7	88.3	31.77529	121.3	0.923
84156	0.35	4	1.37	5.31	84	2.586	0.655	4.27	5.9	17.4	2	1	1	2	2	0	1	2	29	4	166.8	92.9	33.39055	108	1.09
92970	2.82	4	0.93	5.11	47.76	2.095	0.971	3.47	5.1	1.2	2	1	1	2	2	0	1	2	25	3	165.5	73.5	26.83437	93.8	1.08
91559	0.25	2	1.06	5.555	93.708	2.351778	1.36498	4.06	5.4	2.5	2	1	1	2	2	0	1	2	30	3	163	66.8	25.14208	95.9	0.919
83909	1.132366	4	1.4	5.77	99.78	2.586	0.565	4.24	5.9	3.3	2	1	1	2	2	0	1	2	49	3	159.1	95.7	37.80694	117.6	1.14
92770	2.22	4	1.14	5.5	69.96374	1.629	0.723	3.1	5.2	0.3	2	1	1	2	2	0	1	2	18	3	168.4	111.1	39.17688	102.7	0.569
85805	1.11848	3	1.6	5.5	72.72	2.353	0.497	4.19	5.2	2.4	2	1	1	2	2	0	1	2	18	3	172.5	91.3	30.68263	102.2	0.61
88840	5	4	1.99	5.117989	44.5974	2.125866	0.65718	4.01	5.1	6.3	2	3	1	2	2	0	1	2	20	4	167.2	94.6	33.83908	101	0.87
85095	2.87	3	0.93	5.48	53.52	2.198	0.824	3.52	5.1	3.1	2	3	1	2	2	0	1	2	22	2	155.4	56.8	23.52049	82.4	0.957
85556	0.63	3	1.24	4.39	29.4	2.5854	0.85286	4.42	5.4	1.3	2	1	1	2	2	0	1	2	24	6	155.3	59.1	24.50443	81.3	0.545
90692	0.82	3	0.83	5.9276	147.0348	2.00168	1.48208	3	4.8	7.3	2	1	2	2	2	0	1	2	30	3	157.1	72.9	29.5376	97	1.15
91113	1.36	1	2.02	5.27	25.74	3	0.237	5.12	5.3	0.5	2	2	1	2	2	0	1	2	31	3	162.3	68.6	26.04276	89.2	0.585
86279	1.05	4	1.84	5.1465	48.684	2.661433	0.85372	4.86	5	4.8	2	3	1	2	2	0	1	2	35	3	168.8	76.4	26.81319	87.3	0.752
93619	1.72	2	1.91	5.44	60.6468	3.077	0.677	5.3	5.6	3.7	2	3	1	2	2	0	1	2	44	6	149.3	51.9	23.28347	81.4	0.89
88164	3.52	4	0.96	6.39	162	3.026	2.687	5.22	5.9	1.4	1	1	1	1	1	0	2	2	45	2	158.6	98.8	39.27811	113.8	0.782
84765	0.75	2	1.03	12.83989	198.4491	4.535414	3.03892	6.8	10.9	13.3	2	2	1	1	1	0	2	2	44	2	154.3	103.2	43.34588	113.8	0.789
86167	5	4	1.34	5.22	54.36	3.258	1.716	5.38	5.6	5.8	2	3	1	1	1	0	2	2	36	1	160.6	84.3	32.6841	107.8	0.766
84915	3.45	4	0.72	8.3372	169.1868	2.53828	2.27584	4.22	7.5	7.9	2	3	1	1	1	0	2	2	45	1	161.3	92.2	35.43743	110	0.954
87050	0.75	4	1.37	9.68	355.8	4.577	1.355	6.57	15	50.5	2	2	1	1	1	0	2	2	46	7	153.7	108.6	45.9708	130.1	1.33
93511	0.99	3	1.11	16.8	75.42	2.948	1.208	4.6	12.2	1.5	2	1	1	1	1	0	2	2	40	4	167.3	98.8	35.29922	129.7	1.12
89314	0.91	4	1.16	14.3	199.9284	4.112	1.852	6.13	9.9	11.091	2	1	2	1	1	0	2	2	39	4	169.6	104.1	36.19087	128.8	1.04
88614	1.19	4	1.4	7.1073	213.7536	2.687411	1.74566	4.55	5.5	4.5	2	3	1	1	1	0	2	2	35	2	156.4	69.1	28.2491	91.5	0.504
93024	3.3	5	1.37	13.9	78.54	3.388	0.756	5.09	11.1	4.4	2	3	1	1	1	0	2	2	40	4	168.4	80	28.21018	102.1	0.742
85224	4.71	4	1.34	10.8392	207.4968	2.776526	1.59228	4.55	8.7	4.3	2	3	1	1	1	0	2	2	48	1	155.5	93.8	38.79199	128.5	0.625
86350	4.94	3	1.45	11.8	600.36	3.026	1.389	5.12	8.9	9.3	2	3	1	1	1	0	2	2	49	1	163.2	89.8	33.716	116.5	0.581
92184	2.51	1	1.19	9.66	114.54	1.991	2.687	4.42	6.5	0.4	2	3	1	1	1	0	2	2	40	1	137.6	55.1	29.10146	89.5	0.596
88689	2.16675	2	1.22	7.645290	171.012	2.126693	1.33206	3.13	6.8	1.5	2	3	1	1	1	0	2	2	41	1	147.3	71.7	33.0456	100.7	0.626
91842	0.42	1	1.19	6.55	60.78	4.474	2.224	6.67	6.1	5.5	2	1	1	1	1	0	2	2	49	1	161.1	78.1	30.09262	104	0.82
85912	0.75	3	1.37	8.061390	176.1072	2.52244	1.30832	4.29	5.9	5.3	2	1	1	2	1	0	2	2	49	4	166.4	122	44.06088	142	0.789
86127	0.5	2	1.03	14.7	38.7	4.655	1.084	6.18	11.3	6.4	1	1	1	2	1	0	2	2	46	4	172	93.9	31.74013	115.8	0.838
84685	1.76	3	1.66	5.55	46.8	2.379	0.406	4.22	4.9	2.8	1	1	1	2	1	0	2	2	27	3	166.6	70.4	25.36429	89.5	0.841
88352	0.04	1	1.24	11.8752	138.9456	4.490554	2.124	7.09	9.3	4.8	2	1	1	2	1	0	2	2	27	1	159.3	89.4	35.22946	103.4	0.866
87645	2.67	2	0.98	7.084490	231.492	2.84506	1.99128	4.91	6	2.9	2	3	1	2	1	0	2	2	42	3	164.3	104.8	38.82274	121	0.83
88199	1.23	3	1.45	21.6	46.5	3.595	0.779	5.4	14.4	13	2	1	1	2	1	0	2	2	29	4	167.8	96.1	34.13025	119.2	1.02
88589	3.09	4	1.14	7.234866	239.5953	2.18002	1.0926	3.65	5.8	25.3	2	3	1	2	1	0	2	2	37	4	162.4	110	41.70812	130.6	1.16
90700	2.28	5	1.55	11.8	739.5	3.595	0.926	5.56	10.7	25.3	2	3	1	2	1	0	2	2	40	4	168.5	100	35.22088	110.5	1.26
89223	3.22	4	1.53	7.518666	141.936	2.4396	1.16284	4.19	6.5	1.9	2	3	1	2	1	0	2	2	43	3	166	100.9	36.61634	108.3	0.829
83911	3.3	4	1.63	18.4	42.18	2.017	0.474	3.85	9.9	5.4	2	3	1	2	1	0	2	2	43	4	172.3	91.1	30.68653	101.6	1.04

87665	1.1328	2	0.7	8.469690	301.9923	2.84302	4.09895	5.17	5.5	12.9	2	2	1	2	1	0	2	2	44	1	148.8	70.2	31.70525	102	1.34
83837	2.18	2	1.34	12.5906	231.3384	3.00676	1.60052	4.99	10.7	8	2	3	1	2	1	0	2	2	45	1	148.3	77.5	35.23866	107.6	0.904
87874	3.4	3	0.72	12.74889	183.6768	3.921194	2.78056	5.64	11.3	1.6	2	1	1	2	1	0	2	2	46	4	156.3	78.7	32.2149	110.6	0.782
92812	1.76	4	1.09	6.61	84.9	2.198	0.666	3.59	5.6	5.4	2	3	1	2	1	0	2	2	37	1	158.5	103.8	41.31796	123.9	0.764
91630	1.65	2	0.91	5.76	261.42	2.431	1.287	3.93	5.7	11.9	2	3	1	2	1	0	2	2	29	1	163.8	121.4	45.24711	139.5	0.968
91771	0.61	1	1.11	8.55	92.34	2.845	1.377	4.58	6.7	4.2	2	3	1	1	1	0	2	2	39	1	158.8	74.6	29.5827	92.9	0.659
89305	1.98	4	1.42	8.0516	160.6296	1.98858	1.0199	3.21	5.4	1.9	2	3	1	2	1	0	2	2	32	4	169.1	80.2	28.04705	104.1	0.994
83924	2.5	1	1.11	7.44	179.52	4.474	1.298	6.18	6.8	36.9	2	3	1	1	2	0	2	2	48	3	162.4	93.8	35.56565	122.4	1.32
89810	0.6	3	1.5	6.5876	216.3636	2.5818	1.67946	4.4	6.2	13.3	2	1	1	1	2	0	2	2	24	4	167.4	181	64.59035	171.6	1.27
91945	1.38	5	0.75	6.7354	164.2752	4.39612	3.0443	7.53	6.7	2.3	2	2	1	1	2	0	2	2	39	2	163.4	119.3	44.68238	120.5	0.571
85994	5	5	1.71	6.49	42.96	4.06	0.587	6.03	5.1	1	2	3	1	1	2	0	2	2	43	1	162.2	77.9	29.60982	98	0.887
86238	0.99	5	1.91	5.9446	71.916	2.63721	0.974428	4.55	5.6	3.4	2	3	1	1	2	0	2	2	45	4	165.5	64.7	23.62154	88.6	1.02
91627	5	3	1.5	5.93	42.18	4.034	1.716	6.31	5.4	4.3	1	1	2	1	2	0	2	2	48	3	177.6	101.7	32.24297	112.1	1.02
92413	5	4	1.45	6.27	83.28	4.19848	4.9	7.24	5.9	4.4	2	2	2	1	2	0	2	2	45	1	153.3	79.5	33.82851	93.7	0.808
90852	5	5	0.98	5.77	152.52	2.767	2.856	5.07	5.5	4.2	1	3	1	1	2	0	2	2	34	6	165.8	72.4	26.33719	96.1	0.759
85104	2.11	2	1.16	6.1081	110.7626	3.086786	1.37464	4.97	5.3	2.8	2	1	1	1	2	0	2	2	29	1	156.8	62.3	25.33938	91.1	0.907
92220	1.9	5	1.19	11	68.58	3.077	1.581	4.99	9.4	4	2	1	1	1	2	0	2	2	39	6	158.8	80	31.72408	105.8	0.651
86908	5	3	0.91	5.59	83.94	4.189	3.026	6.49	5.2	1.6	2	2	1	1	2	0	2	2	41	1	156.7	65	26.47131	92	0.813
87031	2.82	5	1.34	5.8898	71.8104	2.640828	1.127368	4.37	5.4	2.2	2	3	1	1	2	0	2	2	48	3	153.1	86.2	36.77536	100.5	0.769
87289	1.19	3	1.24	5.323128	60.4404	2.524418	0.842	4.16	4.9	0.4	1	1	1	2	2	0	2	2	37	4	153	45.1	19.26609	71.8	0.652
84046	5	5	1.53	5.967748	140.2464	4.46144	1.495826	7.5	5.9	12.8	1	3	1	2	2	0	2	2	45	3	165	90.4	33.20478	114.5	1.62
87901	1.2939	3	1.45	5.77	74.22	2.664	0.892	4.53	5.2	2.4	2	1	1	2	2	0	2	2	38	3	164.4	83.2	30.78362	96.4	0.983
91443	2.493333	5	0.93	6.16	232.5	3.155	0.813	4.45	5.9	27.4	2	1	1	2	2	0	2	2	45	4	163.7	121.1	45.19046	136.6	1.22
88565	1.09	4	1.24	5.4862	59.9224	3.202263	1.0055	5.25	5.3	1	2	2	1	2	2	0	2	2	46	3	156.7	87.2	35.51227	106.1	0.944
92542	5	5	1.71	5.2222	68.436	2.894446	0.986443	5.07	4.6	7.1	2	3	1	2	2	0	2	2	36	3	169.1	85.1	29.76065	110.7	0.608
91834	2.59	4	1.71	5.2918	54.5756	2.593925	0.5649	4.45	5.4	2.9	2	3	1	2	2	0	2	2	39	4	167.2	99.2	35.48454	106.5	0.66
91253	2.64	3	1.37	6.05	130.02	2.534	1.095	4.4	5.4	8.4	2	3	1	2	2	0	2	2	44	3	159.8	98.5	38.57293	120.7	0.857
89241	5	5	1.16	6.33	143.1	3.129	1.795	5.12	6	10.8	2	3	1	2	2	0	2	2	44	4	162.5	134.2	50.8213	137.4	1.49
90970	1.41	1	1.24	5.920002	106.578	2.772466	1.15562	4.53	5.7	3.4	2	3	1	2	2	0	2	2	46	1	139	72.8	37.67921	101.2	0.624
92010	0.85	3	1.37	4.72	203.52	2.457	0.655	4.14	5.3	2	2	1	1	2	2	0	2	2	18	4	151.3	84.2	36.78191	107	0.927
91284	4.42	4	1.71	4.88	53.22	2.534	0.452	4.45	5.1	2	2	3	1	2	2	0	2	2	29	3	155.9	61.5	25.30363	83.4	0.772
91138	1.69	4	1.97	5.3679	54.2337	2.225766	0.55066	4.14	5.7	9.1	2	1	1	2	2	0	2	2	35	4	170.2	112.5	38.8359	108.5	0.448
86810	2.29	4	0.88	5.33	66.18	3.155	0.373	4.22	5.5	19	2	1	1	2	2	0	2	2	36	7	159.8	95.5	37.39812	121.2	1.12
84672	1.53	3	1.78	5.5076	78.45648	2.68216	0.809343	4.53	5.6	7.2	2	1	1	2	2	0	2	2	39	4	158.7	88.2	35.01989	107.9	0.933
91338	1.23	4	1.99	5.311328	71.5906	3.18906	0.926045	5.46	5.3	4.7	2	3	1	2	2	0	2	2	40	2	151.3	54.2	23.67671	81.1	0.377
85702	1.65	3	1.11	5.65	84	2.534	0.881	4.06	5.2	3.4	2	1	1	2	2	0	2	2	40	1	150.8	83.5	36.7184	93.7	0.735
89031	3.92	4	2.02	5.234583	36.31594	2.330356	0.517809	4.45	4.9	0.4	2	3	1	2	2	0	2	2	41	7	162	76.7	29.22573	84.1	0.528
88230	3.33	4	1.78	5.151	42.98523	2.82136	0.77457	4.94	5.1	0.8	2	3	1	2	2	0	2	2	42	3	151.4	57.1	24.91061	88.1	0.7
89348	1.41	4	2.17	5.1212	41.6472	2.269601	0.559787	4.37	5.3	0.6	2	1	1	2	2	0	2	2	43	4	166.7	74.7	26.88125	86.7	0.566
90873	5	5	1.71	4.39	44.46	2.948	0.621	4.94	5.1	0.9	2	3	1	2	2	0	2	2	46	4	163.4	64.6	24.19516	81.9	0.524
91900	5	5	1.47	5.27	242.28	3.077	1.219	5.12	5.8	12.6	2	3	1	2	2	0	2	2	46	1	162.3	96.4	36.59654	119.5	1.05
86210	2.49	3	1.16	4.83	22.98	3.026	0.406	4.37	5.6	0.5	2	1	1	2	2	0	2	2	47	4	167.7	104.9	37.30005	102.4	0.645
90889	5	4	0.98	6.49	98.22	2.689	3.872	5.46	5.8	4.1	2	2	1	2	2	0	2	2	47	3	166.8	94.3	33.89375	107.8	0.693
90542	5	4	1.55	5.14	46.08	3.103	0.632	4.94	5.5	0.4	2	3	1	2	2	0	2	2	47	4	156.9	63	25.59144	79.6	0.72
85858	5	3	1.22	5.746377	84.41493	2.911489	1.133875	4.76	5.6	1.3	2	3	1	2	2	0	2	2	48	4	162.7	93.4	35.28351	112.3	0.674
88964	1.98	3	0.67	6.4882	237.4272	2.40294	1.84592	3.72	5.4	48	2	1	1	2	2	0	2	2	19	7	161.4	142.5	54.70258	134.9	1.36
83903	2.08	4	1.16	5.38	111.18	2.095	0.881	3.67	5.3	15.9	2	3	1	2	2	0	2	2	47	4	161.1	110.2	42.46103	123.8	0.943
86572	0.85	4	1.01	5.2698	79.8504	3.16944	1.61788	5.15	5.1	0.4	2	1	1	2	2	0	2	2	40	6	148.7	47.7	21.5723	79	0.628
87711	4.74	5	2.17	5.31	65.52	2.405	0.689	4.89	5.1	3	2	3	1	2	2	0	2	2	46	1	149.7	82.9	36.99227	111.7	0.709
88361	0.94	3	0.93	5.16	103.56	4.086	1.298	5.61	6	2.3	2	1	2	2	2	0	2	2	36	7	163.4	90.3	33.82078	102.8	0.886
88542	3.98	5	1.53	5.88	106.8	2.56	0.542	4.34	5.1	0.7	2	3	2	2	2	0	2	2	28	4	164.1	58	21.53827	74.1	0.653
88625	2.99	4	1.32	5.61	73.44	2.508	0.881	4.24	5.2	11.2	2	3	2	2	2	0	2	2	33	4	159.6	99.9	39.21929	113.1	0.912
91642	3.22	5	1.55	5.500657	63.1896	3.955849	1.071614	6.31	5.1	0.4	2	3	2	2	2	0	2	2	34	3	170.4	71.3	24.55559	99	0.847
89642	5	4	1.78	5.42	50.28	2.353	0.768	4.5	5	2.7	2	3	2	2	2	0	2	2	37	3	168.6	103.2	36.30484	112.5	0.71
87303	1.19	5	1.11	5.4228	102.1577	2.3915	1.09156	4.09	5.5	1.8	2	1	2	2	2	0	2	2	40	1	159.6	58.8	23.08403	86.8	0.708
88531	0.69	3	1.5	5.2812	75.4148	2.166608	0.77968	3.83	5.1	16.2	2	1	2	2	2	0	2	2	40	4	165.9	121	43.96353	129.5	1.37
86626	4.96	5	0.8	6.113733	180.9408	2.1226	1.60064	2.97	6	10.9	2	3	2	2	2	0	2	2	41	6	163.8	83.9	31.27045	104.1	0.605
86534	2.2	4	1.6	5.33	86.88	3.569	1.129	5.69	5.3	3	2	3	2	2	2	0	2	2	43	4	155.4	85.9	35.5706	100.8	0.921
87018	3.63	5	1.32	5.76	106.08	2.379	0.926	4.11	5.6	4	2	3	2	2	2	0	2	2	49	2	155.6	70.6	29.15987	84.7	0.847
87154	0.77	2	1.09	5.88	105.9	4.732	2.924	7.16	5.4	0.8	2	2	2	2	2	0	2	2	30	1	155.2	62.5	25.94756	93.2	0.696

87100	1.85	4	0.83	5.601	135.7158	3.6208	1.64508	5.48	5.6	3.3	1	1	1	2	2	0	2	2	23	6	159.8	65.4	25.61086	90.4	0.971
84818	1.76	4	1.34	5.1612	53.586	2.314478	0.64226	4.06	5.3	0.2	1	1	1	2	2	0	2	2	33	3	162.4	59.6	22.59822	78.4	0.566
91477	1.58	4	1.42	5.27	38.82	2.25	0.655	3.98	5.3	14.8	1	3	1	2	2	0	2	2	35	1	152.5	104.4	44.89116	124.2	1.13
85587	3.43	5	2.07	5.4248	68.1564	3.87798	1.076283	6.26	5.5	2.3	1	3	2	2	2	0	2	2	49	3	163.7	83.2	31.04745	97.2	0.817
85229	5	5	1.22	5.53035	110.7508	2.712426	1.22558	4.47	5.2	12.2	1	3	1	2	2	0	2	2	29	3	171.2	122.4	41.76129	127.4	1.08
87226	1.84	2	2.04	5.437	39.6814	2.14566	0.598926	4.22	5.1	0.3	1	1	1	2	2	0	2	2	41	6	155.6	52.8	21.80794	83.2	0.628
93181	4.96	3	1.37	5.5	42	3.388	0.948	5.2	5.2	3.3	1	1	1	2	2	0	2	2	46	1	155.1	72.6	30.17957	90.1	0.728
86532	0.5	2	1.29	5.387	48.12	1.905542	0.574568	3.49	5.3	0.08	2	1	1	2	2	0	2	2	19	4	161.5	69.6	26.68481	80.7	0.513
88890	1.83	1	1.03	5.44	88.2	2.276	1.016	3.78	5.7	10.6	2	1	1	2	2	0	2	2	21	3	165.4	124.8	45.61877	143	1.21
91592	3.79	3	1.81	5.5176	147.7164	2.68782	1.035428	4.47	5.7	24.4	2	1	1	2	2	0	2	2	25	4	159.5	101.9	40.05464	117.1	0.944
91566	1.63	4	1.32	5.4538	104.6556	2.37028	0.92066	4.11	5.5	11.9	2	1	1	2	2	0	2	2	25	3	148.1	101.7	46.3672	116.7	1.44
91025	2.031186	5	1.06	5.27	166.92	3.051	0.768	4.47	5.4	2.3	2	1	1	2	2	0	2	2	26	4	158.2	84.8	33.88308	100.1	0.875
92566	3.37	4	2.02	5.1708	38.5968	1.772069	0.47871	3.75	5	0.5	2	1	1	2	2	0	2	2	27	3	156.5	57.7	23.55847	88.3	0.57
89211	3.81	3	1.09	5.284	97.57656	3.15596	1.446968	4.99	5.3	5.9	2	1	1	2	2	0	2	2	29	3	153.1	74.7	31.86913	98.5	0.826
85293	5	5	0.41	6.003966	182.0868	2.87094	2.24268	4.81	5.5	14.1	2	1	1	2	2	0	2	2	29	6	167.3	98.5	35.19203	115.8	1.03
87258	2.59	4	2.15	5.1132	67.95	2.632	0.85718	4.84	5.1	11.8	2	1	1	2	2	0	2	2	30	2	161.5	68.9	26.41643	90.1	0.636
87539	1.25	2	1.84	5.22	14.28	2.638	0.813	4.84	5.4	10.9	2	1	1	2	2	0	2	2	31	2	155.9	40.3	16.58108	70.9	1.03
86009	2.04	4	1.34	5.3662	53.15894	2.861856	0.641293	4.55	5.6	0.3	2	1	1	2	2	0	2	2	33	3	169	75.6	26.46966	90.3	0.693
84143	0.32	1	1.32	6.11	69	2.457	0.824	4.16	5.4	2.4	2	1	1	2	2	0	2	2	35	7	159.3	73.4	28.92441	100	0.845
85859	1.43	5	1.22	5.287833	90.53316	3.30605	1.23508	5.2	5.2	2.3	2	1	1	2	2	0	2	2	35	3	164.8	130.8	48.16076	142.1	0.947
93375	0.92	2	1.66	5.253	36.8094	2.2504	0.59088	4.06	5.4	0.6	2	1	1	2	2	0	2	2	36	3	154.3	42.7	17.93478	66.5	0.511
85661	2.14	2	1.06	4.94	8.88	2.638	1.942	4.58	5.2	5.3	2	1	1	2	2	0	2	2	36	3	154.7	54.6	22.81456	83.4	1.41
83851	1.86	3	1.01	4.39	74.16096	3.75	0.734	5.09	5.2	2.8	2	1	1	2	2	0	2	2	37	3	155.3	85.1	35.28472	106.5	0.878
90980	0.52	3	1.29	5.602948	104.556	4.13038	1.672966	6.7	5.7	12.6	2	1	1	2	2	0	2	2	38	4	169.7	75.5	26.21702	94	0.931
84349	2.24	4	1.47	5.16	49.26	2.328848	0.66336	4.11	5.3	1.9	2	1	1	2	2	0	2	2	39	3	160.8	88.1	34.07249	104.8	0.996
89575	1.85	4	1.55	5.210981	34.539	2.38473	0.65838	4.11	5.3	1.2	2	2	1	2	2	0	2	2	25	2	164	82.8	30.78525	98.6	0.744
88047	1.49	3	1.53	5.3426	46.7968	2.120309	0.62818	3.8	5.2	0.8	2	2	1	2	2	0	2	2	29	1	163	73.4	27.62618	90.7	0.81
92320	0	4	1.66	5.6286	84.6324	3.005355	1.26984	4.97	4.5	11	2	2	1	2	2	0	2	2	29	3	170.8	70.8	24.26932	94.3	1.33
88262	4.37	5	2.02	5.184992	22.40363	2.39764	0.456825	4.4	5	0.4	2	2	1	2	2	0	2	2	41	3	177.1	62.1	19.79951	82.1	0.604
93093	1.75	4	1.4	5.61	65.6508	3.388	0.903	5.2	5.1	10.2	2	2	1	2	2	0	2	2	45	3	155.3	107.6	44.61381	127.7	1
85252	0.94	4	1.63	5.05236	38.3684	2.19543	0.64018	4.06	5.1	0.9	2	3	1	2	2	0	2	2	22	4	157	83.5	33.87561	106.2	0.49
88060	2.12	4	1.27	5.22	35.64	2.043	0.734	3.65	5	0.5	2	3	1	2	2	0	2	2	23	3	165	53.4	19.61433	73.8	0.9
92788	2.98	3	1.47	5.33	52.92	3.491	1.14	5.48	5.1	1	2	3	1	2	2	0	2	2	31	3	163.6	93.2	34.82165	102.7	0.736
85783	1.57	4	1.63	5.08	48.24	3.75	1.592	6.1	5.1	4.7	2	3	1	2	2	0	2	2	32	2	164.5	61	22.54229	80.7	0.818
93073	1.63	5	1.81	5.11	20.64	1.422	0.553	3.49	5.1	0.3	2	3	1	2	2	0	2	2	33	2	167.5	60.3	21.49254	77.8	0.411
86686	3.09	5	2.4	4.72	42	1.681	0.463	4.29	5.2	0.3	2	3	1	2	2	0	2	2	37	4	165.4	92.7	33.8851	95.1	0.559
87169	5	5	1.66	5.207248	48.98222	2.515295	0.71754	4.5	5.3	6.5	2	3	1	2	2	0	2	2	39	3	156.6	52.5	21.40798	70.7	0.973
86566	2.01	4	2.17	5.11	32.16	2.664	0.418	5.02	4.8	0.6	2	3	1	2	2	0	2	2	40	4	163.4	58.7	21.98538	73.8	0.676
88272	3.51	4	1.58	5.4988	56.00848	3.226556	0.792533	5.15	5.6	3.1	2	3	1	2	2	0	2	2	43	3	167.2	79	28.25885	97.8	0.808
87163	2.06	5	1.4	6.22	138.9	4.293	1.389	6.34	5.6	15.6	2	3	1	2	2	0	2	2	44	3	166.8	120.9	43.45445	134.2	1.24
86736	5	5	2.22	4.88	33.72	2.198	1.073	4.91	5.1	3.3	2	3	1	2	2	0	2	2	45	3	169.5	59	20.53585	82.7	0.583
85118	3.56	4	1.03	5.601866	109.0176	3.05818	1.66862	4.99	5.4	7.1	2	3	1	2	2	0	2	2	45	3	154.7	115.9	48.4287	137	1.08
90190	2.47	3	1.19	5.709056	119.0001	2.1671	0.99676	3.75	5.6	7.2	2	3	1	2	2	0	2	2	46	1	166.2	101	36.56447	114.5	1.03
90157	2.53	3	1.19	5.553848	125.1732	4.42104	1.816146	6.85	5.5	14.3	2	3	1	2	2	0	2	2	46	3	162.5	92.1	34.87811	121	1.41
89536	2.53	3	1.81	5.83	72.12	2.47416	0.69299	4.42	5.3	0.5	2	3	1	2	2	0	2	2	47	3	150.5	60.5	26.71052	85.8	0.619
91582	5	5	2.38	5.2789	38.96041	2.832057	0.62478	5.25	5.3	0.08	2	3	1	2	2	0	2	2	48	3	167.8	62.7	22.26812	85	0.759
91222	5	5	1.32	5.66	40.02	2.069	1.129	3.9	5.4	4.4	2	3	1	2	2	0	2	2	48	3	161.6	78.2	29.94498	94.8	0.978
91108	5	5	1.97	5.6646	53.26314	3.6825	0.939216	6.26	5.5	0.5	2	3	1	2	2	0	2	2	49	3	171.8	68.6	23.24223	94.4	0.623
90344	5	5	1.22	4.66	57.12	2.379	1.152	4.11	5.2	0.4	2	1	1	2	2	0	2	2	18	4	161.6	50.4	19.29958	68	0.329
86329	2.06	4	1.6	4.91	25.98	3.051	0.892	5.07	5.1	0.4	2	3	1	2	2	0	2	2	18	2	154	48.5	20.45033	71.4	0.385
93162	0.74	5	2.2	5.05	31.30788	1.565396	0.460215	3.23	5	0.08	2	3	1	2	2	0	2	2	18	1	155.8	57.6	23.72944	81	0.395
93294	0.12	1	1.42	5.137066	50.1504	1.851809	0.62376	3.44	4.9	0.3	2	1	2	2	2	0	2	2	18	1	163.2	51.6	19.37356	70	0.556
91278	0.35	2	1.27	5.2794	51.15102	2.59486	0.68904	4.32	5.4	0.08	2	1	1	2	2	0	2	2	18	4	157.2	56.1	22.70167	71.5	0.566
90989	0.65	1	0.85	5.5432	93.38333	1.878106	1.2204	3.05	5.2	0.08	2	1	2	2	2	0	2	2	18	2	164.1	61.3	22.76372	74.4	0.606
85505	1.55	3	1.34	5.283	60.13062	1.8221	0.686044	3.36	5.6	2.2	2	3	2	2	2	0	2	2	18	2	160.6	57.4	22.25465	79.3	0.78
86311	0.77	2	1.16	5.66	76.3266	1.991	0.384	3.34	5.6	3.9	2	1	2	2	2	0	2	2	18	1	148.7	82.8	37.44626	104.4	0.949
88918	1.0116	2	1.66	5.66	61.26	3.258	0.813	5.28	5.3	0.3	2	1	1	2	2	0	2	2	18	4	163	63.4	23.8624	76.2	0.97
89552	1.48	4	1.78	4.66	48.6	2.172	0.587	4.22	5.3	0.7	2	3	1	2	2	0	2	2	19	4	170.2	64	22.09331	76.5	0.54
87740	5	5	1.71	4.97	68.94	2.457	0.339	4.32	5.1	0.8	2	3	1	2	2	0	2	2	19	1	158.5	65.9	26.23173	92	0.784
91677	2.1	4	1.09	3.55	29.88	3.026	0.373	4.29	5.3	1.4	2	2	1	2	2	0	2	2	19	7	164.7	75.9	27.98044	89.4	0.839

88944	3.55	4	1.63	4.61	43.5	2.276	0.542	4.16	5.2	8	2	3	1	2	2	0	2	2	20	4	158.4	61.2	24.39164	76.8	0.802
84058	2.2	4	1.94	4.94	21.36	2.353	0.44	4.5	5.1	0.08	2	2	1	2	2	0	2	2	21	3	165.4	63.2	23.10181	78.3	0.59
93556	0.52	3	1.76	5.5748	57.35226	3.191373	0.86976	5.38	5.5	0.4	2	1	1	2	2	0	2	2	21	4	169.7	85.2	29.5853	98.1	0.638
88700	0.25	3	0.88	5.3845	115.6068	3.231975	1.491308	5.09	5	8.3	2	1	1	2	2	0	2	2	21	1	159.2	92.1	36.33904	109.1	1.18
86868	2.6	5	1.86	5.1951	32.99388	1.651646	0.457554	3.54	5.4	0.08	2	3	1	2	2	0	2	2	22	2	163.8	48.6	18.11375	64.2	0.547
90914	1.56	5	1.5	4.55	24.84	2.715	0.576	4.47	5	1.7	2	2	1	2	2	0	2	2	23	3	168.6	51	17.94135	66.7	0.556
86467	1.36	5	1.5	5.13748	56.988	2.947153	1.08226	5.02	4.9	1.7	2	1	1	2	2	0	2	2	23	1	154.3	55.3	23.22701	82.2	0.583
91790	0.865066	2	1.01	5.3624	77.5155	2.009373	0.797795	3.23	5.1	0.6	2	1	2	2	2	0	2	2	23	2	161.9	56.2	21.44088	76.2	0.65
84532	1.49	2	1.55	5.287733	35.43554	1.947442	0.585075	3.72	5.3	0.3	2	1	1	2	2	0	2	2	23	1	154.3	61.9	25.99913	85.1	0.654
92634	5	5	1.22	5.961261	121.737	2.528866	1.0955	4.22	5.8	3	2	3	1	2	2	0	2	2	23	6	166.4	72.1	26.03926	83	0.665
88568	0.61	1	1.16	5.61	62.16	2.715	0.26	4.01	5.2	4.1	2	1	1	2	2	0	2	2	23	1	168.8	95.4	33.48139	97	0.882
84200	2.55	4	2.51	4.83	37.32	4.37	1.344	7.5	4.9	4.6	2	3	1	2	2	0	2	2	24	3	172.6	84.4	28.33093	100.4	0.394
92287	1.49	4	1.45	5.053333	42.25608	1.734199	0.615628	3.62	5	0.08	2	1	1	2	2	0	2	2	24	2	151.1	54.9	24.04603	77.2	0.413
90586	1.28	4	1.86	5.270166	56.6182	2.64606	0.783692	4.78	5.3	8.5	2	3	1	2	2	0	2	2	24	4	160.9	123.8	47.81989	132	0.512
92957	4.76	5	1.22	5.22	72.6	3.207	0.644	4.71	4.9	1.8	2	1	1	2	2	0	2	2	24	4	157.7	81.4	32.73113	81.3	0.742
89868	5	5	2.12	5.463565	43.24498	2.479767	0.57199	4.73	5.3	0.08	2	3	1	2	2	0	2	2	25	3	171.2	62.4	21.29007	76.4	0.413
84671	2.81	5	2.12	5.225821	46.7902	2.798188	0.6939	5.02	4.9	0.6	2	3	1	2	2	0	2	2	25	3	157.7	63.8	25.65413	81.8	0.564
85692	3.09	5	1.42	5.22	38.94	1.758	0.576	3.44	5.7	0.3	2	3	1	2	2	0	2	2	25	1	160.1	44.6	17.40012	65.8	0.643
89270	0.91	1	1.24	4.39	40.56	2.405	3.252	5.15	4.9	4.9	2	1	1	2	2	0	2	2	25	1	154.2	67.1	28.21979	99.5	0.647
91351	1.26	5	1.91	5.33	69.24	2.353	0.802	4.63	5.5	0.08	2	3	1	2	2	0	2	2	25	3	163.7	54.6	20.37489	75.7	0.674
89038	2.23	4	1.63	5.010978	60.99	2.643408	0.60603	4.5	4.8	2.5	2	3	2	2	2	0	2	2	25	3	160.8	69.7	26.95633	84.1	0.818
92226	2.86	5	2.22	5.5	70.62	1.345	1.592	4.29	4.8	5.9	2	3	1	2	2	0	2	2	26	3	183.4	92.4	27.47093	95.8	0.492
92037	3.79	5	2.07	5.5	43.8	1.965	0.316	4.19	5.1	0.8	2	3	1	2	2	0	2	2	26	4	168.7	81.3	28.56672	90.4	0.534
91174	4.07	5	2.09	5.16	32.34	1.526	0.237	3.72	4.7	0.4	2	3	1	2	2	0	2	2	26	2	158.9	47.1	18.65405	72.1	0.621
89950	2.95	5	2.2	4.72	80.46	1.293	0.327	3.65	5.2	0.4	2	3	1	2	2	0	2	2	26	4	171.7	58.7	19.9112	74.2	0.821
90608	5	5	1.97	5.394781	42.403	2.580511	0.631512	4.65	5.3	2.3	2	3	1	2	2	0	2	2	27	3	170.5	65.5	22.53163	83.2	0.688
88992	4.28	5	1.14	5.59195	80.58673	2.372613	1.19538	4.22	5.3	4.8	2	3	1	2	2	0	2	2	27	3	167.3	120.3	42.98073	111.9	1.08
88744	0.8344	2	0.83	5.8614	147.99	2.63512	1.70972	4.42	5.5	13.2	2	1	1	2	2	0	2	2	27	1	153.7	88.1	37.29307	124	1.18
91161	2	4	2.07	5.316698	64.1784	4.02744	1.126173	7.03	4.9	1.2	2	3	2	2	2	0	2	2	28	4	167.9	65.1	23.09296	87.6	0.381
87129	4.12	5	1.47	5.345328	66.02691	2.227142	0.640628	3.96	5.2	1	2	3	2	2	2	0	2	2	28	6	172.4	65.6	22.07137	85.9	0.386
83962	1.65	5	1.5	5.176341	39.5928	2.28962	0.612773	4.14	5.3	0.08	2	3	1	2	2	0	2	2	28	4	166.5	68.4	24.67332	88	0.576
91525	4.03	4	2.02	5.66	38.64	2.948	0.497	5.2	5	0.08	2	3	1	2	2	0	2	2	29	6	157.1	50.9	20.62364	71.8	0.352
91216	5	5	1.09	5.507977	104.8337	3.76286	1.88754	6.03	5.3	2.3	2	3	1	2	2	0	2	2	29	7	155.3	82	33.99938	104.3	0.581
85476	1.58	3	2.28	5.6086	121.0716	2.98422	1.26014	5.25	4.8	9.9	2	1	1	2	2	0	2	2	30	2	163.4	83.1	31.12411	105.9	0.555
93330	2.99	4	1.68	5.94	30.18	3.646	0.497	5.56	5.8	0.08	2	2	1	2	2	0	2	2	30	6	148.9	50.2	22.64198	79.1	0.595
89399	5	5	2.35	5.2983	40.25281	2.992755	0.75192	5.56	5.3	0.5	2	3	1	2	2	0	2	2	30	6	151.2	54.8	23.97049	73.2	0.597
89904	4	1	1.66	4.94	17.04	3.129	0.485	5.02	5	3.7	2	3	1	2	2	0	2	2	30	6	154.5	48	20.10871	71.3	0.644
93499	2.95	5	1.89	5.33	55.92	2.379	0.632	4.55	5.1	0.6	2	3	1	2	2	0	2	2	30	4	157.8	68.1	27.34847	86.2	0.705
89174	3.25	5	1.63	5.211954	44.7033	2.038695	0.681592	3.9	5.4	3.7	2	3	1	2	2	0	2	2	30	6	155.4	53.9	22.31962	78.5	0.861
88677	5	4	1.84	5.2762	61.2864	3.448683	0.932021	5.69	4.9	2.4	2	3	2	2	2	0	2	2	31	1	148.3	79	35.9207	108.2	0.436
90964	5	5	1.91	5.4883	42.0258	3.150193	0.7236	5.53	5	0.08	2	3	1	2	2	0	2	2	31	3	170.1	74.8	25.85193	84.1	0.477
85159	2.23	3	1.71	7.11	475.98	3.378877	1.44814	5.48	5.3	3.2	2	3	1	2	2	0	2	2	31	4	165.5	63.9	23.32947	89.1	0.668
92216	3.63	3	1.63	5.2	68.64	1.552	0.621	3.47	4.9	3	2	3	2	2	2	0	2	2	31	2	159.6	66.9	26.26397	89.9	0.723
93535	0.88	2	1.4	5.485933	70.6008	2.77584	0.77771	4.45	5.6	3.3	2	1	1	2	2	0	2	2	31	2	160.9	123.4	47.66538	132.7	0.765
88257	2.6346	1	1.19	6.044402	125.8596	2.862245	1.23492	4.65	5.7	3.2	2	3	1	2	2	0	2	2	31	2	158.3	69.2	27.61495	94.3	0.894
86352	2.29	5	1.32	5.7687	114.5234	2.420404	0.91989	4.19	6.1	2.5	2	3	1	2	2	0	2	2	31	4	164	100.7	37.44051	120.2	0.952
88653	5	5	1.06	5.936345	154.1119	2.356353	1.30106	4.03	5.6	16.7	2	3	1	2	2	0	2	2	31	1	161.9	130.7	49.8634	132.5	1.36
89959	3.29	5	1.63	4.88	20.34	1.371	0.519	3.23	5.1	0.08	2	3	1	2	2	0	2	2	32	3	166.8	67.2	24.15334	85	0.447
83881	2.06	5	1.86	5.20025	45.1986	2.959075	0.76032	5.12	5.1	0.7	2	3	1	2	2	0	2	2	32	3	162.7	63.2	23.87492	80.3	0.628
83742	1.03	4	0.72	5.8058	140.0904	3.0851	1.762455	4.91	5.6	3	2	1	1	2	2	0	2	2	32	1	151.3	64.5	28.17616	93.3	0.717
85483	1.29	4	0.88	5.5	108.24	2.172	2.348	4.14	5.5	11.9	2	1	1	2	2	0	2	2	32	3	148	67.3	30.72498	90.8	1.2
86649	0.17	3	0.93	5.5994	136.8636	2.5799	1.55986	4.27	5.5	13	2	1	1	2	2	0	2	2	32	1	163.4	144.2	54.00838	132.2	1.25
86922	5	5	2.17	6.33	19.68	2.095	0.485	4.5	5.4	0.2	2	3	1	2	2	0	2	2	33	6	165.5	53.6	19.56901	75.8	0.42
86056	2.11	4	1.37	5.3246	51.858	1.965311	0.64534	3.59	5.1	0.9	2	3	1	2	2	0	2	2	33	3	160.9	74.1	28.6224	86.4	0.549
84768	2.46	3	1.4	5.3902	62.4018	2.756326	0.836241	4.53	5.3	1.5	2	1	1	2	2	0	2	2	33	1	146.1	79.4	37.19804	103.4	0.694
87965	5	5	1.55	5.373331	48.8672	2.29238	0.56304	4.14	5.3	0.08	2	3	1	2	2	0	2	2	34	3	155.9	55.6	22.87613	76	0.4
85437	0.55	4	1.66	5.194393	34.8	1.95912	0.619845	3.85	5.3	0.4	2	1	1	2	2	0	2	2	34	4	166.6	74.9	26.98558	83.7	0.561
91404	4.95	5	1.32	5.16	49.26	3.672	0.994	5.46	5.4	1.1	2	1	1	2	2	0	2	2	34	6	160.6	65.9	25.5502	80.3	0.61
87179	1.26	2	2.51	5.83	151.02	2.483	1.818	5.82	5.7	5.4	2	3	1	2	2	0	2	2	34	2	156.4	96.3	39.36886	104.6	0.673
93677	1.76	5	1.63	4.77	19.26	2.793	0.418	4.6	5.2	0.3	2	2	1	2	2	0	2	2	34	3	167.4	62.2	22.19624	83.3	0.752

92438	3.17	4	1.6	5.296248	36.5932	1.777097	0.577575	3.31	5.3	1.9	2	3	1	2	2	0	2	2	34	2	157.3	44.2	17.86343	69.4	0.776
90153	1.04	2	1.34	5.3797	72.5136	2.62946	0.7953	4.34	5	3.5	2	1	2	2	2	0	2	2	34	1	148.6	68.6	31.06608	94.7	0.795
87090	2.2	3	1.4	5.25	112.26	2.741	1.106	4.65	5	1.6	2	3	1	2	2	0	2	2	34	2	164.9	87.2	32.06824	106.3	0.835
91711	4.12	5	0.98	5.5646	110.2039	2.393396	1.4671	4.22	5.3	3.9	2	3	1	2	2	0	2	2	34	7	148.9	79.7	35.94752	109.2	1.21
86399	2.46	3	1.97	5.38	80.76	2.87	0.44	5.04	5.2	0.5	2	3	1	2	2	0	2	2	35	6	154.1	56.5	23.79267	87.5	0.442
90746	5	5	1.03	5.11	90.9	3.103	1.648	4.89	5.5	3.3	2	1	1	2	2	0	2	2	35	2	154.4	62.1	26.04936	85.6	0.445
87436	1.07	1	1.4	5.38	27.66	2.224	0.44	3.83	5.1	0.08	2	1	1	2	2	0	2	2	36	1	158.3	72	28.73232	95.4	0.434
86456	3.72	4	2.09	5.431483	41.3568	2.915352	0.6782	5.17	5	0.08	2	3	1	2	2	0	2	2	36	2	153.3	47.8	20.33966	74.2	0.454
85076	1.22156	4	1.45	5.42	32.46	1.655	0.711	3.44	5.8	0.5	2	1	1	2	2	0	2	2	36	1	157	54.5	22.11043	76.6	0.516
89026	5	5	2.12	5.5	198	4.681	1.377	7.42	5.3	6.4	2	3	1	2	2	0	2	2	36	7	167.6	71.2	25.34732	100.7	0.55
87800	1.002133	1	0.72	5.9352	99.59453	3.01992	1.649	4.97	5.9	1.3	2	1	1	2	2	0	2	2	36	1	147.5	64.8	29.78454	94.1	0.663
91317	2.37504	4	1.4	5.233283	64.7028	1.893672	0.59111	3.39	5	2	2	3	2	2	2	0	2	2	36	4	176.7	124.5	39.87459	121	0.75
86123	5	5	1.5	5.3628	55.94102	2.021685	0.71224	3.62	5.2	6.1	2	3	1	2	2	0	2	2	36	3	167.7	74.9	26.63273	95.6	0.755
89711	4.54	5	1.78	5.22	25.56	2.612	0.61	4.68	5.1	4.6	2	3	1	2	2	0	2	2	36	3	158.3	86.2	34.39897	108.4	0.871
91054	2.02	1	2.69	5.0488	54.1542	4.419606	1.39548	8.17	5	3.3	2	3	1	2	2	0	2	2	37	6	146.5	61.9	28.84134	98.9	0.307
92663	3.41489	5	1.66	6.33	62.76	2.741	0.881	4.81	5.9	0.08	2	3	1	2	2	0	2	2	37	6	153	57.8	24.69136	80.8	0.447
88384	2	4	1.27	5.33585	77.1096	2.61552	0.78884	4.29	5.3	1.8	2	1	1	2	2	0	2	2	37	3	168	80.9	28.66355	89.3	0.653
91479	0.35	3	1.45	5.69	60.06	2.986185	1.05795	4.86	5.6	1.1	2	1	1	2	2	0	2	2	37	4	161.8	80.8	30.86415	99.2	0.671
85781	2.79	4	0.78	5.553	112.3356	3.148620	1.60164	4.86	4.6	6.8	2	1	1	2	2	0	2	2	37	1	158.7	79.7	31.64495	109.1	0.803
91487	4.33	5	1.76	5.225466	44.8716	2.58761	0.71058	4.71	5.2	1.8	2	3	1	2	2	0	2	2	37	3	178	69.7	21.99849	83.5	0.919
92977	0.02	4	1.42	5.33	48.6	3.129	1.118	5.07	5.3	2.8	2	1	1	2	2	0	2	2	38	1	146.1	43.4	20.33243	69.1	0.713
85194	2.59	3	1.29	5.2444	59.0508	2.533522	0.8051	4.27	5.1	1	2	1	1	2	2	0	2	2	38	2	167.6	66.1	23.53171	89.7	0.731
92625	2.29	4	0.93	5.55	78.9	3.672	1.298	5.2	5.4	2.4	2	1	1	2	2	0	2	2	38	7	172.1	93	31.39939	102.7	0.932
86637	4.23	5	2.46	5.114592	22.35943	2.324258	0.470708	4.45	5.1	0.3	2	2	1	2	2	0	2	2	40	4	172.6	64.2	21.55031	77.8	0.519
84607	2.1251	1	1.58	5.719977	76.626	3.709753	1.25039	5.84	5.8	2.6	2	3	1	2	2	0	2	2	40	1	150.9	65.9	28.94056	94.3	0.633
88829	5	5	1.22	5.4588	62.2068	2.190993	0.952780	3.88	5.1	0.4	2	3	1	2	2	0	2	2	41	6	172.9	60.1	20.10409	78.5	0.5
89743	5	5	1.58	4.8	17.94	3.129	0.745	5.04	5.3	0.5	2	3	1	2	2	0	2	2	41	3	159.5	56.1	22.05167	78.4	0.596
90640	1.86	4	1.34	5.37156	60.99	1.866782	0.581028	3.41	5.1	0.9	2	1	2	2	2	0	2	2	41	3	163.1	86.9	32.6672	111.9	1.15
92670	2.604281	5	1.78	5.166013	37.1934	2.129157	0.58234	4.19	5.5	0.2	2	3	1	2	2	0	2	2	42	6	156.7	50.9	20.72907	71.4	0.392
90856	2.6	5	1.84	5.1588	40.1268	2.063537	0.56479	4.01	5.2	0.7	2	3	1	2	2	0	2	2	42	2	155.7	50	20.62494	69.8	0.595
84617	3.14	5	1.45	5.3391	67.47915	2.038256	0.649494	3.52	5	3.1	2	3	2	2	2	0	2	2	42	3	170.5	88	30.2715	109.7	0.795
89684	2.06	4	1.76	5.2	61.26	2.819	0.632	4.86	4.9	5.6	2	3	1	2	2	0	2	2	42	2	157.5	62.5	25.19526	85.1	0.828
90633	0.89	4	1.01	7.18	159.36	2.948	1.694	4.73	5.6	7.7	2	1	1	2	2	0	2	2	42	1	165.7	135.9	49.49649	117.7	0.866
88497	1.44	4	1.16	5.336966	97.272	3.28166	1.35388	5.12	5.2	8	2	1	1	2	2	0	2	2	42	3	151.4	97	42.3175	101.7	0.936
91788	2.82	5	1.16	5.2723	124.29	2.95837	1.273849	4.78	5.3	7.6	2	1	1	2	2	0	2	2	42	1	161.2	99.7	38.36764	115.9	0.966
88385	2.552	5	1.29	5.1532	45.43242	1.60319	0.586834	2.95	4.9	0.08	2	3	2	2	2	0	2	2	43	6	158.7	53	21.0437	80	0.438
85209	2.74	4	1.66	5.44	38.46	2.948	1.344	5.22	5.3	2.8	2	3	1	2	2	0	2	2	43	3	166.3	70.7	25.56436	88.5	0.63
92420	1.9353	4	1.14	5.35005	86.55744	2.55442	0.922553	4.24	5.3	2.6	2	1	1	2	2	0	2	2	43	2	155.2	58.3	24.20389	78.8	0.719
85337	5	4	2.25	5.289122	43.3436	3.045745	0.6647	5.38	5.4	1.2	2	3	1	2	2	0	2	2	43	4	158.6	98.7	39.23835	118.7	0.871
88339	4.12	4	1.84	5.05	56.04	2.689	0.531	4.76	5.2	5.1	2	3	1	2	2	0	2	2	43	3	160.5	89.3	34.66581	107.6	1
87229	3.71	5	1.58	5.415757	52.69606	3.110873	0.973907	5.28	5.3	0.6	2	3	1	2	2	0	2	2	43	3	170.1	70.9	24.50404	90.1	1.05
90651	5	5	2.82	5.38	39.3	2.896	0.61	6	5.5	0.08	2	3	1	2	2	0	2	2	44	6	152.9	57.6	24.63812	83.9	0.355
88907	1.55	4	1.4	11.51974	126.2359	4.5116	1.9309	7.76	12.4	2.1	2	3	1	2	2	0	2	2	44	1	160.3	57.2	22.2602	81.2	0.397
88138	4.42	5	2.12	4.88	61.08	3.465	0.576	5.84	5.3	1.5	2	3	1	2	2	0	2	2	44	2	158.5	88.6	35.26754	107.1	0.56
91271	1.59	3	1.06	5.488	118.9224	3.77564	1.68082	5.69	5.7	15.2	2	1	1	2	2	0	2	2	44	3	161.8	74.8	28.57226	96.3	0.692
91126	1.86	3	1.55	5.794948	111.4272	4.20344	1.4131	6.7	5.9	7.3	2	3	1	2	2	0	2	2	44	4	154.9	93.9	39.13477	104.8	0.863
83961	3.7	4	1.14	5.3304	107.3834	3.055786	1.40172	4.97	5.3	2.5	2	1	1	2	2	0	2	2	44	3	154.7	73.2	30.58655	97.5	1.05
90121	4.41	5	1.47	5.77	91.68	3.569	1.671	5.82	5.9	10.7	2	3	1	2	2	0	2	2	44	3	163.8	121.3	45.20984	126.8	1.34
91328	5	5	1.4	5.487123	59.581	2.992729	0.935876	4.97	5.4	0.6	2	3	1	2	2	0	2	2	45	7	161.2	65.8	25.32187	93.7	0.556
90323	3.52	3	1.89	5.226125	65.5608	2.456014	0.719	4.4	5	3.9	2	3	2	2	2	0	2	2	45	1	154.1	73.3	30.8673	87.1	0.746
88830	1.374266	1	0.96	5.444985	84.35533	3.039095	1.385473	4.91	5.6	0.3	2	1	1	2	2	0	2	2	45	1	156.3	59.2	24.23281	87.2	0.801
84316	3.27	5	1.24	5.4948	94.33693	2.29996	1.01202	4.03	5.3	12.1	2	1	1	2	2	0	2	2	45	3	164.3	96.1	35.59986	112.2	1.14
87297	4.22	5	2.35	5.05	27.96	1.396	0.361	3.9	5.3	0.3	2	3	1	2	2	0	2	2	46	7	163.5	82.6	30.89901	96.1	0.517
84077	4.78	3	0.98	5.6672	88.698	3.87326	2.27548	6.44	5.7	0.6	2	1	1	2	2	0	2	2	46	2	157.4	76.4	30.83784	105.3	0.607
83854	2.39	5	1.24	5.59	198.42	3.103	0.96	4.78	5.7	8.3	2	1	1	2	2	0	2	2	46	1	162.7	110.2	41.63001	110.2	0.686
86169	3.25	5	1.45	6.2046	87.6048	2.811008	0.98923	4.58	6.3	0.9	2	3	1	2	2	0	2	2	46	6	159.2	59.1	23.31854	85	0.69
89435	1.42	5	1.45	5.194388	55.1644	2.27362	0.61878	4.11	5.2	0.9	2	1	1	2	2	0	2	2	46	3	152.1	66.4	28.70183	88.5	0.719
88460	4.54	3	1.06	5.510514	66.34776	2.267166	0.992768	3.9	5.3	4.6	2	3	1	2	2	0	2	2	46	3	156.9	93	37.77783	105.7	0.81
89622	5	5	1.81	5.5	34.68	3.051	0.723	5.2	5.1	0.5	2	3	1	2	2	0	2	2	46	3	161.9	56.8	21.66979	74.2	0.832
83953	5	5	2.2	5.39058	43.6409	3.160595	0.789693	5.82	5.3	1.1	2	3	1	2	2	0	2	2	46	1	153.8	67.9	28.70497	93	0.908

87311	5	5	2.15	5.05	20.22	1.629	0.689	4.09	4.8	0.5	2	3	1	2	2	0	2	2	47	3	175.3	71.1	23.13693	83.3	0.567
92194	1.92	4	1.97	5.3265	48.31948	2.838435	0.78758	4.99	5.2	2.9	2	3	1	2	2	0	2	2	47	4	161.3	63.7	24.48334	86.3	0.701
91603	0.64	2	1.14	5.66	137.28	4.45197	2.07396	8.48	5	2.6	2	1	1	2	2	0	2	2	47	6	158.9	61.3	24.27799	93	0.966
89496	1.03	5	1.37	5.2624	54.8808	2.57308	0.66932	4.27	5.6	0.3	2	1	1	2	2	0	2	2	48	6	151.2	48.3	21.12728	81.2	0.527
89640	1.02	3	1.68	5.161013	42.2874	2.7529	0.770253	4.73	5.5	1.1	2	1	1	2	2	0	2	2	48	6	159.2	50.2	19.80695	77.2	0.553
88766	5	5	1.42	5.724777	78.472	2.857786	0.968273	4.71	5.7	1.8	2	3	1	2	2	0	2	2	48	6	162.9	95	35.79987	118.1	0.669
90768	3.3	5	0.88	5.802256	156.1034	3.57026	1.97032	5.64	5.7	6.2	2	3	1	2	2	0	2	2	48	2	156.7	80.9	32.94659	99.8	0.854
87250	3.930687	5	2.12	5.44	43.92	2.767	0.824	5.28	5.3	6	2	3	1	2	2	0	2	2	49	7	165.4	67.9	24.81983	92.9	1.02
91397	1.26	5	1.32	5.4568	86.36688	3.9784	1.36534	6.1	5.6	2.4	2	1	2	2	2	0	2	2	49	2	164	86.5	32.16092	96.2	1.25
88858	0.8354	2	1.29	5.66	128.22	2.922	0.519	4.45	5.2	4.6	2	1	1	2	2	0	2	2	18	4	165.6	105.8	38.58025	118.9	0.822
91445	0.54	3	1.6	4.61	28.62	2.302	0.542	4.16	5.5	1.9	2	1	1	2	2	0	2	2	25	4	159.4	62.4	24.55885	83.2	1.01
92827	0.42	3	1.4	5.16	30.9	2.61226	0.797428	4.27	5.2	4.9	2	1	1	2	2	0	2	2	37	4	162	89.6	34.14114	102.4	0.793
88879	0.42	3	2.46	4.39	70.62	4.02318	1.706556	7.78	5.1	4	2	2	1	2	2	0	2	2	32	4	171.9	97.2	32.89384	108.4	0.398
87056	2.29	4	1.66	4.61	39.96	3.026	0.847	5.07	5.1	0.8	2	3	1	2	2	0	2	2	18	4	162.3	52.4	19.89272	66.3	0.333
90433	5	5	1.37	5.05	91.32	2.845	0.79	4.58	5.1	3.2	2	3	1	2	2	0	2	2	18	3	163.8	61.2	22.80991	78.2	0.525
90319	3.96	5	1.76	5.19925	31.15954	1.731369	0.530688	3.75	5	0.2	2	3	1	2	2	0	2	2	18	6	156.8	44.4	18.05888	68.6	0.554
83890	0.95	3	1.78	5.33	58.14	3.795475	1.415927	6.18	5.3	1.6	2	3	1	2	2	0	2	2	18	6	157.4	50.5	20.38365	69.3	0.998
87814	2.18	3	1.29	5.280014	63.2116	2.68786	0.920268	4.42	5.2	3.7	2	1	1	2	2	0	2	2	20	1	150.4	47.7	21.08738	77.7	0.346
86272	3.1892	4	2.95	5.22	17.52	2.767	0.858	6.1	5.5	0.08	2	2	1	2	2	0	2	2	22	4	158.8	42.5	16.85342	69.1	0.964
87850	1.03	4	1.66	5.09925	42.7068	2.949465	0.72882	4.99	5.1	0.7	2	3	1	2	2	0	2	2	28	6	159.4	52.9	20.81992	73.5	0.605
84347	2.78	5	1.27	5.55	51.6	3.388	0.474	4.86	5	0.3	2	3	1	2	2	0	2	2	28	3	163.5	74.3	27.79414	90	0.66
89794	4.058477	5	1.91	4.66	29.28	3.31	1.682	6	5.3	2.3	2	3	1	2	2	0	2	2	29	6	166.7	58.4	21.01559	80	0.338
87797	0.74	5	1.63	5.231613	30.80013	2.3181	0.565115	4.16	5.5	0.08	2	2	1	2	2	0	2	2	30	4	165.5	58.1	21.21193	77.7	0.627
84459	3.22	5	1.81	4.51	56.34	2.457	1.05	4.76	5.2	12.9	2	3	1	2	2	0	2	2	31	6	158.9	62	24.55522	87.3	1.07
85785	1.06	1	1.63	4.83	38.46	3.181	1.05	5.3	5.8	2.4	2	1	1	2	2	0	2	2	32	1	151.9	65.2	28.25739	98.5	0.574
89957	2.66	3	1.16	5.344614	66.6955	2.850386	0.95025	4.53	5.2	0.9	2	1	1	2	2	0	2	2	33	6	144.9	59.9	28.52923	90.2	0.685
92212	4.99	5	1.5	5.27	37.68	3.026	0.689	4.84	5.2	4	2	3	1	2	2	0	2	2	33	6	149.5	71.2	31.85647	91.8	0.73
90618	5	5	1.55	5.5	67.02	2.353	0.531	4.14	4.9	0.6	2	3	2	2	2	0	2	2	34	6	159.5	59	23.1916	89	0.604
89705	1.51	4	2.02	5.5748	70.8108	3.81026	1.186766	6.18	5.5	2.4	2	3	1	2	2	0	2	2	34	4	164.7	85.7	31.5932	107.1	0.797
90703	1.72	4	1.71	5.83	94.56	2.974	1.061	5.17	5	5	2	2	1	2	2	0	2	2	35	6	158.6	50.7	20.15587	80.6	0.518
88476	5	1	1.99	5.565243	49.9245	2.958893	0.70944	5.09	5.2	1	2	3	1	2	2	0	2	2	35	3	160	73.4	28.67188	87.2	0.519
89820	2.82	5	2.15	4.97	35.16	2.25	0.632	4.68	5.1	1.3	2	3	1	2	2	0	2	2	35	1	157.8	67.7	27.18784	91.4	0.596
90036	5	5	1.84	5.22	83.52	3.905	0.779	6.1	5.5	1.8	2	3	1	2	2	0	2	2	36	6	161	81.4	31.40311	101.7	0.565
92861	0.99	4	1.81	5.07921	46.1994	2.576630	0.542603	4.55	5.6	1.3	2	3	1	2	2	0	2	2	36	4	162.1	71.1	27.05849	89.2	0.675
87653	1.23	1	1.34	5.715285	84.936	2.37916	0.800588	4.03	6	0.6	2	1	1	2	2	0	2	2	37	2	154.9	66.9	27.88196	89.6	0.76
87640	1.42	3	1.03	5.03	134.28	2.431	1.761	4.27	5.4	9.3	2	1	1	2	2	0	2	2	37	2	160	75.5	29.49219	105	0.768
88496	1.83	4	1.29	5.27	29.04	2.948	0.677	4.55	5.4	0.9	2	2	1	2	2	0	2	2	37	3	164.8	109.8	40.42853	118	0.772
83885	3.62	4	1.34	4.94	29.28	5.146	0.994	6.96	5.2	0.08	2	2	1	2	2	0	2	2	38	6	149.3	48.7	21.84788	74.5	0.458
84795	5	5	1.53	6.11	54.18	3.931	0.644	5.74	5.4	2	2	3	2	2	2	0	2	2	38	3	163.1	80.5	30.26133	94	0.767
92319	5	4	1.47	5.27	71.04	3.207	0.666	4.99	5.9	0.8	2	1	1	2	2	0	2	2	40	6	156.4	61.4	25.10122	91.8	0.622
85075	0.62	3	1.34	5.83	120.6	3.62	1.298	5.56	6.4	11.9	2	1	1	2	2	0	2	2	41	2	144.5	81.5	39.0321	110.2	0.919
84456	1.76	3	1.78	5.0376	36.1876	2.165422	0.63519	4.19	4.9	1.3	2	3	1	2	2	0	2	2	42	6	150.8	46	20.2281	71.9	0.514
90642	1.48	1	1.42	4.44	41.28	2.741	0.463	4.37	5.6	1.4	2	1	1	2	2	0	2	2	42	4	170.1	56.6	19.56175	74.1	0.591
88699	0.61	1	1.4	5.4106	73.6116	2.120616	0.684528	3.52	5.6	3.8	2	1	2	2	2	0	2	2	42	1	163.9	97.3	36.22054	104.7	0.912
93333	4.99	3	1.37	5.6843	64.8888	3.068232	1.27284	5.07	5.6	0.8	2	3	1	2	2	0	2	2	43	1	156.8	90.1	36.64651	106.8	0.458
92297	2.61	5	1.76	5.227602	35.9754	2.178777	0.601445	4.27	5.3	0.3	2	3	1	2	2	0	2	2	44	1	157.1	62.1	25.16166	82.7	0.368
93459	1.24	1	1.71	5.23516	49.4592	2.7391	0.77988	4.58	5.7	2.1	2	3	1	2	2	0	2	2	44	2	155.5	81.5	33.70519	98.9	0.767
89476	2.1	4	1.63	4.5	67.32	2.767	0.565	4.65	5.9	5.9	2	3	1	2	2	0	2	2	44	4	161.7	106	40.54019	106.8	0.926
90470	2.33	3	1.16	5.9084	128.61	4.18058	1.784526	6.21	6.1	14.4	2	3	1	2	2	0	2	2	44	1	147.3	90.8	41.84854	113.6	1.06
84826	0.95	3	1.81	5.77	42.6	6.181	1.163	8.53	5.8	0.9	2	1	1	2	2	0	2	2	45	6	153	49	20.93212	76.5	0.65
86007	5	5	1.09	5.511977	127.338	3.78825	2.07656	6.05	4.9	2.5	2	3	1	2	2	0	2	2	46	6	156.4	61.4	25.10122	90.7	1.12
92762	5	5	1.09	6.004	168.3528	2.90432	1.66456	4.84	5.8	10.3	2	3	1	2	2	0	2	2	47	6	153.2	73.1	31.14583	91.3	0.964
90298	4.46	4	1.81	5.5172	46.67168	2.977446	0.730693	5.02	5.4	3.3	2	3	1	2	2	0	2	2	48	6	165.3	57.9	21.19009	83.4	0.813
91675	0.65	4	0.88	5.6836	100.212	3.32772	1.49414	5.12	5.7	1.2	2	1	1	2	2	0	2	2	48	2	165.4	87.4	31.94776	104	0.845
88708	0.68	1	1.22	5.549	60.96	2.6507	0.83313	4.29	5.3	0.3	2	1	1	2	2	0	2	2	49	6	150.1	53.1	23.56856	85.3	0.705
91596	1.15	3	2.15	4.39	24.96	2.508	0.768	5.02	4.7	3.4	2	3	1	2	2	0	2	2	42	4	160.2	82.3	32.06822	110.2	0.48
85352	1	3	0.59	5.86465	144.1452	3.78752	2.34297	5.82	5.5	9.9	2	1	1	2	2	0	2	2	38	1	153.2	104.6	44.56708	124.5	1.1
89829	0.49	1	1.63	5.88	95.52	3.2129	1.09438	5.25	5.5	8.4	2	1	1	2	2	0	2	2	39	1	150.3	71.7	31.73958	95.6	1.02
85158	2.6	4	2.33	6.05	31.44	2.302	0.553	4.89	6.1	2.3	2	2	1	2	2	0	2	2	44	6	150.3	62.2	27.5342	84.7	0.498
87913	2.76	2	1.45	4.74	41.28	2.353	0.418	3.98	5.2	0.4	2	3	1	2	2	0	2	2	26	3	168.5	75.5	26.59176	96	0.999

90366	0.93	2	1.06	5.8618	119.9136	2.23514	1.23628	3.72	5.9	2.9	2	1	1	2	2	0	2	2	28	4	164.3	91.4	33.85876	114.6	0.841
88843	1.23	1	2.35	4.27	20.28	3.258	0.644	5.9	5.1	6	2	3	1	2	2	0	2	2	28	1	153.2	59.4	25.30865	90.4	1.32
93240	2.9686	1	1.4	6.55	178.98	2.819	0.926	4.63	5.6	0.6	2	3	1	2	2	0	2	2	31	1	150.5	67.1	29.6244	90.2	0.701
92203	0.21	1	0.96	5.6536	128.7731	2.28296	1.3828	3.88	5.6	8.8	2	1	1	2	2	0	2	2	40	1	156.3	102.3	41.87528	134.2	0.939
85401	0.92	4	1.37	5.66	78.06	2.25	0.463	3.83	5.1	7.4	1	2	2	2	2	0	2	2	46	1	154	125.2	52.79136	135	0.779
90919	0.18	1	2.02	5.26905	55.4817	2.44274	0.627251	4.42	4.7	0.5	2	3	2	2	2	0	2	2	28	1	152.6	59.8	25.67983	86.9	0.585
87147	1.27	2	1.99	5.441413	77.1945	2.5918	0.87788	4.55	5.5	10.2	2	1	2	2	2	0	2	2	20	7	158.9	83.6	33.10995	108.9	0.598
86618	1.24	5	0.7	5.6246	167.4852	3.44302	1.80574	5.43	5.8	2.5	2	1	2	2	2	0	2	2	34	6	155.4	71.3	29.52484	93.7	0.774
91921	0.74	3	1.24	5.6359	81.3192	3.76736	1.50694	5.74	5.6	5.3	2	1	2	2	2	0	2	2	45	6	154.2	82.5	34.69646	103.2	0.518
89025	0	1	1.4	6.27	53.94	3.905	1.027	5.77	6.2	1.3	2	1	2	2	2	0	2	2	48	6	149.2	64.4	28.92999	97.4	0.739
91833	5	5	1.78	5.27	22.98	2.25	0.576	4.29	5.2	1	1	2	1	2	2	0	2	2	27	2	172.2	63	21.24586	74.4	0.82
90744	1.99	4	1.03	5.66	110.52	2.87	0.734	4.24	5.1	2.3	1	3	1	2	2	0	2	2	20	3	172.7	86.4	28.9687	105.8	0.85
93430	0.78	4	1.68	5.2276	55.72476	3.57794	0.98838	5.66	5.4	0.08	1	3	1	2	2	0	2	2	26	3	162.4	57.4	21.76406	78.9	0.587
85431	1.49	2	1.19	7.47	233.52	4.06	1.705	6.03	6.3	7.6	1	3	1	1	2	0	2	2	47	1	173.3	123.8	41.22147	129	0.861
84778	1.88	4	2.17	5.38	43.38	1.371	0.666	3.85	5	0.3	2	1	1	2	2	0	2	2	20	4	173.4	65.1	21.65124	79.9	0.703
92526	0	5	0.93	5.38	208.56	2.974	1.535	4.6	5.5	6.3	2	1	1	2	2	0	2	2	22	6	160.3	88.4	34.40212	111.6	0.935
89562	0.1	2	1.29	5.500052	62.48862	2.885806	0.886973	4.63	5.1	4	2	1	1	2	2	0	2	2	34	4	168.7	111.1	39.03767	113.4	0.959
90528	1.65	4	1.5	5.33338	62.5824	3.00332	1.060971	5.04	5	6	2	1	1	2	2	0	2	2	40	3	157.5	96.3	38.82086	113.5	1.03
86889	0.16	3	0.8	5.38	65.46	4.086	2.529	6.05	5.8	2.7	2	1	1	2	2	0	2	2	47	3	164.8	69.1	25.44273	88.3	1.1
84620	1.55	4	1.27	5.6878	106.194	2.938286	1.094787	4.81	5.3	6.8	2	2	1	2	2	0	2	2	21	6	155.1	60.2	25.02493	87.7	0.955
91597	5	5	1.4	5.525577	86.271	4.03356	1.41344	6.54	4.7	2.5	2	2	2	2	2	0	2	2	23	3	161	64.4	24.84472	82.1	0.373
91938	0.58	2	0.83	5.83885	134.3146	3.48894	2.36725	5.46	5.4	4.3	2	2	1	2	2	0	2	2	32	1	146.2	73.1	34.19973	103.3	0.796
84899	1.86	4	2.33	5.238	29.40994	2.509588	0.56986	4.84	5.1	0.7	2	2	1	2	2	0	2	2	32	3	168	65.1	23.06548	83.9	0.872
92536	1.94	4	1.68	5.332348	71.2986	4.17132	1.373458	6.72	4.7	7.7	2	3	1	2	2	0	2	2	25	2	148.8	48.9	22.08528	77.8	0.646
88704	0.71	4	1.73	4.88	33.06	3.103	1.423	5.48	5.1	0.08	2	3	1	2	2	0	2	2	29	3	169.9	58.7	20.33534	82.2	0.682
87318	0.95	5	1.22	4.83	65.7	1.94	0.316	3.31	5	0.5	2	3	2	2	2	0	2	2	30	4	167.3	82.4	29.43983	97.9	0.567
89589	2.53	5	1.97	5.056449	42.1846	2.443047	0.602866	4.68	5	0.7	2	3	1	2	2	0	2	2	30	7	157.4	52.2	21.06983	73.8	0.631
85656	3.751347	5	2.3	5.341004	40.2195	2.201407	0.621686	4.47	5	0.3	2	3	1	2	2	0	2	2	34	3	158.8	49.7	19.70858	81.2	0.48
91785	3.14	5	2.02	5.181983	32.35308	1.690756	0.421207	3.8	5	0.2	2	3	1	2	2	0	2	2	34	7	161.8	63.7	24.33226	84.5	0.499
90576	2.67	3	1.71	5.55	28.02	2.198	0.79	4.27	5.1	0.3	2	3	1	2	2	0	2	2	34	3	173.5	69.7	23.15442	81.8	0.69
87664	1.49	4	2.17	5.2132	51.93326	2.96013	0.686666	5.17	5.7	1.6	2	3	1	2	2	0	2	2	34	3	171.4	94.2	32.06485	111.9	0.976
91261	1.02	5	1.53	4.61	43.32	1.81	0.926	3.75	5.2	5.5	2	3	1	2	2	0	2	2	44	3	163	84.1	31.65343	102.8	0.805
90668	5	5	1.99	4.94	26.52	2.302	0.745	4.63	5.4	5.5	2	3	1	2	2	0	2	2	18	3	165.7	68.2	24.8393	85.6	0.458
88754	1.05	2	1.4	5.3104	51.7668	2.7303	0.69048	4.47	4.7	0.3	2	1	1	2	2	0	2	2	18	1	171.8	78	26.42703	86.6	0.778
92935	0	2	1.03	5.33	142.08	2.120773	1.13262	2.95	5	0.5	2	1	2	2	2	0	2	2	18	3	173.8	87.7	29.03354	95	1.31
83862	2.45012	4	1.45	5.33	86.82	3.24076	1.168098	5.25	5.5	0.7	2	1	1	2	2	0	2	2	19	2	157.1	60.3	24.43233	78.7	0.732
93562	0.63	2	1.34	5.42	43.26	2.043	0.666	3.7	5.8	2.3	2	1	1	2	2	0	2	2	19	7	166.7	74.9	26.95322	81.6	0.933
88702	1.86	3	1.16	5.459014	80.418	2.396693	0.932181	4.22	5.3	2.8	2	1	1	2	2	0	2	2	20	3	167.6	92.5	32.93015	102.6	0.877
88951	5	4	1.81	5.16	37.56	1.965	0.655	4.09	5.1	0.4	2	3	1	2	2	0	2	2	20	3	166.3	63.5	22.96092	85.9	0.94
93509	0.49	4	1.27	5.38	32.94	2.56	0.7	4.14	5.2	0.3	2	1	1	2	2	0	2	2	21	1	156	50.7	20.83333	69.4	0.444
87120	5	4	1.71	5.38	31.74	2.767	0.429	4.68	5.3	0.08	2	2	1	2	2	0	2	2	21	3	164.9	52.6	19.34392	71	0.475
88903	4.04	5	1.68	4.66	16.86	1.991	1.016	4.14	5.1	2.1	2	3	1	2	2	0	2	2	22	3	167	46.6	16.7091	66.4	0.371
88433	4.54	3	1.66	5.3442	60.1992	2.668285	0.64214	4.5	4.9	0.6	2	3	1	2	2	0	2	2	22	3	162.8	55.7	21.01582	71.1	0.502
90805	5	3	1.91	5.1903	69.5142	3.07006	1.22552	5.28	4.4	5	2	3	1	2	2	0	2	2	22	4	163	108.5	40.83707	118.1	0.881
92985	2.68	4	1.11	5.21	73.2924	2.068047	0.675546	3.44	5.1	3.1	2	3	2	2	2	0	2	2	22	2	166.6	64.2	23.1305	85.7	1.05
89364	5	3	1.34	5.3865	69.85728	2.175506	0.79195	3.59	4.7	1.3	2	3	2	2	2	0	2	2	23	4	167.1	78.7	28.18525	102.3	0.702
90377	2.37115	2	1.76	5.3972	66.8304	1.935683	0.529655	3.47	5.6	1.7	2	3	1	2	2	0	2	2	23	1	160.3	63.3	24.6341	94.7	0.718
89584	1.3	4	1.09	5.239342	66.0399	2.157278	0.86856	3.8	5.2	1.3	2	3	2	2	2	0	2	2	23	1	155.8	66.5	27.39597	89.9	0.737
90250	4.63	5	2.38	5.3564	41.9286	2.98537	0.74512	5.48	5.1	0.6	2	3	1	2	2	0	2	2	23	7	163	57.6	21.6794	72.5	1.08
83761	0	5	1.06	5.27	79.38	4.189	2.269	6.28	5.7	1.7	2	1	2	2	2	0	2	2	24	6	156.4	61.8	25.26475	79.5	0.614
89340	1.98	5	1.4	4.88	33.12	1.603	0.429	3.21	4.9	1.2	2	3	1	2	2	0	2	2	24	1	155.2	62.8	26.07211	90.5	0.815
87369	1.34	3	1.68	4.86	32.4	2.508	0.7	4.5	5.3	1	2	3	1	2	2	0	2	2	24	2	159.5	59.4	23.34883	86	0.98
90093	1.36	4	1.22	6.16	142.308	3.31	0.553	4.78	5.8	24.1	2	1	1	2	2	0	2	2	24	1	158.1	121.8	48.72856	135.8	1.19
86059	1.32	5	1.68	5.16	54.18	3.569	0.813	5.61	4.8	0.6	2	3	1	2	2	0	2	2	25	6	159.8	54	21.14658	74.1	0.532
89802	5	5	2.53	5.1344	43.1024	2.492834	0.61304	4.68	5.3	1.4	2	3	1	2	2	0	2	2	25	6	163.3	56	20.99982	75.5	0.539
85147	0.25	2	1.03	5.292852	80.7996	3.168415	1.35024	5.04	5	1	2	1	1	2	2	0	2	2	25	1	148.3	60.5	27.50889	92.7	1.05
88055	0.42	5	2.15	5.38	29.04	1.733	0.294	4.01	5.1	0.08	2	3	1	2	2	0	2	2	26	6	154.6	47.8	19.99903	71.4	0.506
86090	4.22	3	1.5	5.3534	70.9248	1.967591	0.70066	3.41	4.9	3.7	2	3	2	2	2	0	2	2	26	3	165.9	60.6	22.0181	79.5	0.528
88673	2.11	5	1.19	5.05	29.1	2.25	0.553	3.7	5	0.4	2	3	1	2	2	0	2	2	26	6	172.3	65.2	21.96226	83.5	0.596
89816	0.75	2	1.47	5.77	63.3	3	0.463	4.68	5.3	0.9	2	1	1	2	2	0	2	2	26	1	162.5	62	23.47929	82.1	1.06

92741	3.82	5	2.3	5.11	24.78	2.586	0.519	5.12	5.2	0.08	2	2	1	2	2	0	2	2	27	6	160.3	56.9	22.14345	75.2	0.362
90113	2.8	4	1.14	18.6	40.2	4.888	1.163	6.57	11.9	2.7	2	3	1	2	2	0	2	2	27	4	164.8	105.8	38.95572	115.4	0.714
87474	4.064	5	1.29	5.77	327.66	2.664	1.705	4.73	5.7	2.8	2	3	1	2	2	0	2	2	27	6	159.6	65.4	25.67509	86.8	0.822
87131	1.49	3	1.45	5.278733	60.5226	2.945066	0.949408	4.78	5.1	1.8	2	1	1	2	2	0	2	2	27	2	165.1	84.5	31.00006	107.5	0.838
85706	5	2	2.09	5.3202	91.6872	3.41413	1.265	5.66	4.8	7.5	2	3	1	2	2	0	2	2	28	1	159.8	46.3	18.13124	75.4	0.497
86776	1.86	3	1.16	5.2982	93.85176	3.59816	1.45804	5.66	5	3.6	2	1	1	2	2	0	2	2	28	1	157.5	70.8	28.54119	97.4	0.751
90544	3.14	2	1.29	6.072111	112.9812	2.970406	1.168473	4.91	5.8	4.4	2	1	1	2	2	0	2	2	28	1	163.8	85.5	31.86679	97.2	0.855
84847	2.47	3	1.47	5.61	58.68	2.451262	0.73116	4.27	4.8	0.9	2	3	2	2	2	0	2	2	28	1	163.9	65.1	24.23389	84.5	1.02
91156	3.47	5	1.34	5.755977	77.31	2.973928	0.927268	4.81	5.7	1.9	2	3	1	2	2	0	2	2	29	6	166.7	86.1	30.98361	103.4	0.746
89792	5	4	1.06	5.3432	91.8336	3.140140	1.339926	4.94	5.1	1.1	2	1	1	2	2	0	2	2	30	1	155.8	73.7	30.36215	98.7	0.679
90718	3.73	5	1.29	5.3866	68.063828	2.785048	1.01586	4.65	5.1	4.7	2	1	1	2	2	0	2	2	31	6	158.8	71.6	28.39305	91.2	1.14
87555	1.49	4	1.22	5.33	59.22	2.974	0.96	4.63	5	3.3	2	1	1	2	2	0	2	2	31	3	148.6	91.2	41.30068	112.2	1.14
88110	1.49	4	1.11	5.2404	68.3871	2.212398	0.85974	3.78	4.6	0.5	2	1	2	2	2	0	2	2	32	1	164.3	84.1	31.15451	104.8	0.731
84833	3.13	4	1.5	5.2008	63.8148	2.315270	0.74432	4.11	4.6	11.7	2	3	2	2	2	0	2	2	32	4	170.2	61.2	21.12673	85	1.1
87882	3.4	5	1.37	5.5438	89.34744	1.8888	0.7853	2.9	5.1	8.7	2	3	2	2	2	0	2	2	32	3	163.2	57.7	21.66384	78.5	1.51
88572	2.11	3	0.93	5.54	49.86	1.758	1.231	3.26	5.3	1.6	2	1	1	2	2	0	2	2	34	2	158.7	74.9	29.73911	96.2	0.709
90588	0.57	2	1.45	5.648805	63.0524	2.94504	0.814073	4.76	5.4	1.4	2	1	1	2	2	0	2	2	35	1	152.7	78.8	33.79466	102.8	0.797
90426	0.02	3	1.22	5.83	155.64	2.715	2.416	5.04	5.9	0.9	2	1	1	2	2	0	2	2	35	1	148.4	58.2	26.42745	88.6	0.814
84363	2.64	1	1.19	5.61	88.74	2.767	1.152	4.47	5.5	1.3	2	3	1	2	2	0	2	2	36	1	152.9	69.8	29.85661	86.3	0.667
91438	1.25	1	1.29	5.758938	74.7564	2.786406	1.015281	4.53	5.6	1.7	2	1	1	2	2	0	2	2	38	1	154.7	56.4	23.56668	83.4	0.552
88386	5	4	2.02	5.38	43.449	3.036391	0.63354	5.38	5.8	1.1	2	3	1	2	2	0	2	2	38	6	160.5	54.2	21.04017	73.4	0.811
91928	0.26	3	1.22	5.252633	88.726	2.096446	0.992968	3.47	5.2	6.5	2	1	2	2	2	0	2	2	38	4	163.3	65.2	24.4498	87.7	0.876
83828	0.49	3	1.42	5.61	53.04	2.948	1.14	4.89	5.6	0.6	2	1	1	2	2	0	2	2	39	1	162	71.3	27.16811	94.6	0.753
90125	1.84	5	1.16	5.1306	105.366	3.32573	1.31752	5.2	5.2	2.8	2	1	1	2	2	0	2	2	39	1	162.8	109.8	41.42796	117.6	0.77
88089	2.074283	2	1.68	5.351028	50.10956	2.719222	0.63567	4.71	5.1	0.9	2	3	1	2	2	0	2	2	41	1	157	54.9	22.27271	78	0.659
88392	1.41	1	1.47	5.546952	61.9434	2.888206	0.920333	4.76	5.5	2.1	2	1	1	2	2	0	2	2	42	6	147.5	49.1	22.56823	78	0.492
86259	2.7	5	1.78	5.166813	40.2342	2.256732	0.62974	4.24	5.5	0.5	2	3	1	2	2	0	2	2	43	3	166.8	65.5	23.54232	88.2	0.778
91026	2.56	5	1.14	4.39	60.9	2.419292	0.86877	4.34	4.7	0.08	2	1	1	2	2	0	2	2	45	4	160.5	70.4	27.32893	81	0.403
90061	1.38	3	1.6	5.4278	58.30536	3.422473	0.9904	5.59	5.4	0.7	2	3	1	2	2	0	2	2	45	2	162.1	88.1	33.52817	105.7	0.663
85831	2.18	4	1.5	6.146	83.094	4.1661	1.82263	6.41	5.9	2.4	2	3	2	2	2	0	2	2	46	4	155.4	75.4	31.22262	96	0.701
90281	0.59	5	0.88	10.9596	156.8892	4.162094	2.05982	6.18	9.5	4.3	2	1	1	1	2	0	2	2	46	1	163.7	79.4	29.62942	99.8	0.705
91425	2.07	3	0.85	5.7246	135.0373	2.20798	1.26788	3.78	5.5	6.1	2	3	1	2	2	0	2	2	46	3	157.8	99.1	39.79785	120.5	0.972
92186	5	4	0.96	5.38	48.6	3.258	0.835	4.6	5.2	0.9	2	1	1	2	2	0	2	2	48	3	158.1	67.5	27.00474	88.5	0.628
84437	2.892228	4	1.71	5.61	10.8	3	0.463	4.91	5.7	3.4	2	3	1	2	2	0	2	2	48	4	162.7	80.4	30.37253	99.2	0.932
89809	3.37	4	1.29	5.5922	96.8052	3.65208	1.344515	5.84	5.4	10.3	2	3	1	2	2	0	2	2	48	3	156.3	88.1	36.06268	113.4	1.22
93039	0.98	1	0.93	5.954652	120.326	3.233585	2.42105	5.38	5.6	6.6	2	2	1	2	2	0	2	2	49	1	150.6	69.3	30.55507	105	0.927
90454	1.04	3	0.96	5.09995	68.832	2.237598	0.79578	3.65	5	2.4	2	1	2	2	2	0	2	2	20	3	169.8	92.2	31.97831	105.3	0.797
84034	1.55	4	1.89	5.220413	43.9614	2.4998	0.515	4.4	5.5	0.2	2	1	1	2	2	0	2	2	25	4	150.8	44.6	19.61246	68.8	0.555
85019	0.77	2	1.27	4.83	41.7	3.336	0.474	4.81	5.4	10.6	2	1	1	2	2	0	2	2	36	4	165.3	112.8	41.28225	123.5	1.07
84522	4.444987	5	1.66	5.233509	42.56883	2.866431	0.722385	4.84	5.1	0.5	2	3	1	2	2	0	2	2	19	2	160.3	75.1	29.22624	95.4	0.841
87166	3.22	5	1.47	5.176378	59.27451	1.970799	0.574272	3.62	4.5	1.4	2	3	2	2	2	0	2	2	18	3	149.6	48.3	21.58162	68.8	0.399
87192	2.41	5	1.86	4.9919	35.2524	2.034967	0.443390	3.98	4.7	0.8	2	3	1	2	2	0	2	2	18	4	158.1	81.1	32.4457	92	0.633
93493	2.570490	4	1.11	6.22	195.12	2.276	0.542	3.65	5.3	3.5	2	3	1	2	2	0	2	2	18	1	153.3	88.2	37.53049	107.5	0.748
85382	1.6	4	1.6	5.11645	41.19994	2.3636	0.57302	4.32	4.8	0.08	2	3	1	2	2	0	2	2	18	2	159.3	49.3	19.42743	66.7	0.782
86155	3.16	5	1.81	4.94	50.7	2.767	0.406	4.76	5	0.5	2	3	1	2	2	0	2	2	18	3	168.5	82.3	28.98678	87.2	0.815
87687	1.42	3	1.19	5.25	239.34	2.741	1.253	4.5	5.2	4.4	2	1	1	2	2	0	2	2	18	2	163.8	115.9	43.1972	132	0.988
92732	0.21	3	1.03	5	58.08	2.508	0.632	3.83	5.5	2.6	2	1	1	2	2	0	2	2	18	4	163.9	64.5	24.01053	77.3	1.01
91573	0.21	3	1.22	4.88	71.94	2.767	0.632	4.27	5	0.3	2	1	1	2	2	0	2	2	19	3	155	63.1	26.26431	79	0.622
85621	0.02	2	1.86	5.66	59.16	3.077	0.689	5.25	5.4	0.9	2	3	1	2	2	0	2	2	19	2	146.7	53.3	24.76663	77.7	0.648
85778	0.98	2	1.42	5.277	61.1448	1.944362	0.57344	3.49	5.4	1.1	2	1	2	2	2	0	2	2	19	1	162	56	21.33821	80.2	0.732
92826	2.48	3	1.09	5.5452	109.9764	3.77148	1.766	5.77	5.7	3.4	2	3	1	2	2	0	2	2	19	1	162.6	83.1	31.43113	96.3	0.736
91313	1.28	4	1.11	5.359566	92.9424	3.19576	1.42894	5.02	5.1	3.8	2	1	1	2	2	0	2	2	19	1	154	67.2	28.3353	94.6	0.888
88611	5	5	1.37	5.2432	74.94187	2.542770	0.85858	4.34	5.3	5.2	2	3	1	2	2	0	2	2	19	1	163.9	86.5	32.20017	103	1.1
89749	1.55	1	1.06	5.33	33.9	3.026	1.118	4.6	5.4	0.4	2	1	1	2	2	0	2	2	20	2	146.5	46.4	21.61935	78.6	0.669
83822	1.62	4	2.04	4.44	70.56	3.077	0.474	5.33	5.4	1.1	2	3	1	2	2	0	2	2	20	4	164.8	60.4	22.23937	78.4	0.781
89152	0.59	5	1.16	5.399333	97.9128	3.061815	1.367293	4.94	5.3	2.1	2	1	1	2	2	0	2	2	20	1	169.3	83	28.95771	94.2	1.09
86858	3.92	4	1.27	5.560325	74.628	4.3145	1.582005	7.21	4.9	2.4	2	2	1	2	2	0	2	2	20	4	162.1	68.3	25.9929	84.7	1.31
84535	2.89	3	1.19	4.77	59.94	1.81	0.7	3.31	4.7	1.7	2	3	2	2	2	0	2	2	21	3	161.5	79.2	30.36548	107	0.647
86031	0.78	4	1.34	4.94	49.68	1.267	0.305	2.74	5.2	0.3	2	1	1	2	2	0	2	2	22	1	155.8	64.8	26.69563	91	0.757
85492	1.58	4	1.22	5.3934	68.42892	2.151538	0.82384	3.83	5.4	0.7	2	3	1	2	2	0	2	2	22	3	164.5	71.9	26.57034	90.2	0.911

90268	0.21	3	1.03	5.32055	71.0451	2.191986	0.74622	3.39	5.4	3.3	2	1	2	2	2	0	2	2	22	1	157.9	80	32.08675	104.1	1.12
86308	0.2	2	1.24	5.66	127.26	2.457	1.694	4.47	5.7	11.2	2	1	1	2	2	0	2	2	22	2	146.4	66.7	31.12029	102.3	1.35
87790	2.66	4	2.46	5.1456	41.07384	3.04114	0.87142	5.38	5.2	0.08	2	3	1	2	2	0	2	2	24	6	161.3	54.1	20.79354	72.4	0.488
91074	5	4	1.5	5.5	40.62	2.845	0.531	4.58	5.1	3.8	2	3	1	2	2	0	2	2	24	6	149.4	53.4	23.92435	81.8	0.565
85063	3.67	3	0.93	5.68665	71.7986	1.87478	0.945852	3.13	5.3	0.8	2	3	2	2	2	0	2	2	26	4	153.5	51.3	21.77211	75.5	0.537
84402	0.08	4	1.42	5.604	73.215	3.747293	1.4528	6.1	5.3	1	2	2	2	2	2	0	2	2	27	2	170.6	56.9	19.55034	75.8	0.417
87720	4.39	3	1.53	5.42	64.32	2.095	0.61	3.9	5.6	32.1	2	3	1	2	2	0	2	2	27	4	157.1	71	28.76776	84	1.39
85823	1.86	2	1.97	5.7143	61.20236	3.29734	0.86048	5.51	5.7	0.6	2	1	1	2	2	0	2	2	28	1	156.7	63.9	26.02333	92.5	0.528
83871	0.87	1	1.84	5.169028	41.2248	2.39622	0.69662	4.32	5	1.3	2	3	1	2	2	0	2	2	30	1	139.9	53.7	27.43714	82.4	0.559
92485	0.78	2	1.45	4.72	22.92	1.94	0.61	3.67	5.2	3.1	2	1	1	2	2	0	2	2	30	1	150.6	57.7	25.44051	82.1	0.801
89359	0.86	4	1.27	5.66	107.46	1.81	0.903	3.49	5.5	1.1	2	1	2	2	2	0	2	2	30	3	143.7	61.5	29.78253	102	0.856
87600	2.53	4	1.19	5.4262	86.07677	3.000981	1.057846	4.63	5.2	1.7	2	1	1	2	2	0	2	2	30	3	165.7	102.1	37.18611	107.2	0.877
87918	0.84	4	1.32	5.7416	98.4336	3.05432	1.276073	4.97	5.3	9.8	2	1	1	2	2	0	2	2	33	2	157.3	69.3	28.00759	102.8	1.24
87772	0.48	2	1.4	5.54	50.64	2.974	0.903	4.78	5.4	5	2	1	1	2	2	0	2	2	36	1	155.5	60.6	25.06178	95.2	0.895
92179	1.03	5	1.24	5.360966	112.6872	2.970795	1.292113	4.78	5.3	6.4	2	1	1	2	2	0	2	2	37	6	152.2	50.1	21.6276	75.1	1.06
89072	1.25	1	2.02	5.3502	46.0248	3.009213	0.7029	5.22	5.7	0.5	2	3	1	2	2	0	2	2	39	1	155.5	52.2	21.58787	83.3	0.464
88485	0.983133	1	0.75	15.8	72	1.888	0.745	2.97	10.2	22.5	2	1	1	2	2	0	2	2	40	1	152.5	80	34.39936	106.3	1.01
85722	0.69	1	1.06	5.94	64.08	1.5	0.689	2.87	5.7	3.3	2	1	1	2	2	0	2	2	41	1	146.8	69.8	32.38943	106.5	0.646
88058	1.3	3	0.83	5.653466	93.07973	2.2001	1.32202	3.96	5.8	0.7	2	1	1	2	2	0	2	2	41	1	156	65.6	26.95595	91.1	0.693
85073	0.59	1	0.91	5.77	121.4972	2.50532	1.42118	4.22	5.6	5.3	2	1	1	2	2	0	2	2	41	1	170.1	94.2	32.55684	104	0.961
83875	1.82	5	1.71	5.33576	52.0986	1.702993	0.5196	3.03	5.8	1.6	2	3	1	2	2	0	2	2	42	4	163.1	91.6	34.43401	99.4	0.729
87283	2.46	2	1.42	5.453133	59.0856	2.369502	0.74447	4.11	5.4	1.4	2	1	1	2	2	0	2	2	42	4	163.6	81	30.26345	96.7	0.731
89892	0.6	1	0.85	6.017	128.4968	2.754246	1.32988	4.45	5.5	20.4	2	1	1	2	2	0	2	2	42	1	156.3	73.2	29.96354	101.7	0.875
89284	0.83	2	1.09	5.55	156.42	3.178755	1.81928	5.33	5.7	9.4	2	1	1	2	2	0	2	2	42	1	163.7	92.5	34.5179	101.9	1.02
90721	2.97	4	1.22	5.22	91.2	1.474	1.377	3.31	5.4	0.08	2	1	2	2	2	0	2	2	43	6	154.9	69.9	29.13227	93.3	0.732
87276	1.23	3	1.63	5.289778	48.6504	3.21758	0.965026	5.46	5	3.8	2	1	1	2	2	0	2	2	44	1	155.8	71	29.24984	99.2	0.818
92668	1.31	5	1.24	4.94	25.74	3.698	0.632	5.22	5.5	0.6	2	2	1	2	2	0	2	2	45	4	162	64.9	24.72946	83	0.592
87776	0.853466	1	1.42	5.1768	56.4504	2.029978	0.66518	3.62	5.6	2.2	2	1	1	2	2	0	2	2	48	2	159.5	82.1	32.2717	105.7	0.978
88721	1.76	5	1.32	7.99	101.1	2.069	0.655	3.7	7.1	4.3	2	3	1	1	3	0	2	2	42	4	153.9	91.9	38.80058	120.1	0.725
85420	2.55	4	0.88	6.687	152.1043	4.07402	2.13154	6.26	6.4	6	2	3	2	1	3	0	2	2	21	6	160.1	84.1	32.81054	98	0.772
84774	0.863741	3	1.01	6.05	144.18	3	1.468	4.68	6	6.3	2	1	1	2	3	0	2	2	48	4	170.4	100.5	34.61201	109.6	0.88
90307	5	4	0.98	6.26932	209.1507	2.2775	1.3557	3.9	5.9	31.1	2	3	1	2	3	0	2	2	42	4	157	108.5	44.01801	137.7	1.25
83971	2.27	4	1.53	5.5612	72.0828	3.08824	1.060801	4.91	5.1	12.9	1	1	1	2	3	0	2	2	26	3	167.5	109.2	38.92181	112.4	0.647
93568	0.76	2	1.6	5.587777	67.3224	3.96998	1.190243	6.23	5.4	2.9	1	3	2	2	3	0	2	2	46	3	152.4	85.5	36.81257	116.5	0.559
84913	2.24	2	1.45	6.94	113.88	1.991	1.14	3.96	6.9	12.4	2	3	1	2	3	0	2	2	47	1	165.3	101.8	37.2565	120.8	1.06
88302	2.79	4	1.78	7.608866	187.7388	2.66404	1.40968	4.84	5.9	0.3	2	3	1	1	1	1	2	2	44	4	162.2	80.3	30.52206	98	0.628
90282	0	1	0.8	11.6	213.66	4.448	1.942	6.13	9.9	5.3	2	1	1	1	1	1	2	2	49	1	157.1	78.1	31.64453	105.4	0.872
88000	0.31	2	1.01	8.580290	175.8444	2.73238	1.49179	4.34	6.8	49.1	1	1	1	2	1	1	2	2	43	1	160.6	125.7	48.73536	139.4	1.1
87026	0.95	2	1.58	8.102090	164.0436	2.56642	1.187808	4.32	5.4	1.6	2	1	1	2	1	1	2	2	21	1	165	91.3	33.53535	119.2	0.607
91583	2.47	4	1.53	8.6239	206.4488	3.3205	1.26087	5.43	7.4	22.7	2	3	1	2	1	1	2	2	46	6	159.2	78.9	31.13084	111.5	1.28
91622	0.74	2	0.91	6.3648	133.7808	2.794886	1.74212	4.37	5.5	8	2	1	1	1	2	1	2	2	34	1	164.9	106.9	39.31302	119.7	1.09
93075	3.49	5	1.32	5.847	92.5536	4.13864	1.63144	7.42	5.5	0.7	2	3	1	1	2	1	2	2	49	6	160.5	59.4	23.05878	82.4	0.754
88366	1.22	1	1.03	6.2858	93.9132	3.29566	1.63394	5.15	5.5	1.7	2	1	2	1	2	1	2	2	42	1	155.9	69.1	28.43059	83.2	0.703
91485	5	4	1.73	5.854	65.7396	4.16492	1.461818	6.85	5.2	3.1	2	3	1	1	2	1	2	2	24	7	160.7	68.5	26.52521	89.7	1.08
90931	2.53	5	1.16	5.27	236.28	2.793	2.348	5.04	5.1	1.1	1	1	1	2	2	1	2	2	35	1	164.5	102.3	37.80453	106.1	0.874
84734	0.44	3	0.67	7.350466	179.0904	2.3445	1.8484	3.75	6.8	21.5	1	1	1	2	2	1	2	2	36	3	167.6	124.2	44.2154	135.8	1.39
88081	2.09	5	0.65	5.8878	178.8464	3.012175	2.33788	5.17	6	10.4	1	3	1	2	2	1	2	2	36	4	154.6	77.6	32.46704	105.6	1.12
90184	0.89	4	1.55	5.223	53.136	2.84608	0.7442	4.6	5.6	1.9	2	1	1	2	2	1	2	2	36	4	165.6	85.2	31.0684	109.7	0.891
92349	5	3	2.43	5.1616	58.5954	3.282306	1.14398	5.74	5.1	3.7	2	1	1	2	2	1	2	2	41	3	163	92.1	34.66446	113.3	0.851
91807	2.11	5	0.98	5.55	178.74	2.715	3.692	5.38	5.5	10.5	2	2	1	2	2	1	2	2	25	7	167.1	77.7	27.82711	94.8	1.04
88480	1.15	4	1.24	5.9932	124.5408	2.0647	0.85082	3.75	5.9	7.3	2	3	1	2	2	1	2	2	22	7	171	162.1	55.43586	152.2	0.98
90720	4.78	4	1.11	5.557777	85.4712	3.67066	1.536133	5.77	5.5	2.1	2	3	1	2	2	1	2	2	44	2	157.8	78	31.32424	107.1	0.818
91804	1.58	4	1.01	5.9996	138.8796	3.12829	1.62696	5.12	6	7.1	2	1	1	2	2	1	2	2	45	3	170.9	100.8	34.5125	104	0.998
89189	3.45	4	1.16	5.592466	77.34772	2.197546	0.94942	3.72	5.5	5.4	2	3	1	2	2	1	2	2	48	4	155.9	83.6	34.39648	104.4	0.813
84383	5	4	1.32	5.11	31.92	2.767	0.565	4.34	5	2.8	2	2	2	2	2	1	2	2	32	7	154.2	68.4	28.76652	87.5	0.79
91453	0.84396	1	0.8	5.915	94.3064	2.175069	1.30436	3.57	5.5	0.4	2	1	2	2	2	1	2	2	43	2	146.8	51.5	23.89765	82.5	0.793
85111	3.33	5	1.5	7.07	134.58	4.448	1.118	6.47	6.7	18.2	2	3	2	2	2	1	2	2	49	2	157.3	77.3	31.24079	104.8	0.98
88680	1.92	4	2.28	5.2297	37.6356	2.004666	0.507506	4.22	5.1	0.4	1	1	1	2	2	1	2	2	38	4	166.5	56.1	20.23645	80.3	0.706
85244	1.49	1	0.96	5.628	121.6208	3.20156	1.8096	5.33	5.5	16.2	1	1	1	2	2	1	2	2	43	2	152.9	73	31.22539	99.6	1.28
91753	0.72	4	1.32	5.33	50.28	5.043	2.066	7.32	5.2	0.3	1	1	1	2	2	1	2	2	48	3	162.4	55.4	21.00573	80.8	0.716

88900	3.7	4	1.89	5.8066	71.2632	3.09656	0.992803	5.25	5.6	4.4	1	3	1	2	2	1	2	2	49	2	161.9	114	43.49218	132.4	0.887
90297	0.63	4	1.45	5.233333	37.4484	1.881269	0.5618	3.59	5.4	0.4	2	1	1	2	2	1	2	2	21	4	158.8	57.1	22.64306	71.5	0.729
84780	1.25	4	2.17	5.2609	87.9738	3.35332	1.228545	5.92	4.6	12.6	2	1	1	2	2	1	2	2	29	2	146.1	74.4	34.8556	113.6	0.617
86607	3.4	5	1.11	5.44	87.78	2.948	0.903	4.47	5.5	4.5	2	1	1	2	2	1	2	2	30	4	169.8	103.6	35.93225	115	0.813
91555	2.87	5	2.33	5.14	49.44	2.741	1.468	5.74	5.1	5.3	2	1	1	2	2	1	2	2	35	2	164.7	70.6	26.0266	89.5	0.488
89685	2.84	2	1.4	5.304368	75.4676	3.30605	1.20258	5.35	5.4	2.7	2	1	1	2	2	1	2	2	41	1	158.9	85.9	34.02087	103.4	0.9
92140	1.46	4	1.06	5.491366	84.9864	3.80712	2.05285	6.23	4.9	3.5	2	1	1	2	2	1	2	2	48	3	159.9	70.6	27.61263	94.3	1.14
91527	1	4	1.27	4.72	29.46	2.431	0.7	4.01	5	1.2	2	2	1	2	2	1	2	2	41	3	163.6	68.5	25.59316	88.4	0.598
89183	0.77	4	1.09	5.16	113.64	1.914	0.519	3.23	5.6	2.7	2	3	2	2	2	1	2	2	24	1	149.4	107.5	48.16231	133.6	0.959
86472	4.38	5	2.12	5.205366	55.9272	2.51573	0.8789	4.73	5.1	11.3	2	3	1	2	2	1	2	2	30	3	160	71.1	27.77344	102.8	0.664
86552	1.06	3	2.22	5.33	33.12	3.103	0.655	5.64	5	0.8	2	3	1	2	2	1	2	2	37	3	168.1	65.3	23.10882	88	0.873
87742	1.56	4	1.22	5.6136	71.6604	4.077173	1.6199	6.34	5.6	0.8	2	3	1	2	2	1	2	2	41	2	160.3	63.8	24.82868	92.2	0.979
86364	4.95	3	1.37	5.612	98.2356	2.790221	1.11794	4.68	5.4	7.8	2	3	1	2	2	1	2	2	42	3	158.8	74.5	29.54305	89.7	0.764
91838	5	5	2.17	5.315066	45.32988	2.300348	0.540151	4.47	5.1	0.6	2	3	1	2	2	1	2	2	47	3	159.1	57.2	22.59725	82.5	0.488
88703	3.55	4	1.97	5.27	136.26	2.612	1.095	5.07	5.4	1.5	2	3	1	2	2	1	2	2	47	4	159.4	70.5	27.74677	95.5	0.79
92975	2.47	2	1.47	5.506613	49.578	1.989998	0.638135	3.49	5.5	3.5	2	1	1	2	2	1	2	2	18	4	165.6	61.5	22.42614	79.4	1.03
89273	2.24	4	1.24	5.203233	52.16996	1.823682	0.628243	3.28	5	0.4	2	1	2	2	2	1	2	2	19	2	167.9	67.6	23.97979	81.8	0.584
87523	4.22	4	1.22	5.423457	81.30396	3.353906	1.2728	5.22	5.4	2.2	2	1	1	2	2	1	2	2	19	6	161.1	88.1	33.9457	109.3	0.661
92717	1.23	3	1.34	5.16	61.98	1.81	0.576	3.41	5.6	2.1	2	1	2	2	2	1	2	2	22	4	167.3	86.2	30.79749	101.7	0.743
86629	1.52	5	1.86	5.01396	27.54370	1.661086	0.44219	3.72	5.1	0.3	2	3	1	2	2	1	2	2	23	3	165.8	69.1	25.13674	84.3	0.602
84618	2.85	4	1.45	5.55	99.84	3.103	0.666	4.86	4.9	0.5	2	3	1	2	2	1	2	2	23	1	153.6	78.5	33.27264	106.7	0.667
88332	0	3	1.58	5.4669	97.4382	3.19602	1.46972	5.38	5.3	7.1	2	2	1	2	2	1	2	2	23	6	158.3	83.7	33.40132	96.9	0.754
90925	2.705093	3	1.06	5.4938	93.408	4.01662	2.0099	6.28	5.4	8.6	2	1	1	2	2	1	2	2	23	2	158.4	91.6	36.50775	111	1.39
91133	0.4	5	1.66	5.22	60.42	2.87	1.061	5.02	5.1	0.08	2	3	1	2	2	1	2	2	24	6	162.6	52.2	19.74374	76.5	0.566
91869	1.49	4	1.91	5.23556	56.5548	2.505130	0.54536	4.42	5.4	1.8	2	3	1	2	2	1	2	2	25	1	160.9	47.4	18.30907	64.9	0.689
86833	2.81	5	1.71	5.165566	62.496	2.624998	0.88878	4.55	5.1	14.1	2	1	1	2	2	1	2	2	26	3	166.7	75.1	27.02519	83.2	0.541
92338	0.68	5	1.78	5.94	48.24	2.483	0.779	4.63	5.2	0.5	2	3	1	2	2	1	2	2	27	6	162.4	66.7	25.29029	89.8	0.512
93169	5	5	1.94	5.4839	57.33891	2.014520	0.52133	3.88	4.9	0.7	2	3	2	2	2	1	2	2	27	2	162.8	70.1	26.449	85.6	0.626
88867	2.88	5	2.38	4.77	63.84	5.844	1.987	9.13	4.7	9.1	2	3	1	2	2	1	2	2	28	3	172.8	83.3	27.897	101	0.481
84137	2.1	5	2.04	5.05	24	3.543	0.7	5.9	5.1	1.3	2	3	1	2	2	1	2	2	28	6	161.6	50.8	19.45275	72.7	0.789
90262	3.17	4	2.3	5.2913	52.83203	3.14904	0.749903	5.59	5.1	1.5	2	3	1	2	2	1	2	2	30	2	152.7	59.7	25.60332	86	0.737
91497	5	5	1.63	5.466857	69.80736	3.092386	0.911342	5.35	5.4	0.3	2	3	1	2	2	1	2	2	31	1	168.2	63.3	22.37442	85.2	0.571
89730	0.33	4	1.09	5.9868	128.514	2.9761	1.49074	4.91	5.9	4.4	2	1	1	2	2	1	2	2	32	4	163.9	95	35.36435	112	0.605
89911	5	5	1.58	5.5462	92.4816	2.82425	0.9623	4.81	5.1	20.2	2	3	1	2	2	1	2	2	32	2	159.7	96.6	37.87628	106.4	0.738
88920	5	5	1.63	5.6408	58.3428	3.56662	0.882842	5.56	5.6	1.3	2	3	1	2	2	1	2	2	33	6	160.5	70.5	27.36775	97.9	0.696
88533	3.95	4	2.2	5.66	34.2	1.758	0.61	4.24	5.1	0.08	2	3	1	2	2	1	2	2	34	6	158.5	50.9	20.26092	80	0.379
91839	1.4	5	1.76	4.88	32.94	2.095	0.282	3.98	5	1.2	2	3	1	2	2	1	2	2	35	4	167	76	27.25089	92.1	0.926
88529	0.61	3	1.99	6.16	134.94	3.491	0.666	5.79	6.1	14.2	2	1	1	2	2	1	2	2	35	2	162.9	114.8	43.26132	135.4	1.05
87447	4.86	5	1.99	5.55	27.18	2.948	0.813	5.3	5.7	0.08	2	2	1	2	2	1	2	2	37	2	153	42.3	18.06997	65.5	0.326
84946	0.34	1	1.22	5.71	55.2	2.405	0.813	3.98	5.4	0.7	2	1	1	2	2	1	2	2	38	2	152.6	58.9	25.29334	85.1	0.913
89886	4.49	5	1.14	5.885185	122.298	3.81036	1.69642	5.69	5.7	14.6	2	3	1	2	2	1	2	2	38	3	160	101.7	39.72656	118.8	1.31
91290	0.91	5	1.34	4.88	69.9	2.043	1.705	4.16	5	0.7	2	1	1	2	2	1	2	2	39	6	153.5	63.4	26.90745	87.3	0.56
85231	0.92	1	1.22	5	49.56	2.353	0.892	3.98	5.7	1.9	2	1	1	2	2	1	2	2	40	2	152.7	55.3	23.71631	83	0.718
93277	1.94	2	1.47	4.88	58.14	3.258	1.152	5.25	5.6	7.6	2	1	1	2	2	1	2	2	40	1	150	81.4	36.17778	103.6	0.778
89525	5	5	2.69	5.2893	37.06038	3.015006	0.64832	5.48	5.2	0.08	2	3	1	2	2	1	2	2	41	6	160	46.4	18.125	70.3	0.328
84016	5	5	1.86	6.0994	61.8636	2.9245	0.8797	5.09	6.3	1.4	2	3	1	2	2	1	2	2	41	1	169	68.9	24.12381	88.4	0.63
86181	3.38	5	1.89	5.33385	35.5224	2.110507	0.526696	4.24	5.1	0.08	2	3	1	2	2	1	2	2	42	3	152.2	49.5	21.36859	83.5	0.366
90028	0.7	1	1.89	5.83	55.92	3.957	0.971	6.28	5.4	2.7	2	1	1	2	2	1	2	2	43	1	158.8	72.1	28.59132	101.8	0.836
84229	0.75	1	1.84	5.55	83.1	4.655	0.689	6.8	6	1.1	2	3	1	2	2	1	2	2	43	4	159.6	82.8	32.50608	120.2	0.924
89041	2.76	3	0.98	5.31	109.32	4.138	1.942	6	5.7	8.4	2	1	1	2	2	1	2	2	48	3	175.9	78.2	25.27406	101.1	1.43
93228	3.83	5	1.29	5.44	47.4	2.302	0.553	3.85	4.7	0.08	2	3	1	2	2	1	2	2	18	2	156.3	50.5	20.67157	67	0.684
92433	1.38	1	1.19	5.3422	70.62312	2.034713	0.89193	3.44	5.8	0.8	2	1	1	2	2	1	2	2	18	1	170	84.3	29.16955	100	1.04
93115	0.63	2	1.89	5.55	103.2	1.836	1.084	4.22	5.2	3	2	1	1	2	2	1	2	2	28	1	161.1	76	29.28347	94.1	0.444
91080	5	5	1.34	5.3206	48.93754	2.615795	0.732986	4.53	5.1	0.3	2	3	1	2	2	1	2	2	29	4	163.1	86.8	32.6296	99.9	0.556
86728	1.06	4	1.91	5.06275	40.5024	2.75254	0.62526	4.99	4.9	0.08	2	3	1	2	2	1	2	2	32	2	159.5	56.3	22.13029	79.5	0.538
93352	1.84	5	1.37	5.17	57.6278	2.8249	0.77335	4.63	5.4	0.7	2	1	1	2	2	1	2	2	40	6	154.5	55.1	23.08313	84.6	0.773
91363	1.14	3	0.75	5.8042	88.33824	2.67702	1.36748	4.27	5.4	1.8	2	1	1	2	2	1	2	2	40	1	144.9	60.8	28.95788	88.8	1.05
87219	3.67	3	0.85	5.605533	86.6016	3.28164	1.76738	5.38	5.4	0.5	2	1	1	2	2	1	2	2	42	4	154	59.9	25.25721	90.9	0.728
89175	4.52	1	1.19	5.27	65.16	3.207	1.739	5.2	5.3	4.1	2	1	1	2	2	1	2	2	44	1	144.7	65.4	31.23494	92	0.83
90185	5	5	1.42	6.16	141.72	2.043	1.366	4.09	5.8	4.6	2	3	1	2	2	1	2	2	46	6	155.1	77.7	32.29962	94	0.881

90011	1.461	2	1.78	5.438	50.6536	2.612429	0.6985	4.65	5.2	0.5	2	3	1	2	2	1	2	2	48	6	158.8	87	34.49993	99.9	0.544
86409	1.47	3	1.06	5.5694	106.1016	2.66606	1.22276	4.37	5.5	2.7	2	1	1	2	2	1	2	2	21	4	173.2	129.4	43.13586	121.8	0.613
88216	0.05	2	1.34	5.4278	93.5008	2.028992	0.8691	3.39	5.4	7	1	1	2	2	2	1	2	2	28	4	150.7	90	39.62926	104.4	1.1
85649	0.6	4	1.42	5.3494	45.0888	2.37858	0.61951	4.14	5.6	0.4	1	1	1	2	2	1	2	2	43	3	159	50.4	19.93592	73.2	0.63
88799	1.03	4	1.94	5.44	29.76	1.888	0.7	4.14	5.2	0.08	1	3	1	2	2	1	2	2	21	3	151.9	40.7	17.6392	65	0.463
93429	1	3	1.55	5.159013	45.3652	2.4804	0.6047	4.27	5.5	0.9	1	1	1	2	2	1	2	2	26	1	166.5	51.9	18.72142	77.3	0.8
83865	2.14	2	1.6	5.3736	70.59414	2.57508	0.6761	4.4	5	0.5	2	1	2	2	2	1	2	2	21	3	156.3	39.7	16.25072	65.5	0.602
92624	0.56	1	1.29	5.16	53.16	0.828	1.005	2.59	5.1	2.6	2	1	2	2	2	1	2	2	23	4	170.6	84.1	28.89601	89.4	0.986
92361	4.46	3	2.04	5.4206	39.07937	2.34502	0.53496	4.34	5.6	0.08	2	1	1	2	2	1	2	2	29	7	161.6	50.5	19.33787	73.8	0.554
87769	1.36	3	1.29	6.05	157.5	2.457	1.242	4.32	5.7	0.7	2	1	1	2	2	1	2	2	37	7	156.4	92.1	37.65183	101.2	0.735
89970	0.82	2	1.16	6.0558	130.3764	3.1901	1.3948	5.28	6.2	10	2	1	1	2	2	1	2	2	47	4	157.4	72.6	29.30402	105.8	1.3
84417	4.22	5	1.68	5.452	51.9756	2.633147	0.61094	4.6	5.6	1.5	2	3	1	2	2	1	2	2	18	6	168.8	80.3	28.18193	91.1	0.754
83836	2.76	4	1.66	5.83	111.18	3.905	1.208	6.1	5.5	1.3	2	3	1	2	2	1	2	2	18	1	156.5	68.6	28.00886	89.3	0.804
88027	2.27	3	1.27	5.525	92.3244	2.08122	1.02398	3.78	5.6	3	2	3	1	2	2	1	2	2	20	1	160.8	83.5	32.29345	99	0.949
84140	0.29	4	2.4	5.11765	45.30936	3.119893	1.017605	5.51	5.3	0.2	2	3	1	2	2	1	2	2	21	4	166.2	64.9	23.49539	74.6	0.612
86960	0.64	4	1.53	5.22	38.7	1.94	0.61	3.75	4.8	0.9	2	1	2	2	2	1	2	2	22	3	164.2	65.3	24.21959	93.8	0.823
84565	3.2	5	1.89	5.38	43.92	1.784	0.452	3.88	5.5	1.1	2	3	1	2	2	1	2	2	25	6	156.1	43.6	17.8929	69.1	0.513
88348	1.02	3	1.94	5.281383	50.6613	2.593814	0.728486	4.5	5	2.8	2	1	1	2	2	1	2	2	26	2	165.9	104.2	37.85951	118.2	0.771
85256	2.95	4	1.6	5.233628	61.344	2.18165	0.55152	3.98	4.7	0.6	2	3	2	2	2	1	2	2	27	3	169.5	60.6	21.09275	77.9	0.604
93161	2.51	5	1.78	4.51	66.36	2.974	0.835	5.15	5.3	1	2	3	1	2	2	1	2	2	27	6	162.6	55.3	20.91626	74.2	0.695
84974	3.82	4	1.5	5.25638	56.6664	2.04814	0.614695	3.85	5.5	2.9	2	3	1	2	2	1	2	2	28	3	163.2	69.4	26.05668	82.1	0.841
87165	1.2526	1	1.01	5.3502	82.0236	3.163255	1.52052	5.12	5	1	2	1	1	2	2	1	2	2	35	1	153.5	65.4	27.75626	103.1	0.662
91581	5	5	1.63	5.459457	72.7452	3.9188	1.166269	6.41	4.8	2.3	2	3	2	2	2	1	2	2	35	3	155.9	74.8	30.7758	102.8	0.818
93345	3.03	5	1.53	5	90	3.543	0.666	5.38	5.9	24.3	2	3	1	2	2	1	2	2	36	4	166.1	173	62.70567	165	1.26
84880	2.46	5	1.58	5.6914	75.40976	2.959359	0.98935	5.02	5.7	0.6	2	3	1	2	2	1	2	2	44	4	170.6	97.3	33.43142	101.1	0.661
93206	1.26	4	1.84	5.25	30.3	3.129	0.576	5.22	5.2	0.9	2	2	1	2	2	1	2	2	44	2	152.6	77.2	33.15189	101.2	0.809
84394	1.23	2	1.81	5.5152	63.4752	2.88962	0.885433	4.94	5.7	2.4	2	1	1	2	2	1	2	2	45	2	159.3	82.3	32.43159	98.9	0.865
86184	3.54	5	1.11	5.3388	70.0732	1.855962	0.71608	3.34	5.2	0.08	2	3	1	2	2	1	2	2	18	2	168.6	76.2	26.80648	85.7	0.676
92846	1.77	3	1.19	5.25	92.88	2.689	1.31	4.47	5.4	7.3	2	1	1	2	2	1	2	2	23	3	161.5	102	39.10706	123.6	0.947
87598	0.8328	1	1.29	5.94	192.9	2.483	1.242	4.34	5.4	35.6	2	1	1	2	2	1	2	2	27	2	155	93.7	39.00104	102.7	1.15
84410	0.59	2	0.83	5.66	48.42	2.017	1.264	3.41	5.3	14.7	2	1	1	2	2	1	2	2	28	2	150	64	28.44444	97.6	1.07
90794	0.95	3	1.37	5.11	36.42	2.689	0.621	4.34	5.3	0.3	1	1	1	2	2	1	2	2	33	6	145.3	48	22.73579	77.8	0.627
93275	3.75	5	1.76	5.55	50.22	2.793	1.061	5.04	5	3.2	2	3	2	2	2	1	2	2	40	3	145.7	58.5	27.55731	92.5	0.898
84783	0.75	1	1.11	6.33	105.48	2.586	1.174	4.24	5.6	2	2	1	1	2	2	1	2	2	47	2	141.9	59.7	29.64897	87.4	0.692
88048	2.06	5	1.68	4.83	35.28	2.146	0.452	4.03	5.1	0.08	2	3	1	2	3	1	2	2	31	3	162.7	45.2	17.0751	71.8	0.6
92748	1.19	4	1.84	5.27	25.8	3.336	0.723	5.51	5.7	0.3	2	2	1	1	2	2	2	2	41	4	163.9	71.4	26.5791	93.6	0.479
87044	1.1	4	1.73	4.44	43.68	4.189	1.129	6.44	5.2	0.08	2	1	1	2	2	2	2	2	32	4	172.3	81.5	27.45283	95	0.517
89329	0.25	4	2.79	5.24365	48.666	3.034253	0.897925	5.33	5.1	0.5	2	1	1	2	2	2	2	2	43	4	160.2	57.1	22.24903	76.8	0.778
91043	2.2	4	1.22	5.5577	107.2546	3.253026	1.26964	5.2	5.6	2.8	2	1	1	2	2	2	2	2	44	3	169.7	77.4	26.87678	98.4	0.927
92353	2.82	4	1.27	6.16	101.1	3.103	1.185	4.91	6	16.9	2	1	1	2	2	2	2	2	47	3	161.8	117	44.6919	123.5	1.03
90403	2.49	4	1.89	5.436	59.3052	3.4254	0.95562	5.84	5	1.4	2	2	1	2	2	2	2	2	23	3	168.2	70.9	25.06076	86.5	0.72
90504	0.5	1	1.16	4.72	57.48	3.517	0.7	4.99	5.2	4.4	2	1	1	2	2	2	2	2	18	4	174.4	84.9	27.91353	92.5	0.989
87648	5	5	2.02	4.77	21.3	3.724	0.463	5.95	5.2	0.4	2	2	1	2	2	2	2	2	39	3	161	60.6	23.37873	89.5	0.768
90354	1.07	2	1.4	5.351566	57.2576	3.410833	1.2439	5.46	5.2	0.9	2	1	1	2	2	2	2	2	43	1	153.2	60.9	25.94775	79.7	0.741
88414	0.51	4	0.88	5.4902	77.0964	2.3822	1.38232	4.14	4.6	1.8	2	1	2	2	2	2	2	2	25	3	162.7	52.6	19.87058	73.8	0.359
93612	3.7	5	1.16	5.33	55.62	1.629	4.268	4.76	5.6	3.4	2	1	1	2	2	2	2	2	36	6	162.4	65.7	24.91112	89	0.584
90895	1.03	2	2.17	5.387666	60.75996	3.84432	1.01999	6.65	5.3	0.08	2	1	1	2	2	2	2	2	49	6	154.4	54.5	22.86135	78.6	0.381
84238	1.07	3	1.63	5.0961	48.8598	2.65	0.70271	4.4	5	2.1	1	1	1	2	2	2	2	2	25	4	162.1	78.1	29.72248	97.2	0.83
85525	0.56	1	1.24	5.38	26.52	3.414	3.308	6.18	5	0.4	2	1	1	2	2	2	2	2	40	1	152.1	58.3	25.20056	91	0.787
85443	4.68	3	1.19	5.38	52.86	3.491	1.242	5.25	5.3	0.9	2	1	1	2	2	2	2	2	48	3	158.6	68.2	27.11303	95	0.966
84473	2.27	4	1.14	5.3282	77.2332	2.28352	0.99085	4.16	4.9	1.4	2	3	1	2	2	2	2	2	22	3	152	64.5	27.91724	88.4	0.995
87571	0	1	1.47	5	56.7	1.836	0.621	3.59	4.7	0.08	2	1	2	2	2	2	2	2	19	4	158.5	57.4	22.84827	76	0.564
88876	0.49	3	1.09	4.88	82.56	2.069	0.666	3.47	5.1	3.6	2	1	2	2	2	2	2	2	19	1	159.8	60.1	23.53536	83.5	0.746
92695	0.69	4	1.42	5.44	60.72	3.294666	0.857825	5.22	5.2	5.1	2	1	1	2	2	2	2	2	21	4	167.9	91.9	32.59974	104.4	0.592
88986	1.6	5	1.32	5.94	185.1	1.991	0.96	3.75	5.4	5.3	2	3	1	2	2	2	2	2	22	2	153.9	79.3	33.48081	97.7	0.954
85789	2.45	2	2.15	5.5	52.92	2.56	0.542	4.97	5.3	0.4	2	1	1	2	2	2	2	2	33	4	153.1	57.7	24.61645	85.5	1.1
91810	2.11	2	2.04	5.4182	44.927	2.43238	0.556332	4.6	5.4	0.08	2	1	1	2	2	2	2	2	19	2	157.9	48.4	19.41248	71.7	0.588
93261	0.46	5	1.68	5.44	23.34	2.017	0.565	3.96	5	0.08	2	3	1	2	2	2	2	2	24	6	169.6	50.4	17.5218	72.5	0.558
90540	1.41	1	0.98	5.44	92.28	1.577	2.89	3.88	5.4	1.9	2	1	1	2	2	2	2	2	24	1	150.5	56.7	25.03284	82.7	0.623
88608	0.63	1	1.45	5.22	45.1452	1.776789	0.574328	3.1	5	0.08	2	1	2	2	2	2	2	2	28	4	160.9	54.2	20.93569	73.7	0.474

88795	0.54	2	1.5	6.1	94.8	2.483	1.14	4.5	5.4	2.3	2	1	1	2	2	2	2	2	30	1	151.7	67.3	29.24448	88.8	0.665
93428	0.3	1	1.34	4.77	79.44	2.276	1.14	4.14	5.9	5.5	2	1	1	2	2	2	2	2	30	1	149.9	65.8	29.28348	90.9	0.755
87330	0.74	3	1.73	5.1462	59.4096	2.9325	0.92018	5.02	5.1	5.2	2	1	1	2	2	2	2	2	36	3	158.2	66.6	26.611	86.6	0.892
91017	4.22	2	0.59	4.72	124.62	2.302	1.073	3.39	5.9	15.8	2	3	1	1	1	3	2	2	48	2	155.6	91.8	37.91609	113.6	0.311
86082	2.68	4	0.96	7.3654	199.224	3.64982	2.10216	5.59	6.3	5.6	2	3	1	2	1	3	2	2	49	2	173.7	103.5	34.30368	115.5	0.912
84641	0.15	2	0.98	7.604090	178.9128	2.699476	1.69784	4.34	6.1	2.3	2	1	2	2	1	3	2	2	28	4	165.8	93.1	33.8673	113	0.855
92922	0.85	2	0.91	8.2902	174.048	2.42158	1.43974	3.54	7.8	28.1	1	1	1	1	2	3	2	2	30	4	163.6	114.1	42.63036	134.1	1.53
84409	1.03	3	1.89	5.83	40.5	3.62	1.061	6	5.6	7.9	1	1	1	1	2	3	2	2	47	3	152.3	44.2	19.05559	69.9	1.17
88351	1.24	4	1.19	4.72	52.5	2.44648	0.92164	4.11	5.3	7.7	1	1	1	2	2	3	2	2	41	3	162.6	103.8	39.26054	118.8	1.13
87712	0.77	3	1.37	4.72	65.4	2.073809	0.71252	3.47	5.4	2.6	1	1	2	2	2	3	2	2	33	4	163.5	60.2	22.51962	85.3	0.84
84581	2.02	5	1.42	5.11	43.62	2.327	0.632	4.03	4.9	7.4	1	2	1	2	2	3	2	2	32	2	160.9	105.8	40.86708	123.5	1.04
85286	0.04	3	0.83	4.05	37.8	3.077	0.79	4.27	4.5	0.8	2	1	1	2	2	3	2	2	18	7	164.3	61.7	22.85652	83.6	0.566
85243	2.67	4	0.91	5.944911	125.7746	3.84852	1.77264	5.77	6.2	6	2	3	1	2	2	3	2	2	43	2	161.9	104.9	40.02043	116.9	0.75
93074	5	5	1.11	5.1898	105.0888	3.104273	1.5871	5.04	5.1	1.5	2	1	1	2	2	3	2	2	44	3	172.4	86.1	28.96867	99	0.708
91663	0.88	3	1.55	5.33	40.56	3.051	0.745	4.94	5.3	0.6	2	1	1	2	2	3	2	2	46	6	161.3	63.8	24.52178	92.2	0.699
88005	0.03	3	1.32	5.31	65.34	2.508	1.061	4.32	5	1.4	2	1	2	2	2	3	2	2	21	1	159.5	88.4	34.74809	111.7	0.837
87891	0.34	3	1.63	4.94	54.96	2.87	1.502	5.2	5.2	1.3	2	1	1	2	2	3	2	2	42	3	168.9	82.8	29.02492	98.8	0.855
91793	1.83	2	1.63	5.83	112.86	3.233	0.881	5.28	5.6	0.4	2	1	1	2	2	3	2	2	44	3	163.7	62.1	23.17364	82.1	0.575
92363	0.09	3	1.42	5.11	153.6	2.612	0.61	4.32	5.6	2.8	2	2	1	2	2	3	2	2	27	4	170	138	47.75087	134.7	1.08
87174	0.62	4	1.89	5.61	40.74	3.258	0.745	5.48	5.3	3	2	3	1	2	2	3	2	2	27	3	169.3	61	21.28217	85.4	0.768
87236	3.54	5	1.06	11.9004	208.0776	3.7513	1.7114	5.64	9.8	6.1	2	3	1	1	1	0	2	2	45	2	156.5	81.7	33.35749	109.7	0.953
89762	1.380657	2	1.22	5.55	50.16	2.793	1.434	4.65	5.4	7.1	2	1	1	1	2	0	2	2	35	1	152.5	70	30.09944	95.9	0.821
86586	2.15	2	0.85	5.6832	78.4692	2.173592	0.961	3.67	4.7	1	2	3	1	2	2	0	2	2	28	1	152.6	88.2	37.8756	101.8	0.629
90527	2.88	1	1.29	5.2224	56.5548	2.97958	0.9199	4.84	5.1	0.08	2	1	1	2	2	0	2	2	38	6	158.2	48.8	19.49875	72.5	0.356
90755	1.86	4	1.94	4.72	34.08	1.991	0.452	4.14	4.9	2	2	3	1	2	2	0	2	2	39	4	161.9	77	29.3763	97	0.569
88696	5	5	1.71	5.77	45.78	3.491	0.734	5.53	5.5	4	2	3	1	2	2	0	2	2	47	2	165.7	87.1	31.72292	108.1	0.871
83853	5	3	1.58	6.99	42	4.241	1.005	6.28	5.1	0.9	2	3	1	2	2	0	2	2	49	3	166.7	76.1	27.38504	88.7	0.718
86855	1.03	4	0.91	5.785	170.976	2.29694	1.2062	3.59	5.5	4.3	2	1	2	2	2	0	2	2	39	2	159.4	145.3	57.1859	151.3	0.789
87925	5	5	0.93	5.3162	114.9828	3.028900	1.674386	4.94	5.4	1.4	2	1	1	2	2	0	2	2	33	3	157.4	104.5	42.18003	118.4	0.572
85468	3.57	4	1.66	5.2824	54.6804	3.199151	1.003093	5.38	5.2	2.1	2	1	1	2	2	0	2	2	46	6	146.7	54.1	25.13837	81	0.701
84893	2.473516	4	2.12	6.44	34.5	2.224	0.914	4.76	5	1.3	2	2	1	2	2	0	2	2	46	4	164.9	79.9	29.38363	107.7	0.943
85362	1.79	4	1.09	5	20.82	2.605388	0.88742	4.58	5.4	1.4	2	3	1	2	2	0	2	2	20	3	162.7	55	20.77723	81.2	1.17
91405	0.25	1	1.68	5.156833	34.206	2.13246	0.61144	4.16	5	0.4	2	3	1	2	2	0	2	2	24	1	152.5	74.2	31.9054	94.4	0.872
86707	1.36	4	1.34	5.94	157.02	2.146	0.723	3.83	5.7	6	2	3	1	2	2	0	2	2	30	4	156.1	93.6	38.41228	115	1.1
89076	1.73	4	2.15	4.9772	37.1004	2.278166	0.539586	4.58	5	0.08	2	3	1	2	2	0	2	2	37	3	161.2	50.3	19.35699	72	0.485
93186	2.08	4	2.3	5.024066	58.9392	3.40482	1.1506	5.74	4	5.3	2	3	1	2	2	0	2	2	38	4	170	85.3	29.51557	101.4	0.705
85204	0.5	3	1.58	5.12885	40.9626	2.23538	0.60791	4.11	4.6	0.3	2	3	1	2	2	0	2	2	20	2	157.3	57.6	23.27904	84.8	0.485
89529	1.56	4	1.01	5.367	77.08253	3.048573	1.24822	4.91	5.3	0.3	2	1	1	2	2	0	2	2	20	2	166.1	75.1	27.22079	92	0.817
87326	1.27	5	2.02	5.2594	44.3316	3.080748	0.68627	5.33	5.5	0.5	2	3	1	2	2	0	2	2	25	3	164	57.7	21.453	75.7	0.529
86425	0.13	3	1.32	5.42	126.72	2.276	0.734	3.93	5.7	19.2	2	1	1	2	2	0	2	2	25	1	156	119.6	49.1453	122.8	0.975
85264	3.54	5	1.89	5.127	47.7492	2.723073	0.856033	4.89	5.1	7.9	2	3	1	2	2	0	2	2	26	3	170.2	61.2	21.12673	78.8	0.495
90832	2.12	5	1.71	5.14	38.58	1.598975	0.5297	3.03	5	0.5	2	3	1	2	2	0	2	2	26	2	163.3	61.3	22.98731	78	0.613
90754	1.251760	3	1.27	5.264747	68.4264	2.19918	0.786948	3.83	5.2	3.6	2	1	1	2	2	0	2	2	27	1	153.3	62.4	26.55219	90.3	0.612
84277	0.46	1	0.98	5.483	100.0152	3.84432	2.03598	6.05	5.6	5.8	2	1	1	2	2	0	2	2	28	1	155.8	124.9	51.45499	144.1	0.43
84230	2.3	5	1.45	5.4722	94.9272	2.390686	0.96964	4.16	5.5	14	2	1	1	2	2	0	2	2	28	2	156.8	142.1	57.79656	141	1.23
85171	2.64	4	1.06	5.25	40.08	2.431	1.242	4.06	5	1.4	2	1	1	2	2	0	2	2	29	2	148.4	66.3	30.10549	91.4	0.681
88910	3.62	5	1.32	5.33	99.18	3.931	1.174	5.79	5.4	5	2	3	2	2	2	0	2	2	29	4	155.6	98.8	40.80729	114.4	1.15
89345	5	5	2.07	4.74	13.44	3.155	0.418	5.4	5.1	2.1	2	2	1	2	2	0	2	2	30	2	155.9	55.8	22.95842	86.1	0.784
86923	5	5	1.37	5	44.58	3.569	1.197	5.48	5.5	2.6	2	3	1	2	2	0	2	2	31	6	159.6	70.2	27.5595	96.8	0.78
91876	2.414483	5	1.14	5.27	100.92	2.818406	0.995301	4.5	5	0.6	2	1	1	2	2	0	2	2	33	6	164.5	78.8	29.1202	95.5	0.826
92823	5	5	1.29	5.11	18.72	1.758	0.587	3.31	5.2	0.5	2	3	1	2	2	0	2	2	34	6	159.5	47.7	18.74982	70.6	0.566
92735	4.53	3	1.16	5.759056	96.6345	2.404953	1.06578	4.01	5.6	5.1	2	3	1	2	2	0	2	2	34	6	153.8	88.3	37.32914	101	1.07
90798	0.7	1	0.65	6.7358	218.0628	2.2675	2.04584	3.75	5.6	14.6	2	1	1	2	2	0	2	2	35	2	151.2	71.7	31.36285	97.2	1.16
85193	5	4	1.81	5.16265	40.40888	2.50393	0.56095	4.45	5.6	0.3	2	3	1	2	2	0	2	2	36	6	156.9	52.9	21.48868	81.3	0.442
84444	3.99	4	1.45	5.418	74.20464	3.04628	1.10432	5.07	5.5	1.8	2	1	1	2	2	0	2	2	36	2	156.5	57.6	23.51764	89.2	0.815
85613	0	5	2.28	4.91	24.42	2.224	0.339	4.65	5.1	1.4	2	2	1	2	2	0	2	2	37	2	171.2	84.2	28.72795	96.2	0.725
88949	4.04	5	1.11	5.467777	88.4016	3.526834	1.68749	5.48	5.4	1.2	2	1	1	2	2	0	2	2	38	2	154.6	63.4	26.52591	93.5	0.693
88591	1.65	4	1.14	4.61	127.32	2.37504	0.997051	4.22	5	1	2	1	1	2	2	0	2	2	38	1	146.4	62.8	29.30067	99.4	0.718
92687	5	5	1.32	5.3688	74.43	3.26768	1.154093	5.17	5.5	1.7	2	1	1	2	2	0	2	2	38	6	158.7	63.4	25.17302	86.1	0.847
92062	3.91	5	1.68	5.55	61.62	1.862	0.802	3.9	5.3	1.1	2	3	1	2	2	0	2	2	40	1	155.7	66.5	27.43118	92.5	0.828

93682	5	5	1.68	6.83	221.16	2.719195	1.552282	4.73	5.5	2.8	2	3	1	2	2	0	2	2	41	6	166.9	58.2	20.89346	80.8	0.328
88933	2.47	4	1.22	5.6928	91.65221	2.622942	0.857251	4.29	5.6	1.9	2	3	1	2	2	0	2	2	41	4	170.8	93.3	31.98202	110.5	0.629
88552	5	5	1.84	5.16	14.58	2.431	0.44	4.47	5.2	0.2	2	2	1	2	2	0	2	2	42	6	166.3	52.1	18.8388	73.9	0.388
84242	3.87	5	1.5	5.358666	47.1856	2.962278	0.78122	4.84	5.5	0.6	2	3	1	2	2	0	2	2	42	6	154.3	52.3	21.96695	80.9	0.515
88185	1.34	1	1.29	5.8093	122.7876	3.246475	1.14892	5.28	5.5	20.7	2	1	1	2	2	0	2	2	42	6	150.1	50.7	22.50332	88.5	1.25
91426	2.822593	4	2.2	5.37105	40.7614	2.947135	0.72452	5.28	5.7	0.08	2	3	1	2	2	0	2	2	43	6	163	50.5	19.00711	78.7	0.312
88950	5	5	2.35	4.72	45.54	2.810365	0.704911	5.28	5.3	0.3	2	3	1	2	2	0	2	2	43	6	153.7	53.8	22.77375	72.8	0.531
93426	2.67	5	2.07	5.43698	42.201	3.095015	0.76936	5.61	5.2	0.08	2	3	1	2	2	0	2	2	43	2	156.7	56.6	23.0504	85.6	0.717
93516	2.62	4	1.16	5.2702	108.9362	3.113106	1.355593	4.91	5.3	3.1	2	1	1	2	2	0	2	2	43	2	165.3	82.7	30.26633	102.2	0.935
88798	1.279266	3	0.91	5.470466	97.6263	2.17124	1.548388	3.93	5.4	7.2	2	1	2	2	2	0	2	2	43	4	150.9	75	32.9369	106.7	0.994
92346	3	5	1.47	5.33	104.16	2.922	1.208	4.94	5.3	1.5	2	1	1	2	2	0	2	2	43	2	155.6	88.6	36.59439	108.8	1.3
84689	5	5	1.5	6.1274	108.9816	2.45794	1.11312	4.14	6.1	19.2	2	3	1	2	2	0	2	2	47	6	159.7	71.9	28.19156	96.7	1.12
93252	1.845992	3	1.63	4.94	31.26	3.362	0.869	5.38	5.6	3.1	2	1	1	2	2	0	2	2	48	2	146	69.9	32.79227	98.6	0.728
89908	3.2324	3	1.63	5.248125	67.9258	2.665436	0.87842	4.65	5	7.9	2	3	2	2	2	0	2	2	30	1	164.7	77.8	28.68088	103.5	0.581
83762	2.12	4	0.88	5.36075	110.6172	2.494566	1.27695	4.24	5	7.6	2	1	1	2	2	0	2	2	27	4	168.5	107.9	38.00333	114.8	0.873
89986	1.029133	2	1.03	6.11	43.74	2.56	2.235	4.63	5.4	1.5	2	1	1	2	2	0	2	2	28	3	154.9	79.2	33.00824	94.5	0.7
86933	0.62	3	1.6	5.77	71.58	1.215	0.598	3.1	5.3	0.8	2	3	2	2	2	0	2	2	30	1	155.7	68.4	28.21492	92.9	0.681
87602	1.96	4	1.27	5.05	58.92	2.638	0.689	4.22	5.5	0.08	2	1	1	2	2	0	2	2	18	7	156.8	52.1	21.19071	71.6	0.478
92871	3.07	5	1.42	5.396733	68.56251	2.091302	0.76686	3.83	5	0.9	2	3	2	2	2	0	2	2	23	3	167.5	53.9	19.21141	69.9	0.639
83823	0.35	1	0.96	5.66	118.62	4.189	3.105	6.57	5.7	4.8	2	2	1	2	2	0	2	2	29	1	150	66.8	29.68889	96.5	0.66
91775	2.18	5	2.33	5.24245	41.65054	3.010715	0.71718	5.53	5.1	0.3	2	3	1	2	2	0	2	2	30	3	167.7	82	29.15733	99.2	0.785
92825	0.28	2	1.55	5.397985	56.25222	2.88294	0.88756	4.76	5.3	3.2	2	1	1	2	2	0	2	2	31	4	166.2	80.5	29.14297	92	0.936
90851	2.514751	5	1.55	4.88	22.5	1.471888	0.614020	2.97	4.9	0.5	2	3	1	2	2	0	2	2	32	7	158	55.5	22.23201	81.8	0.591
88803	0.92	4	1.37	5.0446	71.7216	3.148626	0.9685	4.86	4.8	3.7	2	1	1	2	2	0	2	2	32	4	160.6	74.7	28.96206	90.4	0.692
86172	3.23	1	1.29	5.61	154.92	1.655	0.847	3.34	5.2	4.8	2	3	1	2	2	0	2	2	33	3	164.9	102.5	37.6949	112	0.999
92107	0.7	1	1.42	4.94	57.42	1.991	0.937	3.85	5.8	1.9	2	1	1	2	2	0	2	2	38	6	150.6	81.6	35.97826	104.2	0.535
91189	1.4	4	2.15	5.5	57.6	1.707	0.474	4.06	5.2	0.08	2	1	1	2	2	0	2	2	39	6	163.1	57.7	21.69042	84	0.453
84745	2.11	1	1.76	5.287181	38.658	1.979646	0.510115	3.88	5.3	0.08	2	3	1	2	2	0	2	2	42	1	156.9	57.5	23.35726	81.2	0.613
91505	1.38	1	1.53	4.39	57.6	3.233	0.632	5.04	5.9	0.3	2	1	1	2	2	0	2	2	45	6	165.2	61.3	22.46158	81.4	0.386
86200	0.91	1	1.63	4.94	44.7	3	0.531	4.86	5.3	3.4	2	1	1	2	2	0	2	2	48	7	167.3	94.8	33.8701	113.7	0.657
92233	1.32	3	1.09	5	38.04	2.948	2.868	5.35	5.4	1.2	2	1	1	2	2	0	2	2	49	6	156.9	53.9	21.8949	80.3	0.511
101168	0.06	2	1.27	5.5498	60.92862	2.87506	0.683633	4.47	5.2	0.91	2	1	1	2	2	0	2	2	18	3	163	47.1	17.72743	70	0.379
97313	5	5	1.91	5.27	38.7	2.56	0.734	4.81	5.2	3.76	2	3	1	2	2	0	2	2	18	3	166.9	57.1	20.49856	68	0.395
98883	0.96	2	1.42	5.3854	64.2768	2.59278	0.71198	4.29	5	0.47	2	1	2	2	2	0	2	2	18	1	158.6	59.1	23.49531	76	0.44
95420	1.16	3	1.24	5.262595	58.2252	1.941098	0.694043	3.49	5	0.29	2	1	2	2	2	0	2	2	18	1	150.8	57.7	25.37308	82.2	0.47
95948	2.64	5	1.4	5.2824	48.16574	2.530849	0.66608	4.29	5.2	0.37	2	3	1	2	2	0	2	2	18	3	170.8	67.6	23.1724	84.9	0.477
95927	3.585120	5	2.09	6.16	39.36	3	0.621	5.38	5	0.49	2	3	1	2	2	0	2	2	18	4	160.5	50.2	19.48739	66.4	0.51
99201	5	5	1.94	5.472466	96.6774	2.90044	1.189293	5.17	5.4	8.94	2	3	1	2	2	0	2	2	18	7	162.3	66	25.05572	77.3	0.51
94148	1.73	5	1.53	5.140770	44.9832	2.559915	0.580633	4.42	5.3	0.21	2	1	1	2	2	0	2	2	18	1	165.3	44.7	16.35919	66	0.517
98022	1.64	5	1.47	5.168228	56.65221	1.779349	0.510948	3.34	4.9	0.73	2	3	2	2	2	0	2	2	18	4	162.4	62.5	23.6978	81.3	0.52
97806	0.29	3	1.81	5.159813	42.5952	2.65812	0.57315	4.55	5.5	0.36	2	1	1	2	2	1	2	2	18	3	165.3	61	22.32462	81.2	0.531
94467	0.93	4	1.86	4.8534	31.5162	2.07086	0.521473	4.19	4.8	0.36	2	3	1	2	2	0	1	2	18	6	161.6	56.3	21.55885	75.3	0.547
96565	1.41	4	1.19	5.33	105.78	2.612	0.971	4.24	5	0.38	2	1	1	2	2	0	2	2	18	1	168.8	110.9	38.92124	114.5	0.55
100778	1.79	5	1.76	4.72	27.48	2.043	0.565	4.06	5.1	0.24	2	3	1	2	2	0	2	2	18	4	176.2	50.6	16.29816	66.3	0.56
94723	1.15	3	1.45	5.204913	43.16534	2.65884	0.66271	4.4	5.5	0.35	2	1	1	2	2	0	2	2	18	3	174.3	59.1	19.45327	75.9	0.593
99275	0.87	4	1.47	5.0278	56.7168	2.05432	0.68337	3.9	5	0.88	2	3	2	2	2	0	1	2	18	2	162.4	71.8	27.22403	94.8	0.6
98212	0	1	2.35	5.3406	75.423	3.719048	1.37329	6.31	5.3	1.12	2	3	1	2	2	1	2	2	18	3	160.8	55.4	21.42583	77.1	0.611
94235	0.17	3	1.53	5.2111	57.4728	1.73436	0.5832	3.26	5	2.4	2	1	2	2	2	0	1	2	18	3	164	63.2	23.49792	80.5	0.616
100940	0.68	5	1.66	5.103228	28.49688	1.894887	0.534342	3.88	5	0.29	2	3	1	2	2	0	2	2	18	3	163	44.5	16.74884	66.8	0.63
93938	3.37	5	1.63	5.246481	48.43282	2.72819	0.74086	4.58	5.3	8.72	2	3	1	2	2	0	2	2	18	7	163.2	63.1	23.69131	92.1	0.63
99757	0.8754	3	1.66	5.0626	23.49590	1.717476	0.541354	3.44	5	0.79	2	1	1	2	2	1	1	2	18	2	154.6	47.7	19.95719	69.3	0.64
100287	4.93	5	1.11	5.61	44.58	2.612	0.576	3.98	5.1	3.75	2	3	1	2	2	0	2	2	18	1	157.3	54.5	22.02617	75.1	0.646
94305	3.71	5	1.42	5.349533	63.23571	2.022151	0.704728	3.78	4.9	0.82	2	3	2	2	2	0	2	2	18	3	152.2	60.4	26.074	82.5	0.66
95628	1.02	2	1.6	5.55	40.92	2.896	0.463	4.71	5.3	0.72	2	1	1	2	2	0	2	2	18	7	166.2	55	19.91135	74.8	0.662
94001	2.3164	4	1.29	5.16	46.32	1.552	0.587	3.1	5.1	0.45	2	1	1	2	2	0	2	2	18	1	158.6	51.6	20.51367	75.8	0.67
96241	5	5	1.94	5.600948	60.7188	3.995293	1.05764	6.72	5	1.56	2	3	2	2	2	0	2	2	18	6	161	47.3	18.24775	75.1	0.69
97918	2.03	3	1.47	4.83	36.54	3	0.914	4.89	5.1	1.25	2	1	1	2	2	0	2	2	18	6	165	69.4	25.49128	89.8	0.699
101318	5	5	1.657191	5.121333	62.4244	2.569192	0.6101	4.529733	5	2.421846	2	3	2	2	2	0	2	2	18	3	161.2	53.1	20.43452	70.5	0.7
96516	0.08	4	2.17	4.66	43.8	2.767	1.264	5.51	5	3.08	2	3	1	2	2	0	2	2	18	3	160.5	64.9	25.19385	87	0.705
98277	5	5	1.34	5.3944	80.7804	2.699333	0.983366	4.5	5.1	14.4	2	1	1	2	2	0	1	2	18	3	162.2	58.1	22.08383	76.1	0.737

100978	2.23	4	1.24	5.05	55.92	3.258	0.802	4.86	5.3	1.33	2	1	1	2	2	0	2	2	18	1	157.2	48.5	19.62622	71.4	0.772
94994	1.923147	4	1.14	5	92.88	3.284	1.389	5.07	4.9	3.71	2	1	1	2	2	0	2	2	18	2	147	59.3	27.44227	98.7	0.78
96693	2.76	5	1.47	5.1948	52.4964	2.79502	0.69382	4.63	5.1	1.5	2	3	1	2	2	0	2	2	18	7	153.8	47.2	19.95397	66.9	0.781
102366	2.03	4	0.85	5.5666	79.94333	2.401984	1.07286	4.14	5.2	0.93	2	1	1	2	2	0	2	2	18	1	159.8	79.5	31.13247	106.3	0.784
98170	2.114270	4	1.16	5.289233	97.6044	3.242915	1.4043	5.04	5.2	2.08	2	1	1	2	2	0	2	2	18	1	162.9	75.1	28.30074	88.1	0.79
95874	0.82	4	1.71	5.83	127.86	2.017	0.294	3.85	5.5	1.46	2	3	1	2	2	0	1	2	18	2	160.4	84.9	32.99886	97.7	0.8
97882	5	5	1.4	5.4078	78.5988	2.15498	0.879786	3.88	5.4	5.98	2	3	1	2	2	0	1	2	18	1	162.6	83	31.3933	95.8	0.88
100277	2.64	4	1.37	5.66	124.56	3.233	1.569	5.33	5.2	12.88	2	3	1	2	2	0	1	2	18	3	164.2	96.3	35.71741	100.5	0.917
102086	0.28	3	0.98	5.8922	147.3144	2.22508	1.26166	3.41	5.4	5.33	2	1	1	2	2	0	1	2	18	4	158.2	94.7	37.83877	113.7	1.05
100262	0	2	1.34	5.593	97.2168	2.111251	0.84394	3.49	5.5	1.74	2	1	2	2	2	0	1	2	18	1	167.5	67.9	24.20138	85.5	1.06
102741	3.73	4	1.03	6.1772	163.1708	2.87666	1.5281	4.6	6.2	8.06	2	3	1	2	2	0	1	2	18	6	166.6	107.5	38.73098	113.7	1.06
94394	0.87	2	1.5	5.664338	77.692	2.8457	0.89581	4.6	5.9	3.3	2	1	1	2	2	0	2	2	18	7	157.4	101	40.7673	106.7	1.1
97631	3.03	5	1.37	5.5316	89.62987	1.790164	0.753175	2.38	5.4	6.4	2	3	2	2	2	0	1	2	18	4	165.6	100	36.46526	102.7	1.15
97173	4.6	5	1.32	5.77	63.3	1.862	0.553	3.44	5.8	2.27	2	3	1	2	2	0	2	2	18	3	166.5	64.6	23.30258	81.8	1.17
98411	4.6	5	1.37	5.731	99.9456	4.25512	1.485646	6.52	5.2	15.18	2	3	1	2	2	0	1	2	18	7	166.1	93.4	33.85381	99.2	1.24
100035	0.92	3	1.03	5.5928	119.8332	2.133	1.23328	3.49	5.5	8.3	2	1	2	2	2	0	1	2	18	4	149.3	54.5	24.44989	82.7	1.29
101130	2.65	5	1.78	4.77	44.94	1.552	0.677	3.65	5.4	0.99	2	3	1	2	2	0	2	2	19	2	161.8	54.6	20.85622	72.7	0.284
98408	1.71	3	1.45	5.44	56.7	1.991	0.429	3.65	5.1	0.8	2	3	1	2	2	0	1	2	19	3	163.8	54.1	20.16366	70.1	0.368
99771	1.64	4	1.32	5.1763	73.5564	2.6475	0.85775	4.37	4.9	2.45	2	1	2	2	2	0	1	2	19	7	160.4	59.1	22.97094	73.6	0.38
100003	0.12	4	1.3827	5	73.5276	2.5343	0.77108	4.239821	5	5.7567	2	1	2	2	2	0	2	2	19	2	165	62.5	22.95684	83.7	0.402
98440	0.74	4	1.53	4.94	23.16	1.914	0.418	3.62	5.7	1.45	2	1	1	2	2	1	1	2	19	4	154.6	47.7	19.95719	64.7	0.412
94214	4.57	5	1.63	5.310133	67.33531	1.679497	0.53346	2.77	5	2.13	2	3	2	2	2	0	2	2	19	3	159.6	56.4	22.14182	77.8	0.45
97621	0.74	4	1.45	5.018692	41.5	1.543356	0.471166	2.95	5.3	0.36	2	1	1	2	2	1	1	2	19	4	163.6	54.7	20.43717	72	0.452
100457	2.7605	4	1.63	5.2042	67.7676	2.39867	0.57184	4.29	4.9	1.24	2	3	2	2	2	0	1	2	19	2	152	54.8	23.71884	70.1	0.453
99013	1.52	3	1.81	5.22	49.8	2.069	0.463	4.09	4.6	0.11	2	3	1	2	2	0	1	2	19	1	159.2	52.8	20.83281	76.6	0.481
97589	0.74	3	1.6	4.83	19.92	1.655	0.542	3.52	5.3	0.63	1	1	1	2	2	1	1	2	19	2	153	57.4	24.52048	78.6	0.487
95741	0.25	2	1.78	4.94	16.14	1.5	0.485	3.52	5	0.39	2	1	1	2	2	1	2	2	19	3	163.2	64.3	24.14186	64.5	0.497
94364	0.96	3	1.91	5.2527	94.422	2.925775	1.112668	5.2	5.3	12.07	2	1	1	2	2	0	2	2	19	3	166.3	75.9	27.44462	90	0.53
97146	5	5	1.22	5.5	76.5	2.845	1.027	4.53	5.1	0.15	2	3	1	2	2	0	2	2	19	7	154.2	50.3	21.15433	73.2	0.531
102487	5	5	2.22	5.61	37.98	1.862	0.768	4.45	5	0.27	2	3	1	2	2	0	2	2	19	6	164.2	42.3	15.68896	65	0.54
94546	0.37	3	1.55	5.140213	43.9012	2.766133	0.64698	4.53	5.5	0.61	1	1	1	2	2	1	2	2	19	7	164	66.1	24.57615	76.9	0.57
96734	2.21	4	1.27	5.188133	49.26896	1.874332	0.65918	3.49	5.3	0.36	2	1	1	2	2	0	2	2	19	4	154.7	62.5	26.11556	84.1	0.58
99428	3.3	5	1.37	5.285333	45.89894	1.825309	0.57602	3.49	5.1	0.41	2	3	1	2	2	0	2	2	19	2	159.4	55.4	21.80385	77.9	0.583
94587	3.1	5	1.68	5.61	25.2	2.198	0.237	3.98	5.3	0.31	2	3	1	2	2	0	2	2	19	3	158.6	50.7	20.15587	69.5	0.599
93858	0.33	4	1.78	5.248933	31.8784	1.854770	0.60214	3.83	5.2	0.57	2	1	1	2	2	0	1	2	19	7	147.3	36.2	16.68411	56.4	0.67
100014	3.08	3	1.06	5.5486	65.21573	2.1248	0.97584	3.9	5.1	2.05	2	3	1	2	2	0	1	2	19	6	155.9	57.5	23.65787	73.8	0.671
100009	4.21	3	1.42	5.200625	63.4332	2.884768	1.092408	4.81	5.1	10.69	2	1	1	2	2	0	2	2	19	3	153.5	86.5	36.71126	102.3	0.68
102660	1.2	4	2.02	4.39	38.16	1.707	0.361	3.88	4.6	1.42	2	3	1	2	2	0	1	2	19	1	156.4	89.7	36.67068	90.8	0.702
94516	0.17	2	1.47	4.88	39.42	3.31	0.452	4.99	5.4	1.24	2	1	1	2	2	0	2	2	19	4	158.9	62.3	24.67404	83.9	0.705
97184	1.72	4	1.03	5.22	79.98	1.836	0.723	3.21	5.3	1	2	3	2	2	2	0	1	2	19	3	166.3	79.2	28.63787	96.5	0.741
94078	2.364655	4	0.96	5.2262	67.0287	2.77322	0.95311	4.45	4.6	0.4	2	1	1	2	2	0	2	2	19	3	173.4	78.9	26.24091	89.9	0.779
96311	1.14	3	1.47	5.22	47.88	1.526	0.373	3.18	5.6	2.36	2	3	1	2	2	0	1	2	19	4	170.9	54.6	18.69427	66	0.781
97723	0.45	1	0.78	6.0142	140.2388	2.14438	1.3137	3.62	5.5	3.89	2	1	2	2	2	0	2	2	19	1	165.3	75.8	27.74109	94.4	0.804
102133	0.33	4	1.63	5.55	38.64	1.707	0.497	3.57	5.3	1.39	2	1	1	2	2	0	1	2	19	3	161.9	62.1	23.69179	78.3	0.818
97694	0.99	3	1.06	5.4075	92.574	3.184728	1.420428	5.04	5.1	6.84	2	1	1	2	2	0	2	2	19	3	163	95.5	35.94415	109.6	0.849
97378	1.91	4	1.29	5.27	52.44	2.405	0.316	3.85	4.8	5.26	2	3	2	2	2	1	1	2	19	4	168.9	109.7	38.45451	119.6	0.853
98111	2.45	2	1.22	5.46	88.88664	2.415816	1.038393	4.16	5	4.64	2	1	1	2	2	0	2	2	19	7	171.7	84	28.49303	97.5	0.855
101113	4.86	4	0.75	5.44	102.9	3	0.587	4.01	5.8	0.46	2	1	1	2	2	0	2	2	19	3	160.3	65.1	25.33459	82.4	0.876
102416	2	4	1.63	5.16	77.64	2.586	0.485	4.45	5.3	3.91	2	3	1	2	2	0	1	2	19	1	170	82	28.3737	102	0.913
94353	1.39	4	0.93	5.8582	136.6398	2.88852	1.172675	4.58	5.3	80.09	2	2	1	2	2	0	1	2	19	3	159.3	50.4	19.8609	80.4	0.916
102323	1.19	5	1.42	5.11	36.54	1.345	0.519	3	5.3	2.1	2	3	1	2	2	0	2	2	19	2	155.5	48.2	19.93362	73.9	0.92
95198	0.04	3	1.01	5.5972	105.1181	3.165795	1.52454	5.02	5.6	2.41	2	1	1	2	2	0	2	2	19	4	163.5	99.7	37.29578	107.1	0.922
102019	0	2	1.34	5.38	75.18	2.793	0.666	4.45	5.3	0.78	2	1	1	2	2	0	2	2	19	3	153.1	54.2	23.12325	85.4	0.957
93778	5	5	1.24	5.4926	75.2916	2.5072	1.16676	4.24	5.5	3.47	2	3	1	2	2	0	2	2	19	4	163.6	73.3	27.38655	84.4	0.957
94905	5	5	1.11	5	63.72	1.991	0.61	3.39	4.9	0.76	2	3	2	2	2	0	2	2	19	3	161	51.3	19.7909	72.4	0.96
99892	0.5	3	1.4	5.33	84.06	1.94	0.937	3.78	5	1.21	2	1	2	2	2	0	1	2	19	3	165.4	58	21.20103	73.8	0.995
95195	0.41	3	1.22	5.30475	80.3904	1.67116	0.690848	3.1	5.5	3.01	2	1	2	2	2	0	1	2	19	2	169.1	124.9	43.67926	136.6	1.02
95808	0	1	1.4	6.669	142.0848	4.42098	1.90564	8.02	5.4	33.15	2	2	1	2	2	0	2	2	19	3	163.9	69.3	25.79736	87.9	1.03
96759	1.907562	4	1.09	5.22	112.14	2.689	1.592	4.5	4.8	8.8	2	1	1	2	2	0	1	2	19	3	166.4	123	44.42204	134.8	1.06
98255	0.815975	2	0.91	5.7092	118.4592	2.77474	1.4551	4.27	5.5	2.87	2	1	1	2	2	0	2	2	19	4	155.3	66.6	27.61413	84.8	1.08

101089	0.01	2	1.14	5.5596	123.4764	2.181086	1.2496	3.72	5.5	13.24	2	1	1	2	2	0	2	2	19	1	159.7	125.7	49.28621	134.5	1.1
99968	0.55	3	0.91	5.790666	166.2132	2.09372	1.22128	3.05	5.6	9.47	2	1	2	2	2	0	1	2	19	3	155.4	85.1	35.23932	104.2	1.12
96280	1.88	4	1.11	6.005	147.5654	2.240265	1.27972	3.96	5.7	8.05	2	3	1	2	2	0	1	2	19	1	159.1	87.5	34.56748	108.5	1.16
100814	0.49	2	1.4	5.232719	73.1988	3.068741	1.206953	5.07	4.7	6.34	2	1	1	2	2	0	2	2	19	3	167.7	110.1	39.14905	123.2	1.23
95766	2.89	5	1.24	5.83	93.36	2.457	1.016	4.16	5	4.28	2	3	2	2	2	0	2	2	19	3	163.9	92.6	34.47094	99.8	1.27
102393	0.41	4	1.63	5.38	21.3	1.784	0.497	3.65	5.4	0.36	2	1	1	2	2	0	1	2	20	3	171	52.6	17.98844	71.7	0.419
99110	0.58	3	1.58	5.22	40.38	1.758	0.711	3.67	5.3	2.29	2	1	1	2	2	0	1	2	20	1	146.6	43.2	20.10091	70.5	0.487
102899	1.68	4	1.66	4.94	58.02	2.689	0.926	4.76	5	0.85	2	3	1	2	2	0	1	2	20	3	171.9	69	23.35056	82.2	0.501
96355	0.45	2	1.55	5.307633	37.0128	2.067858	0.61584	3.93	5.1	0.43	2	1	1	2	2	0	2	2	20	1	149.5	39.1	17.49421	71.2	0.504
96035	2.41	3	1.5	5.16	57.6	2.638	0.768	4.5	5.1	0.71	2	3	1	2	2	0	2	2	20	6	157	55.7	22.59727	81.6	0.56
95037	1.11	3	1.71	4.77	84	3.491	2.687	6.44	5.5	8.17	2	2	1	2	2	0	2	2	20	1	163.9	70.5	26.24407	107.4	0.56
93870	3.79	4	1.6	5.33	48.6	2.25	0.406	4.03	5	0.3	2	3	1	2	2	0	1	2	20	3	158.1	47.4	18.96333	72	0.568
99125	5	4	1.5	5.22	28.5	2.043	0.802	3.9	4.9	0.63	2	3	1	2	2	0	1	2	20	6	148.6	48.8	22.09949	73.4	0.58
97507	3.59	4	1.4	5.3804	65.3904	1.684693	0.590635	2.9	4.9	0.63	2	3	2	2	2	0	1	2	20	2	154.9	51.9	21.6304	70	0.6
101688	3.4	3	1.09	5.1109	65.46768	1.991982	0.668696	3.39	5	1.07	2	3	2	2	2	0	2	2	20	3	161.1	52.7	20.30577	73.6	0.62
97671	0.66768	1	1.24	5.5386	107.892	2.3419	1.132333	4.01	5.4	8.69	2	1	2	2	2	0	2	2	20	2	145.9	70.8	33.26003	86.6	0.63
94453	1.77	4	1.14	5.72	94.08	2.302	1.242	4.01	5	2.18	2	3	1	2	2	0	1	2	20	3	166.4	85.8	30.98708	97.7	0.67
99761	0.80603	2	1.24	5.38	55.62	3.75	1.411	5.64	5.6	1.46	2	1	1	2	2	0	2	2	20	2	158.8	51.4	20.38272	79.6	0.68
98531	1.49	3	2.38	4.94	20.7	1.681	0.96	4.5	5.1	1.63	2	1	1	2	2	0	1	2	20	3	152.4	49.1	21.14032	67.7	0.714
95113	3.25	3	1.42	5.2702	72.876	2.018979	0.72888	3.65	5	2.65	2	3	2	2	2	0	2	2	20	1	160.7	84.3	32.64343	105.7	0.721
97522	0.82	4	1.4	5.0966	51.48	2.886733	0.840616	4.76	5.2	0.68	2	1	1	2	2	0	2	2	20	1	161.6	57.1	21.8652	75.7	0.73
93908	1.0339	3	1.4	6.72	118.2	2.121	0.463	3.72	6	0.5	2	1	1	2	2	0	1	2	20	6	162	71.1	27.09191	91.3	0.76
97249	1.14	4	1.06	5	48.9	1.552	0.655	2.92	5.3	2.3	2	1	1	2	2	0	2	2	20	4	162.4	84.5	32.03942	102.4	0.776
102015	1	3	1.27	5.22	82.26	1.19	0.756	2.79	5.6	0.91	2	1	1	2	2	0	1	2	20	3	160.8	67.9	26.26018	89.3	0.79
96663	0.08	3	1.37	5.16	38.28	2.327	0.576	3.96	5.3	0.67	1	1	1	2	2	3	2	2	20	3	163.4	57.4	21.49848	71.8	0.808
100029	1.7	3	1.55	5	58.62	2.302	0.745	4.19	5	4.46	2	3	1	2	2	0	1	2	20	1	154.8	54.3	22.6599	76.1	0.809
99459	1.08	3	1.03	5.2928	60.2676	3.46206	1.56792	5.51	5.1	1.14	2	1	1	2	2	1	1	2	20	2	162.1	109.5	41.67236	114	0.83
99931	1.6	2	1.29	5.4412	57.5616	2.43764	0.77627	4.22	4.8	1.9	2	1	1	2	2	0	2	2	20	6	155.4	58.1	24.05881	79	0.832
96093	0.21	3	0.93	4.77	34.26	2.508	0.598	3.72	5.2	39.53	2	1	1	2	2	0	2	2	20	3	156.8	95.9	39.00556	101.1	0.871
99721	1.46	4	1.45	5.11	112.44	2.974	0.406	4.6	5.2	2.66	2	3	1	2	2	0	1	2	20	4	174	74.8	24.70604	87.5	0.9
98788	2.72	4	0.98	4.77	161.46	3.207	1.377	4.81	5.2	1.28	2	1	1	2	2	0	2	2	20	1	167.7	106.7	37.94008	118.4	0.9
102903	2.061325	3	1.427222	5.27	60.3772	2.947681	1.017308	4.755582	5.3	3.29463	2	1	1	2	2	0	2	2	20	4	162.1	70.1	26.67792	87.4	0.924
100961	1.99	3	1.71	5	73.86	2.198	0.79	4.27	5	8.79	2	1	1	2	2	0	1	2	20	3	169.3	49.4	17.23507	69.9	1.02
99233	3.32	2	1.58	5.373981	56.3784	2.049545	0.655861	3.65	5.3	4.27	2	3	1	2	2	0	2	2	20	1	154.6	49.6	20.75213	81	1.18
98817	4.29	4	1.84	5.148453	41.93005	2.354542	0.54414	4.37	4.9	0.54	2	3	1	2	2	0	1	2	21	3	163.3	52.8	19.79983	73	0.325
98435	1.2	4	1.66	5.066402	35.283	2.06403	0.56874	4.06	5.2	0.24	2	3	1	2	2	0	1	2	21	4	157.9	61	24.46615	81.3	0.418
100775	1.03261	3	1.5	5.145613	30.8448	1.734696	0.515072	3.49	5.1	0.42	2	1	1	2	2	0	1	2	21	3	156.7	50.6	20.60689	75.6	0.43
99924	5	4	2.2	4.94	28.26	2.224	0.395	4.6	5	0.97	2	2	1	2	2	0	2	2	21	6	157	52.4	21.25847	67.3	0.44
98796	1.302	4	1.78	5.195142	34.8348	1.782380	0.49714	3.75	5.4	0.37	2	1	1	2	2	0	1	2	21	4	162.5	60.3	22.8355	76.2	0.472
99339	2.72	3	1.29	4.77	62.1	3.75	3.342	6.57	5.1	14.9	2	2	1	2	2	0	2	2	21	1	159.4	93.4	36.75955	108.6	0.559
96967	0.64	3	1.71	5.1556	33.2418	2.3082	0.60575	4.22	5.3	0.44	2	3	1	2	2	0	2	2	21	6	157.7	48.1	19.34112	72.8	0.574
1e+05	1.08	4	1.09	5.648066	97.5456	2.1189	0.9994	3.21	5.7	13.09	2	1	1	2	2	0	1	2	21	4	156	73.9	30.36654	92.7	0.606
99795	1.05	3	1.47	5.378366	94.7892	3.054775	1.143368	5.04	5.3	12.17	2	1	1	2	2	0	2	2	21	2	153.3	122.1	51.95548	127.4	0.689
98443	1.19	3	1.42	5.4167	57.4548	1.6877	0.641866	2.9	5.4	3.33	2	1	2	2	2	0	2	2	21	3	164.1	64.5	23.95204	83.6	0.74
96485	0.96	4	1.4	5.17896	54.216	2.022469	0.69778	3.75	5.1	1.41	2	3	2	2	2	0	2	2	21	3	154.8	75.3	31.42339	93.5	0.74
97702	3.4	4	1.37	5.2619	52.791	2.685985	0.67552	4.45	5.3	0.91	2	3	1	2	2	0	1	2	21	4	169.3	90.4	31.53948	103.7	0.74
95488	2.08	2	1.63	4.72	112.26	2.638	0.982	4.71	4.6	17.55	2	3	1	2	2	0	2	2	21	3	163	101.1	38.05186	126.8	0.804
94631	2.37	3	1.78	5.16	25.98	2.095	0.463	4.09	5.4	6.88	2	1	1	2	2	0	1	2	21	4	163.8	69.4	25.86614	79	0.86
96523	4.74	5	1.89	4.83	141.3	3.491	1.231	5.95	5.4	11.97	2	3	1	2	2	0	2	2	21	2	166.8	106.4	38.24279	115.7	0.86
100675	1.54	3	1.32	3.44	34.2	2.896	0.598	4.5	4.9	2.53	2	1	1	2	2	0	2	2	21	4	156.7	73	29.72931	104.2	0.868
93783	0.41	3	1.4	5.38	40.8	2.896	0.621	4.58	5.7	0.97	2	1	1	2	2	0	2	2	21	2	156.9	59.9	24.33218	82	0.87
96379	4.31	4	1.22	4.77	87.42	2.896	0.813	4.47	5	2.75	2	3	1	2	2	0	1	2	21	1	175.8	72.3	23.39379	86.1	0.877
95233	1.22	3	0.75	6.72	323.7	2.845	3.172	5.04	5.7	2.86	2	2	1	2	2	0	2	2	21	1	157.5	94.8	38.21618	108	0.9
101009	1.41	3	0.93	5.72	261.72	2.121	1.479	3.72	5.5	8.43	2	3	1	2	2	0	1	2	21	1	172.3	146.6	49.3814	143	0.93
99949	4.52	3	1.24	5.6226	70.42068	2.671775	0.95388	4.37	5.6	2.31	2	3	1	2	2	0	2	2	21	6	154.6	80.8	33.80589	99	0.94
102672	5	5	1.4	5.202607	71.41044	2.749928	0.94607	4.55	4.8	3.71	2	3	2	2	2	0	2	2	21	4	172.6	65.2	21.88598	80.1	0.958
98925	1.11	3	1.14	6.49	588	2.121	1.253	3.83	5.6	8.61	2	3	1	2	2	0	1	2	21	1	167.5	106.1	37.81689	114.4	0.983
95184	2.980266	4	1.55	5.5654	105.5844	2.678113	1.09618	4.65	4.8	33.53	2	3	1	2	2	0	1	2	21	3	158.4	73.8	29.41345	95.6	1.04
98589	2.41	4	1.09	5.90132	164.4717	2.51202	1.23674	4.09	5.4	18.92	2	3	1	2	2	0	1	2	21	1	165.2	144	52.76457	134.9	1.06
95973	1.39	2	1.27	5.615085	70.7544	2.446058	0.86695	4.14	5.5	2.05	2	1	1	2	2	0	2	2	21	2	154.5	60.7	25.42914	89.5	1.13

102093	0.12	2	1.4	6.22	132.48	2.612	0.666	4.32	5.4	28.05	2	1	1	2	2	0	2	2	21	2	155	69.6	28.96982	95.5	1.25
102037	1.24	3	1.55	5.55	59.7	1.862	0.519	3.65	5.1	44.49	2	1	2	2	2	0	1	2	21	1	157.8	53.8	21.6057	72.3	1.48
96860	4.62	5	1.24	5.27	35.04	3.465	0.982	5.15	5	9.59	2	3	1	2	2	0	2	2	22	3	163.3	74.2	27.82477	92.5	0.378
95211	3.83	4	1.73	5.026853	59.0868	2.544313	0.651486	4.5	4.6	3.85	2	3	1	2	2	0	2	2	22	1	151.8	45.6	19.78888	73.9	0.4
100026	2.55	4	0.83	6.49	180.24	2.508	4.358	5.33	5.9	5.74	2	2	1	2	1	0	2	2	22	7	161	75.4	29.08838	94	0.49
95699	1.79	4	1.29	4.55	8.28	1.371	0.463	2.87	5.4	0.49	2	1	1	2	2	0	1	2	22	4	160.8	64.7	25.02259	74.9	0.53
94306	1.73	4	1.91	5.07096	35.6808	2.0843	0.4964	4.01	5.1	0.47	2	3	1	2	2	0	1	2	22	3	170.2	53.7	18.53767	75.7	0.54
96497	1.81404	3	1.4	4.61	72.9	2.948	2.45	5.46	5.3	5.66	2	2	1	2	2	0	2	2	22	3	168	103.9	36.81264	125.8	0.6
101330	1.71	4	1.86	5.16	48.84	2.793	0.869	5.04	5.2	6.13	2	3	1	2	2	0	2	2	22	3	164.5	58.7	21.69233	76.7	0.603
94251	2.79	3	1.24	4.94	68.58	2.948	1.423	4.84	5.1	12.39	2	1	1	2	2	0	2	2	22	3	162	65.1	24.80567	81.9	0.604
100365	0.41	3	1.09	5.3459	59.0379	2.8844	0.97431	4.63	5	0.93	2	1	1	2	2	0	2	2	22	1	156.9	63.7	25.87579	85.3	0.648
94160	4.04	4	1.53	5.176	46.19423	2.938433	0.85275	4.97	5	1.07	2	3	1	2	2	0	2	2	22	3	176.7	72.1	23.09203	82.1	0.653
101790	2.17	3	1.22	5.712	121.6599	1.81778	0.89056	2.97	5.5	4.35	2	3	1	2	2	0	1	2	22	4	159.4	80.4	31.64313	110.4	0.672
100246	3.5019	5	1.55	5.145810	45.5748	2.000051	0.730578	3.65	5.2	5.54	2	3	1	2	2	0	2	2	22	6	155.6	44	18.17329	66.5	0.69
102283	3.35	4	1.16	5.33	64.8	3.155	0.542	4.58	5.4	0.33	2	1	1	2	2	0	2	2	22	3	158.5	78.6	31.28701	103.3	0.7
100438	1.95	4	1.29	5.0085	62.7576	2.124506	0.68095	3.9	4.7	1.11	2	3	2	2	2	0	1	2	22	1	159.7	60.5	23.72169	80.2	0.701
95522	0.33	4	1.68	5.080493	43.7796	2.595455	0.64668	4.6	5.2	1.03	2	3	1	2	2	0	1	2	22	3	164.6	76.5	28.23591	98.2	0.715
99401	3.4	5	1.86	4.66	26.64	2.56	0.463	4.63	5.4	1.15	2	2	1	2	2	0	2	2	22	6	176.9	62.7	20.03604	73.6	0.757
99151	1.24	4	1.4	4.993733	64.4664	1.924182	0.590458	3.44	4.9	1.77	2	3	2	2	2	0	2	2	22	3	162.2	69.2	26.30295	83.1	0.76
99396	1.52	4	1.03	5.38	183	3.077	1.163	4.65	5.7	1.2	2	1	1	2	2	0	2	2	22	1	159.7	84	32.93589	95.7	0.79
99436	1.58	4	1.6	5.94	148.86	4.707	0.971	6.75	5.7	5.88	2	1	1	2	2	0	1	2	22	4	173.5	100.8	33.48587	114.2	0.79
100202	1.2	4	1.66	5.27	77.16	1.991	0.61	3.93	4.7	2.17	2	3	2	2	2	0	1	2	22	1	156.4	85.7	35.03542	104.5	0.804
97709	2.01	3	1.32	5.22	55.68	1.371	0.418	2.87	5.5	1.38	2	3	1	2	2	0	1	2	22	3	160.3	84.2	32.76763	110.4	0.81
100148	0.890055	2	1.16	5.658666	68.01	2.024853	0.74808	3.44	5.5	0.33	2	1	1	2	2	0	1	2	22	2	170.6	61.2	21.02778	78.2	0.82
100068	1.037286	3	1.16	5.5	41.82	1.94	1.095	3.59	5.4	0.47	2	1	1	2	2	0	1	2	22	3	154.5	57.8	24.21424	90.6	0.828
101076	4.25	3	1.34	5.11	28.2	3.051	0.745	4.73	5.3	2.51	2	1	1	2	2	0	2	2	22	7	158.6	58.8	23.37604	78.5	0.92
101867	1.79	4	1.4	5.2094	72.1992	2.877668	1.019372	4.73	5	4.73	2	1	2	2	2	0	2	2	22	2	149.2	71.9	32.29916	88.8	0.92
100971	0	4	1.11	5.764666	132.0696	3.279705	1.59152	5.15	5.1	4.6	2	1	1	2	2	1	1	2	22	4	142.3	85.5	42.2237	118.9	0.97
95609	5	5	1.55	5.362566	87.9426	3.1502	1.05244	5.02	4.9	17.61	2	3	1	2	2	0	1	2	22	3	155	78.1	32.5078	104.2	0.99
97100	0.939429	4	1.06	5.5694	119.1612	3.93794	1.68348	5.9	5.7	4.26	2	1	1	2	2	0	1	2	22	3	171	115.8	39.60193	123	1.1
95955	1.47	3	1.24	5.72	84.48	2.198	0.44	3.65	5.2	5.17	2	1	1	2	2	0	1	2	22	3	164	102.1	37.96104	125.1	1.14
99198	0.753644	2	1.09	5.6798	84.11413	2.73026	1.076	4.27	5.4	4.05	2	1	1	2	2	0	2	2	22	1	162.5	76.2	28.8568	100.4	1.18
98442	1.99	3	0.88	5.16	162.36	6.025	1.129	7.42	5.1	3.93	2	1	1	2	2	0	2	2	22	4	163.7	94.5	35.26423	109.4	1.23
96839	5	4	0.91	6.11	269.28	2.896	0.79	4.16	5.6	10.21	2	3	1	2	2	0	1	2	22	7	171.6	132.2	44.89489	130.7	1.42
94219	3.496066	4	1.42	5.2802	72.243	2.251848	0.819546	4.03	4.9	2.71	2	3	2	2	2	0	1	2	23	1	155.6	55	22.71661	67.7	0.29
101709	2.55	4	2.33	4.44	70.38	2.664	1.942	5.87	4.3	4.5	2	3	1	2	2	0	2	2	23	2	169.3	83.5	29.13215	102	0.319
94211	2.97	5	1.71	5.256478	33.49234	1.956907	0.525173	3.93	5.1	0.28	2	3	1	2	2	0	2	2	23	3	162.8	49.4	18.63881	67.4	0.375
97402	0	5	1.58	5.354	86.0256	2.703133	0.77806	4.55	5.2	0.63	2	3	2	2	2	0	2	2	23	6	160.7	50.7	19.63253	69.2	0.48
94673	2.697769	4	1.22	5.11	55.08	2.405	0.779	3.98	4.6	0.43	2	3	1	2	2	0	1	2	23	4	165.3	60.8	22.25143	74.6	0.5
99583	1.07	5	1.34	5.073561	43.50062	1.905569	0.6214	3.65	5	0.32	2	3	2	2	2	0	2	2	23	3	158.7	51.7	20.52753	74	0.52
96619	0.96	3	2.3	5.18553	42.2904	2.89876	0.80574	5.3	5	0.23	2	1	1	2	2	2	2	2	23	3	172.9	44.3	14.81882	68.8	0.53
102202	2.29	4	1.5	5.2341	42.6378	2.326858	0.58418	4.03	5.2	0.72	2	3	1	2	2	0	2	2	23	7	164.2	57.6	21.36369	73.4	0.55
101025	1.24	5	1.97	5.38	47.58	2.819	0.914	5.2	5.3	2.1	2	3	1	2	2	0	2	2	23	6	166.9	68.2	24.4834	88.4	0.6
93999	4.98	4	1.53	5.33	73.68	1.655	0.316	3.34	4.9	0.61	2	3	2	2	2	0	1	2	23	4	166.7	69.2	24.90204	81.5	0.6
95200	0.81	3	1.27	5.169133	58.0956	1.957698	0.707315	3.65	5	0.89	2	1	2	2	2	0	1	2	23	1	153.3	58.9	25.06288	88.1	0.623
98851	3.95	4	1.32	5.3116	72.144	2.524630	0.743755	4.29	5.3	2.98	2	3	1	2	2	0	2	2	23	7	160.4	55.7	21.64943	71.5	0.63
102126	2.68	4	1.66	5.246447	41.4006	1.86762	0.582185	3.7	5.5	0.56	2	3	1	2	2	0	1	2	23	2	182.4	78.2	23.50483	86.1	0.64
95202	5	5	1.71	5.157683	38.80383	2.550561	0.96202	4.76	4.8	0.98	2	3	1	2	2	0	1	2	23	3	173.5	75.6	25.1144	95.6	0.648
94697	3.34	5	1.55	5.309983	37.7718	1.991713	0.496806	3.9	5.4	0.29	2	3	1	2	2	0	2	2	23	3	169.1	52.6	18.39495	72	0.65
97600	4.7	4	1.24	5.5178	84.4644	1.942068	1.13411	2.95	4.7	5.43	2	3	2	2	2	0	1	2	23	6	156.3	62.8	25.70643	84.7	0.657
98958	1.35	3	1.32	5.05	38.94	2.534	0.96	4.29	4.1	1.11	2	1	1	2	2	0	1	2	23	3	149	39.8	17.92712	72.4	0.69
101588	2.69	3	1.63	5.11	32.28	2.715	0.734	4.68	4.7	1.4	2	3	1	2	2	0	1	2	23	2	158.7	49.9	19.81284	74.5	0.758
96756	1.2	4	2.07	5.55	176.64	2.508	0.553	4.84	5	19.76	2	3	1	2	2	0	1	2	23	3	163.2	88.1	33.07772	99.4	0.76
97756	0.42	2	0.96	6.0618	131.2392	2.24732	1.1696	3.49	6	9.42	2	1	1	2	2	0	1	2	23	1	160.9	154.6	59.71692	143	0.775
96405	0.840290	2	1.09	5.673252	109.6066	2.988415	1.24884	4.78	5.4	2.66	2	1	1	2	2	0	2	2	23	2	147.9	64.8	29.62366	98.1	0.788
98295	4.34	4	1.29	5.1292	76.8888	2.782548	1.115295	4.84	4.7	2.86	2	3	1	2	2	0	1	2	23	1	157	66.8	27.10049	92.5	0.822
98073	0.16	1	1.81	5.253733	37.4686	1.870862	0.561728	3.54	5.2	1.43	2	1	1	2	2	0	2	2	23	2	143.9	40.4	19.51011	68.6	0.823
95239	0.48	3	1.11	6.66	90.78	2.974	2.055	5.02	6.4	2.65	2	1	1	1	3	0	1	2	23	1	155.9	74.6	30.69351	102.8	0.848
96103	2.360866	5	1.27	4.72	138	3.077	1.276	4.94	5.3	2.7	2	1	1	2	2	0	2	2	23	6	159.2	109.4	43.16495	108	0.863
101462	2.46	5	1.11	4.83	194.22	2.741	1.806	4.68	5.3	3.29	2	1	1	2	2	0	2	2	23	3	160.8	156	60.33267	143.2	0.937

101099	0.54	2	1.47	5.227133	56.5752	3.45826	1.15264	5.79	4.6	1.71	2	1	1	2	2	2	1	2	23	3	159.8	90.9	35.59675	116.8	0.94
95232	0.61	2	1.42	5.27	54.78	2.095	0.44	3.72	5.7	3.15	2	1	1	2	2	0	2	2	23	4	147.7	77.7	35.61722	105	0.995
99463	0.91	5	1.53	5.33	46.02	2.276	0.361	3.96	5.3	2.82	2	1	1	2	2	0	2	2	23	2	159.1	77.6	30.65641	93.6	1.02
95153	1.59	3	1.5	5.2101	88.1454	2.923375	1.1028	4.91	4.9	11.37	2	1	1	2	2	0	2	2	23	3	158.4	108.8	43.36292	121.7	1.13
97116	1.1	4	1.16	5.552466	119.778	3.77144	1.96894	5.9	5.5	10.88	2	1	1	2	2	0	1	2	23	1	159.2	76.9	30.34172	95.4	1.18
94250	3.37	5	1.42	5.38	97.68	2.483	0.61	4.19	5.1	7.07	2	3	2	2	2	0	2	2	23	3	166.7	117.7	42.35506	118.3	1.19
97575	2.57	5	1.94	5.1284	47.45476	2.553327	0.61462	4.65	5.4	1.68	2	3	1	2	2	0	2	2	24	3	169.6	53.8	18.70383	67.2	0.36
100089	3.91	4	1.32	5.22	86.04	1.629	0.237	3.05	4.8	0.3	2	3	2	2	2	0	1	2	24	3	154.5	50.1	20.98847	72.8	0.47
96229	0.87	1	1.01	5.2722	80.6091	2.12056	0.942116	3.41	4.9	1.2	2	1	2	2	2	0	2	2	24	3	147	38.2	17.67782	63.7	0.49
93902	0.806255	2	0.91	5.8316	83.5056	2.78872	1.31736	4.27	5.4	1.75	2	1	1	2	2	0	2	2	24	1	158.2	68.8	27.49005	92.4	0.5
97951	5	5	1.78	4.83	70.5	2.431	0.553	4.47	5.2	0.67	2	3	1	2	2	0	2	2	24	6	153	50.4	21.53018	69.5	0.51
94176	3.48	4	1.5	5.1949	49.37114	2.534137	0.63802	4.32	5.2	0.48	2	3	1	2	2	0	2	2	24	3	162.1	72.9	27.74352	90.4	0.548
98145	3.398885	5	1.37	5.3168	56.26131	1.8162	0.547503	2.77	4.7	0.57	2	3	2	2	2	0	2	2	24	3	172.6	65.3	21.91955	77.5	0.552
95445	0.787222	2	1.11	5.77	101.88	2.121	1.253	3.8	5	0.64	2	1	1	2	2	0	1	2	24	2	162.1	65.1	24.77507	87.2	0.59
93998	1.83	3	1.29	4.66	20.16	1.707	0.723	3.34	5.5	0.34	2	1	1	2	2	0	1	2	24	3	159.1	51.5	20.34543	78.6	0.616
100641	2.233283	2	1.84	7.55	228	2.069	0.756	4.24	4.8	0.42	2	3	1	2	2	0	2	2	24	6	160.5	49.1	19.06037	71	0.63
96031	2.43	4	1.55	5.5	20.4	1.862	0.485	3.65	5.4	3.76	2	3	1	2	2	0	1	2	24	2	170.9	68.2	23.35072	79.8	0.66
95703	2.8658	4	1.71	5.3262	45.6144	2.15416	0.57542	4.16	5.7	0.57	2	3	1	2	2	0	1	2	24	4	180.4	111.7	34.32259	104.4	0.68
98285	5	4	1.5	5.417466	62.30244	3.052496	0.99258	5.04	5.5	0.57	2	1	1	2	2	0	2	2	24	6	170.8	66.5	22.79533	86.2	0.692
96453	0.41	1	1.16	5.377	76.2324	2.60414	0.873978	4.29	4.7	0.68	2	1	1	2	2	0	2	2	24	2	150.8	51.5	22.64668	77.7	0.7
100090	3.645799	5	1.42	5.221581	69.16584	2.299350	0.78776	3.98	5.3	6.75	2	3	2	2	2	0	2	2	24	1	153.8	63.3	26.7603	86.1	0.709
96661	2.49	5	1.91	5.055028	39.961	2.674128	0.66902	4.91	4.7	0.96	2	3	1	2	2	0	2	2	24	6	164.9	53.9	19.822	73.8	0.737
98030	1.14	3	0.96	5.5	40.44	2.431	0.361	3.54	5.5	2.18	2	1	1	2	2	0	1	2	24	4	150.9	55.6	24.41723	78.3	0.74
102105	1.04	5	1.37	5.3217	81.57	2.39534	0.801321	4.06	5.7	2.37	2	1	1	2	2	0	2	2	24	4	165.8	111.6	40.59711	122.6	0.744
100269	1.14	4	1.19	5.4464	85.28963	3.017053	1.11656	4.86	5.1	0.57	2	2	1	2	2	0	2	2	24	3	163.3	76.4	28.64976	91.2	0.77
102589	0.6	2	1.89	5.313	36.0404	1.97616	0.49173	3.96	5.4	0.34	2	1	1	2	2	0	2	2	24	4	154.5	92.6	38.79306	105.1	0.86
99207	1.18	3	1.86	5.3176	50.95582	2.976377	0.78442	5.15	5.3	0.62	2	3	1	2	2	0	2	2	24	4	150.2	66.7	29.56555	86.8	0.895
94786	2.79	4	1.32	5.88	81.36	3.698	1.197	5.56	5.4	5.71	2	3	1	2	2	0	1	2	24	3	164.7	86.7	31.96185	102.3	0.9
97958	0.68	3	1.53	6.33	173.28	2.508	0.282	4.16	5.4	0.95	2	1	1	2	2	0	2	2	24	4	165	92.6	34.01286	107.5	0.9
102255	3.62	5	1.32	5.380333	70.1436	3.337225	1.151593	5.25	5.1	0.99	2	1	1	2	2	0	2	2	24	3	165.2	73.3	26.85863	92.3	0.986
94560	2.13	4	0.93	6.33	237.9	2.353	0.542	3.54	5.9	11.63	2	3	1	2	2	0	2	2	24	3	171.1	104.9	35.83236	125.7	1.09
94379	2.55	3	1.06	5.16	88.14	3.129	1.219	4.76	5.4	4.3	2	1	1	2	2	0	2	2	24	3	157.9	71.8	28.79786	101.5	1.18
101427	3.31	5	1.99	4.66	19.38	1.758	0.779	4.11	4.6	4.91	2	3	1	2	2	0	2	2	24	3	169	73	25.55933	91.8	1.24
96958	1.21	4	1.16	6.108333	158.052	2.503805	1.23808	4.16	5.8	29.12	2	1	1	2	2	0	1	2	24	4	163.5	104.5	39.09136	120.6	1.33
100965	0.78	4	1.84	4.27	36.6	2.069	0.316	4.06	4.9	0.37	2	3	1	2	2	0	1	2	25	4	159.2	45.4	17.91306	68.7	0.319
98416	0.66	5	1.68	4.83	13.44	2.121	0.44	4.01	5.2	0.33	2	3	1	2	2	0	2	2	25	6	159	52.4	20.72703	70.5	0.35
93746	3.4	5	1.84	5.3958	61.0546	2.67523	0.64352	4.53	5.7	4.44	2	3	1	2	2	0	2	2	25	4	158.4	62.1	24.75034	74.4	0.435
101733	1.46	4	1.27	5.11	53.46	1.655	0.576	3.18	5.4	0.38	2	1	1	2	2	0	1	2	25	7	158.1	56.6	22.64398	75.2	0.451
95799	1.63	4	2.59	4.94	36.36	2.457	0.384	5.22	5.2	0.46	2	3	1	2	2	0	2	2	25	3	168	69.9	24.76616	88.5	0.519
98982	2.06	4	1.97	4.11	37.44	1.629	0.418	3.78	5.2	0.45	2	3	1	2	2	0	1	2	25	4	158.4	53.8	21.44233	72.9	0.57
96913	4.78	5	1.71	5.257010	52.16962	2.95122	0.785046	4.99	5.2	4.9	2	3	1	2	2	0	1	2	25	6	149.8	58.5	26.06947	84.2	0.57
98494	5	5	1.5	5.55	61.8	2.379	0.531	4.11	5.6	0.39	2	3	1	2	2	0	2	2	25	7	165	64.2	23.58127	77	0.587
98445	2.982237	3	1.5	5.4168	66.8352	1.699689	0.54294	3.23	5	0.88	2	3	2	2	2	0	2	2	25	2	161.8	68.3	26.08937	84.4	0.62
102580	0.8421	3	1.42	5.098076	43.7124	1.875223	0.61161	3.62	5	0.39	2	1	2	2	2	0	1	2	25	1	153.5	67.3	28.56264	91.5	0.63
102184	2.39	4	1.73	5.14055	49.6368	2.793120	0.695988	4.84	5	3.93	2	3	1	2	2	0	2	2	25	6	151.5	87.5	38.12262	115	0.66
100258	2.77	2	1.16	5.4668	103.4544	2.778046	1.333693	4.5	4.9	8	2	1	1	2	2	0	2	2	25	3	159.5	88.4	34.74809	115.2	0.691
93782	4.65	4	0.96	5.27	60.48	2.483	0.373	3.62	5.3	0.46	2	3	1	2	2	0	1	2	25	4	164.7	65.1	23.99904	76.4	0.723
96966	1.44	4	1.24	5.55	21.36	2.069	0.858	3.7	5	4.94	2	3	1	2	2	0	1	2	25	3	160.8	67.9	26.26018	88.8	0.73
99089	3.81	5	1.5	5	34.32	2.508	0.294	4.14	4.7	1.76	2	3	1	2	2	0	1	2	25	4	158.2	61.4	24.53327	76.1	0.756
95351	5	5	1.11	5.562666	119.6604	3.48079	1.66959	5.51	5.3	5.35	2	3	1	2	2	0	1	2	25	6	155.2	76.8	31.88437	92	0.77
98256	2.77	3	1.6	5.38	45.6	3.129	0.519	4.97	4.8	1.22	2	3	1	2	2	0	2	2	25	4	163	75.7	28.49185	94.2	0.793
100672	1	4	1.01	5.3842	91.17384	3.757065	1.66814	5.72	5.3	3.23	2	1	1	2	2	0	1	2	25	1	156.9	75	30.466	97	0.802
94004	3.82	5	1.24	5.4212	103.0068	2.245953	1.04434	3.96	5.4	5.8	2	3	1	2	2	0	1	2	25	6	159.2	76.3	30.10498	101.6	0.814
102653	4.08	5	1.45	5.55	65.52	1.862	0.474	3.52	5.2	0.38	2	3	2	2	2	0	2	2	25	2	156.8	51.8	21.0687	81.2	0.82
101308	4.81	5	1.03	5.38	67.98	2.095	0.587	3.39	5.2	0.76	2	3	2	2	2	0	2	2	25	3	171.4	76.9	26.17609	85.6	0.83
98784	0.83	3	1.22	5.3358	115.4304	2.11958	1.127153	3.75	5.4	9.48	2	1	1	2	2	0	1	2	25	7	169.8	102	35.37731	113.2	0.864
94012	2.15	4	1.37	5.83	63.66	4.474	1.355	6.47	5.4	5.2	2	3	2	2	2	0	2	2	25	7	164.8	68.4	25.18498	92.5	0.93
94677	1.91	3	1.34	5.16	131.58	2.224	0.881	3.98	5.4	7.48	2	1	1	2	2	0	1	2	25	3	165.3	96.4	35.28022	106.1	0.982
96600	2.73	4	1.29	5.33	54.66	2.121	0.35	3.57	5.4	2.25	2	3	1	2	2	0	1	2	25	4	155.8	103.1	42.47406	111.1	0.99
99871	0.71	4	1.01	5.5624	135.2316	2.48768	1.52568	4.22	5.4	8.32	2	1	1	2	2	0	1	2	25	1	161.6	99.5	38.10135	119.6	1.01

96677	2.79	3	1.45	5.2678	69.2362	3.12653	1.035461	5.09	5.2	3.44	2	1	1	2	2	0	2	2	25	3	165.3	93.9	34.36528	107.3	1.04
102460	1.75	2	1.03	5.516652	109.741	2.7851	1.28138	4.45	5.5	4.45	2	1	1	2	2	0	2	2	25	3	168.9	60.7	21.27793	75.4	1.1
95501	0.81	3	1.22	5.6738	105.4848	2.62118	1.13244	4.32	5.5	6.97	2	1	1	2	2	0	2	2	25	3	159.8	98.3	38.49461	128.7	1.12
94936	1.02	4	1.55	5.3205	63.1092	3.128675	0.925251	5.15	5.5	2.18	2	1	1	2	2	0	2	2	25	4	156.5	87	35.52144	105.5	1.16
94430	2.2	4	0.78	7.3648	203.964	2.35632	1.7357	3.8	6.9	12.49	2	3	1	1	1	0	2	2	25	7	172.1	155.4	52.46737	142	1.27
98113	0.91	4	1.78	4.94	91.68	2.198	1.061	4.47	5.3	7.16	2	3	1	2	2	0	1	2	25	3	167	123.5	44.28269	139	1.28
95174	1.05	4	1.09	5.5229	86.738	2.28194	1.26986	3.83	5.2	17.11	2	1	1	2	2	0	1	2	25	7	158.2	73.7	29.44791	91.4	1.3
102638	3.32	5	1.66	5.185887	49.2204	2.547312	0.56088	4.5	5.1	0.57	2	3	1	2	2	0	2	2	26	3	168.7	63.6	22.3474	76.3	0.413
102272	3.29	4	1.47	4.88	22.32	1.474	0.869	3.34	4.8	0.44	2	3	1	2	2	0	1	2	26	3	169.6	74.4	25.86552	84.2	0.45
101289	1.579566	2	1.4	5.403052	65.3892	3.326655	1.3191	5.35	5.2	8.5	2	1	1	2	2	0	2	2	26	1	155.5	84.5	34.94588	109.8	0.47
97174	5	5	1.09	5.55	67.74	1.836	1.84	3.78	5.2	0.5	2	1	1	2	2	0	2	2	26	7	150.7	49.8	21.92819	77.7	0.56
97027	0.08	5	1.4	5.1246	56.2722	2.05178	0.71134	3.8	4.7	0.48	2	3	2	2	2	0	1	2	26	6	154.4	59.7	25.04262	82.5	0.56
94775	1.22	4	1.24	5.27	97.14	2.638	0.576	4.14	5	0.25	2	1	1	2	2	0	2	2	26	4	159.3	81.1	31.95871	100.5	0.566
98052	2.951841	4	1.68	5.2306	57.2556	2.989575	0.86124	5.15	5.4	1.7	2	3	1	2	2	0	2	2	26	4	163.9	69.7	25.94627	82	0.57
94434	2.43	4	1.34	5.149	44.36739	2.653737	0.66984	4.42	5.2	0.48	2	1	1	2	2	0	2	2	26	6	158.6	68.6	27.27205	90.7	0.59
99793	4.86	5	1.63	5.273	65.15571	2.313052	0.561462	4.19	4.8	0.74	2	3	2	2	2	0	2	2	26	3	164.3	78.2	28.96887	91.3	0.59
97041	5	4	1.76	5.05	67.62	1.577	0.282	3.47	5.2	1.3	2	3	1	2	2	0	1	2	26	4	160.9	84.7	32.71684	93.9	0.627
95586	0.96	4	1.47	5.274356	42.0672	1.886687	0.64959	3.8	5.4	0.71	2	1	1	2	2	0	1	2	26	4	161.2	51	19.62638	68.5	0.635
97057	4.78	5	1.68	4.94	26.76	3.982	1.027	6.13	5.1	1.73	2	3	1	2	2	0	2	2	26	6	160.2	57.2	22.288	80.1	0.67
101164	3.25	5	1.89	5.271613	43.009	2.337987	0.53643	4.32	5.5	0.55	2	3	1	2	2	0	2	2	26	6	158.8	51.4	20.38272	71.8	0.687
99400	1.95	3	1.47	5.4466	77.5272	2.800651	1.060028	4.68	5.5	5.01	2	1	1	2	2	0	2	2	26	1	152	74.3	32.15893	100.5	0.687
102383	0.58	3	1.55	4.61	26.04	2.819	0.271	4.5	5.5	1.18	2	1	1	2	2	1	2	2	26	1	150.4	55.7	24.62405	80.3	0.71
97304	0.59	3	0.91	5.88	81	2.793	2.179	4.71	5.5	2.27	2	1	1	2	2	0	2	2	26	1	161.1	95.6	36.83552	114.7	0.72
94916	0.64	3	1.27	5.3328	94.2856	2.187893	0.95852	3.9	5.2	11.77	2	1	2	2	2	0	1	2	26	4	161.1	86	33.13656	91.5	0.726
99525	1.8396	4	1.37	5.261493	52.6348	1.720591	0.632128	3.18	5.3	0.85	2	1	1	2	2	0	2	2	26	3	171.8	82.6	27.98554	94	0.73
96325	2.38	4	1.22	5	67.86	2.612	0.632	4.11	5.3	2.28	2	1	1	2	2	0	2	2	26	7	148.2	73.1	33.28289	94.2	0.788
95793	0	2	1.42	5.22	52.44	3.026	1.332	5.07	5.3	0.9	2	1	1	2	2	2	2	2	26	1	150.7	42.4	18.66979	72.6	0.8
94200	3.59	4	1.4	5.3565	75.453	2.031598	0.689516	3.47	5	4.71	2	3	2	2	2	0	1	2	26	2	155.4	91.3	37.8067	97.9	0.81
98921	2.06	5	1.16	4.94	168.06	1.629	2.823	4.09	5.296884	1.98	2	1	1	2	2	0	2	2	26	3	164	104.8	38.9649	103.9	0.815
102380	1.62	3	1.32	5.405166	78.5184	3.301795	1.212808	5.28	5.3	5.11	2	1	1	2	2	0	2	2	26	4	164.1	78.7	29.22521	88.8	0.829
97011	5	4	1.11	5.16	65.22	2.483	0.598	3.88	4.6	0.85	2	3	1	2	2	0	1	2	26	3	157.7	71.1	28.58948	90.2	0.85
98222	3.87	5	1.94	5.185343	34.362	2.153842	0.709818	4.16	5.2	4.43	2	3	1	2	2	0	2	2	26	3	167.2	63.7	22.78594	76	0.87
94463	1.203	3	1.27	5.83	97.98	1.94	0.914	3.62	6.1	0.76	2	1	1	2	2	0	1	2	26	4	162.1	111.9	42.58573	117.2	0.89
101701	3.65	5	1.34	5.2658	67.04563	2.028773	0.865826	3.7	5.3	4.94	2	3	1	2	2	0	1	2	26	2	156.5	69.5	28.37632	88.8	0.95
93755	2.07	4	1.24	6.11	122.46	3.129	0.711	4.71	5.7	4.86	2	1	1	2	2	0	2	2	26	4	178.5	93.8	29.43923	94.2	0.964
102113	2.566293	4	1.71	5.2258	62.6208	1.958618	0.569671	3.7	5	2.89	2	3	2	2	2	0	1	2	26	1	160.3	75.5	29.3819	88.5	0.98
95040	2.43	4	1.84	5.598948	96.786	4.18836	1.277445	6.59	5.4	19.4	2	3	1	2	2	0	1	2	26	3	168.5	143.6	50.57718	138.3	1.05
100160	1.5	3	1.22	5.88	67.56	1.629	0.689	3.15	5.7	3.8	2	3	1	2	2	0	2	2	26	3	150.6	75.8	33.42099	103.5	1.08
98483	0.61	4	1.16	5.5	54.18	1.707	0.666	3.18	5.2	1.16	2	1	1	2	2	0	2	2	26	3	157.9	51.6	20.69595	77.1	1.12
99005	1.82	3	0.98	4.77	191.4	3.465	0.892	4.86	5.6	9.72	2	1	1	2	2	0	2	2	26	4	168.1	108.1	38.25518	115.3	1.3
99887	5	4	1.91	6.27	45.6	2.715	0.734	4.97	5.5	3.1	2	3	1	2	2	0	2	2	27	3	170.7	76.1	26.11666	90.4	0.34
97325	1.54	3	1.73	5.0607	42.1164	2.885409	0.88914	5.15	4.9	1.01	2	3	1	2	2	0	2	2	27	1	157.9	60.8	24.38593	79.8	0.502
97278	0.85	4	1.42	5.638085	80.8884	2.968433	0.86272	4.78	5.9	0.44	2	1	1	2	2	0	2	2	27	4	153.1	74.8	31.91179	98.1	0.53
94795	5	4	2.09	5.252104	45.75008	2.464291	0.498187	4.6	5.3	0.49	2	3	1	2	2	0	2	2	27	1	160	53.8	21.01563	72.2	0.55
98010	1.5	5	1.6	5.15436	42.7696	1.9964	0.647853	3.9	5.4	1.02	2	3	1	2	2	0	2	2	27	6	152.9	52	22.24274	80.4	0.577
97183	4.12	5	1.66	5.393613	54.95868	2.531165	0.61936	4.37	5.5	2.674933	2	3	1	2	2	0	2	2	27	2	163	66.8	25.14208	79	0.58
94221	0	4	1.5	5.627	79.5222	3.587553	1.07214	5.56	5.1	0.63	2	2	2	2	2	0	2	2	27	4	154	56.1	23.65492	71.5	0.599
102386	1.6	4	1.5	5.056941	45.094	2.69612	0.663373	4.65	5.3	1.14	2	3	1	2	2	0	2	2	27	6	153.8	74.8	31.62197	99	0.62
96063	0.58	4	2.56	5.0436	36.8124	2.601025	0.53202	4.86	5.1	0.39	2	1	1	2	2	0	2	2	27	6	161.6	52.1	19.95056	74.3	0.621
99751	3.04	4	1.5	5.1444	69.4032	2.54095	0.628486	4.34	4.8	1.18	2	3	2	2	2	0	1	2	27	3	164	90.8	33.75967	99.7	0.66
96590	0.59	2	1.24	5.83	79.5	3.051	0.542	4.55	5.4	2.08	2	1	1	2	2	0	2	2	27	1	153.6	96.4	40.85965	115.2	0.665
100577	0.14	3	1.68	5.18935	31.69050	1.71188	0.52082	3.15	5.4	0.56	2	1	1	2	2	1	1	2	27	4	162.2	58.3	22.15985	74.1	0.72
94291	1.71	3	1.4	5.379933	54.7864	1.954811	0.7757	3.54	5.2	1.09	2	1	1	2	2	0	2	2	27	1	147.2	60.2	27.78311	85	0.724
97231	2.94	4	0.96	6.49	489.18	2.974	2.066	4.89	5.9	4.25	2	3	1	2	2	0	2	2	27	6	155.5	75	31.01705	98	0.776
100032	5	5	1.71	5.5	27.12	2.638	0.542	4.6	5.2	1.47	2	2	1	2	2	0	2	2	27	6	152.5	48	20.63961	70.6	0.78
97293	1.2	5	1.53	5.72	45.6	3.181	1.366	5.33	5.6	6.2	2	1	1	2	2	0	2	2	27	1	153	66.2	28.27972	91.7	0.78
100108	4.86	5	1.63	5.249742	36.942	1.975983	0.541186	3.8	5.1	0.35	2	3	1	2	2	0	1	2	27	3	173.8	71.5	23.67045	81.4	0.78
102149	0	2	1.6	5.38	306.12	3.077	0.508	4.91	5.2	1.81	2	1	1	2	2	0	2	2	27	4	158	81.7	32.72713	113.6	0.81
100299	2.6	4	1.29	4.83	58.56	5.275	0.79	6.93	5.4	4.96	2	1	1	2	2	0	2	2	27	4	165.7	112.1	40.82823	123	0.839
99577	5	5	1.99	5.314966	84.699	3.51084	1.116566	5.87	5.4	4.83	2	3	1	2	2	0	2	2	27	3	166.1	84.2	30.51918	90.3	0.852

99879	5	3	1.29	5.3547	81.85288	2.439890	0.937646	4.19	5.1	4.1	2	1	1	2	2	0	2	2	27	4	159	49.7	19.65903	71.6	0.863
94569	4.04	4	1.45	4.83	52.98	2.638	0.869	4.47	4.7	0.42	2	3	1	2	2	0	1	2	27	3	152.7	58.7	25.17445	84	0.868
94130	3.3732	4	2.15	5.33	25.14	2.302	0.463	4.65	5.3	0.95	2	2	1	2	2	0	2	2	27	4	163.7	70.9	26.4575	95.4	0.882
97450	3.98	5	1.94	5.44	59.82	2.353	0.237	4.4	5.7	13.46	2	3	1	2	2	0	1	2	27	4	165.1	119	43.65689	95.7	0.913
98420	5	5	2.51	5.05	12.84	2.896	0.474	5.61	5	0.51	2	2	1	2	2	0	2	2	27	3	171.2	58.5	19.95944	73.9	0.946
100095	4.08	5	1.45	5.44	97.56	2.198	0.768	4.01	5.3	2.38	2	3	2	2	2	0	1	2	27	2	167.6	94.4	33.60655	115.4	0.98
99038	0.18	1	1.4	5.384552	58.4364	2.81932	0.937472	4.65	5.1	5.28	2	1	1	2	2	0	2	2	27	4	165	92.1	33.8292	104	1.02
97670	3.05	4	1.29	5.3906	77.802	3.097586	1.117066	4.97	5.3	5.24	2	1	1	2	2	0	2	2	27	7	167	89.1	31.94808	99.5	1.04
98460	0	2	1.27	5.437652	110.6556	2.973915	1.129913	4.78	5.3	6.36	2	1	1	2	2	0	2	2	27	1	158.6	82.4	32.75826	100.5	1.13
95850	3.25	4	0.62	6.1596	199.5278	2.750530	2.171355	4.73	6.1	3.99	2	3	1	2	2	0	2	2	27	4	163.4	107.9	40.41265	120.5	1.3
102348	1.7	3	1.03	5.33235	85.1724	2.3688	1.127108	4.19	5.2	8.7	2	1	1	2	2	0	2	2	27	1	158.8	109.4	43.38267	113.2	1.37
99318	0.73	4	2.22	5.11	36.48	2.121	0.892	4.76	5.2	2.69	2	3	1	2	2	0	1	2	28	4	173.6	64.5	21.40229	76	0.369
100254	3.572326	5	1.5	5.61	40.86	2.689	0.734	4.53	5.3	0.46	2	3	1	2	2	0	2	2	28	4	169.7	72.4	25.14056	84.3	0.374
96865	1.13	4	1.55	5.16	18.3	1.733	0.373	3.47	5.5	0.29	2	1	1	2	2	1	1	2	28	4	172.5	67.4	22.6507	78.5	0.4
96774	2.69	5	2.09	5.11	26.22	3.026	0.406	5.3	5	0.22	2	2	1	2	2	0	1	2	28	6	153.9	52.3	22.08129	84	0.455
101799	5	5	1.89	5.22	17.52	2.379	0.384	4.45	5.1	0.37	2	2	1	2	2	0	2	2	28	3	168.7	47	16.51459	64.6	0.46
99197	1.19	3	1.29	4.83	25.86	3.129	1.513	5.12	5.1	0.59	2	1	1	2	2	0	2	2	28	2	154.7	54	22.56385	81.8	0.48
100440	1.410277	3	1.27	5.3266	59.9016	2.65534	0.79058	4.34	5.1	0.73	2	1	1	2	2	0	2	2	28	1	154.3	61.3	25.74712	86.7	0.48
94446	0.73	2	1.32	4.77	54.48	3.931	0.869	5.64	5.3	2.1	2	1	1	2	2	3	2	2	28	2	165.7	87.4	31.83218	115	0.53
102548	1.2	5	1.27	5.88	44.34	2.586	1.569	4.58	5.2	18.22	2	1	1	2	2	0	1	2	28	7	164.7	88.9	32.77288	104	0.549
101166	1.69	5	1.71	4.83	18.18	2.353	0.666	4.37	5	1.02	2	2	1	2	2	0	2	2	28	3	164.7	49.8	18.35871	69.4	0.57
100619	2.88	5	1.86	5.2415	49.66342	2.59535	0.64354	4.5	5.4	5.29	2	3	1	2	2	0	2	2	28	3	170.6	87	29.89243	103.2	0.57
100070	3.04	3	1.5	5.306728	45.21734	2.816415	0.69142	4.65	4.9	0.33	2	3	1	2	2	0	2	2	28	3	162.2	59.2	22.50194	82.4	0.62
100171	1.36	4	0.59	5.11	309.66	2.28804	5.205	3.13	4.9	5.14	2	1	2	2	2	0	1	2	28	1	158.2	70.7	28.24922	102.6	0.62
97941	4.34	5	1.63	5.164448	53.976	2.554087	0.63486	4.42	5.3	2.56	2	3	1	2	2	0	2	2	28	1	158.8	69.8	27.67926	86.3	0.63
97036	4.98	5	1.71	5.135483	63.31035	2.201160	0.729702	4.16	4.7	2.941378	2	3	2	2	2	0	2	2	28	4	177.1	71.4	22.76466	83.9	0.64
97945	2.19	4	1.03	5	41.46	1.758	0.937	3.23	5	2.34	2	3	1	2	2	0	1	2	28	1	146.2	65.3	30.55051	91.4	0.67
95190	1.66	4	1.84	5.33	14.34	1.862	0.395	3.88	5.2	2	2	3	1	2	2	0	1	2	28	1	155.9	46.1	18.96744	70.2	0.672
102461	3.69	5	1.4	6.38	48	1.707	0.576	3.36	5.3	0.98	2	3	1	2	2	0	2	2	28	6	148.8	54.3	24.52415	79.7	0.69
94783	1.5	4	1.19	5.517613	90.71453	2.6672	1.101911	4.37	5.5	1.06	2	1	1	2	2	0	2	2	28	6	157.8	58.7	23.5735	83.2	0.707
97469	0.18	3	1.5	5.181	43.4688	2.888653	0.76614	4.6	5.4	0.89	2	1	1	2	2	3	2	2	28	4	156.3	62	25.37895	81.2	0.708
96548	2.62	4	1.22	6.27	75.54	2.638	0.869	4.24	5.1	0.74	2	3	1	2	2	0	2	2	28	1	171.5	70.8	24.0716	94.3	0.71
96125	3.69	5	1.63	5.3357	64.2216	2.568138	0.59864	4.45	5	0.68	2	3	2	2	2	0	2	2	28	3	165.9	61.1	22.19977	74.3	0.722
98169	0.85	4	1.6	5.279928	58.7148	3.211982	1.04424	5.4	5.1	3.93	2	3	1	2	2	0	1	2	28	2	152.7	75	32.16497	102.8	0.726
98571	2.511084	4	1.84	4.88	58.02	3.103	0.463	5.15	5	1	2	3	1	2	2	0	1	2	28	4	174.5	91.6	30.08185	100.3	0.729
95543	5	5	1.24	5.7544	61.6826	3.083876	1.0273	4.86	5.6	0.91	2	3	1	2	2	0	2	2	28	6	157.7	60.1	24.16635	78	0.742
94927	1.896841	5	0.91	5.05	247.74	2.715	1.423	4.27	5.6	2.44	2	1	1	2	2	0	2	2	28	6	154.4	101.9	42.74444	108.4	0.754
102324	5	5	1.11	6.107556	168.9396	2.740353	1.58196	4.6	5.6	4.79	2	3	1	2	2	0	2	2	28	1	177.2	90.6	28.85365	97.3	0.81
98509	0.72	3	1.01	13.06209	164.3028	4.323974	2.0009	6.34	11.3	3.41	2	1	1	1	1	0	2	2	28	3	180.6	109.7	33.63343	119.4	0.81
95788	4.25	5	1.81	5.214495	45.28143	2.65941	0.741895	4.78	5.2	1.9	2	3	1	2	2	0	2	2	28	3	163.9	72.9	27.13749	91.2	0.82
101669	0.51	2	0.98	5.33	138.12	3.077	1.242	4.63	5	9.8	2	1	1	2	2	0	2	2	28	3	153.5	99.8	42.35589	125.5	0.84
98298	3.38	5	1.32	5.33	114.42	1.5	0.914	3.23	5.4	6.03	2	3	2	2	2	0	2	2	28	7	158.8	111.3	44.13612	124.1	0.91
95990	3.28	4	1.66	5.55	40.98	2.302	0.734	4.29	4.7	12.72	2	3	1	2	2	0	2	2	28	3	167.5	59.9	21.34997	79.6	0.974
95186	4.86	5	1.22	5.8522	81.29596	2.906568	0.98086	4.58	5.9	2.35	2	3	1	2	2	0	2	2	28	6	158.4	68.3	27.22139	95.8	0.99
94416	1.96	3	0.88	5.3338	113.946	3.314155	1.304	5.17	5.1	8.56	2	1	1	2	2	0	2	2	28	3	177.7	105.4	33.37842	119.7	1
99768	4.45	5	1.01	10.0234	171.6144	3.5656	2.00104	5.61	7.9	5.54	2	3	1	1	1	0	2	2	28	3	166.4	120	43.33857	131.2	1.01
95393	5	5	1.4	5.5	162	4.965	1.592	7.09	5.6	15.23	2	3	1	2	2	0	2	2	28	6	154.6	81.8	34.22428	89.3	1.05
98971	0	4	1.58	5.6546	59.76853	3.086468	0.77278	4.94	5.5	0.88	2	3	1	2	2	0	2	2	28	4	155.6	79.5	32.83583	102.6	1.06
95385	3.82	5	1.66	5.165381	44.9736	2.346802	0.717752	4.27	5.3	6.65	2	3	1	2	2	0	2	2	28	6	168.3	68.9	24.3249	97	1.11
101693	2.11	5	1.06	6.11	170.58	3.31	3.613	6.03	5.6	2.57	2	2	1	2	2	0	2	2	28	3	157.5	85.5	34.46712	108.9	1.12
101896	1.42	3	1.09	8.1	83.1	2.715	0.644	4.09	6.3	3.93	2	3	1	1	1	0	2	2	28	3	161.2	94.9	36.52045	106.5	1.19
93845	1.93	3	1.4	5.171114	54.8604	2.86012	0.919968	4.63	5.2	1.24	2	1	1	2	2	0	2	2	28	3	165.3	67.9	24.84987	89.9	1.26
96392	3.52	3	1.24	5	144.72	3.077	2.055	5.25	5	16.69	2	1	1	2	2	0	2	2	28	1	164.1	81.6	30.30212	97.5	1.33
100896	0.31	2	1.14	4.94	67.86	3.569	1.49	5.38	5.6	9.15	2	1	1	2	2	0	2	2	28	3	160.8	95.8	37.05044	118.5	1.52
100583	1.2	1	1.94	5.21596	43.64116	2.513582	0.638809	4.5	5.1	0.91	2	3	1	2	2	0	2	2	29	1	151.7	63.8	27.72359	85.7	0.372
96198	0.73	5	1.24	5.22	33.66	1.965	0.485	3.44	5.6	0.32	2	3	1	2	2	0	2	2	29	4	161.5	51.4	19.70689	69.5	0.373
98065	0.81	4	1.86	5.021902	32.0022	2.1453	0.58148	4.29	5.1	0.3	2	3	1	2	2	0	1	2	29	7	160.3	51.2	19.92521	71.6	0.396
93846	0.87	4	1.68	5.356066	37.1494	1.527809	0.493886	2.95	5.8	0.27	2	1	1	2	2	0	1	2	29	4	165.9	54.6	19.83809	68	0.441
97691	0.17	4	1.22	5.38	62.22	3	0.982	4.65	6	2.77785	2	1	1	2	2	0	2	2	29	4	163	84.3	31.72871	102	0.454
101620	5	4	1.86	5.176114	35.6244	2.08377	0.55158	3.98	5.1	0.46	2	3	1	2	2	0	1	2	29	6	160.8	53.4	20.65234	76	0.531

97716	1.17	2	1.42	5.3442	52.236	2.6682	0.73872	4.34	4.9	1.27	2	1	1	2	2	0	2	2	29	3	170.2	65.9	22.74921	79.2	0.609
96313	4.81	3	1.58	5.3962	79.074	3.90856	1.374087	6.15	5.2	7.66	2	3	2	2	2	0	2	2	29	6	155.1	56.9	23.65313	89.3	0.61
96243	3.94	4	2.33	5.216014	54.9876	4.622435	1.823425	10.76	4.8	3.61	2	3	1	2	2	0	2	2	29	3	160.6	67.9	26.32563	91.7	0.629
99062	2.9487	4	1.58	5.2608	78.336	3.88372	1.274889	6.23	4.9	4.51	2	3	2	2	2	0	2	2	29	2	144.8	57	27.18553	90.2	0.64
101372	2.43	3	1.55	5.88	34.98	1.396	0.756	3.28	5.2	0.79	2	3	1	2	2	0	2	2	29	6	156.3	58.1	23.78254	80.8	0.673
97122	1.7	4	1.47	5.0324	56.1252	2.809299	0.82164	4.81	5	0.96	2	3	1	2	2	0	1	2	29	4	167.8	110.2	39.13791	118.8	0.769
99446	2.85	5	2.33	4.72	13.56	3.026	0.305	5.48	5.3	1.14	2	2	1	2	2	0	2	2	29	3	167.4	71	25.33655	86.9	0.803
100999	2.41	5	1.24	5.1568	95.4147	2.88805	1.210756	4.78	4.7	1.48	2	1	1	2	2	0	2	2	29	3	166.4	91.1	32.9012	103.8	0.83
100428	2.89	4	1.78	5.351066	55.39168	3.384316	0.763496	5.43	5.5	3.54	2	3	1	2	2	0	2	2	29	3	149.8	68	30.30298	91.8	0.832
99466	1.37	1	1.45	5.66	66.72	2.922	1.332	4.99	5.6	3.99	2	1	1	2	2	0	2	2	29	2	146.4	90.1	42.03806	113.9	0.865
97356	1.25	3	1.19	5.3263	101.4708	2.4029	1.231601	4.16	5	7.3	2	1	2	2	2	0	2	2	29	1	151.1	81.5	35.69675	108.5	0.89
97217	0.35	2	2.04	4.61	30.78	3.233	0.44	5.48	4.7	3.13	2	3	1	2	2	0	2	2	29	4	161.7	60	22.94728	84.4	0.912
100117	0.09	4	1.16	5.646066	117.5544	3.781385	1.8466	5.72	5.4	5.13	2	1	1	2	2	0	1	2	29	6	154.9	64.6	26.92339	87.2	0.912
93980	5	5	1.6	5.16	42.54	2.586	1.264	4.76	4.8	0.67	2	3	1	2	2	0	1	2	29	3	173.8	58	19.2012	76.7	0.921
101481	1.82	4	1.68	5.6041	97.0536	3.73632	1.10998	5.9	5.6	7	2	3	1	2	2	0	1	2	29	3	149.7	60.4	26.95215	91.2	1
97986	2.89	4	0.8	4.77	96.9	1.629	1.761	3.23	5.7	5.96	2	1	1	2	2	0	1	2	29	4	165.6	121.3	44.23236	118.8	1.02
98322	0.8	3	0.83	5.88	127.2	1.733	0.914	2.97	5.1	4.56	2	1	2	2	2	0	1	2	29	3	164.5	114.7	42.3869	124.4	1.07
94672	5	5	1.37	5.424711	92.73888	4.15982	1.369973	6.49	5.5	2.39	2	3	2	2	2	0	2	2	29	3	157.4	88.5	35.72184	107.8	1.08
99526	5	5	1.06	5.88	171.42	1.655	1.185	3.26	6.1	6.27	2	1	1	2	2	0	2	2	29	6	153.2	97.7	41.62718	120	1.08
94924	1.2	4	1.06	5.425233	86.5488	3.43588	1.39267	5.3	5.3	5.27	2	1	1	2	2	0	2	2	29	3	158.1	79.3	31.72557	110	1.14
95587	3.67	4	1.29	6.029711	144.1032	2.85706	1.25068	4.81	6	7.44	2	3	1	2	2	0	1	2	29	3	170.6	104.4	35.87091	125.8	1.15
102456	1.8	3	1.22	6.55	354.6	2.948	1.468	4.84	6.6	11.41	2	3	1	1	2	0	2	2	29	7	156.6	104.8	42.73442	128.5	1.17
102088	1.21	4	1.45	5.761	91.8945	3.265225	1.59	5.33	5.5	8.95	2	2	1	2	2	0	1	2	29	3	165.9	87.3	31.71914	117.2	1.27
101446	3.73	5	1.53	5.11	28.62	3.129	1.14	5.17	5.2	1.34	2	3	1	2	2	0	1	2	30	7	173.6	57.5	19.07956	74.3	0.358
98328	3.335971	5	1.09	5	65.16	2.224	0.361	3.47	4.9	1.56	2	3	2	2	2	0	2	2	30	6	157.2	64.4	26.06038	93.5	0.39
99861	2.65	5	1.71	5.086421	41.8372	2.809162	0.71242	4.84	5.1	0.7	2	3	1	2	2	0	2	2	30	6	164.6	56.2	20.74324	68.9	0.42
94663	5	5	1.63	5	27.3	2.327	0.677	4.27	5.2	0.49	2	2	1	2	2	0	2	2	30	7	160.2	59	22.98937	80.9	0.45
98729	5	4	3.03	4.72	56.82	0.828	0.847	4.24	5.4	4.53	2	1	1	2	2	0	1	2	30	6	149.2	44.5	19.99044	69.8	0.46
101516	5	5	1.99	5.38	20.52	1.81	0.327	3.96	4.8	0.71	2	3	1	2	2	0	2	2	30	3	166.8	65.4	23.50637	83.3	0.49
100855	0.61	3	1.03	5.32198	60.4083	2.954893	1.20214	4.84	5	1.01	2	1	1	2	2	0	2	2	30	3	167.1	63.5	22.74159	83.6	0.509
101501	5	5	1.55	5.3552	64.5378	2.871409	0.85956	4.81	5	0.68	2	3	2	2	2	0	2	2	30	6	156.6	66.1	26.95367	88.2	0.545
97044	4.588614	5	1.6	5.27	28.62	2.845	0.734	4.78	5.3	0.16	2	3	1	2	2	0	2	2	30	6	155	39.6	16.48283	70.5	0.546
97949	3.45	4	1.94	5.3284	58.5516	2.76184	0.66268	4.73	5	0.66	2	3	1	2	2	0	2	2	30	3	161.8	58.7	22.42235	73.5	0.593
98527	0.02	2	1.53	5.6836	82.4628	2.7406	0.696715	4.4	5.4	0.53	2	1	1	2	2	0	2	2	30	4	149.9	44.8	19.93769	74	0.6
101570	0.99	5	1.53	5	101.16	0.983	0.26	2.64	4.8	1.9	2	3	2	2	2	0	2	2	30	4	166.6	63.4	22.84227	77.7	0.62
99910	1.75	4	1.27	5.1458	59.8407	3.0075	0.79256	4.68	5	0.97	2	1	1	2	2	0	2	2	30	3	156.4	56.4	23.05715	74.3	0.621
94577	5	5	1.73	5.77	26.22	5.793	0.531	7.76	4.8	0.39	2	2	1	2	2	0	1	2	30	3	175.9	60.7	19.6181	81.6	0.627
93965	4.25	5	1.86	4.88	26.82	3.801	0.587	5.92	5.2	3.65	2	3	1	2	2	0	2	2	30	2	159.5	57.5	22.60198	78	0.638
96679	1.89	4	1.16	5.245	72.6327	2.9279	1.12582	4.65	5.1	0.34	2	1	1	2	2	0	2	2	30	3	163.6	51.2	19.12949	77	0.645
99194	2.16	4	1.71	5.3396	56.26152	2.22716	0.61862	3.98	6	3.7	2	3	1	2	2	0	1	2	30	4	154.5	75.9	31.7969	98.7	0.672
94375	3.18	4	1.84	5.33	28.98	3.75	0.689	5.9	5.1	5.12	2	3	1	2	2	0	2	2	30	7	159.7	55.4	21.72201	77.5	0.686
100234	2.71	5	1.45	5.215128	46.4226	2.813106	0.68172	4.55	5.2	1.14	2	3	1	2	2	0	2	2	30	6	151.2	49.2	21.52095	74.3	0.696
98048	3.4	5	1.76478	4.94	45.8484	2.506907	0.54804	4.6149	5.2	1.086006	2	3	1	2	2	0	2	2	30	6	159.4	53.2	20.93799	75.4	0.74
101838	1.22	4	1.34	11.1448	135.6792	3.247026	1.52916	5.2	9.4	2.93	2	3	1	1	1	0	1	2	30	3	157.2	67.2	27.19344	94.6	0.753
101134	4.273586	5	1.86	5.217	61.4574	2.916173	0.839246	5.17	5	12.06	2	3	1	2	2	0	2	2	30	3	161.4	83.9	32.20734	96.3	0.8
98687	2.031750	4	1.6	4.86745	47.396	3.018325	0.843816	5.12	5.1	1.1	2	3	1	2	2	0	2	2	30	3	159	66.6	26.34389	85.6	0.816
94015	1.18	2	1.06	5.38	60.66	3.077	0.519	4.37	4.8	1.23	2	1	1	2	2	0	2	2	30	3	159.8	67	26.23743	91.6	0.85
99710	1.43	4	1.58	5.22	13.44	1.422	0.508	3.23	5.5	1.49	2	1	1	2	2	0	1	2	30	7	163.3	58.2	21.82482	76.9	0.89
100087	2.43	4	0.88	5.3588	109.4988	3.21696	1.48052	5.04	5.1	3.55	2	1	1	2	2	0	2	2	30	3	157.8	91.2	36.62527	119.2	0.895
102793	2.06	4	1.24	5.33748	89.7372	2.39616	1.004063	4.14	5.1	9.12	2	1	1	2	2	0	2	2	30	3	155.1	105.7	43.93912	127.3	0.988
98119	1.93	4	0.88	5.4104	136.0632	2.75046	1.33882	4.37	5.3	7.79	2	1	1	2	2	0	2	2	30	3	168	91.6	32.45465	117.6	1.02
102847	0.15	4	1.09	5.11	45.78	2.224	0.655	3.62	5.1	6.51	2	1	1	2	2	0	1	2	30	3	155.6	62.7	25.89693	87.4	1.03
101228	0.8	3	0.78	5.637533	111.4008	2.23942	1.498	4.14	5.4	3.54	2	1	1	2	2	1	2	2	30	3	159.4	65.6	25.81827	86	1.04
93883	2.578292	4	1.73	5	42.66	1.552	0.44	3.49	5.6	2.77	2	3	1	2	2	0	1	2	30	4	164.7	114.5	42.21029	130.2	1.07
99353	2.49	4	1.24	5.33	228.3	2.845	0.61	4.37	5.5	15.57	2	3	1	2	2	0	1	2	30	4	168.5	138.9	48.9218	146	1.23
99986	0.81	1	1.94	5.2328	55.6092	2.0607	0.69542	3.85	5.2	8.58	2	1	2	2	2	0	2	2	31	1	162.3	101.9	38.68451	112.5	0.384
98661	2.43	2	1.47	5.16	18.3	1.164	0.598	2.92	4.9	0.27	2	3	1	2	2	0	2	2	31	4	168.5	44.6	15.70851	63.2	0.422
98985	5	5	1.71	5.357031	40.70048	2.666568	0.56653	4.71	5.3	0.27	2	3	1	2	2	0	2	2	31	6	165.1	51.3	18.82016	72.3	0.424
102358	2.79	4	1.22	5.3482	70.1036	1.99068	0.8574	3.93	5.4	0.3	2	3	1	2	2	0	1	2	31	6	159.6	57.1	22.41663	74.1	0.49
102948	5	4	1.97	5.11	32.52	3.181	0.587	5.4	5.2	0.55	2	3	1	2	2	0	2	2	31	3	162	52.9	20.15699	69.7	0.496

101370	5	4	2.33	5	21.9	2.508	0.711	5.17	4.9	0.96	2	3	1	2	2	0	1	2	31	2	165	59.9	22.00184	75.4	0.61
97034	1.44	4	1.47	5.22	27.6	3.233	0.632	4.99	5.2	0.36	2	2	1	2	2	0	2	2	31	3	162	57.1	21.75735	80.6	0.62
96312	2.08	4	1.16	5.88	61.08	3.776	1.14	5.46	4.8	0.74	2	3	1	2	2	0	1	2	31	3	154.9	66.4	27.67357	91	0.628
100546	3.41925	5	2.07	5.443	41.8377	3.424895	0.83984	6.05	5.2	0.36	2	3	1	2	2	0	2	2	31	3	182.4	85.5	25.69901	97	0.635
94245	2.88	5	1.89	5.1515	39.09738	1.811756	0.470282	3.72	4.8	0.74	2	3	1	2	2	0	2	2	31	3	164.6	72.1	26.61188	86	0.64
100644	0.13	4	1.06	5.5	125.76	2.017	1.411	3.72	5.3	0.7	2	1	1	2	2	0	1	2	31	3	161.2	62.7	24.1289	86	0.687
95342	0.41	5	1.78	5.27	35.4	1.448	0.485	3.47	5.4	2.96	2	3	1	2	2	0	2	2	31	3	163.7	57.9	21.60634	75	0.69
96275	5	5	1.55	5.094	39.67743	2.880462	0.921016	4.94	4.2	0.11	2	3	1	2	2	0	1	2	31	3	163.1	70.4	26.46456	85.2	0.692
101681	0.93	4	1.37	5.38	61.8	3.026	0.621	4.68	5.4	0.3	2	1	1	2	2	0	2	2	31	4	149.5	67.6	30.24575	86.2	0.695
102212	1.22	4	0.96	5.4544	95.26757	3.17776	1.4302	5.02	5.4	0.67	2	1	1	2	2	0	2	2	31	3	159	62.4	24.68257	90.2	0.703
97349	2.79	5	1.06	5.88	72.42	2.819	1.366	4.5	5.7	4.15	2	3	1	1	2	0	2	2	31	6	155.7	71.5	29.49367	96	0.81
96368	5	4	1.22	5.27	55.68	1.215	2.281	3.47	5.4	1.08	2	1	1	2	2	0	2	2	31	7	157.9	103.9	41.67267	124.4	0.821
98343	1.18	4	1.29	6.61	45.18	1.94	0.463	3.44	6.6	2.74	2	3	1	2	2	0	2	2	31	4	151.8	52.6	22.82665	81.6	0.834
102160	3.529785	5	1.86	5.190754	37.18242	2.378872	0.656478	4.32	5.4	5.04	2	3	1	2	2	0	2	2	31	6	156	51.7	21.24425	73.4	0.89
94436	0.82	2	1.22	6	113.64	2.974	0.835	4.58	5.4	1.45	2	1	1	2	2	0	2	2	31	4	174.9	94.5	30.89244	111.3	0.89
97771	3.4	5	1.45	5.72954	124.6992	3.059041	1.23152	5.07	5.6	18.63	2	3	1	2	2	0	1	2	31	3	175.6	131.7	42.71071	142.5	0.911
98323	1.6	5	1.4	5.5038	68.1824	1.855264	0.653408	3.08	5.1	3.96	2	3	2	2	2	0	1	2	31	3	176	61.6	19.88636	77.4	0.937
98280	4.93	5	0.91	6.61	90.54	2.379	2.958	4.63	6.8	5.6	2	2	1	2	2	0	2	2	31	6	161.4	71.3	27.37048	93.9	0.99
96111	1.9108	4	1.37	5.406099	79.18008	2.980801	0.989812	4.81	5.5	3.31	2	1	1	2	2	0	2	2	31	4	150.9	82	36.01102	95.4	1.01
95089	1.43	2	0.93	7.736890	236.8116	2.813706	1.669495	4.73	6.6	4.32	2	3	1	1	1	0	2	2	31	4	173	98.2	32.81099	114	1.02
95883	4.56	5	1.06	6.11	245.34	2.767	2.032	4.76	5.4	13.08	2	3	1	2	2	0	2	2	31	1	167.6	131.1	46.67181	143.6	1.05
95179	0.91	4	1.71	5.185795	69.0156	3.46262	0.976185	5.64	5.2	10.04	2	3	1	2	2	0	1	2	31	3	161.6	122.7	46.98528	126.1	1.31
94481	1.7	2	1.22	6.012652	153.1584	2.953534	1.36524	4.91	6	10.36	2	3	1	2	2	0	2	2	31	7	162	86.9	33.11233	111	1.43
94281	0.28	3	0.83	5.8432	158.245	2.8203	1.55108	4.45	5.6	6.06	2	1	1	2	2	0	2	2	31	7	171.1	115.7	39.52149	123.6	1.6
95896	2.11	5	1.78	5.068	59.86941	1.866596	0.527736	3.54	4.8	0.53	2	3	2	2	2	0	2	2	32	4	177.7	66.6	21.09111	71.7	0.404
97624	5	5	1.71	5.44	33.12	4.603	0.644	6.59	5.1	0.75	2	2	1	2	2	0	2	2	32	6	146.8	43.6	20.23179	68.4	0.454
93776	1.18	3	2.3	5.5	42.54	2.638	0.282	5.07	5.5	1.2	2	3	1	2	2	0	2	2	32	4	157.8	55.4	22.24825	75.6	0.521
93988	0.65	3	1.66	4.88	12.84	1.603	0.587	3.52	5.1	0.76	2	1	1	2	2	1	1	2	32	3	163.6	49	18.30752	72.5	0.54
100242	2.1646	2	2.66	5.3088	63.3	3.607755	1.121693	6.1	5.4	2.25	2	3	1	2	2	0	2	2	32	4	155.7	55.6	22.93494	79	0.54
101765	4.98	2	1.29	5.9252	88.322	2.400477	0.97607	4.01	5.7	1.18	2	3	1	2	2	0	2	2	32	4	150.6	70.2	30.95189	93.7	0.544
96297	5	5	1.27	4.72	31.98	2.069	1.106	3.85	5	7.15	2	3	1	2	2	0	2	2	32	3	166.8	74.7	26.84902	97.5	0.546
94982	1.82	4	0.96	5.4058	75.27864	2.161003	1.0206	3.96	5.4	0.27	2	1	1	2	2	0	1	2	32	4	168.3	58.9	20.79443	71.2	0.57
97948	2.59	5	1.6	5.2068	49.80816	2.455007	0.63924	4.32	5.4	1.12	2	3	1	2	2	0	2	2	32	3	172.9	55.3	18.49844	74.9	0.59
102035	0.33	5	1.24	5.72	31.38	3.75	0.971	5.43	5	0.57	2	2	1	2	2	0	2	2	32	3	170	77	26.6436	94.7	0.61
96838	3.2	5	1.86	6.05	83.04	3.698	0.813	5.92	5.5	0.77	2	3	1	2	2	0	2	2	32	6	163	74.7	28.11547	93.8	0.612
100077	0.49	2	1.58	5.4698	83.6748	3.236235	1.17642	5.35	5.4	13.28	2	1	1	2	2	0	2	2	32	1	159.8	82.2	32.1898	101.1	0.619
99672	3.66	5	1.37	5.88	66.96	2.302	0.858	4.06	5.1	0.63	2	3	2	2	2	0	2	2	32	6	157.6	59.5	23.95546	77.5	0.625
99481	1.01	3	1.19	5.485883	63.297	1.69282	0.746487	3.23	5	0.58	2	1	2	2	2	0	1	2	32	3	155	64.4	26.80541	76.4	0.63
94382	1	4	2.04	4.96125	41.0316	2.934653	0.89767	5.17	4.8	1.22	2	3	1	2	2	0	2	2	32	3	165.1	51.7	18.9669	70.5	0.645
100190	2.73	4	0.91	5.77	53.16	1.81	2.687	3.96	5.1	1.23	2	1	1	2	2	0	1	2	32	2	168.7	98	34.43467	112.7	0.678
96510	0.85	4	1.37	5.565177	70.9896	3.771306	1.37218	5.82	5.4	1.56	2	2	1	2	2	0	1	2	32	3	160	74.6	29.14063	85.7	0.71
94848	2.65	4	1.06	5.3224	76.8444	2.126808	0.740786	3.52	5	4.26	2	3	2	2	2	0	1	2	32	2	160	74.2	28.98438	93.2	0.72
100196	1.48	3	2.2	5.27	53.82	3.129	0.587	5.59	5.4	0.79	2	3	1	2	2	0	2	2	32	4	170.2	66.5	22.95633	80.6	0.728
102099	1.91	3	1.42	12.5	58.5	1.965	1.095	3.88	5.5	1.23	2	3	1	2	1	0	2	2	32	3	163.2	128.9	48.39635	121.6	0.77
98437	0.6	4	1.53	5.72	37.32	2.25	0.734	4.11	5	2.35	2	2	2	2	2	0	2	2	32	4	164.3	67.9	25.15328	87.8	0.79
97322	2.28	4	1.27	6.051511	129.6158	2.42208	0.91839	4.19	5.9	5.09	2	3	1	2	2	0	1	2	32	4	174.7	110.6	36.23843	118.4	0.812
95726	0.99	5	1.55	5.27	53.46	2.845	0.44	4.6	5.6	1.7	2	1	1	2	2	0	2	2	32	4	167.5	68.6	24.45088	89.5	0.813
96721	5	3	0.83	8.16	165.72	2.689	2.529	4.68	6.6	5.78	2	3	1	1	1	0	2	2	32	1	167	144.6	51.8484	136.6	0.82
97234	0.28	4	0.93	5.38	101.34	3.801	2.066	5.69	5.3	3.2	2	1	1	2	2	0	1	2	32	7	161.1	90.8	34.98604	110.9	0.864
94819	1.14	2	1.97	5.05	24.66	2.121	0.587	4.34	5.1	6.64	2	1	1	2	2	0	2	2	32	1	153	56.1	23.96514	84.3	0.869
96257	1.42	2	1.09	7.11	160.98	4.784	1.716	6.65	5.9	3.76	2	3	1	1	1	0	2	2	32	1	155.1	68.4	28.43364	94.6	0.88
96939	0.71	3	0.98	5.27	30.24	3.155	0.485	4.37	5.5	5.55	2	1	1	2	2	0	2	2	32	3	158	75.9	30.40378	92.1	0.89
96011	0.61	3	1.66	5.238728	51.1314	3.103333	0.96775	5.3	4.8	0.85	2	3	1	2	2	0	2	2	32	1	162.6	64	24.20688	90.6	0.95
97423	0.87	3	1.06	5.803266	131.2596	3.065495	1.39442	4.86	5.7	6.68	2	1	1	2	2	0	2	2	32	2	157.9	97.9	39.26616	110.1	0.99
100434	1.82	4	1.45	5.351781	70.2424	2.092518	0.688256	3.67	5.3	16.75	2	3	1	2	2	0	2	2	32	1	152.5	104.5	44.93416	122.2	1.01
98512	0.06	4	1.58	14	102.78	2.25	1.129	4.34	8.4	32.67	2	1	1	2	1	0	1	2	32	4	161.1	108.3	41.72894	128.8	1.03
98799	5	5	1.06	6.2342	175.2147	2.28366	1.15784	3.41	5.4	44.63	2	1	1	2	2	0	1	2	32	3	170.2	125.9	43.46169	116.5	1.05
97648	3.43	5	1.24	5.38	61.62	2.664	0.813	4.27	5.6	0.71	2	3	1	2	2	0	2	2	32	3	164.8	126.3	46.50385	136.6	1.23
96901	0.85	2	1.55	4.83	14.58	2.56	0.508	4.34	5.1	0.3	2	1	1	2	2	0	2	2	33	1	156.5	64.1	26.17154	82.7	0.43
94388	0.8	4	1.97	5.38	41.46	1.629	0.384	3.78	5.2	0.46	2	1	1	2	2	0	1	2	33	4	165.4	90.6	33.11747	91.7	0.465

97795	2.701604	4	1.84	5.0481	39.12634	2.87564	0.59609	4.99	5.1	0.31	2	3	1	2	2	0	2	2	33	3	161.4	62.1	23.83881	95.9	0.473
97042	5	5	1.89	5.05	14.22	1.474	0.7	3.67	5.1	0.62	2	3	1	2	2	0	2	2	33	3	170.2	65.5	22.61113	81.8	0.52
99918	2.39	5	1.42	5.211110	51.027	2.8069	0.74508	4.55	5.3	1.42	2	3	1	2	2	0	2	2	33	6	160.4	67.2	26.11924	82	0.6
95423	1.22	5	1.29	4.83	93.66	2.741	0.463	4.24	5.7	0.72	2	1	1	2	2	0	2	2	33	6	165.5	72.1	26.32324	87.6	0.624
94368	5	4	1.06	9.8866	127.4748	2.16762	1.58646	2.66	8.2	1.67	2	3	1	1	1	0	2	2	33	3	160.7	65.9	25.51841	83.7	0.64
95115	1.63	1	1.63	5.3688	41.13693	2.362588	0.58458	4.11	5.2	0.7	2	1	1	2	2	0	2	2	33	4	157.1	51.5	20.86675	76.5	0.642
97364	1.02	2	1.16	5.579213	71.1444	2.131391	0.95334	3.75	5.5	0.39	2	1	1	2	2	0	1	2	33	6	164.3	58.1	21.52291	78.7	0.663
102364	5	5	1.55	5.138	37.04673	2.954749	0.951893	5.04	5	0.58	2	3	1	2	2	0	2	2	33	3	170.6	85.3	29.30832	107	0.674
102112	2.36	4	1.53	5.16	29.1	2.405	0.79	4.29	5.2	0.23	2	2	1	2	2	0	2	2	33	4	161.5	42.3	16.21793	64.5	0.68
102044	2.06	5	2.07	5.26133	48.9918	3.188425	0.768963	5.74	5.4	0.38	2	3	1	2	2	0	2	2	33	4	153.5	62.4	26.48304	78.9	0.72
97485	5	5	1.81	5.33	34.68	2.741	0.508	4.78	5.1	0.55	2	3	1	2	2	0	2	2	33	3	168.2	66.5	23.50551	86.6	0.731
101138	5	5	1.34	5.66	126.36	4.138	0.847	5.87	4.8	2.23	2	3	2	2	2	0	2	2	33	3	171.4	118	40.16617	121.4	0.74
100701	5	5	1.42	5.3986	99.0768	3.21022	1.161273	5.2	5.4	4.98	2	3	1	2	2	0	2	2	33	4	163.4	116.5	43.63368	117.4	0.744
94686	3.48	3	1.37	6.16	92.4	3.776	1.502	5.84	5	3.58	2	3	2	2	2	0	1	2	33	3	172.5	76.2	25.60807	97.7	0.746
96725	1.076855	1	1.01	5.27	77.22	2.379	1.118	3.9	5.6	1.1	2	1	2	2	2	0	2	2	33	2	156.4	79.3	32.41901	102.8	0.76
101252	0.82	4	1.34	4.55	51.18	2.405	0.858	4.14	5.3	1.19	2	1	1	2	2	0	2	2	33	7	157	98.5	39.96105	120.8	0.76
99983	0.75	4	1.4	5.44	69.9	4.008	0.666	5.72	5.4	1.99	2	3	1	2	2	1	1	2	33	4	171.6	150.6	51.1435	152	0.77
100072	1.89	2	1.6	6.4976	119.8987	2.41176	1.01428	4.24	6.8	0.6	2	1	1	2	2	1	2	2	33	1	155.2	49.8	20.67502	78.4	0.78
95128	1.696919	4	1.11	5.44	58.68	3.672	0.677	5.09	5.5	15.95	2	1	1	2	2	0	1	2	33	4	151.1	77.4	33.90096	92.3	0.84
96269	0.49	1	1.22	5.61	181.44	3.026	1.197	4.78	5.7	3.52	2	1	1	2	2	0	2	2	33	1	148.9	63.8	28.77606	96.5	0.876
96519	1.18	3	1.24	4.94	32.94	2.043	0.44	3.49	5.1	2.68	2	1	1	2	2	0	1	2	33	4	154	53.6	22.60078	78.2	0.878
101716	1.47	4	0.93	5.5	177.9	3.517	1.321	5.04	5.5	4.57	2	1	1	2	2	0	1	2	33	1	155.4	90.5	37.47542	111.4	0.89
94576	0.58	3	1.29	5.27	114.72	1.94	0.587	3.49	5.3	13.4	2	1	2	2	2	1	2	2	33	1	159	93.8	37.10296	115.6	0.89
96417	4.78	5	1.11	5.27	69.78	2.302	0.553	3.67	5	1.52	2	3	2	2	2	0	2	2	33	6	156.1	54.2	22.24301	82.1	0.93
95442	3.96	5	1.22	5.4382	67.5696	2.706597	0.73436	4.4	5.1	0.93	2	3	1	2	2	0	2	2	33	3	173.8	76.2	25.22641	91.5	1.02
96681	2.2	3	1.29	5.94	143.34	3.284	1.389	5.22	5.3	10.76	2	1	1	2	2	0	2	2	33	7	167.7	91.5	32.53531	106.2	1.05
101125	0.04	3	0.98	5.4472	91.8096	3.77517	2.04932	5.82	5.7	1.25	2	1	2	2	2	0	2	2	33	3	155.7	60.3	24.87368	93.1	1.06
94860	1.26	4	1.55	5.1518	68.5242	2.69281	0.88942	4.63	5	4.48	2	3	2	2	2	0	1	2	33	4	168.7	106	37.24566	123.2	1.07
101139	5	4	1.09	5.4436	86.3832	2.755256	1.1854	4.58	5.1	2.76	2	1	1	2	2	0	1	2	33	3	163.6	70.2	26.22832	99.3	1.11
100144	0.57	4	1.09	5.33	86.82	1.836	1.479	3.59	5.4	1.02	2	1	1	2	2	1	1	2	33	2	156.9	78.7	31.96898	89	1.15
100280	5	3	1.24	5.44	100.5	2.612	1.366	4.47	5.6	5.11	2	1	1	2	2	0	2	2	33	3	161.2	101.3	38.98337	101.2	1.25
101281	0.41	2	1.71	5.61	40.38	3.646	0.632	5.64	5.2	0.45	2	1	1	2	2	2	2	2	34	1	150.3	59	26.11765	86	0.45
102344	2.15	4	1.42	5.17285	47.2764	2.336623	0.61526	4.16	5.2	0.11	2	1	1	2	2	0	2	2	34	6	150.6	42.3	18.6505	67.1	0.469
94044	5	2	1.6	5.61	21.12	1.603	0.271	3.34	5.1	0.19	2	3	1	2	2	0	2	2	34	2	157.5	54	21.76871	73.3	0.496
97463	5	5	1.6	5.38	57.18	2.095	0.621	3.98	5.2	0.31	2	3	1	2	2	0	2	2	34	6	161.5	45.7	17.52149	66.3	0.53
102454	2.52	3	1.42	5.83	49.5	1.784	0.576	3.47	5.6	0.84	2	1	1	2	2	1	2	2	34	1	167.4	65.9	23.5166	83.9	0.55
97946	2.59	4	2.3	5.0798	40.3716	3.021185	0.75828	5.61	5.1	0.44	2	3	1	2	2	0	2	2	34	3	163	82.7	31.1265	100.7	0.56
96737	2.39	5	1.86	5.2472	53.6562	3.33076	0.72291	5.64	5.3	0.97	2	3	1	2	2	0	2	2	34	6	160.3	52.6	20.47004	75.9	0.58
101493	3.06055	2	1.34	6.38	62.58	2.276	0.892	4.03	5.8	1.69	2	1	1	2	2	0	2	2	34	1	157.3	66.8	26.99722	94.7	0.58
96322	1.02	1	1.4	5.05	20.88	2.56	0.903	4.37	4.7	0.87	2	1	1	2	2	0	1	2	34	6	147.5	52.2	23.99311	81	0.602
100500	2.732976	4	1.58	5.38	38.76	3.646	0.813	5.59	5	1.75	2	3	1	2	2	0	2	2	34	4	168	86.8	30.75397	108.5	0.636
95568	2.721633	5	1.42	5.11	43.38	1.241	0.689	2.97	5.2	0.44	2	3	1	2	2	0	2	2	34	6	166	45.4	16.47554	67.5	0.65
93789	5	5	2.43	4.94	38.82	2.146	0.531	4.81	4.9	0.79	2	3	1	2	2	0	2	2	34	3	173.4	71	23.61349	79.9	0.67
99404	2.402733	3	1.99	5.2825	54.46696	2.867355	0.720461	5.04	5	2.21	2	3	1	2	2	0	2	2	34	2	168.3	70.9	25.03099	89.4	0.693
101256	1.18	4	1.14	6.72	208.14	2.069	1.468	3.88	6.6	10.42	2	3	1	1	1	0	1	2	34	4	161.9	138.6	52.87733	141.1	0.699
102532	1.73	4	1.63	5.148141	57.78925	2.53162	0.56119	4.37	5.3	1.61	2	3	1	2	2	0	1	2	34	4	162.7	84.5	31.92138	96.2	0.709
94531	1.52	4	1.22	6.05	126.36	3.336	0.745	4.89	5.7	1.57	2	1	1	2	2	0	2	2	34	1	157.6	73.6	29.6323	101.7	0.73
99182	1.81478	1	1.22	5.387966	59.6415	2.903741	1.0394	4.6	5.3	1.36	2	1	1	2	2	0	2	2	34	2	167.7	79.7	28.3395	99	0.743
97048	0.62	4	1.4	5.44	66.78	3.595	0.485	5.22	5.2	4.83	2	2	1	2	2	0	2	2	34	4	159	85.1	33.66164	96.6	0.758
101378	5	5	1.32	5.675	74.79648	3.799649	1.30696	5.84	5.4	0.9	2	3	2	2	2	0	2	2	34	6	160.1	64.8	25.28089	82.1	0.77
96084	1.65	3	1.14	6.200911	152.1276	2.03098	1.30886	3.57	6.3	7.01	2	3	1	2	2	0	1	2	34	4	170.2	158.2	54.61191	152.7	0.78
97451	3.720437	5	1.4	5.489413	63.6002	2.862926	0.793133	4.63	5.5	1.7	2	3	1	2	2	0	2	2	34	2	161.2	73.9	28.439	91.6	0.79
101517	2.59	5	1.71	5.234413	50.84796	2.71042	0.63586	4.58	5.5	0.75	2	3	1	2	2	0	2	2	34	2	162.3	69.1	26.23258	83.3	0.805
94406	2.033	4	1.99	5.7185	101.6236	3.35564	1.19946	5.51	5.6	4.68	2	3	1	2	2	0	2	2	34	4	165.7	121.2	44.14257	129.6	0.819
95474	0.62	4	1.29	5.72	46.02	2.819	0.565	4.37	5.7	3.5	2	1	1	2	2	0	2	2	34	4	159.7	103.2	40.4641	110.4	0.823
100707	3.56	4	1.22	5.523177	74.23608	3.917393	1.74803	6.28	4.9	1.29	2	3	2	2	2	0	2	2	34	3	168.8	71.6	25.12859	89.5	0.86
95807	5	5	1.78	5.5718	53.11068	2.9983	0.834067	5.02	5.6	3.19	2	3	1	2	2	0	2	2	34	3	161.4	118.4	45.45113	131.6	0.922
94508	4.86	5	1.55	5.621066	58.6844	2.86436	0.75724	4.81	5.6	2.44	2	3	1	2	2	0	2	2	34	4	151.7	70.8	30.76537	91.3	0.971
96074	4.78	5	1.42	6.11	95.16	2.483	0.542	4.16	4.9	6.51	2	3	2	2	2	0	2	2	34	3	171.4	111.9	38.08978	120.7	0.98
96196	0.89	1	1.14	5.5	80.82	2.793	1.106	4.45	5.1	9.59	2	1	1	2	2	0	2	2	34	3	163.2	157.4	59.09686	154.9	0.99

101070	2.63	4	1.24	5.05	67.68	2.25	0.644	3.78	5.4	12.19	2	3	1	2	2	0	2	2	34	1	166.4	89	32.14277	101.4	1.02
98303	0.2	4	1.32	4.94	81.96	2.198	0.485	3.75	5.4	7.54	2	3	1	2	2	0	1	2	34	4	155.8	68.9	28.3847	92.4	1.03
93952	1.1	4	1.06	6.49	164.52	2.327	1.863	4.24	6	8.48	2	1	1	2	2	0	1	2	34	3	161.7	98.8	37.78652	117.2	1.04
98056	1.82	3	0.91	5.05	54.12	3.957	1.976	5.77	5.8	6.26	2	1	1	2	2	0	2	2	34	3	164.6	91.9	33.92	109.8	1.05
95867	1.27	4	0.65	6.33	150.3	2.974	1.389	4.27	5.7	5.75	2	3	1	2	2	0	1	2	34	3	161.4	126.1	48.40698	124	1.12
98028	0.83	4	0.62	5.85225	125.9628	3.512945	2.085	5.51	5.1	4.2	2	1	1	2	2	1	1	2	34	3	155.7	80.1	33.04116	100.3	1.13
97627	3.47	4	0.91	5.87352	154.8176	3.80094	1.89756	5.92	5.8	12.34	2	3	1	2	2	0	1	2	34	3	175.9	161.4	52.16412	153.3	1.19
99279	1.91	2	1.19	5.77	79.68	2.353	1.818	4.37	5.8	15.54	2	1	1	2	2	0	2	2	34	1	150.7	76	33.46471	100	1.22
98035	1.79	3	1.4	5.27	49.5	1.836	1.084	3.72	5	2.01	2	3	2	2	2	0	1	2	35	1	163.6	85.2	31.83266	99.4	0.385
101957	4.09	3	2.15	5.33	21.24	2.043	0.384	4.37	5.2	0.56	2	3	1	2	2	0	1	2	35	6	149.2	58.1	26.09988	76	0.45
93929	5	5	2.4	4.61	27.42	4.965	1.919	8.25	5.2	5.8	2	3	1	2	2	0	2	2	35	3	171.5	79.3	26.96156	102.6	0.51
100673	2.31	2	1.94	5.2756	42.7796	2.278735	0.545342	4.32	5.2	1.06	2	3	1	2	2	0	1	2	35	4	150.5	47.9	21.14767	69.6	0.541
94687	0.58	5	1.76	5.139093	30.8328	2.144707	0.5611	4.19	5.4	0.37	2	3	1	2	2	0	2	2	35	6	158	55.3	22.1519	85.1	0.57
93837	1.03	3	1.06	5.5	32.76	2.689	0.565	4.01	5.2	4	2	1	1	2	2	0	2	2	35	3	166.8	108.7	39.06946	117.4	0.614
98718	1.82	3	1.47	5.213081	57.2974	2.61234	0.819348	4.4	5.3	4.14	2	1	1	2	2	0	2	2	35	2	152.9	66	28.23118	81.5	0.63
95221	0.82	4	2.02	4.9894	39.7903	2.383419	0.640126	4.68	5.1	1.02	2	3	1	2	2	0	1	2	35	7	156.8	59.5	24.20053	82.9	0.67
102064	1.44	4	1.5	5.22	58.2	3	1.253	5.07	5.3	0.62	2	1	1	2	2	0	2	2	35	1	156.3	65.8	26.93444	92.3	0.709
96163	4.43	2	1.16	6.058011	113.7924	2.790292	1.3559	4.63	5.7	4.08	2	3	1	2	2	0	2	2	35	6	145.6	70.4	33.20855	95	0.73
99714	1.18	4	1.14	5.4786	62.418	3.589457	1.4176	5.51	5.3	1.12	2	2	1	2	2	0	1	2	35	3	157.8	92.7	37.22766	122.5	0.791
99442	1.22	4	0.83	5.44	41.04	2.043	0.835	3.26	5.3	1.28	2	3	1	2	2	0	1	2	35	3	159.1	60.9	24.05896	76.2	0.8
97093	3.03	4	1.63	5.55	57.12	2.87	0.598	4.78	5.7	5.68	2	3	1	2	2	0	2	2	35	4	174.8	111	36.32789	106.8	0.909
100313	1.21	4	0.98	5.4094	76.24283	2.939866	1.11206	4.55	5.2	1.55	2	1	1	2	2	0	2	2	35	3	169.5	57.4	19.97894	81.8	0.943
99322	3.73	5	1.24	5.61	60.18	3.957	1.727	6	5.5	0.62	2	3	1	2	2	0	2	2	35	4	155.1	74.9	31.13567	98.6	0.966
98102	3.86	5	1.19	5.61	80.46	2.87	0.847	4.45	5.6	7.45	2	3	1	2	2	0	2	2	35	6	157.3	72	29.0988	97.4	0.988
102821	2.22	4	1.63	5.21465	60.498	3.3245	0.89342	5.56	5.3	1.08	2	3	1	2	2	0	2	2	35	4	178	80	25.24934	86	1.04
101191	2.41	3	1.4	5.16	33.54	3.31	1.614	5.46	5.3	1.62	2	3	1	2	2	0	1	2	35	3	158.5	76.9	30.61032	105	1.05
96907	3.92	4	1.63	5.27	73.14	2.664	1.005	4.76	5	14.18	2	3	2	2	2	0	1	2	35	3	171.6	105.6	35.86157	117	1.05
100025	1.11	5	0.85	7.72	583.38	2.586	0.96	3.88	5.7	13.25	2	1	1	2	1	0	1	2	35	3	167.4	148.9	53.13538	144.6	1.06
102074	0.28	4	2.09	4.44	48.96	2.095	0.497	4.42	5.5	2.29	2	3	1	2	2	0	1	2	35	4	160.3	86.7	33.74054	107.1	1.08
101551	1.58	4	1.6	5.281528	59.5882	3.095	1.012531	5.2	5.1	8.06	2	1	1	2	2	0	2	2	35	3	155.5	81.8	33.82926	109	1.09
97458	2.64	5	1.68	5.382548	47.82478	2.622615	0.663556	4.55	5.3	3.54	2	3	1	2	2	0	2	2	35	3	171.1	101.7	34.73929	119.1	1.11
94857	1.57	4	1.19	6.16	108.84	4.37	2.281	6.59	5.1	4.18	2	1	1	2	2	1	1	2	35	2	171.7	138.1	46.8439	133.5	1.13
97235	0.02	3	1.16	5.5794	122.2704	3.073379	1.61214	5.07	5.7	6.12	2	1	1	2	2	0	2	2	35	3	168.1	83	29.37262	103.5	1.15
96433	2.44	4	1.06	5.5	50.52	3.336	1.682	5.17	5.3	3.66	2	1	1	2	2	0	2	2	35	3	160.7	89.9	34.81192	119.3	1.19
96123	4.34	5	0.91	5.9206	197.9475	2.22274	1.2236	3.65	5.5	11.24	2	3	1	2	2	0	1	2	35	1	155.1	86.6	35.99932	105	1.33
99920	0.33	4	1.84	4.61	39.36	3.801	1.558	6.36	4.7	2.33	2	3	1	2	2	0	2	2	36	3	172.7	84.6	28.36519	102.9	0.377
95591	4.62	5	2.17	5.55	32.94	1.474	0.485	3.88	5.1	0.39	2	3	1	2	2	0	2	2	36	6	150.3	47.5	21.02692	73.9	0.45
95415	5	5	1.6	5.330921	47.41488	2.658959	0.557586	4.58	5.2	0.56	2	3	1	2	2	0	2	2	36	3	153.9	53.1	22.41905	74.1	0.49
98545	2.67	3	1.73	5.05	36.6	2.095	0.644	4.11	5.1	0.53	2	3	1	2	2	0	2	2	36	2	156.4	59.5	24.32447	85.5	0.58
100623	5	5	1.42	5.16	21.84	2.327	1.558	4.47	4.9	3.63	2	3	1	2	2	0	1	2	36	3	171.3	71.4	24.33232	87.3	0.58
101659	5	4	1.97	5.88	21.36	2.405	0.327	4.53	5.4	2.42	2	2	1	2	2	0	2	2	36	2	166	58.6	21.26579	75.9	0.6
97025	2.5688	1	1.66	10.5	43.5	2.689	1.163	4.89	8.4	5.94	2	3	1	2	1	0	2	2	36	1	161.6	67.5	25.84765	90.5	0.67
97741	1.91	4	1.63	5.83	36.06	2.56	0.316	4.34	5.3	0.6	2	3	1	2	2	0	2	2	36	3	160.1	58.5	22.82302	78.7	0.674
100241	5	5	1.73	5.122466	53.202	2.477168	0.56068	4.47	5.2	0.87	2	3	1	2	2	0	2	2	36	3	161.4	55.5	21.30522	75.3	0.69
99378	5	5	1.22	5.4978	73.70208	2.674110	0.87792	4.32	5.1	0.98	2	3	1	2	2	0	2	2	36	1	158.2	70.5	28.16931	93.6	0.697
96014	5	5	1.78	5.2498	61.10091	2.306712	0.59925	4.24	5	0.74	2	3	2	2	2	0	2	2	36	6	162	46.9	17.87075	74.5	0.7
96711	0.68	4	1.32	5.266466	71.5956	1.873253	0.62616	3.52	5.4	0.83	2	1	2	2	2	1	1	2	36	1	160.7	66.4	25.71203	88.8	0.7
94532	0.8	4	1.11	5.29748	71.5908	3.34582	1.1839	5.22	5	1.65	2	1	1	2	2	0	2	2	36	1	155.1	72.6	30.17957	99.7	0.72
102223	5	5	1.71	5.77	30.18	2.327	1.344	4.65	5.2	13.9	2	3	1	2	2	0	1	2	36	3	158	57.7	23.11328	82.5	0.725
99891	5	5	1.55	5.495723	67.4428	2.972513	0.830300	5.07	5.4	1.81	2	3	1	2	2	0	2	2	36	3	161.6	53.7	20.56324	77.7	0.746
99857	3.32	3	0.98	11.5	155.76	5.508	3.252	7.99	7.9	8.69	2	2	1	2	2	0	2	2	36	1	153.3	86.6	36.84967	105.5	0.761
100204	1.02	1	0.93	4.66	23.4	3.103	1.614	4.78	5.2	3.47	2	1	1	2	2	0	2	2	36	2	173.6	87.2	28.93457	85.8	0.8
102278	3.04	4	1.2604	11.2	146.8692	2.815806	1.56336	4.6216	7.9	6.29	2	3	1	1	1	0	1	2	36	1	168.7	111.3	39.10795	132.2	0.8
101871	0.28	2	1.19	5.620109	79.3872	2.971504	1.0626	4.73	5.4	1.96	2	1	1	2	2	0	2	2	36	3	163.6	81.1	30.30081	95.7	0.82
98096	1.05355	2	1.29	5.484114	58.746	2.52962	0.8041	4.24	5.3	1.39	2	1	1	2	2	0	2	2	36	2	152.4	61.5	26.47922	85.6	0.85
102194	2.86	4	1.24	4.94	48.72	2.327	0.723	3.9	5.3	5.03	2	3	1	2	2	0	1	2	36	1	155.3	98.6	40.88218	119.8	0.85
98733	2.15	4	1.22	5.38	72.72	2.146	1.919	4.24	5.3	1.57	2	3	1	2	2	0	1	2	36	3	166.7	83.4	30.01199	97.1	0.859
98350	3.32	4	1.11	11.9	147.6	5.663	2.879	8.09	8.6	11.63	2	2	1	2	2	0	1	2	36	1	155	100.4	41.7898	122.4	0.884
102005	2.64	4	1.24	5.884145	101.909	3.652386	1.2954	5.56	5.7	5.11	2	3	1	2	2	0	1	2	36	3	168.9	127.6	44.72923	140.5	0.889
100271	5	4	1.19	5.684777	104.547	3.54198	1.52009	5.56	5.1	2.88	2	3	1	2	2	0	1	2	36	3	159.8	81.1	31.75904	109.8	0.89

96609	0.957366	3	1.01	5.77	74.22	2.121	0.621	3.41	5.8	2.4	2	1	1	2	2	0	1	2	36	4	160.8	65.1	25.17729	87	0.9
97035	0.73	4	1.14	5.4258	77.89493	2.63516	0.85958	4.29	5.2	1.63	2	1	1	2	2	0	2	2	36	4	164.1	82.7	30.7106	97.6	0.91
96551	1.42	4	1.11	6.0576	135.692	3.39806	1.45271	5.38	6.1	4.95	2	1	1	2	2	0	1	2	36	7	163	99.1	37.29911	115.3	0.918
101670	5	5	1.4	5.881511	119.4252	2.62587	1.055653	4.42	5.8	5.05	2	3	1	2	2	0	2	2	36	6	157.7	71.1	28.58948	96.2	0.97
95574	1.23	5	1.19	5.291	87.09288	2.64546	0.91691	4.34	5	0.85	2	1	1	2	2	0	2	2	36	3	176.5	97.2	31.2016	105.8	0.971
99864	1.51	4	1.06	6.33	105.42	2.017	0.621	3.36	5.4	3.42	2	3	2	2	2	0	1	2	36	3	168.3	124.3	43.88367	136	0.99
99536	1.24	4	1.29	5.451457	65.316	3.402867	1.00412	5.3	5.4	2.9	2	1	1	2	2	0	1	2	36	4	172.2	126.6	42.69406	129.3	1.06
97270	0.28	2	0.88	6.22	59.04	3.051	0.881	4.34	5.2	3.83	2	1	1	2	2	0	2	2	36	4	156.3	72.1	29.51327	96.6	1.1
94061	0.87	2	1.01	6.8324	151.2384	2.76688	1.55014	4.42	6	45.07	2	1	1	2	3	0	2	2	36	1	154.2	78.3	32.9301	104.9	1.12
96951	1.48	4	1.19	5.55	50.04	3.31	4.11	6.39	5.3	5.25	2	2	1	2	2	0	2	2	36	3	165.1	92	33.75155	116.1	1.14
101115	0.49	4	1.34	5.38	60.72	2.534	0.44	4.09	5.8	7.66	2	1	1	2	2	0	2	2	36	4	168.1	107.7	38.11363	107.2	1.14
100860	0.99	2	1.06	8.16	168.72	3.103	1.163	4.71	6.8	9.01	2	1	1	1	1	0	2	2	36	4	169.7	123.6	42.91951	127.1	1.16
95058	4.81	2	0.98	6.0784	172.2652	2.89556	1.62937	4.84	5.8	9.14	2	3	1	2	2	0	2	2	36	3	160.4	163.2	63.43244	166	1.76
100510	0	2	1.6	5.3546	119.508	2.63102	0.97154	4.47	4.2	3.84	2	1	2	2	2	0	2	2	37	4	149.7	73.2	32.66386	99.1	0.46
95094	0.48	1	1.11	5.787252	103.6193	3.013835	1.32472	4.86	6	1.07	2	1	1	2	2	0	2	2	37	2	143.3	58.4	28.4394	88.1	0.47
100934	5	5	1.71	4.39	61.5	2.457	0.531	4.4	4.8	4.29	2	3	1	2	2	0	2	2	37	3	163.8	105.6	39.35828	109	0.475
94478	0.91	4	2.2	5.27	40.5	1.603	0.339	3.96	5.6	0.6	2	1	1	2	2	0	1	2	37	4	163.6	60.6	22.64154	76.1	0.52
100386	1.99	4	1.73	7.16	505.98	3	1.231	5.3	5.3	5.64	2	3	1	2	2	0	2	2	37	1	159.1	100.2	39.5847	121.3	0.55
99985	2.99	5	1.66	5.5	29.34	1.862	0.418	3.7	5.1	0.86	2	3	1	2	2	0	1	2	37	3	165.5	67.5	24.64381	88.5	0.57
101002	4.81	5	1.71	5.1934	35.4285	1.907795	0.633829	3.78	5	0.49	2	3	1	2	2	0	2	2	37	2	162.3	49.8	18.90568	69.7	0.6
95444	0.88	2	1.24	5.3302	58.5936	2.107551	0.71568	3.7	5.3	0.31	2	1	1	2	2	0	2	2	37	3	154.1	59.4	25.01389	79.2	0.63
96428	5	5	1.55	5.524066	46.91568	3.035812	0.82797	4.89	5.5	0.65	2	3	1	2	2	0	2	2	37	7	162.4	70.1	26.57945	91.2	0.63
99231	5	3	1.42	5.16	74.88	2.25	1.716	4.45	4.7	2.75	2	1	1	2	2	0	1	2	37	3	160.4	76.5	29.73396	101.5	0.63
96474	2.38	5	1.4	5.3394	50.01034	2.972635	0.77202	4.84	5.5	0.46	2	3	1	2	2	0	2	2	37	6	156.1	52.3	21.46327	73.6	0.654
96671	5	5	1.84	5.376413	44.7186	2.745891	0.63562	4.73	5.5	1.8	2	3	1	2	2	0	2	2	37	6	162.8	65	24.52475	85.5	0.66
98023	5	5	1.78	5	139.92	2.586	1.31	4.97	5.4	0.76	2	3	1	2	2	0	2	2	37	4	153.7	84.9	35.9385	105.1	0.66
100838	0.49	4	1.14	5.5536	110.0484	3.87084	1.57872	5.97	5.7	3.62	2	1	1	2	2	0	1	2	37	3	162.5	128.8	48.77633	134	0.732
102346	0.09	2	0.75	16	252.48	3.016394	7.271	4.76	10.9	13.75	2	2	1	2	1	0	2	2	37	3	169.5	145.6	50.67829	144	0.76
99338	1.29	1	1.32	5.05	71.52	3.698	2.134	6	5.2	1.52	2	1	1	2	2	0	2	2	37	1	162.6	70	26.47628	90.2	0.77
94469	5	5	1.53	5.33	51.24	2.845	0.598	4.65	5.4	1.48	2	3	1	2	2	0	2	2	37	7	163.3	103.3	38.73718	95.6	0.77
97449	1.25	4	2.09	5.4842	62.745060	3.265695	0.764346	5.38	5.7	2.81	2	3	1	2	2	0	2	2	37	7	162.7	93	35.1324	115.8	0.81
99938	0	4	3.05	5.270577	69.6684	3.04832	0.876815	5.74	5	1.59	2	3	1	2	2	0	2	2	37	4	161.6	51.5	19.7208	74	0.83
102885	2.79	4	1.55	4.88	69.78	2.741	0.508	4.53	5.4	4	2	3	1	2	2	0	2	2	37	4	167.8	84.7	30.0815	96.9	0.83
98680	2.07	4	1.71	4.61	73.32	3.414	0.655	5.43	5.5	2.38	2	3	1	2	2	0	2	2	37	4	154.7	100.6	42.03561	124.6	0.876
98148	0.76	3	0.83	5.33	56.46	3.62	2.168	5.43	5.2	0.7	2	1	1	2	2	0	2	2	37	3	161.6	56.7	21.71203	82	0.898
99359	4.58	5	0.93	4.83	102.0348	3.129	1.423	4.71	5.1	5.833766	2	1	1	2	2	0	2	2	37	6	155.5	69.4	28.70111	102.4	0.95
100714	1.63	5	1.47	5.77	39.06	2.612	0.869	4.47	5.3	2.94	2	3	1	2	2	0	2	2	37	3	171	78	26.67487	95.5	0.952
97338	0.59	3	1.14	5.88	189.36	3.414	0.971	4.99	5.5	2.61	2	1	1	2	2	0	2	2	37	7	170.7	98.1	33.66681	115.2	1.03
99695	2.15	3	1.19	5.94	120.84	3.233	0.971	4.86	5.6	3.93	2	1	1	2	2	0	2	2	37	3	161.7	127.9	48.91595	118.9	1.06
100947	1.0168	1	0.91	5.84297	103.3104	4.004491	2.64288	6.44	5.4	4.31	2	2	1	2	2	0	1	2	37	3	163.2	99.1	37.20774	121.7	1.09
99024	5	5	1.47	5.22	16.38	2.922	0.35	4.55	5	0.31	2	2	1	2	2	0	2	2	38	6	166.7	64.6	23.2467	77	0.38
97456	0.62	5	2.02	5.44	24.66	1.396	0.113	3.47	5.4	0.43	2	3	1	2	2	0	2	2	38	4	169.7	76.3	26.49481	82.7	0.396
93829	5	5	1.6	5.365	73.53050	2.543437	0.71586	4.4	5.6	7.87	2	3	1	2	2	0	2	2	38	4	161.4	82.5	31.66991	96.8	0.49
98394	3.42	4	2.38	5.0617	37.87643	2.407005	0.55192	4.86	5.1	0.45	2	3	1	2	2	0	2	2	38	2	163.6	66.9	24.99537	86.4	0.54
99009	1.34	4	1.66	5.021764	39.3996	2.09722	0.73182	4.19	4.8	1.09	2	3	1	2	2	0	1	2	38	3	165.5	74.2	27.08993	89.9	0.55
94248	0.87	3	2.04	5.4416	102.3876	2.79448	1.15834	5.12	5.4	13.98	2	1	1	2	2	1	2	2	38	4	161.8	83.9	32.04829	95.5	0.569
102429	2.03	1	0.98	11.1578	211.038	3.883354	2.0228	5.92	9.3	11.2	2	3	1	1	1	0	2	2	38	1	163.1	96	36.08804	119.2	0.578
101415	4.25	4	2.04	5.393483	39.1632	2.920511	0.65972	5.12	5	0.38	2	3	1	2	2	0	2	2	38	3	160.2	53.2	20.72939	76.3	0.61
96723	0.91	3	1.86	4.9821	35.379	2.03886	0.50844	4.01	4.8	0.4	2	3	1	2	2	0	1	2	38	3	163.2	51.9	19.4862	73.8	0.67
98036	4.048633	5	1.11	5.456977	88.63608	3.71472	1.638	5.69	5.4	1.14	2	1	1	2	2	0	2	2	38	6	155.6	79.3	32.75322	97.6	0.67
98470	1.0658	1	1.45	5.591985	81.846	3.130788	1.11968	5.15	5.9	0.58	2	1	1	2	2	0	2	2	38	2	162.4	76.5	29.0061	93.3	0.69
94361	1.554	3	1.24	5.4328	100.642	2.15676	0.987353	3.85	5.4	6.71	2	3	1	2	2	0	1	2	38	1	153.5	78.2	33.18868	99.9	0.69
101229	1.45	4	1.24	5.27	74.16	3.439	0.858	5.07	5.4	1.32	2	1	1	2	2	0	2	2	38	1	151.6	74.5	32.41588	107.2	0.692
94488	0.68	2	1.16	6.05	43.86	2.948	1.095	4.6	5.8	1.71	2	1	1	2	2	0	2	2	38	2	159.3	67.1	26.4418	93.5	0.73
98351	2.92	4	1.86	5.163978	41.87792	1.909703	0.560425	3.88	5.5	2.79	2	3	1	2	2	0	1	2	38	1	166.4	71.1	25.6781	90.2	0.73
102196	5	5	1.53	5.3302	64.9008	2.833022	0.775186	4.71	5	0.57	2	3	2	2	2	0	2	2	38	3	158.4	65.2	25.98587	85.5	0.75
98594	3.85	5	1.01	5.3458	113.1864	2.8259	1.20712	4.45	5.1	4.26	2	1	1	2	2	0	2	2	38	1	160.8	111.1	42.96769	120.6	0.768
100967	1.24	4	1.4	5.1574	67.9278	2.66709	0.7221	4.45	5.2	1.56	2	1	1	2	2	0	1	2	38	3	158.2	69.9	27.92957	91	0.77
95911	1.39	1	1.53	5.598538	65.2332	3.000906	0.91054	4.89	5.4	1.7	2	1	1	2	2	0	2	2	38	1	155.5	61.8	25.55805	81.7	0.785
94021	0.09	1	1.47	5.16	40.02	1.862	0.485	3.57	4.9	6.64	2	1	2	2	2	0	2	2	38	3	159.8	88	34.4611	103	0.81

100713	3.92	4	1.68	4.77	21.42	2.327	0.587	4.27	5.6	1.99	2	2	1	2	2	0	2	2	38	3	164.6	67.6	24.95095	87.2	0.814
99951	2.03	4	0.88	5.3918	119.2308	2.66356	1.27127	4.4	5.1	7.4	2	1	1	2	2	0	2	2	38	2	157.9	67.4	27.03309	80.1	0.837
95251	1.22	2	0.85	5.97705	126.9214	3.35014	2.43521	5.46	5.5	4.93	2	2	1	2	2	0	2	2	38	3	162	106.6	40.61881	128.6	0.837
100476	5	5	1.11	5.623311	76.83	2.851640	1.18668	4.5	5.5	1.31	2	3	1	2	2	0	2	2	38	6	158.9	56.9	22.53536	82.5	0.86
96829	2.037280	4	1.22	5.259433	92.8188	3.18274	1.26748	5.09	4.9	2.19	2	1	1	2	2	0	2	2	38	3	165.3	88.8	32.4988	108.6	0.87
102254	0.6	3	2.25	6.05	50.34	3.595	1.016	6.31	5.9	5.34	2	1	1	2	2	0	2	2	38	1	155.7	67.7	27.92617	96.4	0.9
94666	1.14	4	0.93	5.676	117.9485	3.1208	1.73296	5.02	6	0.93	2	1	1	2	2	0	2	2	38	4	176.8	98.6	31.54368	101.2	0.913
94684	1.02	3	1.29	5.9826	135.1152	2.90564	1.11864	4.78	6	7.06	2	1	1	2	2	0	2	2	38	3	164.7	79.2	29.19698	99.2	0.95
98284	1.27	4	0.98	5.6264	128.0544	2.794046	1.31886	4.42	5.3	13.54	2	1	1	2	2	0	1	2	38	3	165.2	146.6	53.71726	133.6	0.961
96996	2.29	2	1.55	5.107252	62.9634	2.908033	0.9947	4.84	4.9	11.43	2	1	1	2	2	0	2	2	38	2	161.3	79	30.36396	98.5	0.98
101250	4.76	5	1.16	5.601466	81.9128	2.73712	1.11796	4.29	5.4	0.81	2	3	1	2	2	0	2	2	38	3	171.1	92.5	31.5967	97.8	1.08
97424	1.19	4	1.01	5.890466	161.0082	2.971365	1.34878	4.65	5.8	9.32	2	1	1	2	2	0	1	2	38	3	161.9	99.8	38.07473	106.3	1.68
102359	2.578533	3	1.76	5.33	29.4	2.741	0.677	4.81	5.2	0.78	2	3	1	2	2	0	2	2	39	6	151.8	55.1	23.91157	76	0.406
98564	4.22	5	2.12	5.291983	41.7025	2.406332	0.54892	4.6	5.226	0.42	2	3	1	2	2	0	2	2	39	3	168.3	68.5	24.18368	87	0.46
96810	2.89	5	1.32	5.66	49.56	2.612	0.677	4.24	4.7	0.5	2	3	2	2	2	0	2	2	39	6	153.1	65.1	27.7735	85.4	0.49
101412	3.67	5	1.6	5.11	29.94	2.095	0.79	4.06	5.2	0.38	2	3	1	2	2	0	2	2	39	6	155.5	47.6	19.68549	69.7	0.519
97782	2.555778	4	1.86	4.27	40.32	2.534	0.519	4.63	5.1	0.42	2	3	1	2	2	0	2	2	39	4	171	72.6	24.82815	90.2	0.57
96365	1.997	4	1.76	5.2914	80.7394	3.4622	0.991585	5.61	5.3	9.2	2	3	1	2	2	0	2	2	39	3	165.6	71.7	26.14559	90.6	0.6
97275	1.07	3	2.3	5.05	14.4	1.552	0.181	3.93	5.3	0.27	1	1	1	2	2	1	1	2	39	4	172.6	62.2	20.87896	78.8	0.614
101097	0.51	1	1.14	5.27	215.04	3.543	3.263	6.18	5.7	5.41	2	2	1	2	2	0	2	2	39	2	155.3	76.4	31.67747	111.1	0.62
102913	5	5	1.63	5.975511	113.6712	2.4531	1.097173	4.24	6.2	5.65	2	3	1	2	2	0	2	2	39	3	156.9	94.1	38.22467	118.3	0.644
96574	3.06	4	1.78	5.38	31.5	2.793	1.061	5.07	4.9	0.58	2	3	1	2	2	0	2	2	39	3	155.3	58.1	24.0898	86	0.66
95422	4.47	5	1.55	5.38	61.74	2.715	0.485	4.5	5.1	1.31	2	3	1	2	2	0	2	2	39	3	170.8	95.2	32.63332	104.6	0.667
99105	5	5	0.93	5.874911	109.6728	2.189356	1.22352	3.41	6	0.51	2	3	1	2	2	0	1	2	39	6	164.6	63.9	23.58529	84.1	0.67
99246	4.74	5	1.27	5.83	57.84	3.207	0.835	4.86	5.4	1.34	2	1	1	2	2	0	2	2	39	4	155	75.3	31.34235	103	0.68
99808	1.125175	1	1.24	5.27	49.14	3.491	2.348	5.82	5.5	2.75	2	1	1	2	2	0	2	2	39	2	158.7	77	30.57292	102.9	0.68
93989	5	5	1.55	5.275421	45.2512	2.721842	0.82071	4.71	5.1	0.99	2	3	1	2	2	0	2	2	39	3	163.1	56.8	21.35209	80.5	0.71
95165	4.57	4	1.824103	5.584	43.687	2.848422	0.737646	4.859808	5.5	2.247533	2	3	1	2	2	0	2	2	39	4	161.3	71.5	27.4813	88.3	0.72
96191	1.47	3	1.06	12.71589	264.8928	2.94834	1.716415	4.86	10.6	11.82	2	3	1	1	1	0	2	2	39	1	152	76.1	32.93802	107.2	0.73
102315	4.78	5	1.42	5.14095	70.22364	2.336721	0.982646	4.14	4.8	5.56	2	3	2	2	2	0	1	2	39	6	153.8	50	21.13768	74.8	0.737
99961	4.94	5	2.66	6.11	19.44	4.577	0.96	7.68	4.9	0.42	2	2	1	2	2	0	1	2	39	6	164	65.4	24.31588	87.9	0.738
101294	1.06	4	1.4	5.666	96.5961	3.1844	1.15246	5.2	5.7	8.69	2	1	1	2	2	0	1	2	39	4	161.6	95.3	36.49305	122.2	0.77
99939	2.43	4	1.55	5.15998	60.65076	2.631721	0.56834	4.4	5.5	2.29	2	1	1	2	2	0	2	2	39	4	160.5	62.1	24.10691	81.7	0.83
98208	4.07	5	1.71	5.94	46.68	3.077	1.874	5.64	5.1	0.79	2	3	1	2	2	0	2	2	39	3	168.1	65.7	23.25037	95.8	0.836
102345	4.9	5	2.17	5.584933	60.5166	2.8262	0.75572	4.89	5.9	1.9	2	3	1	2	2	0	2	2	39	4	165.2	113.1	41.44217	112.8	0.841
94405	0.78	2	1.29	5.313	78.79348	2.409898	0.802526	4.16	4.7	2.47	2	1	2	2	2	0	2	2	39	3	159.1	78.6	31.05147	94.3	0.85
96490	5	5	1.4	6.349111	155.3148	2.97312	1.624722	5.07	6.3	5.6	2	3	1	1	2	0	2	2	39	6	157	54.3	22.02929	74	0.857
94398	0.81	2	1.24	5.8864	89.2888	2.234784	1.13618	3.93	6.1	3.13	2	1	1	2	2	0	2	2	39	4	167.2	84.7	30.29778	92.1	0.858
94028	4.43	5	1.99	5.1996	34.659	2.05684	0.480119	4.01	4.8	0.78	2	3	1	2	2	0	1	2	39	2	166.8	60.8	21.85302	82.8	0.861
95257	3.47	4	1.68	5	36.06	3.853	0.316	5.69	5.7	4.97	2	3	1	2	2	0	1	2	39	4	157.5	66.7	26.88838	78.6	0.879
99616	2.01	4	1.03	7.6	377.4	1.629	0.576	2.92	5.5	19.24	2	3	1	2	1	0	2	2	39	3	168	179.2	63.49206	162.3	0.88
99098	0.8698	2	1.06	5.5454	102.9216	3.89814	1.8309	6.31	6	3.37	2	1	1	2	2	0	2	2	39	1	169.1	83.1	29.06122	105.7	0.883
94387	4.12	3	1.4	6.55	123.9	3.543	1.129	5.46	5.8	8.37	2	3	1	2	2	0	2	2	39	4	163.5	131.9	49.34115	146.3	0.89
100024	2.28	3	1.22	5.399833	70.49493	2.930581	1.126766	4.89	5.3	0.92	2	1	1	2	2	0	2	2	39	3	170.1	91.2	31.52	104.2	0.903
101718	0.8	4	1.14	6.11	44.4	3.077	0.519	4.45	5.2	1.02	2	1	1	2	2	0	2	2	39	3	154.5	85.2	35.69297	121.5	0.92
95348	2.65	4	0.88	6.72	151.14	2.819	1.682	4.47	6.3	6.43	2	3	1	1	3	0	2	2	39	2	163.8	89.4	33.32036	101.2	0.93
99517	2.64	4	1.16	5.25855	73.6128	2.13604	0.744166	3.57	5.3	5.42	2	3	1	2	2	0	1	2	39	1	161.6	82.2	31.47669	109.9	0.948
102668	0.66	4	1.06	5.5362	91.6676	2.77759	1.2351	4.53	5.2	3.2	2	1	1	2	2	0	1	2	39	3	159.5	45	17.68851	70	0.99
100738	1.88	4	1.6	6.1952	125.3106	2.655105	1.10546	4.53	6.3	17.07	2	1	1	2	2	0	1	2	39	2	158.5	99.5	39.60633	116.5	0.99
102132	0.04	2	1.01	5.6736	94.0992	2.31454	1.34864	3.65	5.4	19.79	2	1	1	2	2	0	2	2	39	1	157	101.1	41.01586	124.7	0.99
94131	3.05	4	1.5	8.161600	184.8024	2.758906	1.70966	4.65	6.7	6.93	2	3	1	1	1	0	2	2	39	3	169.1	129	45.11308	135.5	1.02
99848	0.59	4	1.55	5.400733	49.43582	2.73502	0.838798	4.58	5.3	3.52	2	1	1	2	2	1	2	2	39	3	170.9	69.4	23.76158	95.1	1.03
100356	0.74	3	2.15	5.3698	70.1158	2.710556	0.83228	4.97	5.4	2.53	2	1	1	2	2	0	1	2	39	4	173.7	92.7	30.72417	97.3	1.08
97726	0.73	4	1.34	5.3502	76.8312	2.52709	0.745451	4.24	5.1	6.14	2	1	2	2	2	0	2	2	39	1	155	70.4	29.30281	103.2	1.18
95353	0.4	4	1.03	6.94	39.6	1.371	0.881	2.82	6.4	7.9	2	1	1	2	2	0	1	2	39	1	166.4	100.1	36.15159	102.5	1.22
94972	1.5	3	0.88	5.55	55.44	2.25	1.344	3.75	5.2	7.53	2	1	1	2	2	0	2	2	39	3	171.6	90.6	30.7676	104.8	1.25
97129	5	5	1.58	5.306166	49.8608	2.692708	0.61224	4.6	5.4	0.84	2	3	1	2	2	0	2	2	40	6	165.5	61.4	22.41674	78.3	0.423
98045	0.58	5	2.12	5.1426	44.0424	2.228753	0.50766	4.22	5.7	0.87	2	3	1	2	2	0	2	2	40	6	160.9	64.6	24.95287	82.2	0.47
100451	1.79	4	1.81	5.55	43.8	2.043	0.711	4.19	5.3	0.83	2	3	1	2	2	0	1	2	40	6	155.9	58.7	24.1516	80.8	0.522
102635	5	5	1.86	5.22	23.34	1.681	0.452	3.75	5	0.93	2	3	1	2	2	0	2	2	40	6	166.1	59.7	21.63889	76.7	0.525

100266	0	4	1.86	5.5234	71.022	3.714613	1.20913	6.15	5.4	0.28	2	2	1	2	2	0	2	2	40	3	164.6	49.8	18.38102	72.6	0.534
93849	5	5	1.22	5.3914	67.49876	2.182613	1.01154	3.96	5.2	8.89	2	3	1	2	2	0	1	2	40	3	164.6	61.4	22.66255	84.4	0.54
96222	0.73	4	1.78	5.22	23.16	1.81	0.632	3.88	5.4	0.88	2	1	1	2	2	0	1	2	40	3	161.4	68.4	26.25724	88.9	0.558
100929	2.5894	3	2.28	5.5	66.66	2.379	0.677	4.97	5.3	1.35	2	3	1	2	2	0	2	2	40	4	172.8	107.1	35.86757	111	0.58
93913	3.824733	5	2.48	5.3538	38.0571	2.940855	0.69576	5.59	4.9	0.32	2	3	1	2	2	0	2	2	40	6	160.3	63.7	24.78976	96.9	0.618
102378	0.91	2	0.96	5.490652	121.1338	3.061815	1.30732	4.81	5.4	2.54	2	1	1	2	2	0	2	2	40	1	155.4	69.9	28.94511	97.4	0.63
94660	5	4	2.04	5.338248	64.4868	4.0579	1.169585	6.83	5.1	2.78	2	3	2	2	2	0	2	2	40	1	157.4	81	32.69456	101.8	0.71
99168	4.132233	5	2.17	5.2498	54.6178	3.09025	0.918026	5.53	5.2	5.3	2	3	1	2	2	0	2	2	40	2	152.7	53.5	22.94435	76.6	0.788
101795	0.81	3	0.96	5.366	68.34653	2.134778	1.00712	3.88	4.7	1.88	2	1	1	2	2	0	1	2	40	1	153.8	63.2	26.71803	94.5	0.83
101649	3.59	5	1.24	5.38	49.74	3.931	1.027	5.64	5.5	1.11	2	3	1	2	2	0	2	2	40	3	167.6	74.5	26.52212	91.4	0.89
98714	3.25	3	1.71	5.4753	87.8236	3.45885	1.20834	5.56	5.6	6.1	2	3	1	2	2	0	2	2	40	1	161.3	110.3	42.39423	118.5	0.916
100178	1.56	3	1.09	5.3324	81.132	3.74762	2.08862	6	5.5	3.63	2	1	1	2	2	0	2	2	40	3	155.8	77.6	31.96884	104.8	1.01
97598	1.704526	3	1.4	5.66	32.94	2.379	0.948	4.22	5.5	3.53	2	1	1	2	2	0	2	2	40	1	158	84	33.64845	101.1	1.02
94967	5	5	1.55	5.183010	45.8196	2.433675	0.791392	4.29	5.1	6.53	2	3	1	2	2	0	2	2	40	4	163.5	93	34.78944	97.6	1.02
98783	1.49	4	1.53	8.0042	178.8476	2.771226	1.765	4.86	6.8	6.02	2	3	1	1	1	1	1	2	40	3	169.1	126.3	44.16885	131.3	1.09
96622	1.69	4	1.27	6.11	275.34	2.431	0.768	4.06	5.9	14.73	2	3	1	2	2	0	1	2	40	4	159.6	120.1	47.14952	131.1	1.15
100924	1.301684	3	0.91	5.1532	126.5716	3.237428	1.57474	4.97	4.7	11.62	2	1	1	2	2	0	2	2	40	3	168.3	127.1	44.8722	115.6	1.16
93939	2.97	4	1.14	5.99572	136.0296	2.69776	1.25998	4.42	5.8	12.43	2	3	1	2	2	0	1	2	40	4	153.3	67.7	28.80742	91.7	1.18
97643	1.32	5	1.42	5.05	101.16	3.077	1.581	5.22	5.8	13.65	2	1	1	2	2	0	1	2	40	3	164.1	104.9	38.95456	121.9	1.2
96505	0.59	3	1.37	4.88	102.84	2.741	0.971	4.55	5.2	6.75	2	1	1	2	2	0	2	2	40	7	168	120.8	42.80045	133.4	1.22
97602	1.14	4	1.09	5.94	80.94	2.586	1.027	4.14	5.7	6.39	2	1	1	2	2	0	1	2	40	4	173.8	92.3	30.5564	101.8	1.44
98131	2.8716	4	1.09	6.1578	182.8092	2.62432	1.31326	4.22	6	53.83	2	3	1	2	2	0	2	2	40	4	157.3	95.3	38.51549	117.5	1.52
102473	5	4	1.63	5.315044	48.6996	1.930854	0.521745	3.57	5.5	1.03	2	3	1	2	2	0	1	2	41	2	154	62.5	26.35352	80.6	0.52
102954	1.18	5	1.4	5.22	56.94	2.793	0.553	4.45	5.2	0.81	2	1	1	2	2	0	2	2	41	4	162.6	69.1	26.13587	86.4	0.534
96465	1.56	4	2.09	5.61	32.94	2.767	0.485	5.09	5.3	0.93	2	3	1	2	2	0	2	2	41	6	160.3	56.2	21.87103	76.7	0.553
99659	1.49	3	1.45	5.83	47.7	2.405	0.768	4.22	4.9	0.54	1	1	1	2	2	0	2	2	41	6	158	61.5	24.63548	85.9	0.578
95275	5	5	1.45	5.27	31.44	3.258	1.231	5.28	4.7	1.17	2	3	1	2	2	0	1	2	41	7	160.9	55.4	21.39921	80.7	0.58
100841	5	5	2.22	5.4054	45.7746	3.44587	0.8782	6.1	5.4	0.85	2	3	1	2	2	0	2	2	41	6	160.9	56.6	21.86273	80.4	0.607
93814	5	5	1.29	5	55.62	1.888	0.553	3.44	4.6	0.86	2	3	1	2	2	0	2	2	41	4	168.9	79.3	27.79802	90.7	0.63
101037	3.66	4	1.71	5.66	59.76	3.233	0.847	5.33	5.6	2.01	2	3	1	2	2	0	2	2	41	3	155.1	106.8	44.39639	122.2	0.67
94277	0.62	1	1.11	5.11	28.32	3.724	1.355	5.46	5.2	1.12	2	1	1	2	2	2	2	2	41	2	151.2	64.6	28.25719	103.2	0.722
102811	0.96	3	1.68	5.072428	33.8304	1.955	0.662673	3.9	5.1	1.89	2	3	1	2	2	0	1	2	41	1	164.4	65.8	24.3457	89.8	0.724
99438	0.89	3	1.27	6	52.98	4.086	2.473	6.49	5.9	1.74	2	1	2	2	2	0	2	2	41	6	153.8	69	29.17	90.1	0.731
95610	5	4	1.45	5.327	67.55584	3.68411	1.042946	5.69	5.2	1.89	2	3	2	2	2	0	2	2	41	2	151.6	68.1	29.63116	92.1	0.733
101426	1.82	4	2.04	5.183	45.12	2.036486	0.576575	3.75	5.7	4.87	2	3	1	2	2	0	1	2	41	4	168	124.9	44.25312	119.7	0.74
99893	1.64	4	1.34	5.13	68.0544	2.49106	0.76954	4.32	5.3	2.04	2	1	1	2	2	0	1	2	41	4	159	76.9	30.4181	98.9	0.77
94935	2.29	2	0.96	5.478852	87.52868	3.318315	1.41618	5.15	5.4	1.95	2	1	1	2	2	0	2	2	41	7	163.6	77.2	28.84368	100	0.82
95284	0.34	4	1.09	6.55	122.88	3.026	1.524	4.81	5.3	3.98	2	1	1	2	2	1	2	2	41	2	153.9	87.3	36.85844	100.2	0.84
94889	2.72	1	1.4	5.6788	89.2236	2.371458	0.95202	4.16	5.4	6.09	2	3	1	2	2	0	2	2	41	1	148.5	82.2	37.27511	102.1	0.843
98266	1.667986	4	1.4	5.4813	80.71584	3.45494	1.103173	5.4	5.6	0.78	2	2	1	2	2	0	2	2	41	2	161	93	35.87825	110.7	0.85
98601	1.22	4	1.01	5.8274	151.7988	3.61282	1.52208	5.48	6.2	5.38	2	1	1	2	2	0	1	2	41	4	159	74.7	29.54788	90.6	0.885
95171	1.67	3	1.06	6.33	125.52	1.81	1.592	3.59	7	3.09	2	3	1	1	3	0	1	2	41	3	168.5	114	40.1518	126.8	0.93
97373	1.13	4	0.88	5.5726	89.01	3.77296	1.91014	5.87	5.5	2.35	2	1	1	2	2	0	2	2	41	3	159.3	86.7	34.16548	116.6	0.944
94501	4.08	4	1.42	5.832	89.235	2.777423	1.058533	4.6	6	4.11	2	3	1	2	2	0	1	2	41	6	162.8	93.5	35.27791	101.1	0.95
93907	2.49	3	0.88	5.469933	70.46	2.10142	1.144046	3.83	5	2.92	2	3	1	2	2	0	1	2	41	4	173.7	80.6	26.71378	91.6	0.986
94808	1.46	4	1.14	6.0404	134.0884	3.28042	1.57082	5.38	6.1	9.26	2	1	1	2	2	0	1	2	41	4	163.5	86.7	32.43274	102.7	0.99
98336	1.57	5	1.24	6.55	137.88	1.836	0.756	3.41	6.2	9.07	2	3	1	2	2	0	2	2	41	4	178	110.1	34.7494	120.6	0.998
93957	1.99	4	0.93	7.16	104.4	3.853	1.795	5.61	5.9	3.76	2	3	1	1	1	0	1	2	41	3	166.5	80.7	29.11019	97.5	1.11
93774	3.67	4	1.4	5.422266	73.1136	3.19108	1.141123	5.17	5.5	3.67	2	1	1	2	2	0	2	2	41	3	165.5	70.9	25.88512	90	1.24
101342	2.55	5	1.32	5.77	87	2.146	1.163	4.01	5.2	11.31	2	3	2	2	2	0	1	2	41	3	167.7	159.9	56.85679	148.6	1.25
99019	2.49	3	1.09	6.88	128.58	3.051	0.881	4.55	6.1	10.98	2	1	1	2	2	0	2	2	41	1	168.4	131.9	46.51153	143.5	1.28
101430	4.08	5	2.48	4.83	14.58	2.483	0.463	5.17	5.5	0.8	2	2	1	2	2	0	2	2	42	6	162	56.4	21.49063	71.6	0.461
98033	2.37	4	1.34	4.83	47.4	2.922	0.711	4.6	5.4	0.64	2	1	1	2	2	0	2	2	42	4	168.3	76	26.83153	85.6	0.47
93843	5	5	2.28	5.44	23.16	2.715	0.632	5.28	5.4	0.55	2	2	1	2	2	0	1	2	42	2	163.8	56.3	20.98363	75	0.479
100216	5	4	1.97	5.210333	48.19283	2.613536	0.635945	4.81	5.2	1.03	2	3	1	2	2	0	2	2	42	1	158	52.8	21.15046	74	0.53
96329	1.92	2	1.71	5.61	35.22	1.474	0.474	3.39	5.4	0.56	2	1	1	2	2	0	2	2	42	1	166.1	64.5	23.3787	77.2	0.535
96067	1.08	4	1.27	5.343366	81.3192	3.552692	1.16912	5.72	5.1	4.14	2	1	1	2	2	0	1	2	42	3	159	143	56.56422	134.2	0.578
100172	5	5	1.34	5.606377	83.30488	3.9638	1.190053	6.13	5.4	1.75	2	3	2	2	2	0	2	2	42	3	162.1	83.1	31.62532	99.3	0.59
95362	1.5	2	1.66	5.430628	52.35556	3.092217	0.79762	5.22	5.1	0.89	2	3	1	2	2	0	2	2	42	1	150.9	57	25.03205	81.8	0.6
95280	2.1776	3	1.71	5.228166	57.708	2.499617	0.673728	4.45	5.4	1.65	2	3	1	2	2	0	2	2	42	3	153.5	73.1	31.0242	92.1	0.6

99935	3.88	2	1.63	4.55	59.88	3.258	0.587	5.15	5.1	1.77	2	3	1	2	2	0	2	2	42	1	170.2	91	31.41393	93.9	0.61
96240	1.35	4	0.98	4.94	121.44	3.75	1.434	5.38	5.3	2.84	2	1	1	2	2	0	1	2	42	6	157.4	69.2	27.93165	92.1	0.655
96416	0.96	2	1.76	5.0829	36.5268	2.2101	0.56292	4.01	5	1.24	2	3	1	2	2	0	2	2	42	3	172.4	69.8	23.48448	92.2	0.7
100983	3.43	4	1.34	5.787777	80.84304	3.087066	0.89638	4.91	5.7	1.93	2	3	1	2	2	0	2	2	42	6	154.5	72.1	30.20496	96.5	0.77
101014	0.63	3	1.27	5.55	35.64	3	0.96	4.71	5.3	1.66	2	1	1	2	2	0	2	2	42	3	170	59.1	20.44983	78.5	0.78
95140	4.07	4	2.56	5.3061	47.4372	3.246781	1.0839	5.79	5.1	1.27	2	1	1	2	2	1	2	2	42	1	163	79.2	29.80918	93.7	0.792
98878	1.04	2	1.34	5.320914	68.09228	2.43555	0.76025	4.19	5.2	1.2	2	1	1	2	2	0	2	2	42	1	156.9	74.5	30.26289	95.7	0.8
101547	1.71	4	1.11	6.11	183.36	2.896	2.393	5.09	5.7	3.68	2	3	1	2	2	0	1	2	42	3	160.2	125.5	48.90111	128.5	0.806
94471	1.243044	1	1.11	5.597319	84.96733	3.107001	1.29176	4.94	5.6	1.84	2	1	1	2	2	0	2	2	42	2	161.3	76.5	29.40307	101.6	0.82
94771	3.23	3	2.04	5.2288	75.2976	2.9791	0.965821	5.25	5.3	8.93	2	1	1	2	2	0	2	2	42	3	164.9	69.3	25.48543	86.3	0.868
102134	0.42	3	1.14	5.5672	58.8744	2.71114	0.87834	4.32	5	0.7	2	1	1	2	2	0	2	2	42	3	164.2	79.1	29.33798	102.9	0.89
99282	0.96	4	1.03	5.625266	95.86608	3.99638	1.96828	6.31	5.7	1.75	2	1	1	2	2	0	2	2	42	4	162.9	104.8	39.49291	128.5	0.89
102369	3.0457	4	1.63	5.33	41.64	3.646	0.632	5.56	5.6	3.14	2	3	1	2	2	0	2	2	42	1	149.6	62.3	27.83716	99	0.975
95559	0.92	5	1.16	5.4472	91.18368	3.75154	1.46766	5.84	5.7	2.03	2	1	1	2	2	0	2	2	42	3	158.9	75.5	29.90193	94	1.05
102816	1.96	3	1.55	5.153633	56.2212	2.54204	0.73448	4.42	5.4	4.25	2	1	1	2	2	0	1	2	42	2	152.3	75.3	32.46349	105.2	1.12
101928	2.77	4	1.24	6.16692	146.4172	3.30138	1.53654	5.33	5.9	39.27	2	3	1	2	2	0	1	2	42	4	164.1	105.1	39.02883	121.8	1.13
96344	0.04	3	1.19	5.2554	113.2152	3.49567	1.60294	5.46	4.4	7.35	2	1	1	2	2	0	2	2	42	1	156.2	88.7	36.35478	114.8	1.18
98374	0.58	2	0.75	6.16	141.06	2.741	1.931	4.37	5.6	2.7	2	1	1	2	2	0	2	2	42	3	165.7	95	34.6002	116.4	1.2
99028	1.22	4	1.66	5.11	37.74	3.155	0.802	5.17	5.4	2.78	2	3	1	2	2	0	2	2	42	1	155.2	84.6	35.12262	98.3	1.33
98135	0.69	3	0.78	5.61	178.32	2.948	2.687	4.97	5.7	31.95	2	2	1	2	2	0	2	2	42	3	163.3	128.5	48.1871	135.7	1.37
100191	1.06	4	1.09	5.7996	143.964	2.87356	1.67408	4.76	5.3	18.61	2	2	1	2	2	0	1	2	42	3	161.8	71.2	27.19712	89.5	1.39
93813	1.11	1	1.4	5.66	65.4	3.543	1.152	5.46	5.3	30.31	2	1	1	2	2	0	2	2	42	1	155.3	104.3	43.24555	115.9	1.43
94696	2.06	4	1.24	5.05	31.2	2.483	0.632	4.01	5.2	0.48	2	2	1	2	2	0	2	2	43	3	158.4	55.6	22.15973	79.2	0.49
101885	1.81	5	1.78	5.16	18.24	2.767	0.711	4.89	5.4	0.43	2	3	1	2	2	0	2	2	43	3	172.5	63.2	21.23924	80.4	0.52
98631	1.67	2	1.86	5.3822	46.65496	2.943656	0.64995	5.09	5	1.25	2	3	1	2	2	0	2	2	43	3	163.3	69.1	25.91228	81.9	0.658
100013	3.855	5	0.98	5.61	75.18	3.258	2.281	5.28	5.1	1.42	2	1	1	2	2	0	2	2	43	6	153.6	59	25.00746	86.2	0.679
94706	3.73	5	1.66	5.297913	47.1328	2.726796	0.65442	4.63	5.5	0.68	2	3	1	2	2	0	2	2	43	6	161.6	70.8	27.11131	95.2	0.68
93847	0.1	2	1.4	6.38	71.04	1.552	0.553	3.21	5.8	3.44	2	1	1	2	2	0	2	2	43	1	156.6	100.4	40.94022	111.1	0.715
95436	1.74	3	1.78	5.05	42.84	2.767	0.497	4.78	5.5	0.95	2	3	1	2	2	0	2	2	43	1	156.5	58.8	24.00759	80.7	0.717
94512	1.22	2	1.55	9.38	99.12	2.353	1.806	4.73	7.9	5.32	2	1	1	1	1	1	2	2	43	1	155.4	61.2	25.3425	83.7	0.72
100627	0.12	3	1.29	5.88	25.44	2.845	0.937	4.58	5.4	1.42	2	1	1	2	2	0	2	2	43	7	147.1	42.2	19.50236	68.7	0.74
95278	2.95	2	0.98	7.83	325.5	3.129	1.163	4.65	6.5	4.21	2	3	1	2	1	0	2	2	43	2	168.1	93.7	33.15921	105	0.771
99495	2.08	4	1.55	5.2795	74.2104	3.082376	1.03604	5.3	5.1	1.67	2	1	1	2	2	0	1	2	43	3	155.8	49.2	20.2689	68.8	0.809
98646	0.76	1	1.11	5.69412	108.3516	4.2502	2.69862	6.9	5.4	8.36	2	2	1	2	2	0	1	2	43	1	154	72.9	30.73874	102.3	0.83
99160	2.16	4	1.22	5.72	112.8	2.043	0.835	3.65	5.1	1.53	2	3	1	2	2	0	1	2	43	7	165.9	83.1	30.19314	92.4	0.849
101007	1.02	3	1.06	5.514	94.8612	3.93642	1.95158	6.23	5.8	3.54	2	1	2	2	2	0	2	2	43	3	159.1	78.7	31.09098	99.9	0.884
101549	5	5	0.93	5.3158	73.6476	2.18882	0.946668	3.39	4.7	1.93	2	3	2	2	2	0	1	2	43	6	159.1	64.4	25.44166	83.5	0.89
94764	3.4	4	0.72	5.867977	156.6842	3.39274	2.03732	5.48	5.9	2.85	2	3	1	2	2	0	2	2	43	4	169.5	119.3	41.52418	119.2	0.94
98161	1.08	4	1.58	5.38	36.78	4.163	1.016	6.21	5.2	5.51	2	1	1	2	2	0	1	2	43	3	161	92.1	35.53104	111.1	0.964
100137	1.79	2	1.11	5.5556	83.69348	2.434698	0.96698	4.06	5.5	1.42	2	1	1	2	2	0	2	2	43	2	158.3	89.7	35.79568	103.8	0.98
100200	0.87	4	1.16	5.83	152.94	4.551	1.095	6.21	5.8	4.26	2	1	1	2	2	0	1	2	43	7	162.7	104.5	39.47673	114.6	0.992
101594	4.41	4	1.76	5.141164	47.85923	3.131973	1.003646	5.38	4.8	1.94	2	3	1	2	2	0	1	2	43	3	163	74.5	28.0402	92.5	1.03
95595	1.04	2	1.14	6.2566	150.6336	2.860144	1.34836	4.63	6.1	15.25	2	1	1	2	2	0	2	2	43	3	163	135.6	51.03692	137.2	1.03
94223	0.94	4	0.91	6.83	93.48	3.051	2.935	5.3	5.6	9.43	2	2	1	2	2	0	2	2	43	2	167	81.8	29.33056	92.8	1.09
100288	1.15	2	1.32	5.94	225.12	3.077	1.016	4.86	5.8	7.54	2	1	1	2	2	0	2	2	43	6	162.1	106.2	40.41648	140.4	1.09
95029	1.44	4	1.34	5.11	142.5	3.672	1.14	5.53	5.4	0.67	2	1	1	2	2	0	1	2	43	4	165.5	90.2	32.93143	106.3	1.14
99496	0.33	3	0.96	10.90429	152.586	2.40606	1.50154	3.67	8.2	5.34	2	1	1	1	1	1	2	2	43	4	151.1	57.9	25.36002	91.2	1.17
102923	0.95	3	1.01	6.83	175.44	2.327	1.84	4.19	5.9	14.96	2	1	1	2	3	0	2	2	43	3	173.9	106.1	35.08457	128	1.2
102181	1.14	5	3.9	5.88	38.28	2.25	1.468	6.83	4.9	0.84	2	2	1	2	2	1	1	2	43	4	162.3	84.5	32.07891	106.5	1.32
97923	0.79	3	2.15	5.1346	39.1812	2.489471	0.74972	4.71	4.4	0.48	2	3	1	2	2	0	2	2	43	3	160.5	50	19.40975	79.9	1.41
100816	1.22	1	1.16	8.5904	125.484	2.830246	1.629335	4.94	7	4.36	2	3	1	1	1	1	2	2	43	3	170.8	93.3	31.98202	110.4	1.71
98616	5	5	1.81	5.5888	47.08824	3.55337	0.740857	5.79	5.4	0.28	2	3	1	2	2	0	2	2	44	6	154.5	46.2	19.35464	74	0.439
101544	4.62	4	1.81	5.123998	37.5744	2.10185	0.574581	4.01	5	0.46	2	3	1	2	2	0	1	2	44	3	162.5	71.2	26.96331	85	0.452
96847	2.78	3	1.78	5.40505	48.6564	2.6995	0.622023	4.58	5.8	0.33	2	3	1	2	2	0	2	2	44	4	161	53.4	20.60106	74.2	0.488
96547	5	5	1.99	5.216554	47.1988	2.500371	0.641845	4.63	5.2	1.98	2	3	1	2	2	0	2	2	44	3	162.3	79.7	30.25668	104	0.51
101065	0.8	3	1.66	6.16	87.12	4.422	0.982	6.52	5.4	0.5	2	1	1	2	2	0	2	2	44	1	172.6	71.1	23.86646	88.3	0.546
97974	1.97	2	1.4	5.77	41.58	4.189	1.321	6.18	5.5	1.53	2	3	1	2	2	0	2	2	44	6	141.9	49.3	24.48399	79.5	0.552
99691	5	5	2.09	5.260136	51.13382	2.520515	0.7461	4.68	5.3	5.38	2	3	1	2	2	0	2	2	44	1	166	64.9	23.55204	80.4	0.57
102151	1.67	4	1.66	5.61	73.07756	2.56	0.305	4.34	5.7	6.8652	2	3	1	2	2	0	2	2	44	6	161.4	58.2	22.34168	85.5	0.594
102642	5	5	1.09	8.27	104.58	3.543	1.095	5.12	7.3	3.11	2	3	1	1	1	0	2	2	44	6	164.4	76.6	28.34165	93.3	0.61

95391	0.01	1	1.71	6.38	66.18	2.974	1.039	5.15	5.4	2.62	2	1	1	2	2	0	2	2	44	1	145.5	55.8	26.35774	89.5	0.658
95662	3.01	5	1.22	5.5342	115.1668	3.71261	1.4199	5.9	5.4	3.86	2	3	1	2	2	0	1	2	44	3	151.2	65.2	28.51964	93	0.664
96408	5	5	2.02	5.4544	132.3362	2.788793	1.015713	4.63	4.9	34.38	2	3	1	2	2	0	1	2	44	4	142.7	90.8	44.59004	127.8	0.67
101596	4.32	5	1.47	5.450233	44.2086	1.678357	0.520143	3.08	5.2	0.68	2	3	1	2	2	0	2	2	44	4	150.3	53.7	23.77149	80.4	0.68
100733	3.04	4	1.14	5.72	104.76	2.715	2.269	4.89	6.2	6.7794	2	3	1	2	2	0	1	2	44	6	160.8	86.9	33.60839	112.5	0.68
94731	2.03	4	1.09	5.4184	71.41973	2.029518	0.973435	3.75	5	0.63	2	3	1	2	2	0	1	2	44	4	172.6	79.2	26.58542	98.9	0.699
95210	5	4	1.55	6.11	72.06	3.724	0.756	5.61	5	3.55	2	3	2	2	2	0	2	2	44	3	174.9	86	28.11375	101.2	0.723
95652	5	4	1.86	5.05	25.5	2.095	0.768	4.32	5.3	3.87	2	3	1	2	2	0	1	2	44	1	159.7	58.6	22.97671	86	0.766
99300	1.83	4	1.06	5.11	39.36	3.931	1.411	5.64	5	2.17	2	1	1	2	2	0	1	2	44	2	159.3	70.1	27.62399	87.5	0.767
102434	5	3	2.09	5.4365	54.20608	3.281395	0.777521	5.43	5.2	2.72	2	3	1	2	2	0	2	2	44	4	165.3	111.1	40.66009	123.8	0.773
97121	0.93	4	1.4	5.1784	72.8404	2.63307	0.69391	4.42	5	2.54	2	3	2	2	2	0	1	2	44	7	170.5	101.8	35.01862	107.6	0.787
101965	3.39	5	0.67	5.725577	146.2551	2.877210	1.62404	4.76	5.7	1.15	2	3	1	2	2	0	2	2	44	6	150	64.1	28.48889	86.4	0.8
97841	5	4	1.71	5.472	57.02128	3.397736	0.770033	5.46	5.5	2.66	2	3	1	2	2	0	2	2	44	3	165.5	91.6	33.44256	96.1	0.802
97524	1.15	5	1.32	5.337733	54.7772	2.878553	0.705633	4.55	5.4	0.88	2	1	1	2	2	0	2	2	44	3	173.8	61.1	20.22747	83.8	0.804
97191	0.41	1	1.4	5.219866	57.1536	3.723658	1.6016	6.05	5.3	1.38	2	1	1	2	2	0	2	2	44	1	157	80.8	32.78023	94.7	0.835
102865	5	5	1.06	6.72	205.8	3.077	2.202	5.15	6.6	3.6	2	3	1	1	1	0	2	2	44	3	175	143.8	46.9551	144.3	0.855
102401	0.41	3	1.71	5.298233	61.0572	2.66092	0.8515	4.6	5.4	6.63	2	1	1	2	2	1	2	2	44	2	153.2	46.4	19.76972	80	0.888
97574	1	2	1.37	5.3754	58.5628	1.949042	0.633211	3.44	5.3	1	2	1	2	2	2	0	2	2	44	6	153.7	53.8	22.77375	79.7	0.89
101941	3.3	5	1.99	5.7578	66.096	2.91806	0.86006	5.15	6	1.47	2	3	1	2	2	1	2	2	44	4	161.8	113.4	43.31677	122.3	0.9
96882	0.32	5	1.11	7.981066	153.9744	2.48516	1.4343	4.19	6.4	7.05	2	1	1	1	1	0	2	2	44	6	146.7	76.6	35.59332	102.9	0.92
94486	0.77	4	0.91	7.632800	152.058	2.15882	1.70072	3.52	6.6	1.11	2	1	1	1	1	0	2	2	44	4	176.7	97.4	31.19506	112	0.92
95366	4.78	4	1.22	5.72	94.32	2.845	1.106	4.58	5.3	14.82	2	3	1	2	2	0	1	2	44	1	158.4	123.3	49.14199	135.2	0.928
97095	3.49	4	1.42	5.94	36.66	2.845	1.694	5.04	5.6	1.4	2	3	1	2	2	0	2	2	44	7	161.3	76	29.21089	101.8	0.94
100378	3.49	4	1.19	5.8926	101.4066	4.11024	1.6139	6.26	6.2	5.71	2	3	1	2	2	0	1	2	44	7	148.1	116.6	53.16042	122.4	0.94
102893	1.57	4	1.22	5.33	47.04	3	1.614	4.97	5.7	5.65	2	1	1	2	2	0	2	2	44	3	164.6	73.8	27.23935	95.4	0.951
97142	1.36	1	1.24	7.9758	172.2756	2.792955	1.478715	4.71	6.5	13.14	2	3	1	1	1	0	2	2	44	1	156.4	79.6	32.54165	103.8	1.02
102518	1.380114	2	1.55	5.409252	91.6984	3.45964	1.14453	5.48	5.6	7.73	2	3	1	2	2	0	2	2	44	1	152.7	91.1	39.06972	103.1	1.08
98872	3.4	5	1.76	5.48	55.37028	3.11757	0.856667	5.33	5.5	2.71	2	3	1	2	2	0	2	2	44	4	157.3	98.2	39.68753	126.6	1.15
100730	1.99	4	1.58	5.44	99.3	2.302	1.84	4.73	5.5	12.48	2	1	1	2	2	0	1	2	44	3	157.9	93.9	37.66182	110	1.2
94858	0.78	3	1.97	5.6284	59.2158	3.53502	0.98483	5.72	5.5	2.19	2	3	1	2	2	0	2	2	44	3	161	100.5	38.77165	119.8	1.21
100827	4.08	5	1.804833	4.77	40.6978	2.55913	0.685425	4.665083	5	1.325343	2	3	1	2	2	0	2	2	45	3	167	79.4	28.47001	97.7	0.44
97794	3.15	5	1.6	5.22	14.76	2.302	0.745	4.24	5.3	4.27	2	3	1	2	2	0	1	2	45	6	166.6	67.8	24.42754	81.5	0.469
100145	1.1	4	1.84	5.6282	73.8297	3.59252	1.20444	5.9	5.5	3.77	2	1	1	2	2	0	1	2	45	2	159.5	69.3	27.2403	89.8	0.597
98776	1.49	4	1.34	5.280433	81.066	3.21432	1.028653	4.99	5.1	2.92	2	1	1	2	2	0	2	2	45	3	189.3	152.6	42.58467	133.7	0.661
94047	2.72	3	0.88	7.44	300.3	3	2.235	4.91	6	2.75	2	3	1	2	1	0	2	2	45	1	162.3	93.7	35.57153	109.2	0.68
94174	1.99132	1	1.47	5.83	54.42	3.827	0.847	5.69	5.6	0.63	2	3	1	2	2	0	2	2	45	1	148.8	57.2	25.83391	83.7	0.69
94732	1.71	4	1.102533	13.5338	183.3804	4.22556	1.71052	6.1418	12.2	6.4922	2	3	1	1	1	0	2	2	45	4	176.3	84.9	27.31512	92.5	0.706
98094	1.91	3	1.4	8.119490	130.3452	3.99848	1.61048	6.34	6.7	2.62	2	3	1	1	1	0	1	2	45	1	155.7	94.2	38.85739	106.5	0.714
94565	0.1	3	1.34	5.38	154.68	3.853	1.637	5.95	5.1	2.85	2	1	1	2	2	0	2	2	45	2	168.4	94.3	33.25275	111.1	0.726
101112	4.76	3	2.33	5.7196	183.048	2.853544	1.21734	4.86	5.5	10.38	2	3	1	2	2	0	2	2	45	6	158.4	48.7	19.40969	70.8	0.74
97672	0.48	2	1.22	5.556252	112.6896	3.48962	1.38679	5.38	5.7	3	2	1	1	2	2	0	2	2	45	1	150.4	75.2	33.24468	98.6	0.76
101695	0.79	4	1.32	5.33	57.66	3.724	1.061	5.53	5.5	2.84	2	1	1	2	2	0	1	2	45	7	171.5	124.8	42.4313	132.8	0.778
97123	1.39	2	1.47	5.429761	49.0896	1.850229	0.621516	3.34	5.2	2.17	2	1	1	2	2	0	2	2	45	6	161.4	55.8	21.42038	75.5	0.781
99025	5	4	1.55	5.263	59.3478	3.119273	0.979366	5.17	5.4	1.14	2	3	1	2	2	0	2	2	45	3	159.5	56	22.01236	79	0.8
95680	1.15	3	1.01	5.5742	92.07	2.918520	1.505376	4.68	5.1	6.9	2	1	1	2	2	0	2	2	45	3	151	72.5	31.79685	102.2	0.84
100925	3.08	1	2.17	5.765066	80.6172	3.455451	1.29026	6	5.5	4.98	2	3	1	2	2	0	2	2	45	4	168.4	74.4	26.23546	93.8	0.846
100251	1.85	5	1.5	5.3709	59.86176	3.004744	0.927267	5.12	5.5	0.72	2	1	1	2	2	0	2	2	45	4	171.9	122.9	41.59107	122.3	0.851
99099	1.64	2	1.14	6.22	37.92	3.388	1.739	5.33	5.5	6.79	1	1	1	1	2	0	2	2	45	1	156.3	91.4	37.41349	111	0.87
97909	4.33	4	1.06	5.94	95.64	3.336	1.784	5.22	5.9	1.95	2	3	1	2	2	0	2	2	45	6	161.7	89.4	34.19145	115.5	0.881
94208	3.67	5	1.19	5.11	49.68	2.146	0.621	3.62	6.1	3.56	2	3	1	2	2	0	2	2	45	4	160.4	118.4	46.01961	112.1	1
98144	2.43	4	1.22	5.38	55.5	3.129	1.287	4.94	5.4	7.46	2	1	1	2	2	0	2	2	45	3	157.1	58	23.50042	85.1	1.12
96532	1.14	4	1.4	5.7808	117.7341	3.06746	1.21004	5.02	6.1	7.18	2	1	1	2	2	0	1	2	45	3	161.8	105.1	40.14631	125.3	1.13
100286	1.4294	4	1.4	5.61	54	4.086	0.847	5.87	5.8	17.48	2	3	1	2	2	0	1	2	45	4	170.1	96.1	33.21351	120	1.2
99427	2.64	4	1.14	7.55	305.46	2.612	1.14	4.27	6.4	35.04	2	3	1	2	1	0	2	2	45	3	162.8	107.7	40.63562	134	1.22
98793	5	5	1.47	6.16	40.26	2.974	0.914	4.86	5.5	1.29	2	3	1	2	2	0	2	2	46	6	152.8	53.5	22.91433	78.1	0.425
95563	5	4	2.33	5.1216	53.5632	3.28061	1.10034	5.51	5	4.4	2	3	1	2	2	0	1	2	46	3	173	70.1	23.4221	85.6	0.464
96050	3.1	5	1.97	6	45.48	2.56	0.542	4.78	5.3	3.84	2	3	1	2	2	0	2	2	46	3	164.8	84.7	31.18667	98.5	0.479
96543	5	5	1.47	5.627177	105.64	3.033403	1.29876	5.04	5.7	2.09	2	3	1	2	2	0	2	2	46	4	159.6	63.4	24.88992	88.7	0.49
99494	0.68	2	1.24	5	48.78	2.25	1.208	4.03	5.2	3.07	2	1	1	2	2	0	2	2	46	1	157.2	71	28.73117	92.5	0.49
94457	4.81	5	1.73	5.3102	48.1098	2.390827	0.5668	4.29	5.6	0.7	2	3	1	2	2	0	2	2	46	3	164.6	67.7	24.98786	85.9	0.5

102758	0.64	3	2.04	5.6957	56.3496	3.637988	1.37425	6.15	5.6	0.86	2	3	1	2	2	0	2	2	46	6	155.4	55.8	23.10639	76.2	0.514
102786	5	5	1.11	9.4534	121.95	3.40832	1.80436	5.46	7.9	0.87	2	3	1	1	1	0	2	2	46	6	155.8	57.2	23.56466	84	0.54
93780	1.22	3	1.19	6.05	167.52	2.379	2.755	4.84	5.3	14.37	2	2	1	2	2	0	2	2	46	7	161.7	87.5	33.46478	104.4	0.575
97410	1.87176	1	1.78	5.66	28.92	2.276	0.553	4.32	5.4	0.42	2	2	1	2	2	0	2	2	46	1	151.8	46	19.96247	71.1	0.6
98933	5	5	1.84	4.94	20.76	3.103	0.723	5.28	5.5	1.09	2	3	1	2	2	0	2	2	46	3	163.7	52.9	19.74051	74	0.604
98770	3.88	4	1.66	6.2098	104.4162	2.12058	0.89458	3.47	6.3	8.1	2	3	1	2	2	0	1	2	46	4	167.1	146.2	52.35938	138.1	0.63
102766	0.88	2	1.73	6.16	25.08	2.896	1.106	5.15	5.2	1.35	2	1	1	2	2	0	2	2	46	6	150.8	47	20.66784	74.5	0.66
94740	2.49	5	1.06	5.58	98.5992	3.241669	1.8487	5.48	5.6	0.52	2	3	1	2	2	1	1	2	46	6	155.3	52.7	21.85082	78.2	0.684
99765	1.31	3	1.58	5.05	25.92	2.327	0.621	4.19	5.3	1.84	2	1	1	2	2	0	2	2	46	1	160	66.6	26.01563	86.8	0.689
99966	2.73	5	1.29	5.501833	62.8648	3.316455	1.122863	5.28	5.6	0.53	2	1	1	2	2	0	2	2	46	6	153.5	55.7	23.63951	80.4	0.69
94726	5	5	1.55	10.4	75.06	2.586	2.145	5.12	8.1	2.67	2	3	1	1	1	0	2	2	46	3	169	84	29.41073	101.7	0.73
101084	4.41	4	1.11	5.27	66.0599	2.78991	1.24616	4.73	5	0.43	2	3	1	2	2	0	1	2	46	3	171.4	67.3	22.90833	83	0.737
94105	5	4	1.68	5.33	146.22	3.181	0.926	5.28	5.3	12.95	2	3	1	2	2	0	1	2	46	1	162.7	74	27.95481	97.1	0.75
99242	1.73	3	0.59	5.767433	93.9456	3.47914	2.17894	5.51	5.5	1.74	2	1	1	2	2	0	2	2	46	1	160.4	69.4	26.97433	83.3	0.757
96573	5	5	1.89	6.11	35.7	2.586	0.452	4.68	5.3	1.03	2	3	1	2	2	0	2	2	46	3	165.8	75.2	27.35576	94.4	0.76
94008	0.955055	1	1.37	5.490085	79.9464	2.48052	0.890188	4.24	5.8	3.63	2	1	1	2	2	0	2	2	46	1	160.6	89.5	34.7002	115.2	0.76
100324	1.82	3	1.37	8.38	75.96	4.163	1.152	6.05	6.5	1.5	2	1	1	1	1	0	2	2	46	2	154.1	69.2	29.14076	86.2	0.79
94029	4.15	5	2.51	5.61	24.24	4.112	0.644	6.9	5.3	0.34	2	2	1	2	2	0	2	2	46	3	166.2	68.9	24.94349	85.8	0.814
98082	0.44	4	0.85	17.4	249.96	4.37	2.314	6.28	10.9	5.83	2	1	1	1	1	0	2	2	46	3	164	93.8	34.87507	106.6	0.838
97783	2.73	4	1.81	5.11	34.74	2.379	0.452	4.4	5.7	3.33	2	2	1	2	2	0	2	2	46	4	163.8	124.6	46.43979	127.6	0.91
100532	2.45	2	0.93	5.66	219.3	2.56	0.655	3.8	6.2	6.82	2	3	1	2	2	0	2	2	46	4	162.8	90.6	34.18373	114.6	0.911
101034	1.02	4	1.47	4.61	69.24	2.948	0.824	4.81	4.9	5.31	2	1	1	2	2	0	2	2	46	3	150.1	72.3	32.09053	105	0.915
101894	1.43	5	4.6	5.05	20.22	1.448	0.971	6.49	4.9	3.51	2	3	1	2	2	0	2	2	46	7	168.3	60.6	21.39461	88	0.94
98156	1.1	2	0.8	7.05	204.06	3.54222	5.295	5.53	5.4	2.09	2	2	1	2	1	0	2	2	46	1	154.4	72	30.20215	93.6	0.94
97145	3.8756	5	1.86	5.15675	36.26346	2.10712	0.774006	4.09	4.9	8.93	2	3	1	2	2	0	1	2	46	3	157.3	162.9	65.83603	124.2	1.02
97550	4.17	5	1.6	5.16	22.98	2.508	0.44	4.32	5	0.36	2	2	1	2	2	0	1	2	47	3	163.5	63.1	23.60445	80.2	0.42
102008	1.23	5	2.02	5.5	21.78	4.448	0.531	6.7	5.6	0.38	2	2	1	2	2	0	1	2	47	3	159.8	51.5	20.16757	69.3	0.447
96253	3.98	5	1.55	5.11	39.3	2.741	0.96	4.73	5	8.51	2	3	1	2	2	0	2	2	47	3	176.7	97	31.06695	114.3	0.45
102673	3.08	5	1.91	5.66	38.16	2.121	0.26	4.16	5.1	0.21	2	3	1	2	2	0	2	2	47	6	158.5	45.8	18.23085	71.4	0.517
97138	0.96	3	1.81	5.27	20.52	2.431	0.892	4.65	5.3	0.77	2	1	1	2	2	0	2	2	47	3	172.5	67.7	22.75152	81	0.53
93775	3.73	4	1.81	5.11	44.16	2.172	0.711	4.32	5.4	1.95	2	3	1	2	2	0	1	2	47	6	150.4	58.5	25.86189	87.5	0.636
97452	2.03	4	1.71	5.61	61.2	2.845	0.587	4.81	5.8	3.01	2	3	1	2	2	0	2	2	47	6	155	71.2	29.6358	94.5	0.646
99366	5	4	1.24	6.55	133.02	3.569	1.332	5.43	6	3.16	2	3	1	2	2	0	1	2	47	1	153.2	95.2	40.56201	123.8	0.67
96108	1.38	3	1.24	5.476157	100.759	3.41248	1.40292	5.48	5.4	3.73	2	1	1	2	2	1	2	2	47	1	161.6	98.3	37.64184	108.2	0.739
100115	3.33	2	1.86	5.94	37.74	3.672	0.813	5.9	5.4	1.27	2	3	1	2	2	0	2	2	47	3	159.6	72.4	28.42319	94	0.745
100055	5	5	1.71	5.8594	97.1568	3.6033	1.30268	5.82	6.2	3.55	2	3	1	2	2	0	2	2	47	6	153.1	76	32.42375	99.2	0.751
102759	5	4	3.26	4.94	23.46	1.371	0.7	4.94	5.4	2.2	2	3	1	2	2	0	1	2	47	3	178.9	69.6	21.74646	86.4	0.76
100119	3.79	5	1.22	5.55	171.06	3.595	1.389	5.46	5.2	2.83	2	3	1	2	2	0	2	2	47	3	162.5	116.1	43.96686	130.3	0.786
97509	2.47	3	1.22	5.3174	102.7564	2.118563	1.226093	3.75	4.9	6.97	2	3	2	2	2	0	1	2	47	3	160.7	101	39.11016	104.6	0.79
102201	0.6	3	1.42	5.33	15.3	2.353	1.287	4.37	5.2	3.76	2	1	1	2	2	0	2	2	47	4	172.4	61.5	20.69191	79.5	0.8
99226	1.5408	4	1.4	5.182511	61.452	3.083706	1.14002	5.15	5.1	2.04	2	1	1	2	2	0	1	2	47	2	157.8	73.6	29.55724	92.8	0.804
96432	5	5	1.22	6.49	93.06	3.879	1.502	5.79	5.7	1.21	2	3	1	1	2	0	2	2	47	7	160.7	56.4	21.83973	78.1	0.817
98846	0.58	2	1.5	5.05	30.66	2.56	0.35	4.22	5.4	0.95	2	1	1	2	2	0	2	2	47	3	169.2	83.3	29.09674	97.2	0.82
94685	2.02	4	1.19	5.5044	87.0348	2.46806	1.096003	4.19	5.4	4.2	2	1	1	2	2	0	2	2	47	4	164.2	103.8	38.49914	111.5	0.85
100319	1.66	3	1.24	14.2	204.6	1.784	1.953	3.93	9.7	7.64	2	3	1	1	1	0	2	2	47	3	165.3	119.2	43.62451	122.5	0.892
101307	0.77	3	1.68	6.11	94.26	3.62	0.903	5.72	6.1	5.21	2	1	1	2	2	0	2	2	47	4	170.3	124	42.75554	134.8	0.903
101171	1.590718	3	1.29	5.3279	101.8692	2.35406	0.975228	4.06	5.2	11.97	2	1	2	2	2	0	2	2	47	3	159.5	63.1	24.80322	92.5	0.91
97478	2	1	1.71	6.33	74.1	2.715	0.994	4.89	6.3	0.91	2	3	1	2	2	0	2	2	47	1	159.6	76.1	29.87575	96.4	0.933
95865	1.63	4	1.84	5.440466	77.2164	3.52994	1.126389	5.87	5.4	5.29	2	2	1	2	2	0	2	2	47	4	161.2	99.3	38.21371	112.5	0.969
95914	2.77	3	1.27	5.641045	87.9816	3.62722	1.49754	5.77	5.6	6.43	2	3	1	2	2	0	2	2	47	3	160.5	126.2	48.99021	135.6	0.976
101664	0.49	3	1.84	6.05	135.42	2.638	0.982	4.91	5.9	4.32	2	1	1	2	2	0	1	2	47	3	166.3	76.4	27.62542	102.7	1.01
101265	5	5	1.06	6.456	138.492	4.05754	1.84936	6.26	6.2	4.2	2	3	2	1	2	0	2	2	47	4	161.7	68.2	26.08341	88.2	1.02
96960	0	4	1.06	6.0924	144.0804	3.20327	1.60834	5.25	6.4	10.54	2	1	1	2	2	1	2	2	47	4	169.7	126.4	43.8918	129.7	1.1
97684	3.61	5	0.88	6.070111	167.9666	2.770266	1.66668	4.63	5.9	5.75	2	3	1	2	2	0	2	2	47	3	162.7	134.5	50.80976	145.7	1.1
100528	4.9	4	1.42	5.526	114.3744	3.51962	1.28136	5.51	5.5	12.26	2	3	1	2	2	0	1	2	47	3	167.4	133.8	47.7469	149.2	1.11
97418	1.743857	3	1.27	6.66	52.62	3.051	1.389	4.97	5.8	2.89	2	1	1	1	2	0	2	2	47	3	157	75.1	30.46777	102.2	1.13
94615	5	4	0.98	6.05	185.82	2.819	1.547	4.5	5.5	13.66	2	3	1	2	2	0	1	2	47	2	165	101.8	37.3921	121.9	1.21
100698	5	5	1.29	5.33	23.58	2.767	0.689	4.37	5.1	0.49	2	2	1	2	2	0	2	2	48	6	163.7	60.8	22.68852	80	0.429
99811	1.094657	2	1.47	5.77	65.82	2.664	1.197	4.68	6	1.7	2	1	1	2	2	0	2	2	48	1	149.8	76.5	34.09085	108.5	0.501
98832	1.96	5	1.32	5.83	46.8	2.922	0.79	4.6	5.6	0.79	2	1	1	2	2	0	2	2	48	4	171.9	79.5	26.90391	89.9	0.517

98926	0.64	7	1.94	5.486748	71.38236	4.00992	1.23071	7.71	5.3	0.48	2	2	1	2	2	0	2	2	48	6	152.6	50.9	21.85791	75.3	0.521
94623	5	5	1.99	5.311133	46.6869	2.449143	0.49742	4.53	5.4	0.86	2	3	1	2	2	0	2	2	48	3	166.5	66.2	23.87974	95.7	0.57
97333	3.85	5	1.55	5.595257	63.89376	3.893769	1.169667	6.26	5.4	0.55	2	3	2	2	2	0	2	2	48	6	163.1	55	20.67544	76.6	0.58
99599	5	5	1.71	5.66	24.06	3.077	1.05	5.28	5.8	1.02	2	3	1	2	2	0	2	2	48	3	180.4	90.6	27.8391	93.6	0.591
94385	2.45	1	1.34	5.4256	66.9108	2.763006	0.801653	4.47	5.4	1.14	2	1	1	2	2	0	2	2	48	6	165.6	72.5	26.43731	87.2	0.6
101194	5	5	1.63	5.1938	60.34971	2.242377	0.633428	4.11	5	1	2	3	2	2	2	0	2	2	48	3	168.3	91.8	32.40966	108.8	0.606
100436	0.93	4	1.24	6.44	165.48	2.276	1.626	4.27	5	5.03	2	1	1	2	2	0	1	2	48	1	149.7	112.7	50.28985	125.4	0.64
96534	2.323283	2	2.07	5.4122	55.87256	2.614286	0.61142	4.78	5.4	0.72	2	3	1	2	2	0	2	2	48	4	159.2	60.8	23.98929	81.8	0.67
101438	2.89	5	1.37	5.3939	66.17688	2.772344	0.89686	4.5	5.4	3.99	2	3	1	2	2	0	2	2	48	2	162.2	71.9	27.32922	87.2	0.718
98320	2.694145	4	1.22	5.55	116.82	4.784	1.14	6.52	6	4.44	2	3	1	2	2	0	1	2	48	4	162.9	97.8	36.85502	106	0.724
94879	4.08	4	1.37	4.94	45.18	2.87	0.655	4.55	5.4	0.41	2	3	1	2	2	0	2	2	48	7	175.1	99.1	32.32223	107.2	0.744
100765	3.51875	3	1.24	5.921861	113.6964	2.785136	1.1306	4.5	5.8	2.99	2	3	1	2	2	0	2	2	48	6	154.6	56.5	23.63902	83	0.75
93841	5	5	1.73	5.6328	58.8144	3.55428	0.790327	5.61	5.7	3.19	2	3	1	2	2	0	2	2	48	7	171.5	96.5	32.80946	108.3	0.76
98923	0.4	4	1.47	5.5924	85.344	3.66542	1.13928	5.69	5.8	5.93	2	1	1	2	2	0	1	2	48	4	159.8	104.7	41.00088	120	0.76
97289	4.86	4	2.33	5.3514	47.2192	2.60774	0.58514	4.91	5.7	0.92	2	3	1	2	2	0	2	2	48	3	161.7	75	28.6841	92	0.8
97555	1.99	3	1.06	5.61	81.24	1.319	0.373	2.56	5	0.9	2	3	2	2	2	0	1	2	48	1	160.3	76.5	29.77107	99.6	0.85
94295	3.82	2	1.29	5.5924	105.5988	3.365	1.5632	5.43	5.3	10.63	2	3	1	2	2	0	1	2	48	6	157.4	81.3	32.81566	99.8	0.856
96358	1.66	4	1.27	5.749	118.6771	2.63674	0.881	4.34	5.9	3.19	2	3	1	2	2	0	1	2	48	3	151.1	66.4	29.083	106	0.86
102819	4.81	3	1.24	5.5827	110.8392	2.796550	1.210413	4.53	5.5	2.76	2	3	1	2	2	0	2	2	48	3	164.6	70.9	26.16897	91.2	0.88
95789	3.65	4	1.63	5.136666	45.8716	2.739429	0.69698	4.71	5.2	0.87	2	3	1	2	2	0	2	2	48	3	166	78.6	28.52373	101.1	0.88
95274	3.03	2	1.01	7.16	263.82	1.862	1.366	3.49	6.3	5.61	2	3	1	1	1	0	2	2	48	3	162.9	115.8	43.63816	128.2	0.96
102218	1.54	4	1.5	5.38	30	5.069	0.824	6.96	5	8.3	2	2	1	2	2	0	1	2	48	4	165.7	61.6	22.4355	91.4	0.97
94260	0.32	2	1.16	8.293890	149.0832	2.63998	1.69398	4.42	6.3	5.91	2	1	1	1	1	0	2	2	48	6	147	74.6	34.52265	105.2	0.99
99111	0.88	2	3.05	5.017	34.476	2.301351	0.582326	4.47	5.1	0.36	2	1	1	2	2	0	2	2	48	4	166.3	66.9	24.19032	89.7	1.02
95244	1.46	3	1.06	8.063490	218.5908	2.29218	1.41414	4.11	7.4	9.4	2	3	1	1	1	0	2	2	48	4	164.5	75.2	27.78984	92.1	1.02
98300	2.44	4	1.45	6.38	63.3	2.922	0.971	4.81	5.6	2.49	2	3	1	2	2	0	2	2	48	3	163	91.8	34.55155	109	1.04
99661	1.2	4	1.97	5.258898	46.5876	3.888695	0.94774	6.9	5.2	1.05	2	3	1	2	2	0	2	2	48	4	158.3	62	24.74172	90	1.08
93834	0.28	2	0.8	18.2	124.86	4.613574	4.934	6.9	11.5	29	2	2	1	2	2	0	2	2	48	3	161.7	95.4	36.48617	125.7	1.09
102943	4.31	4	1.5	5.7624	101.2788	3.60554	1.15856	5.77	5.7	9.57	2	3	1	2	2	0	1	2	48	1	181.1	122.7	37.41172	122.9	1.09
96137	0.53	4	0.83	8.283400	159.6984	3.11712	2.21668	5.28	6.4	7.34	2	1	1	1	1	1	2	2	48	3	159.8	93.4	36.57576	101	1.17
99260	4.62	4	1.4	7.72	196.08	2.612	2.314	5.07	6.4	19.21	2	3	1	1	1	0	2	2	48	3	159.2	100.3	39.57444	128.5	1.4
99838	2.79	5	1.01	6.2806	175.5372	3.056155	1.92384	5.12	5.4	104.48	2	3	1	2	2	0	2	2	48	3	152.4	83.7	36.03757	113.9	2.02
93949	5	4	1.03	9.405	162.2088	2.91752	1.92476	5.09	8	0.83	2	3	1	1	1	0	2	2	49	6	150.7	55.9	24.61418	90.5	0.418
97641	2.886516	4	1.58	5.3418	59.72892	2.840390	0.593593	4.6	5.7	5.11	2	3	1	2	2	0	2	2	49	3	161.9	64.2	24.49296	84.1	0.498
97683	4.45	1	1.29	5.3597	66.71504	2.910475	1.0294	4.73	5	0.91	2	3	1	2	2	0	2	2	49	1	155	66.2	27.55463	92.3	0.59
96970	2.04	3	1.24	7.22	21.78	1.965	0.858	3.59	6.3	0.5	2	1	1	2	1	0	2	2	49	6	153.2	51.1	21.77225	77.4	0.62
94139	1.15	4	1.66	5.55	45.54	2.974	0.7	4.94	5.3	5.51	2	3	1	2	2	0	2	2	49	2	163.3	85.4	32.02473	113.2	0.74
94137	4.86	5	1.66	5.5246	45.676	2.79715	0.75023	4.73	5.6	1.35	2	3	1	2	2	0	2	2	49	4	153.3	57.5	24.46716	79.8	0.741
95968	1.67	5	1.84	5.83	20.4	2.198	0.452	4.24	5.7	13.33	2	3	1	2	2	0	1	2	49	6	157.5	50.1	20.19652	71	0.794
97365	1.75	3	1.24	5.271666	75.0696	3.186955	1.331168	5.15	5.2	5.39	2	1	1	2	2	0	2	2	49	3	154	52.4	22.09479	77.2	0.801
101593	0.7	1	1.06	5.4586	71.75093	2.176551	0.98586	3.83	5	1.06	2	1	2	2	2	0	2	2	49	6	147.5	74	34.01321	101.2	0.808
100415	5	4	1.86	5.399533	85.4292	3.59255	1.070969	5.79	5.4	4.97	2	3	1	2	2	0	1	2	49	4	159.4	71.5	28.14034	93.5	0.82
101160	3.47	3	1.29	5.27	70.26	4.112	1.795	6.23	5.2	5.78	2	1	2	2	2	0	2	2	49	1	151.4	65.7	28.66247	88.2	0.832
100717	0.82	4	1.14	6.55	52.86	2.353	1.061	3.98	6.2	13.2	2	1	1	2	2	1	2	2	49	3	168.5	120.1	42.30028	131.4	0.86
94427	1.94	5	0.88	5.498066	126.2376	2.21362	1.47382	3.98	5.5	5.9	2	1	1	2	2	0	2	2	49	6	155.2	89.8	37.28146	102.8	0.89
101386	1.37	4	0.91	5.5716	120.0465	3.8278	1.7422	5.72	5.7	7.3171	2	1	1	2	2	0	1	2	49	2	161.8	67	25.5928	98.5	0.904
98524	2.24	3	1.29	7.584090	167.9652	4.117504	1.63308	6.28	6.3	4.58	2	3	1	1	1	0	2	2	49	4	159.1	91.5	36.14771	106.8	0.914
99257	1.78	3	1.22	6.22	129.12	2.767	1.344	4.6	5.8	5.46	2	3	1	2	2	0	2	2	49	1	155	78.9	32.84079	120	0.915
101875	5	5	1.78	5.452214	100.5006	4.6016	1.51714	8.3	5.6	4.16	2	3	1	2	2	1	2	2	49	6	151.5	65.7	28.62464	90.9	0.94
102081	1.27	3	1.4	4.61	60.96	3.155	2.789	5.82	5.5	0.96	2	1	1	2	2	0	2	2	49	3	155.7	97.3	40.13614	114.2	0.94
102204	1.83	3	1.06	4.94	23.04	4.04502	5.668	9.1	5.7	3.67	2	2	1	2	2	0	2	2	49	3	170.9	78.9	27.01425	103.3	1.03
101106	5	4	1.66	5.94	86.22	3.957	1.287	6.21	5.7	3.64	2	3	1	2	2	0	1	2	49	4	166.3	108.7	39.30475	112.4	1.03
101861	5	4	1.97	5.27	49.92	2.302	0.35	4.42	5.4	1.39	2	3	1	2	2	0	1	2	49	3	160.8	68.2	26.3762	86.1	1.06
100348	5	4	1.24	5.88	37.74	3.336	0.621	4.86	5.8	4.53	2	1	1	2	2	0	2	2	49	4	156.1	84.8	34.80087	102.5	1.06
98046	0.64	2	1.11	6.88	125.88	2.87	1.242	4.55	5.9	19.95	2	1	1	2	2	0	2	2	49	3	164.8	103.1	37.96158	119.7	1.11
100693	4.98	5	1.76	9.71	132.18	2.431	1.637	4.94	8	6.07	2	3	1	1	1	0	2	2	49	4	163.7	97.1	36.23447	118.2	1.16
96651	2.78205	4	1.58	5.66	57.9	3.103	0.519	4.91	5.7	14.31	2	3	1	2	2	0	2	2	49	4	163.9	110.4	41.0971	121.4	1.32
97878	4.07	4	2.15	10.2	60.06	3.103	0.892	5.66	8.2	26	2	3	1	2	1	0	1	2	49	4	163.2	82.3	30.90007	107.9	1.43
102238	0.82	4	1.27	5.601	99.5688	2.2142	0.93326	3.93	5.6	18.6	2	1	2	2	2	0	2	2	49	3	162	148.4	56.54626	148.5	1.5
101052	1.093266	1	1.22	5.83	210.42	3.491	1.321	5.3	6.1	90.51	2	1	1	2	2	1	2	2	49	4	167.2	108.9	38.95429	119.1	1.57
